# Supplementary material for: ALX1‐related frontonasal dysplasia results from defective neural crest cell development and migration
Source: EMBO Mol Med. 2020 Sep 11;12(10):e12013. doi: 10.15252/emmm.202012013 (PMC7539331; doi:10.15252/emmm.202012013)
Supplement: Supplementary file 1 — Expanded View Figures PDF [file EMMM-12-e12013-s001.pdf]

Expanded View Figures

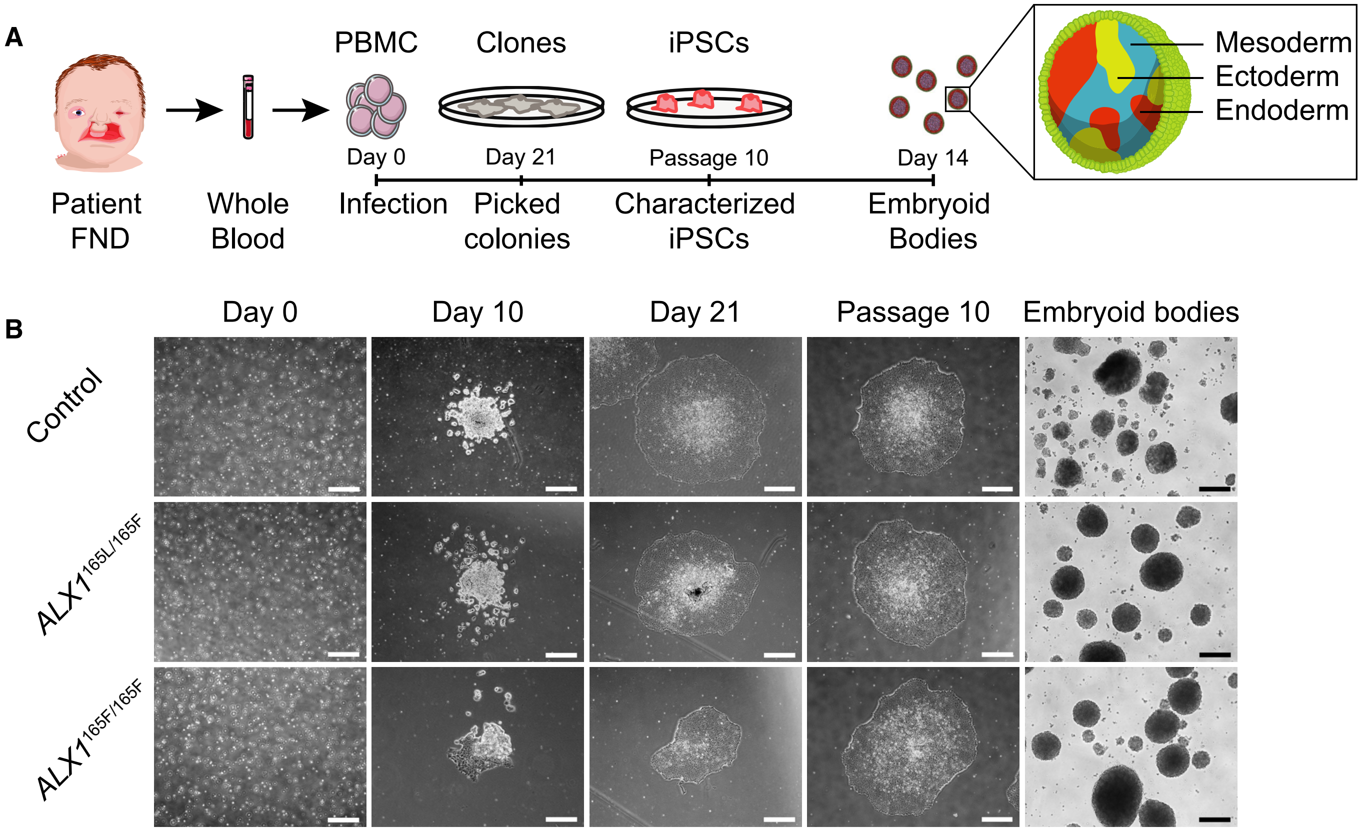

Figure EV1. Induced pluripotent stem cells (iPSC) derivation and EB generation.

A Schematic representation of the strategy used to generate iPSC. Blood samples from an unrelated normal individual, unaffected father (subject 1), and two of the affected children (subjects 5 and 6) were processed. Isolated PBMC were infected with Sendai virus, and individual clones were picked 21 days after the infection. Following expansion until passage 10, iPSC were characterized and embryoid bodies were formed by suspension culture for 14 days.

B The reprogramming process of the PBMC showed that all cells underwent similar morphological changes leading to the formation of iPSC clones by day 21. These clones still displayed embryonic stem cells morphology at passage 10, indicating that the cells are able to self-renew. All iPSC clones were able to form EBs. One clone of each subject is represented. Scale bar is 400 μm.

© 2020 The Authors

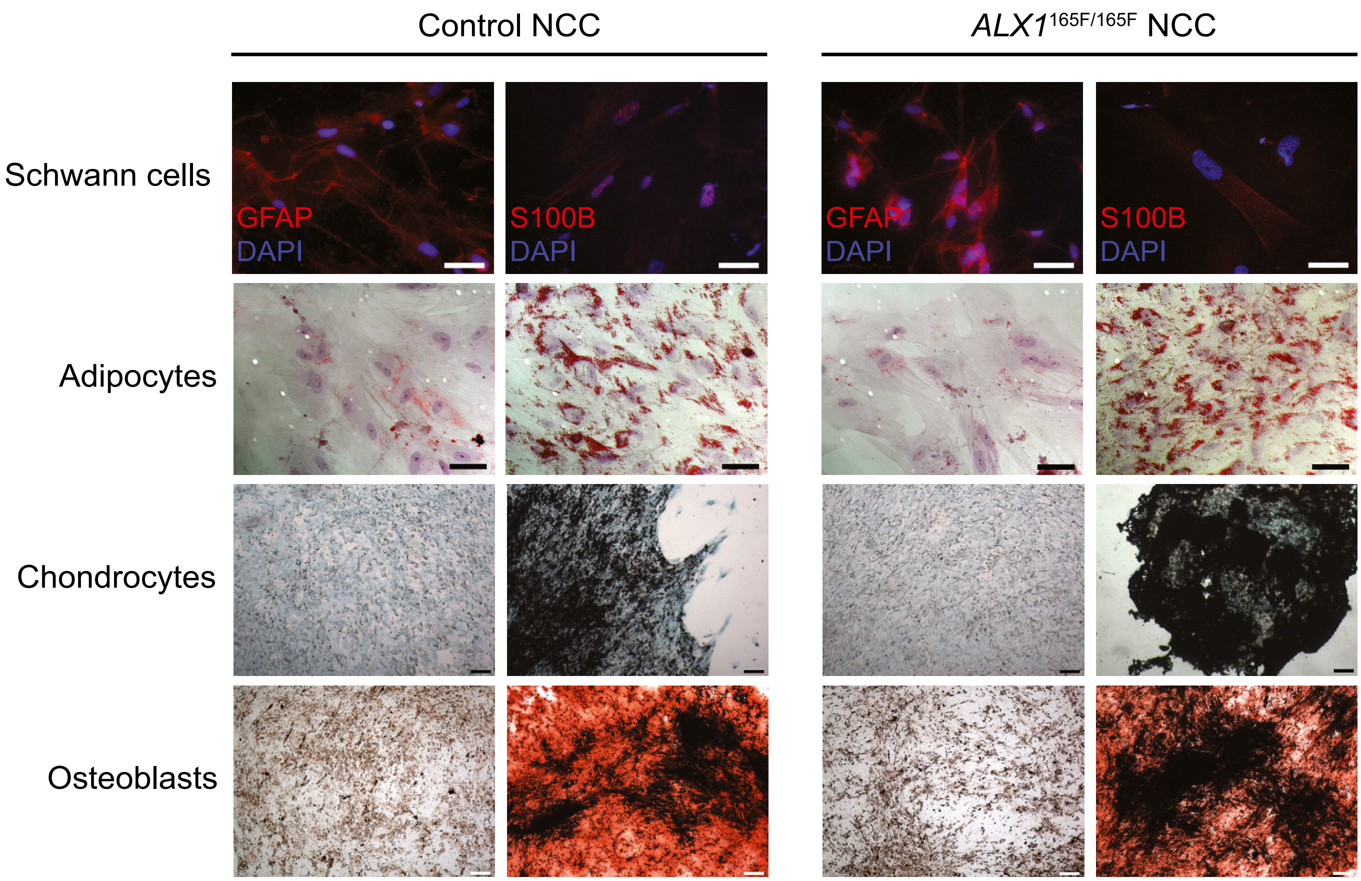

Figure EV2. Characterization of NCC.

Multilineage differentiation experiments revealed that both control and subject-derived NCC are able to differentiate into Schwann cells, shown by the GFAP and S100B-positive immunofluorescence staining; adipocytes, demonstrated by the Oil Red O. positive lipidic droplets; osteoblasts, shown by Alizarin Red S. positive mineralized nodules and chondrocytes, assessed by Alcian Blue-positive cartilaginous matrix. Scale bar is 200 μm for images of Schwann cells and adipocytes, and 400 μm for images of chondrocytes and osteoblasts.

© 2020 The Authors

Figure EV3. Effect of BMP2 and CV2 on NCC migration.

A 10, 50, or 100 ng/ml of soluble BMP2 was added to the culture medium. Surface area analyses and percentages of coverage were measured using ImageJ software (NIH). The data of NCC migration following the treatment with 10, 50, and 100 ng/ml soluble BMP2 are represented as the average of the percentage of closure ± SEM from three independent experiments performed with each clone. To test statistical significance, an ANOVA test was performed. A *P*-value < 0.05 was considered to be statistically significant *: Significantly different from untreated *ALX1*$^{165F/165F}$ NCC: at 12 h, *P* = 0.0038 when comparing BMP2 50 ng/ml and untreated *ALX1*$^{165F/165F}$ NCC, and *P* = 0.0045 when comparing BMP2 100 ng/ml and untreated *ALX1*$^{165F/165F}$ NCC. At 18 h, *P* = 0.0337 when comparing BMP2 10 ng/ml and untreated *ALX1*$^{165F/165F}$ NCC; *P* = 0.0009 when comparing BMP2 50 ng/ml and untreated *ALX1*$^{165F/165F}$ NCC; and *P* = 0.0006 when comparing BMP2 100 ng/ml and untreated *ALX1*$^{165F/165F}$ NCC. At 24 h, *P* = 0.005 when comparing BMP2 10 ng/ml and untreated *ALX1*$^{165F/165F}$ NCC; *P* < 0.0001. when comparing BMP2 50 ng/ml and untreated *ALX1*$^{165F/165F}$ NCC; and *P* < 0.0001 when comparing BMP2 100 ng/ml and untreated *ALX1*$^{165F/165F}$ NCC.

B 10, 50, or 100 ng/ml of soluble CV2 was added to the culture medium. Surface area analyses and percentages of coverage were measured using ImageJ software (NIH). The data of NCC migration following the treatment with 10, 50, and 100 ng/ml soluble CV2 are represented as the average of the percentage of closure ± SEM. Scale bar = 400 μm. *: Significantly different from untreated *ALX1*$^{165F/165F}$ NCC: at 12 h, *P* = 0.0146 when comparing CV2 50 ng/ml and untreated *ALX1*$^{165F/165F}$ NCC, and *P* = 0.0262 when comparing CV2 100 ng/ml and untreated *ALX1*$^{165F/165F}$ NCC. At 18 h, *P* = 0.0028 when comparing CV2 50 ng/ml and untreated *ALX1*$^{165F/165F}$ NCC, and *P* = 0.0035 when comparing CV2 100 ng/ml and untreated *ALX1*$^{165F/165F}$ NCC. At 24 h, *P* = 0.0002 when comparing CV2 50 ng/ml and untreated *ALX1*$^{165F/165F}$ NCC, and *P* < 0.0001 when comparing CV2 100 ng/ml and untreated *ALX1*$^{165F/165F}$ NCC.

C Recovery of subject-derived NCC migration in a migration assay following the combined treatment with 100 ng/ml each of soluble BMP2 and CV2. The data are represented as the average of the percentage of closure ± SEM from three independent experiments performed with each clone. To test statistical significance, an ANOVA test was performed. A *P*-value < 0.05 was considered to be statistically significant * : Significantly different from *ALX1*$^{165F/165F}$ NCC: at 12 h, *P* = 0.0031, at 18 h *P* = 0.0001, and at 24 h *P* < 0.0001).

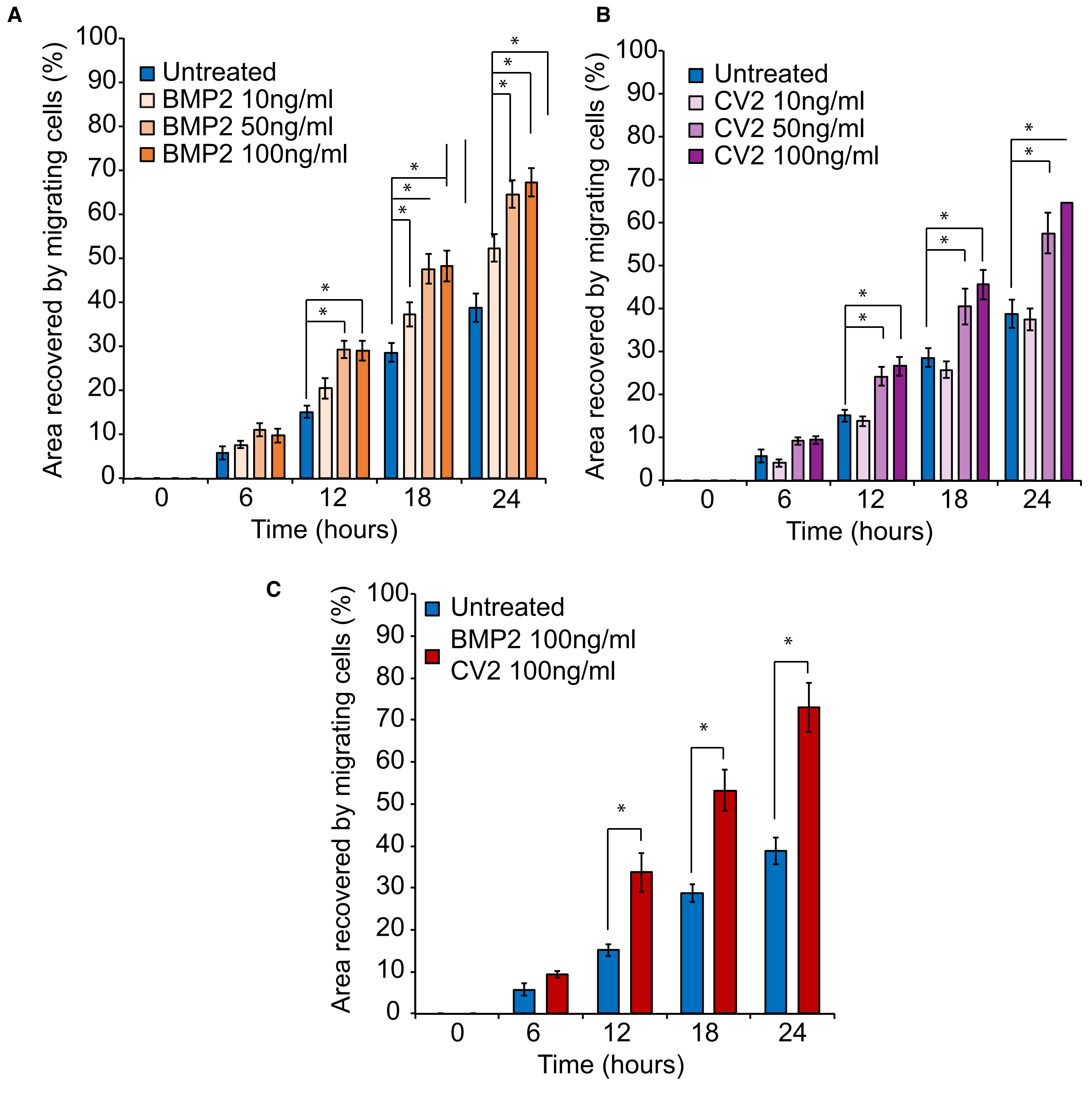

Figure EV3.

 © 2020 The Authors

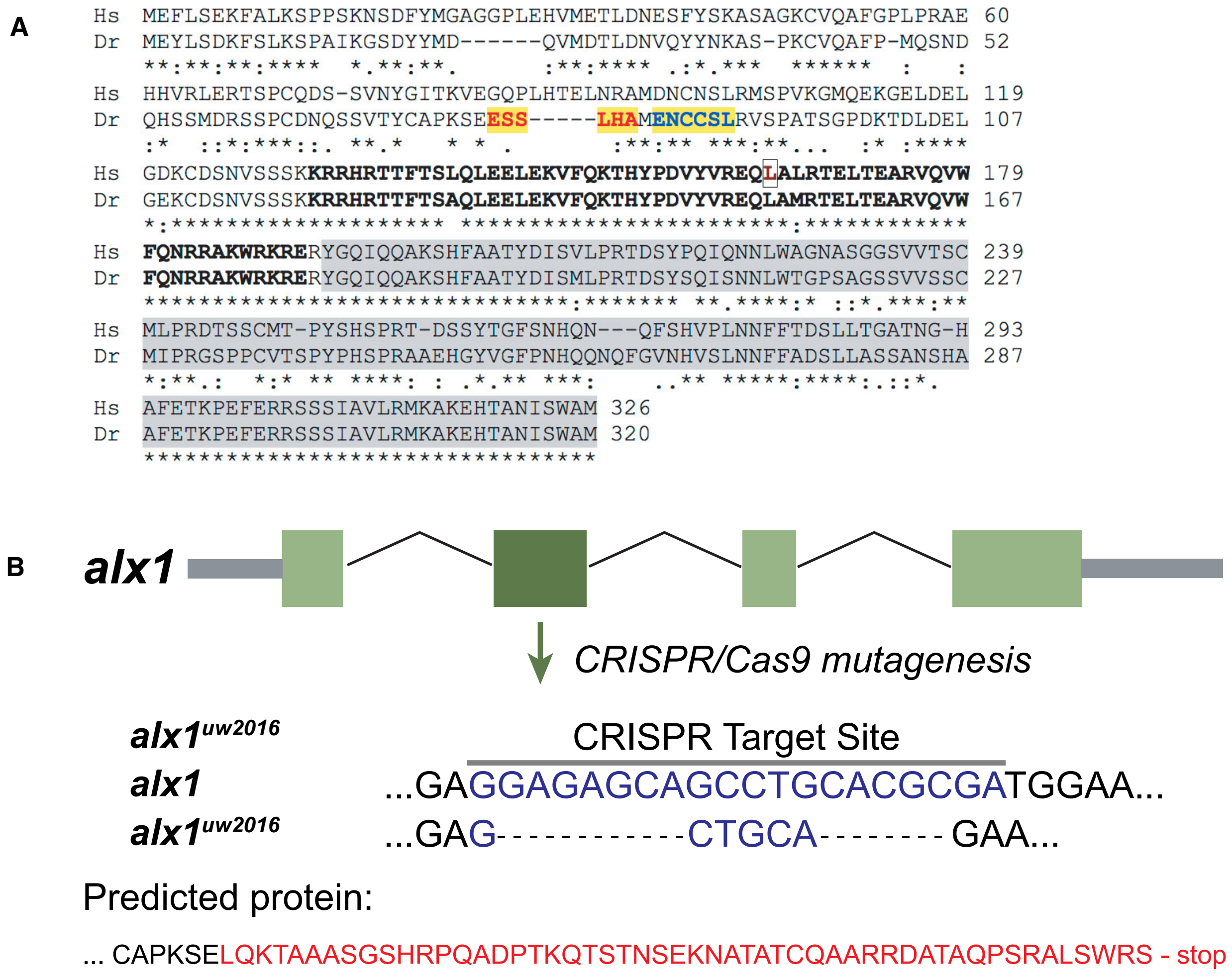

Figure EV4. CRISPR/Cas9 targeted mutagenesis of *alx1* in zebrafish.

A Human ALX1 and zebrafish alx1 protein sequences were obtained from Ensembl and aligned using Clustal Omega (https://www.ebi.ac.uk/Tools/msa/clustalo/) under the default settings. The homeobox DNA-binding domain is shown in bold, with the amino acid residue mutated in the subjects indicated by an outline. The transactivation domain is shaded in gray. Zebrafish *alx1* CRISPR sites #1 and #2 are highlighted in yellow. The red and blue letters visually demarcate the sites.

B Schematic diagram shows the effect of the mutant allele resulting from our choice of target site #1. The allele, termed, $alx1^{uw2016}$, has a net deletion of 16 nucleotides. Red letters denote the abnormal sequence that results from the frameshift mutation.

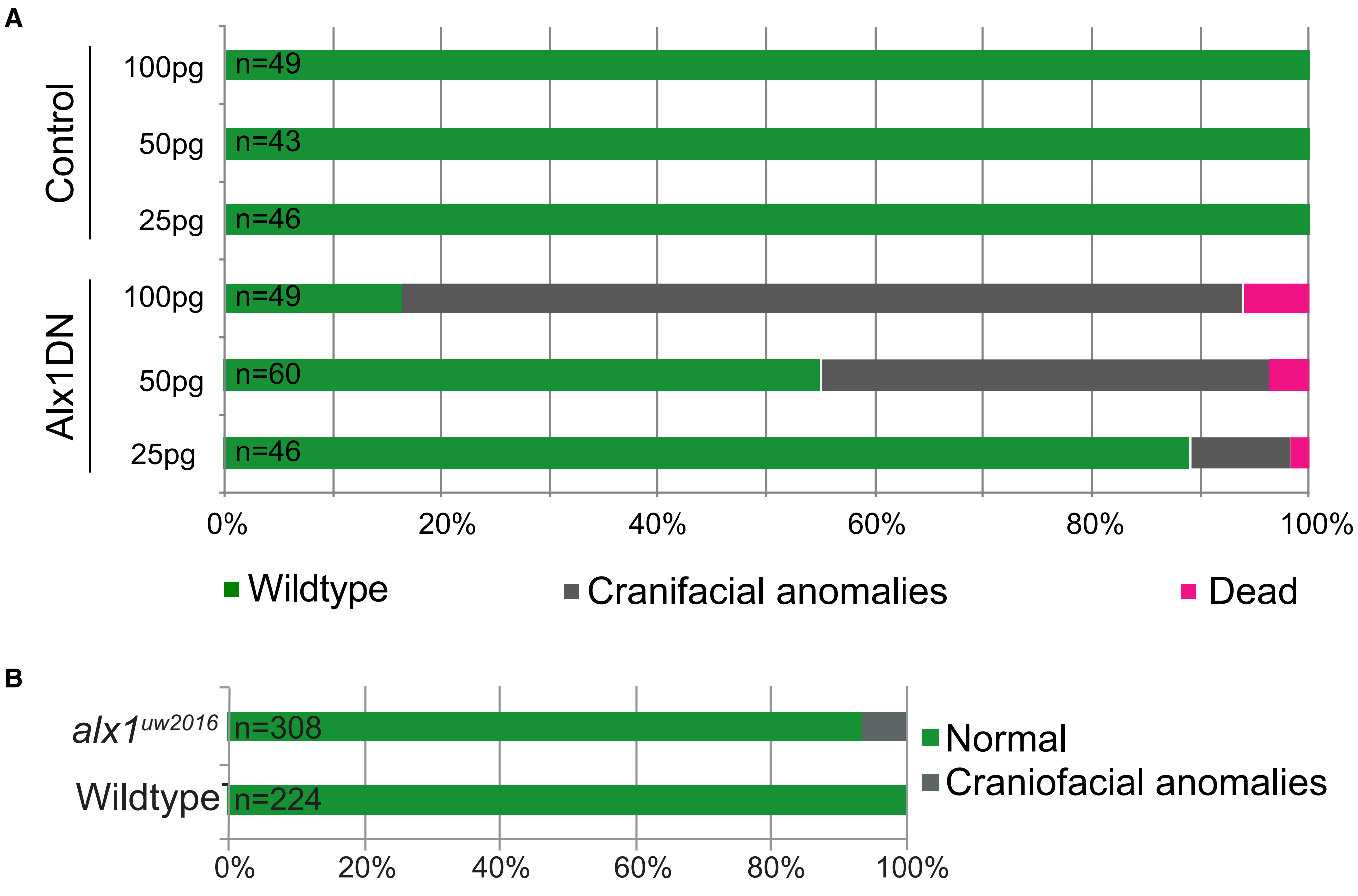

Figure EV5. Qualitative and quantitative characterization of zebrafish mutants.

A The number of embryos displaying craniofacial phenotypes increased with increasing concentration of Alx1DN mRNA injected into the single cell stage embryo. Overview of the relationship of the results of injections of 25, 50, and 100 pg of control mRNA and Alx1DN mRNA with the outcomes of wild-type zebrafish (green), a craniofacial phenotype (gray), and dead zebrafish (magenta).

B The number of embryos displaying craniofacial phenotypes injected with *alx1*^{uw2016} mRNA was very low. Overview of the percent of injected wild-type zebrafish displaying a craniofacial phenotype (gray), compared with uninjected wild-type zebrafish from the identical clutches (gray). Data of the injections are presented as a comparative percentage.

© 2020 The Authors
