# Supplementary material for: ALX1‐related frontonasal dysplasia results from defective neural crest cell development and migration
Source: EMBO Mol Med. 2020 Sep 11;12(10):e12013. doi: 10.15252/emmm.202012013 (PMC7539331; doi:10.15252/emmm.202012013)
Supplement: Supplementary file 2 — Table EV1 [file EMMM-12-e12013-s002.docx]

**Table Expanded View (EV)1:** P-values for Figure 3.

*: Significant differences when compared to undifferentiated iPSC.

| ***ALX1*** | | | | | | | |
| --- | --- | --- | --- | --- | --- | --- | --- |
| **Day** | **2** | **4** | **6** | **8** | **10** | **12** | **14** |
|  | ***ALX1*^165F/165F^** | | | | | | |
| **Undifferentiated** | p=0.0024 | p=5e^-11^ | p=8e^-13^ | p=4e^-11^ | p=4e^-8^ | p=7e^-13^ | p=6e^-14^ |
|  | ***ALX1*^165L/165F^** | | | | | | |
| **Undifferentiated** | NS | p=0.000001 | p=5e^-10^ | p=1e^-7^ | p=1e^-8^ | p=6e^-9^ | p=2e^-7^ |
|  | **Control (*ALX1*^165L/165L^)** | | | | | | |
| **Undifferentiated** | NS | NS | p=4e^-8^ | p=7e^-13^ | p=0.0002 | p=0.00002 | p=1e^-6^ |

| ***ZIC1*** | | | | | | | |
| --- | --- | --- | --- | --- | --- | --- | --- |
| **Day** | **2** | **4** | **6** | **8** | **10** | **12** | **14** |
|  | ***ALX1*^165F/165F^** | | | | | | |
| **Undifferentiated** | p=0.0005 | p=0.0245 | p=0.0158 | p=0.0029 | NS | NS | NS |
|  | **ALX1^165L/165F^** | | | | | | |
| **Undifferentiated** | p=0.0261 | NS | NS | NS | NS | NS | NS |
|  | **Control (ALX1^165L/165L^)** | | | | | | |
| **Undifferentiated** | p=0.0016 | NS | NS | NS | NS | NS | NS |

| ***PAX7*** | | | | | | | |
| --- | --- | --- | --- | --- | --- | --- | --- |
| **Day** | **2** | **4** | **6** | **8** | **10** | **12** | **14** |
|  | ***ALX1*^165F/165F^** | | | | | | |
| **Undifferentiated** | p=0.0163 | p=0.0076 | p=0.0092 | p=0.0136 | NS | NS | NS |
|  | **ALX1^165L/165F^** | | | | | | |
| **Undifferentiated** | NS | p=0.0257 | NS | NS | NS | NS | NS |
|  | **Control (ALX1^165L/165L^)** | | | | | | |
| **Undifferentiated** | NS | p=0.0425 | NS | NS | NS | NS | NS |

| ***PAX3*** | | | | | | | |
| --- | --- | --- | --- | --- | --- | --- | --- |
| **Day** | **2** | **4** | **6** | **8** | **10** | **12** | **14** |
|  | ***ALX1*^165F/165F^** | | | | | | |
| **Undifferentiated** | p=0.00008 | p=1e^-7^ | p=4e^-9^ | p=1e^-6^ | p=0.0329 | p=0.0008 | p=0.0108 |
|  | **ALX1^165L/165F^** | | | | | | |
| **Undifferentiated** | NS | p=0.0024 | p=0.0036 | p=0.0007 | p=0.0456 | p=0.0247 | p=0.0387 |
|  | **Control (ALX1^165L/165L^)** | | | | | | |
| **Undifferentiated** | NS | p=5e^-6^ | p=6e^-10^ | p=3e^-10^ | p=0.0068 | p=0.0034 | NS |

| ***MSX1*** | | | | | | | |
| --- | --- | --- | --- | --- | --- | --- | --- |
| **Day** | **2** | **4** | **6** | **8** | **10** | **12** | **14** |
|  | ***ALX1*^165F/165F^** | | | | | | |
| **Undifferentiated** | p=2e^-7^ | p=3e^-15^ | p=1e^-12^ | p=2e^-14^ | p=1e^-15^ | p=5e^-11^ | p=13e^-14^ |
|  | **ALX1^165L/165F^** | | | | | | |
| **Undifferentiated** | NS | p=1e^-7^ | p=0.0004 | p=1e^-7^ | p=3e^-9^ | p=1e^-10^ | p=7e^-9^ |
|  | **Control (ALX1^165L/165L^)** | | | | | | |
| **Undifferentiated** | NS | p=1e^-6^ | p=8e^-12^ | p=6e^-11^ | p=3e^-9^ | p=2e^-9^ | p=1e^-9^ |

| ***MSX2*** | | | | | | | |
| --- | --- | --- | --- | --- | --- | --- | --- |
| **Day** | **2** | **4** | **6** | **8** | **10** | **12** | **14** |
|  | ***ALX1*^165F/165F^** | | | | | | |
| **Undifferentiated** | p=1e^-15^ | p=7e^-13^ | p=1e^-10^ | p=8e^-15^ | p=6e^-12^ | p=1e^-15^ | p=1e^-14^ |
|  | **ALX1^165L/165F^** | | | | | | |
| **Undifferentiated** | p=9e^-12^ | p=1e^-15^ | p=6e^-8^ | p=8e^-13^ | p=1e^-15^ | p=3e^-12^ | p=1e^-15^ |
|  | **Control (ALX1^165L/165L^)** | | | | | | |
| **Undifferentiated** | NS | p=3e^-6^ | p=7e^-6^ | p=2e^-8^ | p=0.0007 | p=0.0002 | p=0.0011 |

| ***DLX5*** | | | | | | | |
| --- | --- | --- | --- | --- | --- | --- | --- |
| **Day** | **2** | **4** | **6** | **8** | **10** | **12** | **14** |
|  | ***ALX1*^165F/165F^** | | | | | | |
| **Undifferentiated** | p=1e^-8^ | p=3e^-15^ | p=2e^-10^ | p=1e^-15^ | p=1e^-14^ | p=6e^-11^ | p=8e^-12^ |
|  | **ALX1^165L/165F^** | | | | | | |
| **Undifferentiated** | p=7e^-7^ | p=1e^-11^ | p=1e^-8^ | p=3e^-9^ | p=1e^-9^ | p=6e^-12^ | p=7e^-8^ |
|  | **Control (ALX1^165L/165L^)** | | | | | | |
| **Undifferentiated** | NS | p=0.0008 | p=0.0048 | p=0.0186 | NS | NS | NS |

| ***FOXD3*** | | | | | | | |
| --- | --- | --- | --- | --- | --- | --- | --- |
| **Day** | **2** | **4** | **6** | **8** | **10** | **12** | **14** |
|  | ***ALX1*^165F/165F^** | | | | | | |
| **Undifferentiated** | p=0.0182 | NS | NS | NS | NS | NS | NS |
|  | **ALX1^165L/165F^** | | | | | | |
| **Undifferentiated** | p=1e^-6^ | NS | NS | NS | NS | NS | NS |
|  | **Control (ALX1^165L/165L^)** | | | | | | |
| **Undifferentiated** | p=0.0013 | NS | NS | NS | NS | NS | NS |

| ***P75*** | | | | | | | |
| --- | --- | --- | --- | --- | --- | --- | --- |
| **Day** | **2** | **4** | **6** | **8** | **10** | **12** | **14** |
|  | ***ALX1*^165F/165F^** | | | | | | |
| **Undifferentiated** | p=0.0002 | NS | NS | NS | NS | NS | NS |
|  | **ALX1^165L/165F^** | | | | | | |
| **Undifferentiated** | p=0.0210 | NS | NS | NS | NS | NS | NS |
|  | **Control (ALX1^165L/165L^)** | | | | | | |
| **Undifferentiated** | p=0.0082 | NS | NS | NS | NS | NS | NS |

| ***TFAP2A*** | | | | | | | |
| --- | --- | --- | --- | --- | --- | --- | --- |
| **Day** | **2** | **4** | **6** | **8** | **10** | **12** | **14** |
|  | ***ALX1*^165F/165F^** | | | | | | |
| **Undifferentiated** | p=2e^-8^ | p=3e^-12^ | p=5e^-12^ | p=1e^-13^ | p=3e^-10^ | p=5e^-10^ | p=7e^-10^ |
|  | **ALX1^165L/165F^** | | | | | | |
| **Undifferentiated** | p=5e^-9^ | p=9e^-12^ | p=2e^-10^ | p=1e^-15^ | p=7e^-14^ | p=1e^-15^ | p=1e^-14^ |
|  | **Control (ALX1^165L/165L^)** | | | | | | |
| **Undifferentiated** | NS | p=0.00009 | p=0.0126 | p=2e^-7^ | p=0.0001 | p=0.0001 | NS |

| ***SNAI2*** | | | | | | | |
| --- | --- | --- | --- | --- | --- | --- | --- |
| **Day** | **2** | **4** | **6** | **8** | **10** | **12** | **14** |
|  | ***ALX1*^165F/165F^** | | | | | | |
| **Undifferentiated** | p=0.0000009 | p=1e^-15^ | p=7e^-12^ | p=6e^-14^ | p=1e^-15^ | p=3e^-11^ | p=1e^-14^ |
|  | **ALX1^165L/165F^** | | | | | | |
| **Undifferentiated** | p=0.0211 | p=1e^-8^ | p=6e^-12^ | p=8e^-15^ | p=1e^-13^ | p=1e^-15^ | p=6e^-9^ |
|  | **Control (ALX1^165L/165L^)** | | | | | | |
| **Undifferentiated** | NS | p=7e^-6^ | p=1e^-15^ | p=6e^-13^ | p=4e^-8^ | p=8e^-14^ | p=1e^-15^ |

| ***HAND2*** | | | | | | | |
| --- | --- | --- | --- | --- | --- | --- | --- |
| **Day** | **2** | **4** | **6** | **8** | **10** | **12** | **14** |
|  | ***ALX1*^165F/165F^** | | | | | | |
| **Undifferentiated** | p=0.0004 | p=6e^-7^ | p=3e^-12^ | p=1e^-15^ | p=5e^-14^ | p=1e^-12^ | p=6e^-11^ |
|  | **ALX1^165L/165F^** | | | | | | |
| **Undifferentiated** | NS | NS | NS | p=0.0022 | p=0.0003 | p=0.000008 | p=0.00003 |
|  | **Control (ALX1^165L/165L^)** | | | | | | |
| **Undifferentiated** | NS | NS | p=0.0019 | p=0.0001 | p=0.0127 | p=0.0027 | p=0.0019 |

**: Significant differences when comparing undifferentiated iPSCs, *ALX1^165L/165L^* and *ALX1^165L/165F^* iPSC.

| ***ALX1*** | | | | | | | |
| --- | --- | --- | --- | --- | --- | --- | --- |
| **Day** | **2** | **4** | **6** | **8** | **10** | **12** | **14** |
|  | ***ALX1*^165F/165F^** | | | | | | |
| **ALX1^165L/165L^** | NS | p=0.0002 | NS | p=2e^-8^ | NS | NS | NS |
| **ALX1^165L/165F^** | NS | NS | NS | p=0.0021 | NS | NS | NS |

| ***ZIC1*** | | | | | | | |
| --- | --- | --- | --- | --- | --- | --- | --- |
| **Day** | **2** | **4** | **6** | **8** | **10** | **12** | **14** |
|  | ***ALX1*^165F/165F^** | | | | | | |
| **ALX1^165L/165L^** | p=0.003 | p=0.0017 | p=0.0014 | p=1e^-6^ | NS | NS | NS |
| **ALX1^165L/165F^** | p=1e^-1^ | p=5e^-15^ | p=3e^-15^ | p=1e^-15^ | NS | NS | NS |

| ***PAX7*** | | | | | | | |
| --- | --- | --- | --- | --- | --- | --- | --- |
| **Day** | **2** | **4** | **6** | **8** | **10** | **12** | **14** |
|  | ***ALX1*^165F/165F^** | | | | | | |
| **ALX1^165L/165L^** | p=2e^-10^ | p=1e^-15^ | p=6e^-7^ | p=0.0147 | NS | NS | NS |
| **ALX1^165L/165F^** | p=4e^-13^ | p=2e^-9^ | p=3e^-11^ | p=2e^-6^ | NS | NS | NS |

| ***PAX3*** | | | | | | | |
| --- | --- | --- | --- | --- | --- | --- | --- |
| **Day** | **2** | **4** | **6** | **8** | **10** | **12** | **14** |
|  | ***ALX1*^165F/165F^** | | | | | | |
| **ALX1^165L/165L^** | p=0.0000006 | p=0.000009 | p=0.0012 | p=0.0152 | p=0.0003 | p=0.0017 | p=0.00132 |
| **ALX1^165L/165F^** | p=0.0003 | p=7e^-8^ | p=2e^-10^ | p=9e^-6^ | p=0.0045 | p=0.0058 | p=0.0001 |

| ***MSX1*** | | | | | | | |
| --- | --- | --- | --- | --- | --- | --- | --- |
| **Day** | **2** | **4** | **6** | **8** | **10** | **12** | **14** |
|  | ***ALX1*^165F/165F^** | | | | | | |
| **ALX1^165L/165L^** | NS | p=0.0128 | p=0.0235 | p=0.0001 | p=0.0016 | p=0.00002 | p=0.0007 |
| **ALX1^165L/165F^** | NS | p=0.000001 | p=4e^-8^ | p=3e^-8^ | p=0.00001 | p=0.00006 | p=0.0003 |

| ***MSX2*** | | | | | | | |
| --- | --- | --- | --- | --- | --- | --- | --- |
| **Day** | **2** | **4** | **6** | **8** | **10** | **12** | **14** |
|  | ***ALX1*^165F/165F^** | | | | | | |
| **ALX1^165L/165L^** | p=7e^-13^ | p=1e^-15^ | p=0.0134 | p=5e^-15^ | p=3e^-15^ | p=6e^-15^ | p=1e^-15^ |
| **ALX1^165L/165F^** | p=0.003 | p=0.0055 | p=0.008 | p= p=6e^-6^0 | p=0.0495 | p=0.0402 | p=0.0018 |

| ***DLX5*** | | | | | | | |
| --- | --- | --- | --- | --- | --- | --- | --- |
| **Day** | **2** | **4** | **6** | **8** | **10** | **12** | **14** |
|  | ***ALX1*^165F/165F^** | | | | | | |
| **ALX1^165L/165L^** | p=0.000006 | p=2e^-10^ | p=0.0134 | p=3e^-12^ | p=2e^-14^ | p=1e^-15^ | p=1e^-15^ |
| **ALX1^165L/165F^** | p=0.00004 | p=0.000003 | p=0.0018 | p=0.0027 | p=0.00001 | p=0.0037 | p=0.000002 |

| ***FOXD3*** | | | | | | | |
| --- | --- | --- | --- | --- | --- | --- | --- |
| **Day** | **2** | **4** | **6** | **8** | **10** | **12** | **14** |
|  | ***ALX1*^165F/165F^** | | | | | | |
| **ALX1^165L/165L^** | NS | NS | NS | NS | NS | NS | NS |
| **ALX1^165L/165F^** | NS | NS | NS | NS | NS | NS | NS |

| ***P75*** | | | | | | | |
| --- | --- | --- | --- | --- | --- | --- | --- |
| **Day** | **2** | **4** | **6** | **8** | **10** | **12** | **14** |
|  | ***ALX1*^165F/165F^** | | | | | | |
| **ALX1^165L/165L^** | NS | NS | NS | NS | NS | NS | NS |
| **ALX1^165L/165F^** | NS | NS | NS | NS | NS | NS | NS |

| ***TFAP2A*** | | | | | | | |
| --- | --- | --- | --- | --- | --- | --- | --- |
| **Day** | **2** | **4** | **6** | **8** | **10** | **12** | **14** |
|  | ***ALX1*^165F/165F^** | | | | | | |
| **ALX1^165L/165L^** | p=0.00004 | p=1e^-15^ | p=1e^-15^ | p=9e^-15^ | p=2e^-9^ | p=8e^-9^ | p=1e^-15^ |
| **ALX1^165L/165F^** | p=0.00002 | p=0.00003 | p=0.00007 | p=0.001 | p=0.0335 | p=0.0464 | p=0.0008 |

| ***SLUG*** | | | | | | | |
| --- | --- | --- | --- | --- | --- | --- | --- |
| **Day** | **2** | **4** | **6** | **8** | **10** | **12** | **14** |
|  | ***ALX1*^165F/165F^** | | | | | | |
| **ALX1^165L/165L^** | NS | p=2e^-13^ | p=0.0217 | p=0.00007 | p=0.00001 | p=0.0004 | p=0.022 |
| **ALX1^165L/165F^** | NS | p=1e^-15^ | p=5e^-7^ | p=2e^-10^ | p=7e^-10^ | p=0.00001 | p=0.000001 |

| ***HAND2*** | | | | | | | |
| --- | --- | --- | --- | --- | --- | --- | --- |
| **Day** | **2** | **4** | **6** | **8** | **10** | **12** | **14** |
|  | ***ALX1*^165F/165F^** | | | | | | |
| **ALX1^165L/165L^** | NS | NS | NS | NS | NS | NS | NS |
| **ALX1^165L/165F^** | NS | NS | NS | NS | NS | NS | NS |

***: Siginificant differences when comparing undifferentiated iPSC (p<0.05), *ALX1^165L/165L^* and *ALX1^165F/165F^* iPSC (p<0.05).

| ***MSX2*** | | | | | | | |
| --- | --- | --- | --- | --- | --- | --- | --- |
| **Day** | **2** | **4** | **6** | **8** | **10** | **12** | **14** |
|  | ***ALX1*^165F/165F^** | | | | | | |
| **ALX1^165L/165L^** | p=0.00002 | p=4e^-6^ | p=0.0144 | p=5e^-7^ | p=1e^-7^ | p=9e^-8^ | p=0.00003 |

| ***DLX5*** | | | | | | | |
| --- | --- | --- | --- | --- | --- | --- | --- |
| **Day** | **2** | **4** | **6** | **8** | **10** | **12** | **14** |
|  | ***ALX1*^165F/165F^** | | | | | | |
| **ALX1^165L/165L^** | p=0.0021 | p=NS | p=0.00009 | p=0.003 | p=2e^-13^ | p=1e^-15^ | 0,000002 |

| ***TFAP2A*** | | | | | | | |
| --- | --- | --- | --- | --- | --- | --- | --- |
| **Day** | **2** | **4** | **6** | **8** | **10** | **12** | **14** |
|  | ***ALX1*^165F/165F^** | | | | | | |
| **ALX1^165L/165L^** | p=0.0035 | p=0.00006 | p=0.0005 | p=0.0002 | p=0.0018 | p=0.0027 | 0.0008 |
