# Supplementary material for: ALX1‐related frontonasal dysplasia results from defective neural crest cell development and migration
Source: EMBO Mol Med. 2020 Sep 11;12(10):e12013. doi: 10.15252/emmm.202012013 (PMC7539331; doi:10.15252/emmm.202012013)
Supplement: Supplementary file 3 — Table EV2 [file EMMM-12-e12013-s003.docx]

**Table EV2:** P-values for Figure 6.

| ***alx1*** | | | | | |
| --- | --- | --- | --- | --- | --- |
| **Time** | **4 hpf** | **10 ss** | **24 hpf** | **36 hpf** | **48 hpf** |
|  | alx1-/- | alx1-/- | alx1-/- | alx1-/- | alx1-/- |
| 1 cell | NS | NS | p=0.0000004 | p=0.002 | p=0.000003 |
|  | Wild Type | Wild Type | Wild Type | Wild Type | Wild Type |
| 1 cell | p=0.00000002 | p=0.0000002 | p=0.000001 | p=0.0000009 | p=0.00000003 |

| ***alx3*** | | | | | |
| --- | --- | --- | --- | --- | --- |
| **Time** | **4 hpf** | **10 ss** | **24 hpf** | **36 hpf** | **48 hpf** |
|  | alx1-/- | alx1-/- | alx1-/- | alx1-/- | alx1-/- |
| 1 cell | p=0.01 | p=0.006 | p=0.000001 | p=0,00003 | p=0.0000003 |
|  | Wild Type | Wild Type | Wild Type | Wild Type | Wild Type |
| 1 cell | p=0.003 | p=0.0007 | p=0.000006 | p=0.000004 | p=0.0000001 |

| ***alx4a*** | | | | | |
| --- | --- | --- | --- | --- | --- |
| **Time** | **4 hpf** | **10 ss** | **24 hpf** | **36 hpf** | **48 hpf** |
|  | alx1-/- | alx1-/- | alx1-/- | alx1-/- | alx1-/- |
| 1 cell | p=0.004 | p=0.001 | p=0.00007 | p=0.0002 | p=0.00005 |
|  | Wild Type | Wild Type | Wild Type | Wild Type | Wild Type |
| 1 cell | p=0.005 | p=0.0006 | p=0.00004 | p=0.0000002 | p=0.000003 |

| ***alx4b*** | | | | | |
| --- | --- | --- | --- | --- | --- |
| **Time** | **4 hpf** | **10 ss** | **24 hpf** | **36 hpf** | **48 hpf** |
|  | alx1-/- | alx1-/- | alx1-/- | alx1-/- | alx1-/- |
| 1 cell | NS | NS | p=0.003 | p=0.0006 | p=0.00000002 |
|  | Wild Type | Wild Type | Wild Type | Wild Type | Wild Type |
| 1 cell | NS | NS | p=0.012 | p=0.000006 | p=0.000005 |

*: Significantly different from WT zebrafish

| ***alx1*** | | | | | |
| --- | --- | --- | --- | --- | --- |
| **Time** | **4 hpf** | **10 ss** | **24 hpf** | **36 hpf** | **48 hpf** |
|  | alx1-/- | alx1-/- | alx1-/- | alx1-/- | alx1-/- |
| Wild type | p=0.0043 | p=0.117 | p=0.0000028 | p=3e^-8^ | p=0.0179 |

| ***alx3*** | | | | | |
| --- | --- | --- | --- | --- | --- |
| **Time** | **4 hpf** | **10 ss** | **24 hpf** | **36 hpf** | **48 hpf** |
|  | alx1-/- | alx1-/- | alx1-/- | alx1-/- | alx1-/- |
| Wild type | NS | p=0.0055 | p=3e^-10^ | p=4e^-9^ | p=0.0017 |

| ***alx4a*** | | | | | |
| --- | --- | --- | --- | --- | --- |
| **Time** | **4 hpf** | **10 ss** | **24 hpf** | **36 hpf** | **48h pf** |
|  | alx1-/- | alx1-/- | alx1-/- | alx1-/- | alx1-/- |
| Wild type | NS | NS | p=2e^-9^ | p=2e^-8^ | NS |

| ***alx4b*** | | | | | |
| --- | --- | --- | --- | --- | --- |
| **Time** | **4 hpf** | **10 ss** | **24 hpf** | **36 hpf** | **48 hpf** |
|  | alx1-/- | alx1-/- | alx1-/- | alx1-/- | alx1-/- |
| Wild type | NS | NS | NS | NS | NS |
