# Supplementary material for: ALX1‐related frontonasal dysplasia results from defective neural crest cell development and migration
Source: EMBO Mol Med. 2020 Sep 11;12(10):e12013. doi: 10.15252/emmm.202012013 (PMC7539331; doi:10.15252/emmm.202012013)
Supplement: Supplementary file 4 — Table EV3 [file EMMM-12-e12013-s004.docx]

**Table EV3:** Primers used for qPCR (human genes)

| **Gene Name** | **Forward Primer** | **Reverse Primer** |
| --- | --- | --- |
| *GAPDH* | GACAGTCAGCCGCATCTTCT | TTAAAAGCAGCCCTGGTGAC |
| *RPLP0* | CAGATCCGCATGTCCCTTCG | AACACAAAGCCCACATTCCC |
| *OCT4* | TGGAGTTTGTGCCAGGGTTT | CTGTGTCCCAGGCTTCTTT |
| *NANOG* | GTCCCAAAGGCAAACAACCC | GCTGGGTGGAAGAGAACACA |
| *AFP* | CATATGCCAACAGGAGGCCA | CTGAGCTTGGCACAGATCCT |
| *GATA4* | GAAGGAGCCAGCCTAGCAG | CTATTGGGGGCAGAAGACGG |
| *FOXA2* | GCACTCGGCTTCCAGTATGC | TGTTCATGCCGTTCATCCCC |
| *BRACHYURY* | GCTCTGCCCCCTAGAATGTG | ACAAAAGGAGGGGCTTCAC |
| *RUNX1* | GGAAGTCAACCTCTGCTGCT | TCGGACCACAGAGCACTTTC |
| *CD34* | TCTAGGCTCCAGCCAGAAAA | AAAACGTGTTGCCTTGAACC |
| *NESTIN* | GTAGCTCCCAGAGAGGGGAA | CTGTCCTGGGACTCCGTTTC |
| *GFAP* | CAGAGCCTCAAGGACGAGAT | GCTTTTGCCCCCTCGAATC |
| *SOX1* | CAACCAGGACCGGGTCAAAC | CCTCGGACATGACCTTCCAC |
| *ALX1* | CTGAGCGAGAAGTTTGCCCT | TGTCCAGCGTCTCCATAACG |
| *PAX3* | TCCATACGTCCTGGTGCCAT | TTCTCCACGTCAGGCGTTG |
| *ZIC1* | CGAGCGACAAGCCCTATCTT | TCTGTGGAGGGAGACACGAT |
| *TWIST1* | GCATTCTCAAGAGGTCGTGC | TTTGCAGGCCAGTTTGATCC |
| *TFAP2A* | GAGAACAGAACAGGCCGTGA | TGACTCAGTCCCATGAAGCG |
| *MSX2* | CTGGTGAAGCCCTTCGAGAC | AGGGCTCATATGTCTTGGCG |
| *DLX5* | AGAGACTTCACGACTCCCA | ACACTCCTGTCATCGCTCA |
| *P75* | CCTCATCCCTGTCTATTGCTCC | GTTGGCTCCTTGCTTGTTCTGC |
| *FOXD3* | GGCAAGGGCAACTACTGGA | GGCTGTAAGCGCCGAAG |
| *PAX7* | GGATGTGGAGTCGGAACCTG | CTGGGTAGTGGGTCCTCTCA |
| *MSX1* | TCCGCAAACACAAGACGAAC | TACTGCTTCTGGCGGAACTT |
| *SNAI2* | TGTCATACCACAACCAGAGA | CTTGGAGGAGGTGTCAGAT |
| *HAND2* | GACACTCCCGTGTGGTAAGG | AAGGGGTTGAGTAGGTTGGC |
