# Supplementary material for: ALX1‐related frontonasal dysplasia results from defective neural crest cell development and migration
Source: EMBO Mol Med. 2020 Sep 11;12(10):e12013. doi: 10.15252/emmm.202012013 (PMC7539331; doi:10.15252/emmm.202012013)
Supplement: Supplementary file 5 — Table EV4 [file EMMM-12-e12013-s005.docx]

**Table EV4:** Primers used for qPCR (zebrafish genes)

| **Gene Name** | **Forward Primer** | **Reverse Primer** |
| --- | --- | --- |
| *elfa* | CTTCTCAGGCTGACTGTGC | CCGCTAGCATTACCCTCC |
| *18S* | TCGCTAGTTGGCATCGTTTATG | CGGAGGTTCGAAGACGATCA |
| *alx1* | CGTGACTTACTGCGCTCCTA | CGAGTTCGTCGAGGTCTGTT |
| *alx3* | TTATGGGACGCTACGCTGAC | AGTGTGTTTTCCAGGCGAGG |
| *alx4a* | CGAGTTGCCCCAAAACAGTG | ACGGTTGCGTCTTTTCTTGC |
| *alx4b* | ACTGGATGATAGCACAGCCG | ACCGTAGCATGAGATCAGCG |
| *pax3a* | ATACGACCGTGCGTCATCTC | ACGTCAGGAGTTGTGCTCTG |
| *pax3b* | CACAGAGGCACGAGTACAGG | TACGGGGACAGACTCGACAT |
