# Supplementary figures and images for: ALX1‐related frontonasal dysplasia results from defective neural crest cell development and migration
Source: EMBO Mol Med. 2020 Sep 11;12(10):e12013. doi: 10.15252/emmm.202012013 (PMC7539331; doi:10.15252/emmm.202012013)

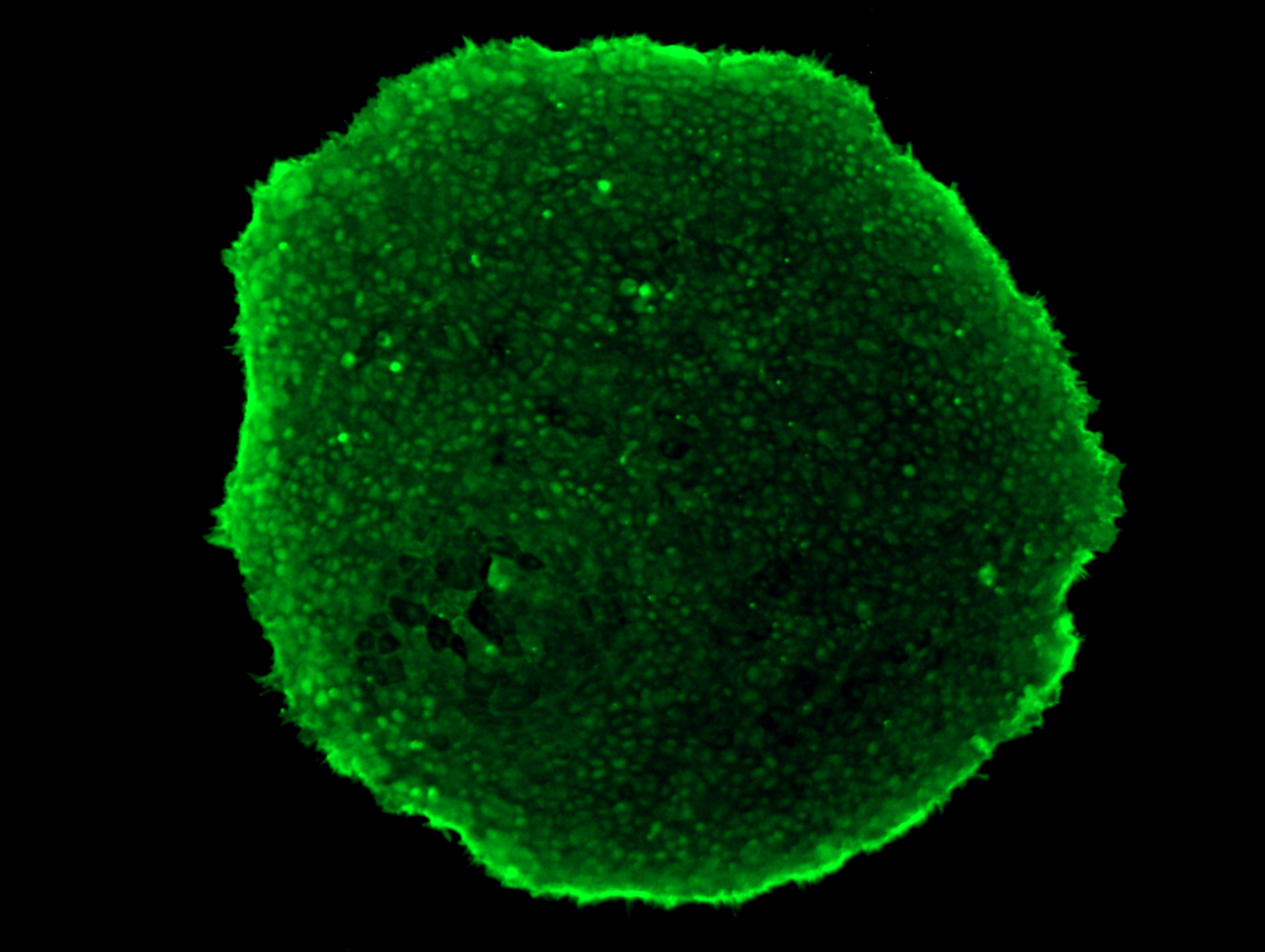

Supplement: Supplementary file 12 — Source Data for Figure 1E [file EMMM-12-e12013-s010.zip › Source_data_Fig_1E/ALX1_165F_165F_SSEA4.tif]

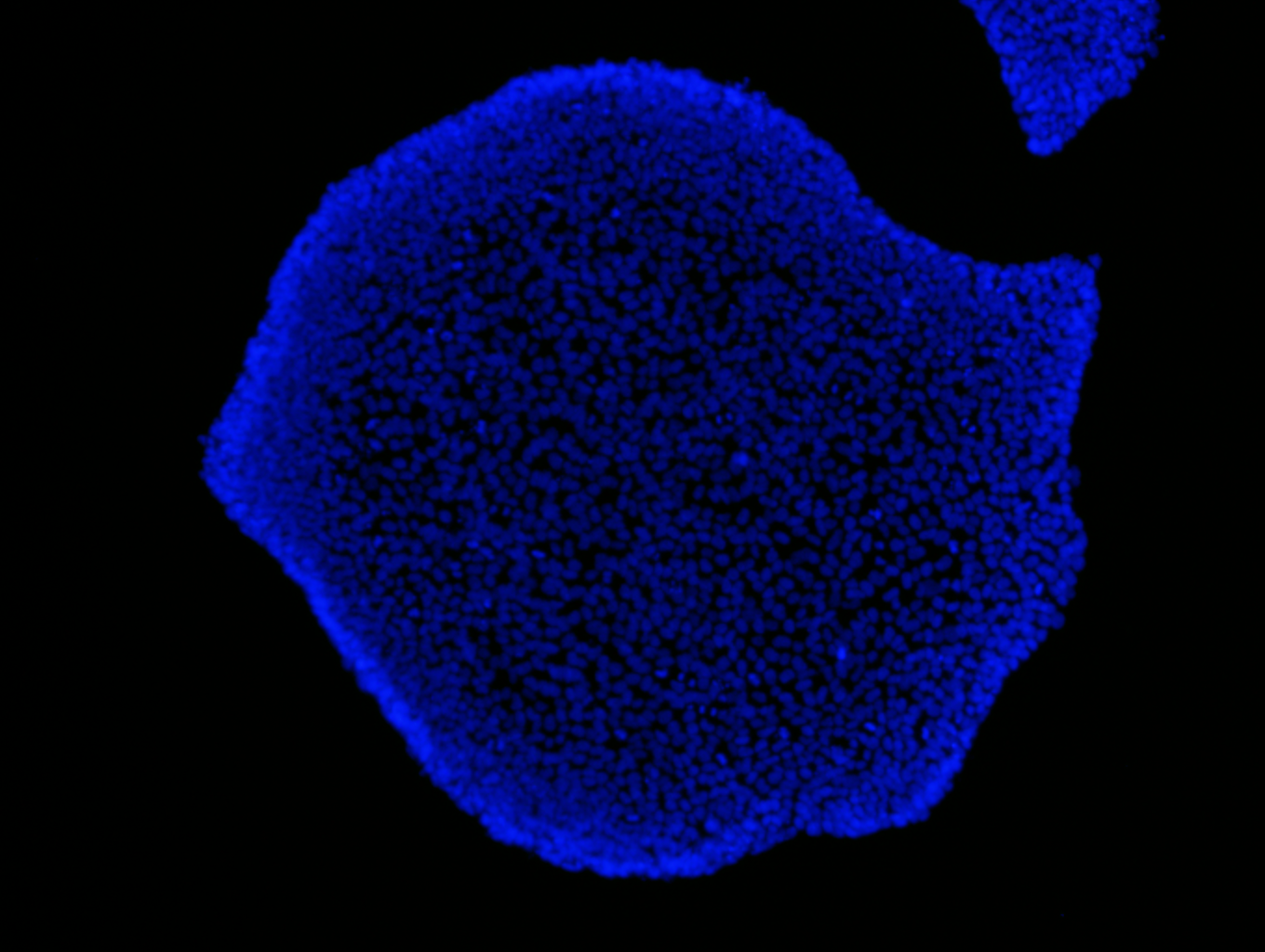

Supplement: Supplementary file 12 — Source Data for Figure 1E [file EMMM-12-e12013-s010.zip › Source_data_Fig_1E/Control_DAPI.tif]

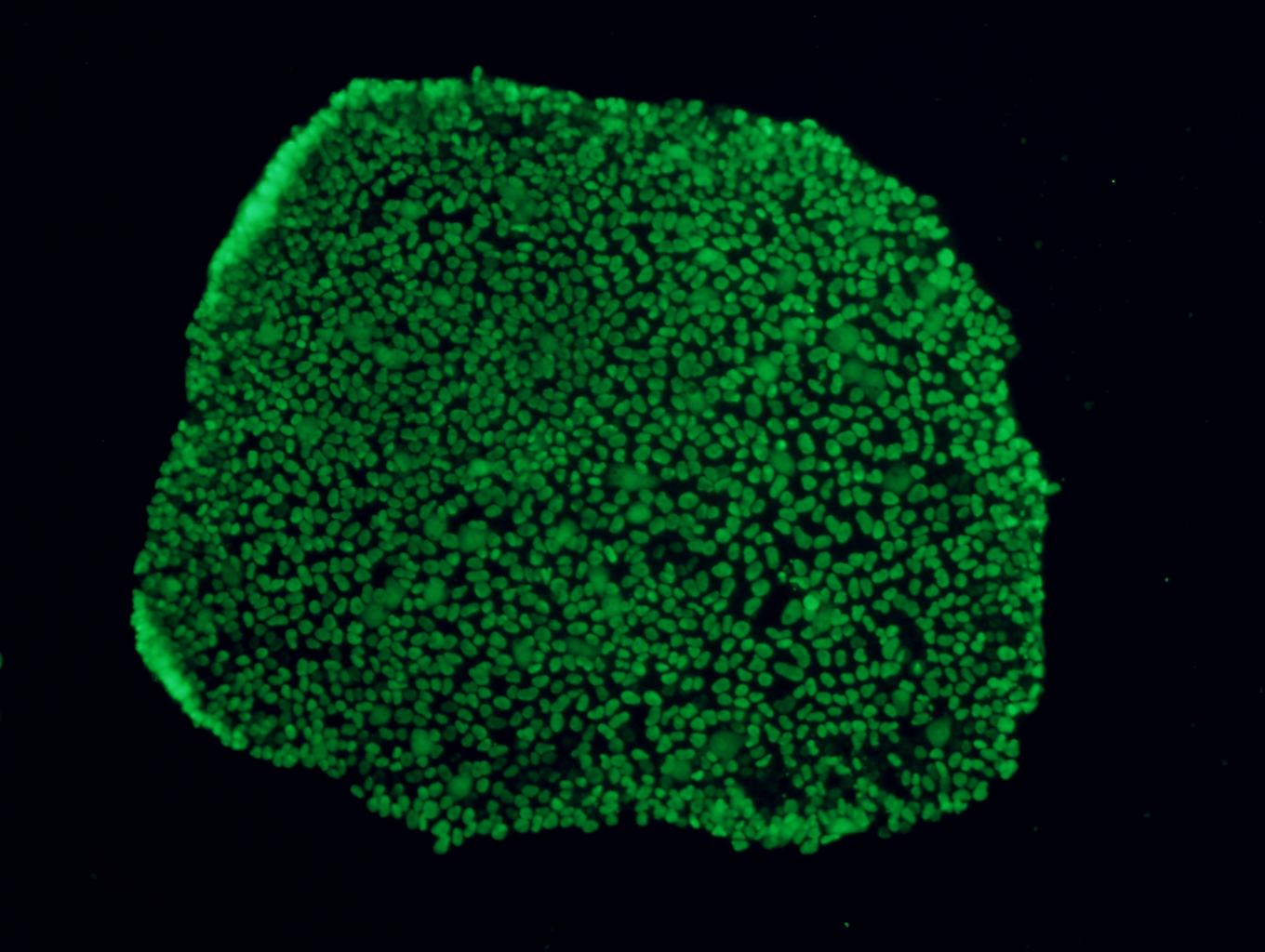

Supplement: Supplementary file 12 — Source Data for Figure 1E [file EMMM-12-e12013-s010.zip › Source_data_Fig_1E/ALX1_165L_165F_SOX2.tif]

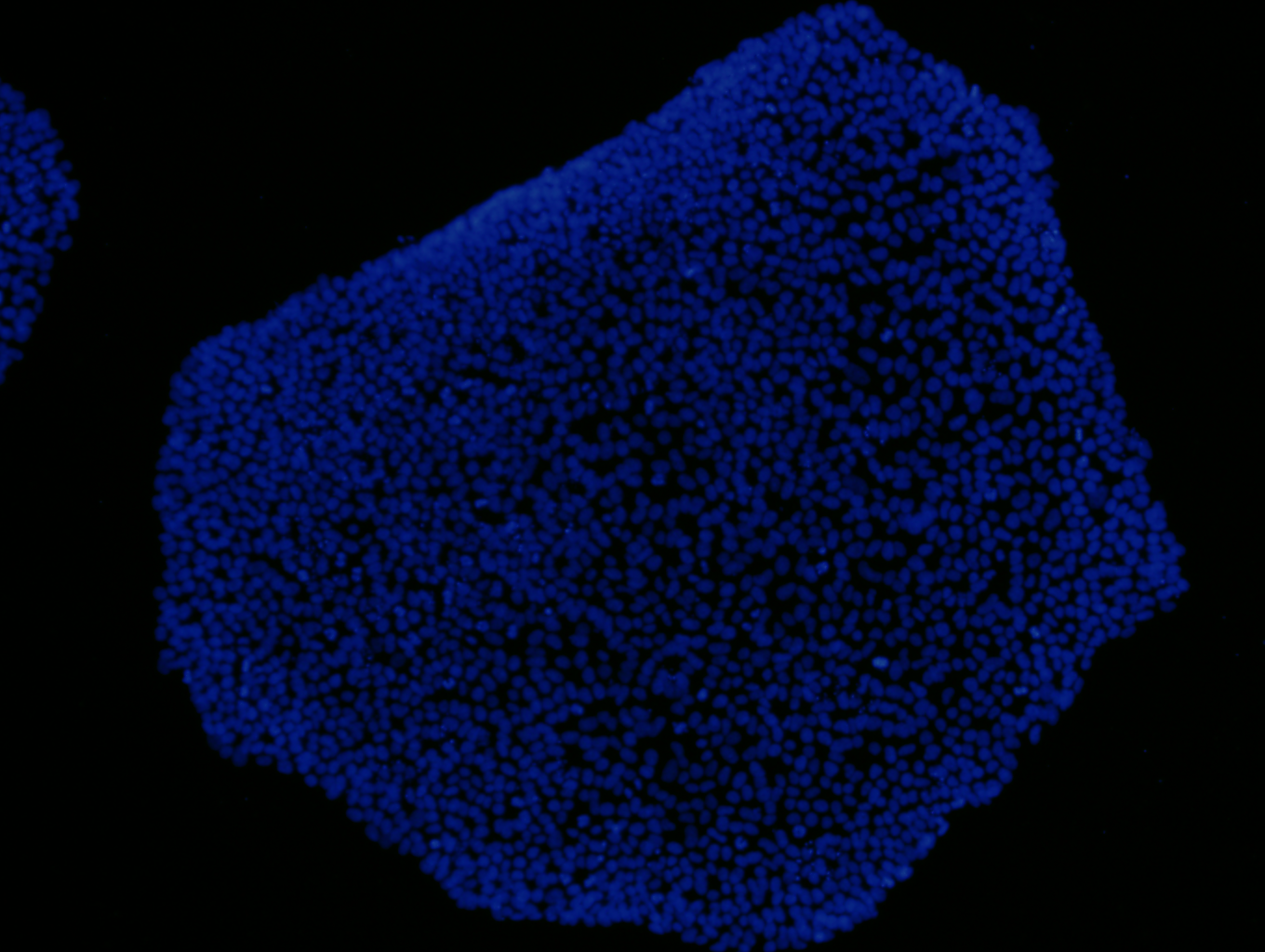

Supplement: Supplementary file 12 — Source Data for Figure 1E [file EMMM-12-e12013-s010.zip › Source_data_Fig_1E/ALX1_165L_165F_DAPI.tif]

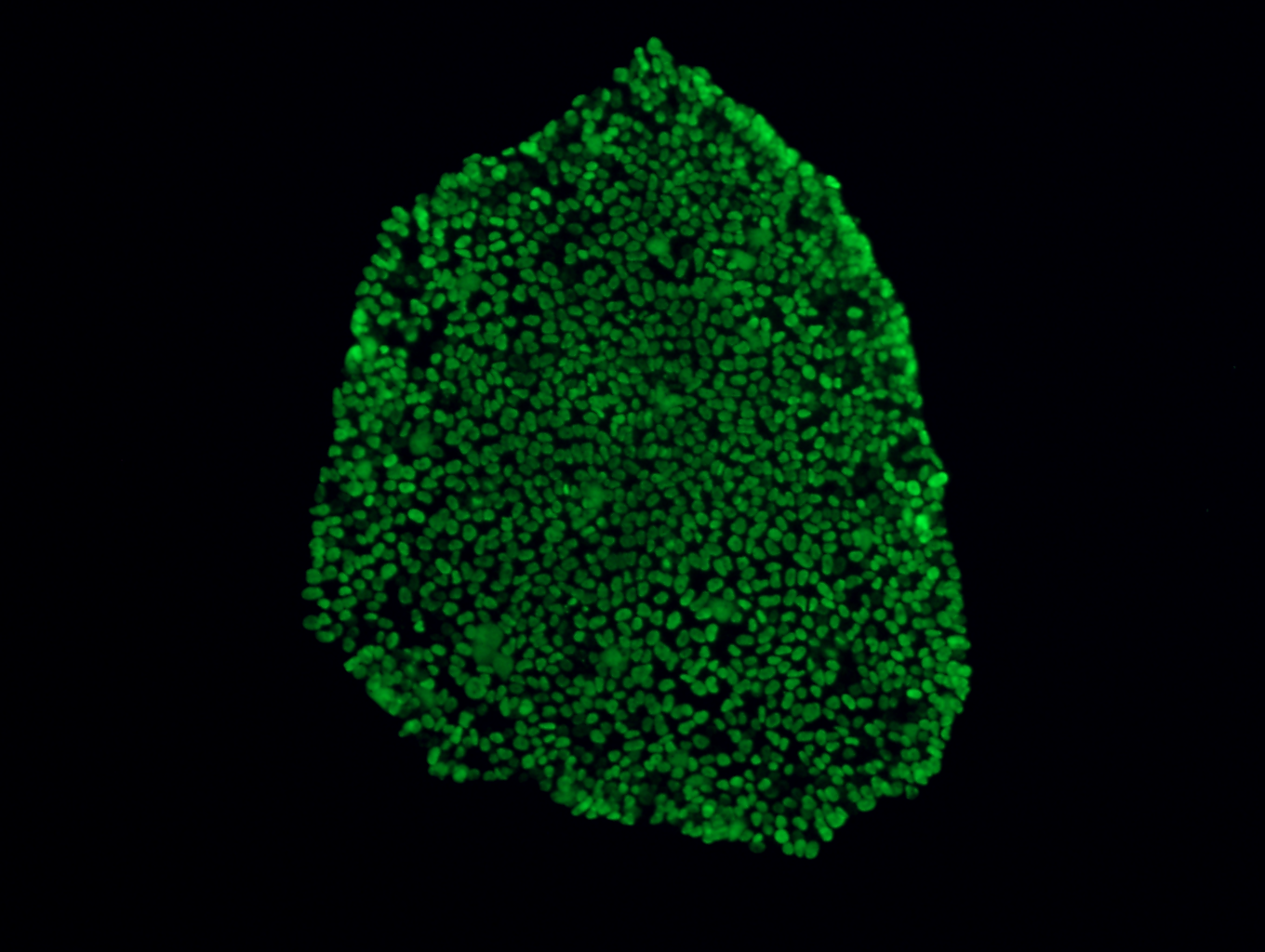

Supplement: Supplementary file 12 — Source Data for Figure 1E [file EMMM-12-e12013-s010.zip › Source_data_Fig_1E/Control_SOX2.tif]

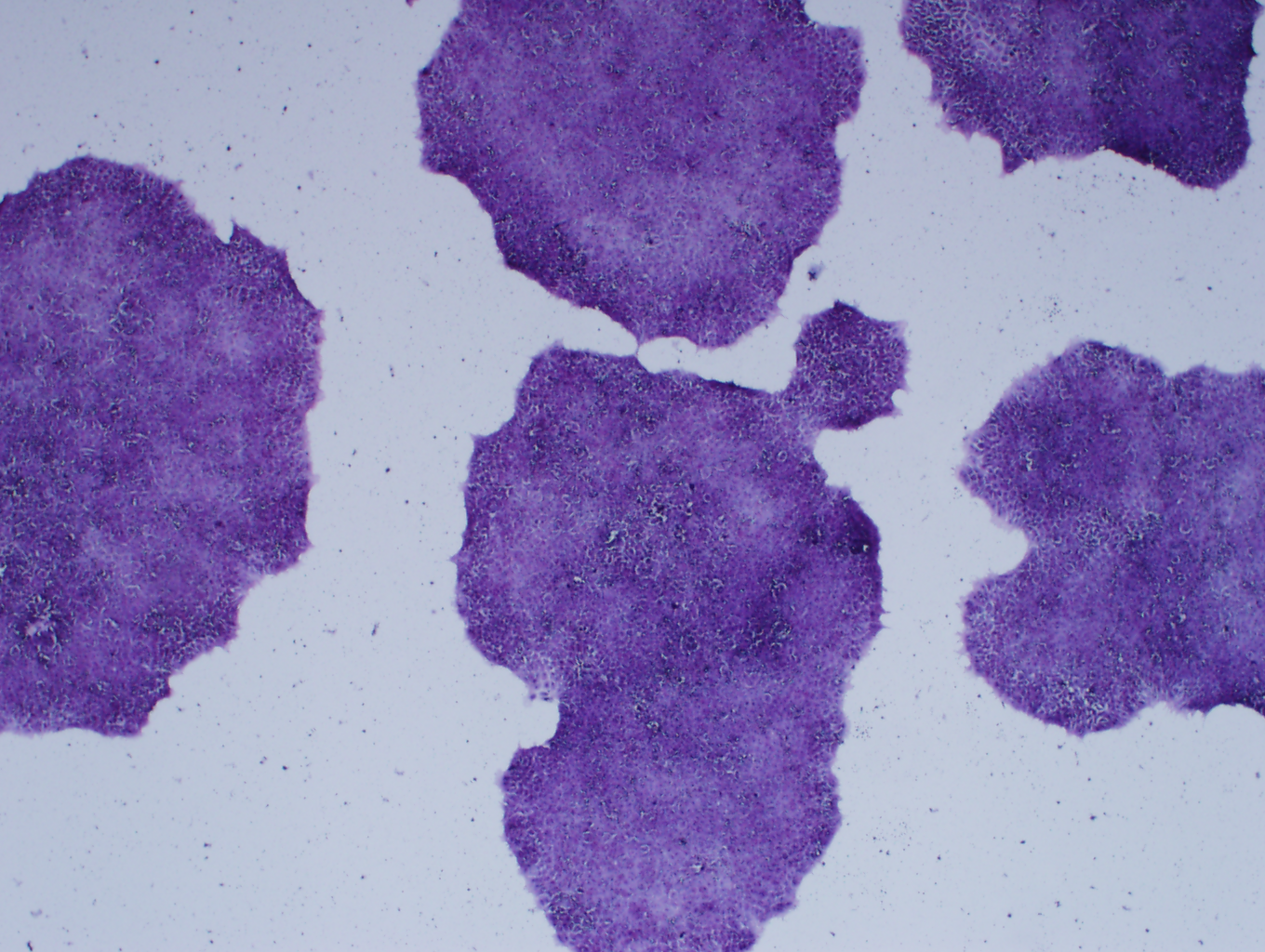

Supplement: Supplementary file 12 — Source Data for Figure 1E [file EMMM-12-e12013-s010.zip › Source_data_Fig_1E/FIG1E_ALX1_165F_165F_ALP.tif]

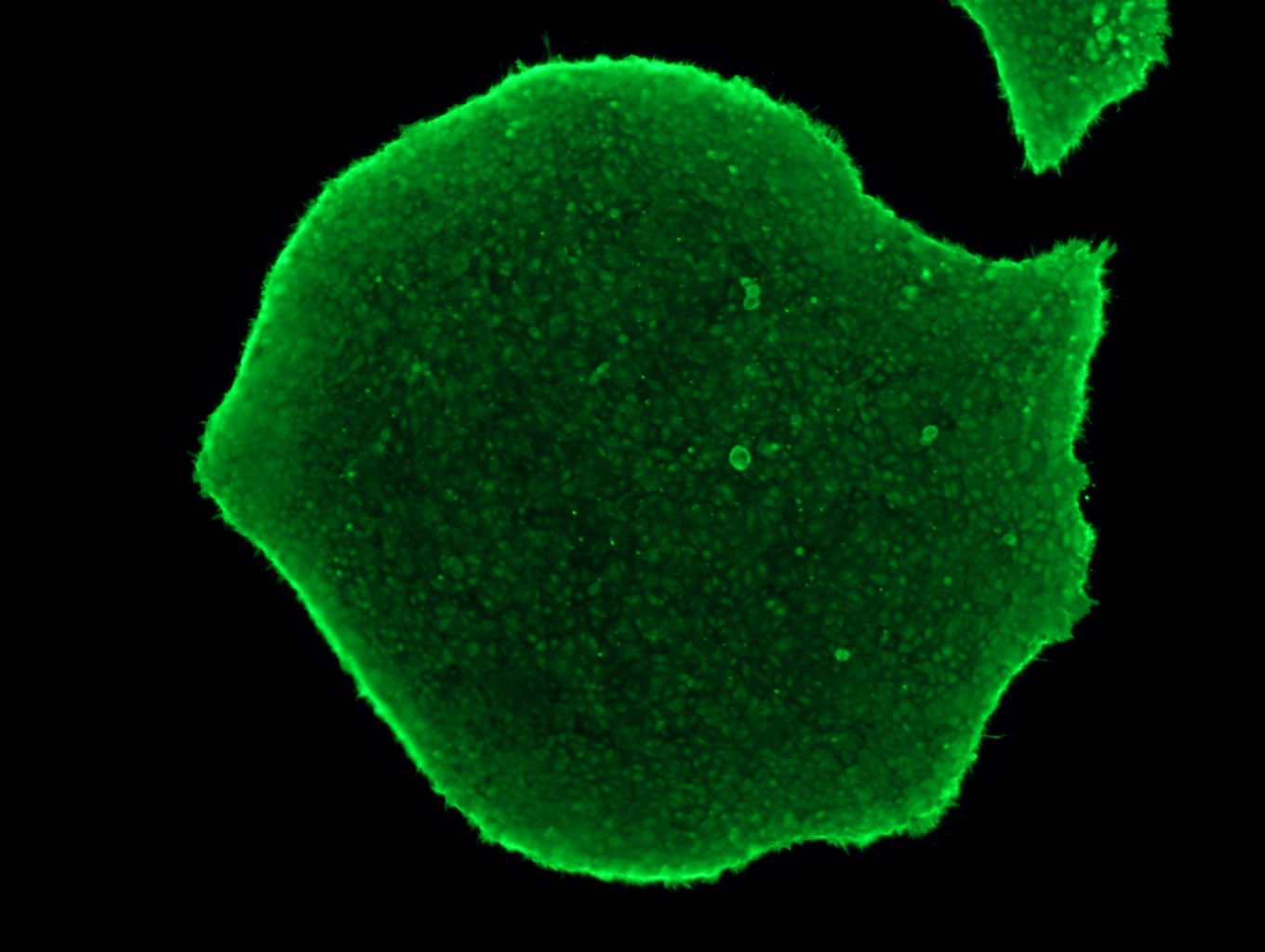

Supplement: Supplementary file 12 — Source Data for Figure 1E [file EMMM-12-e12013-s010.zip › Source_data_Fig_1E/Control_SSEA4.tif]

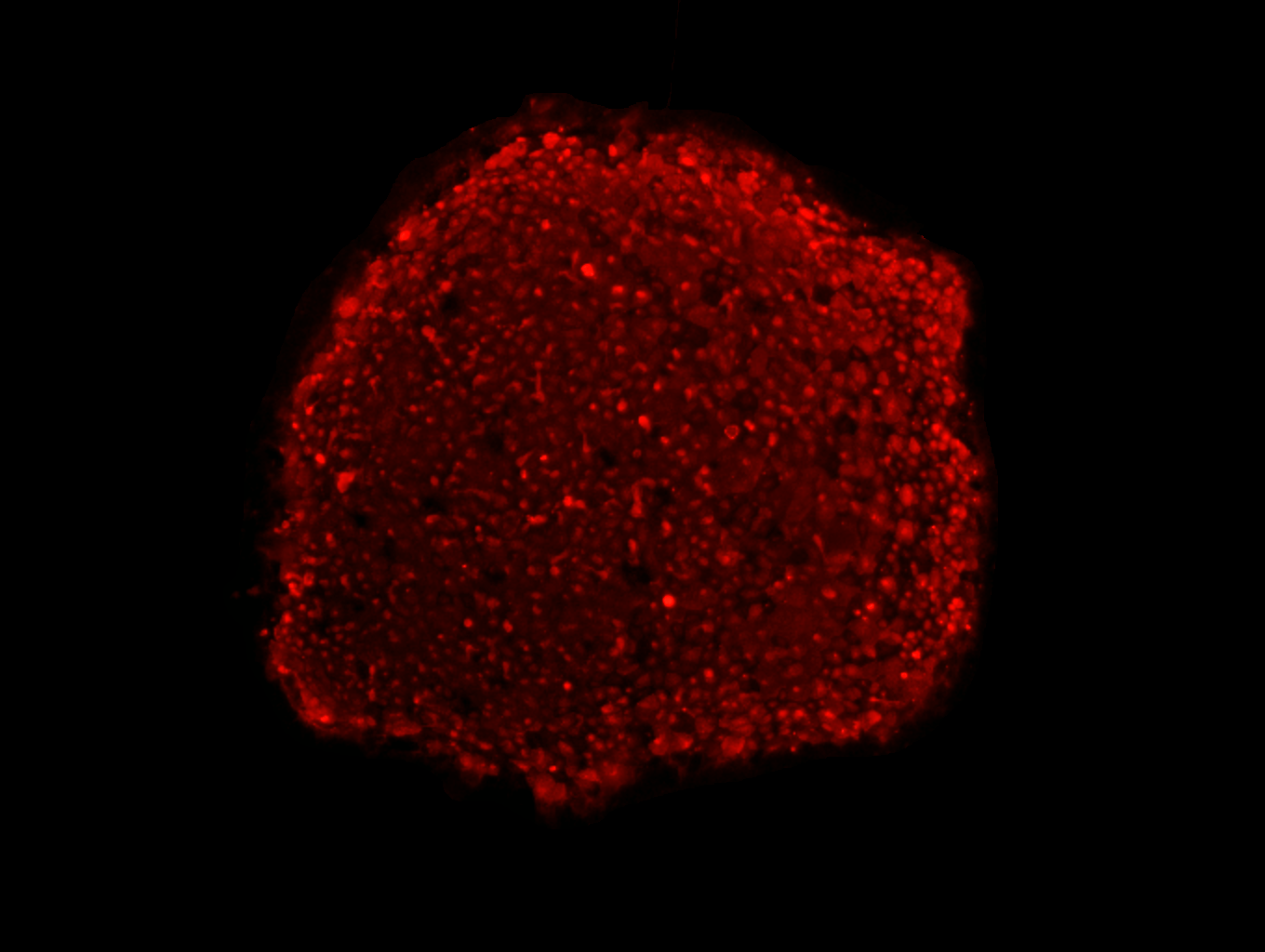

Supplement: Supplementary file 12 — Source Data for Figure 1E [file EMMM-12-e12013-s010.zip › Source_data_Fig_1E/ALX1_165F_165F_TRA-1-60.tif]

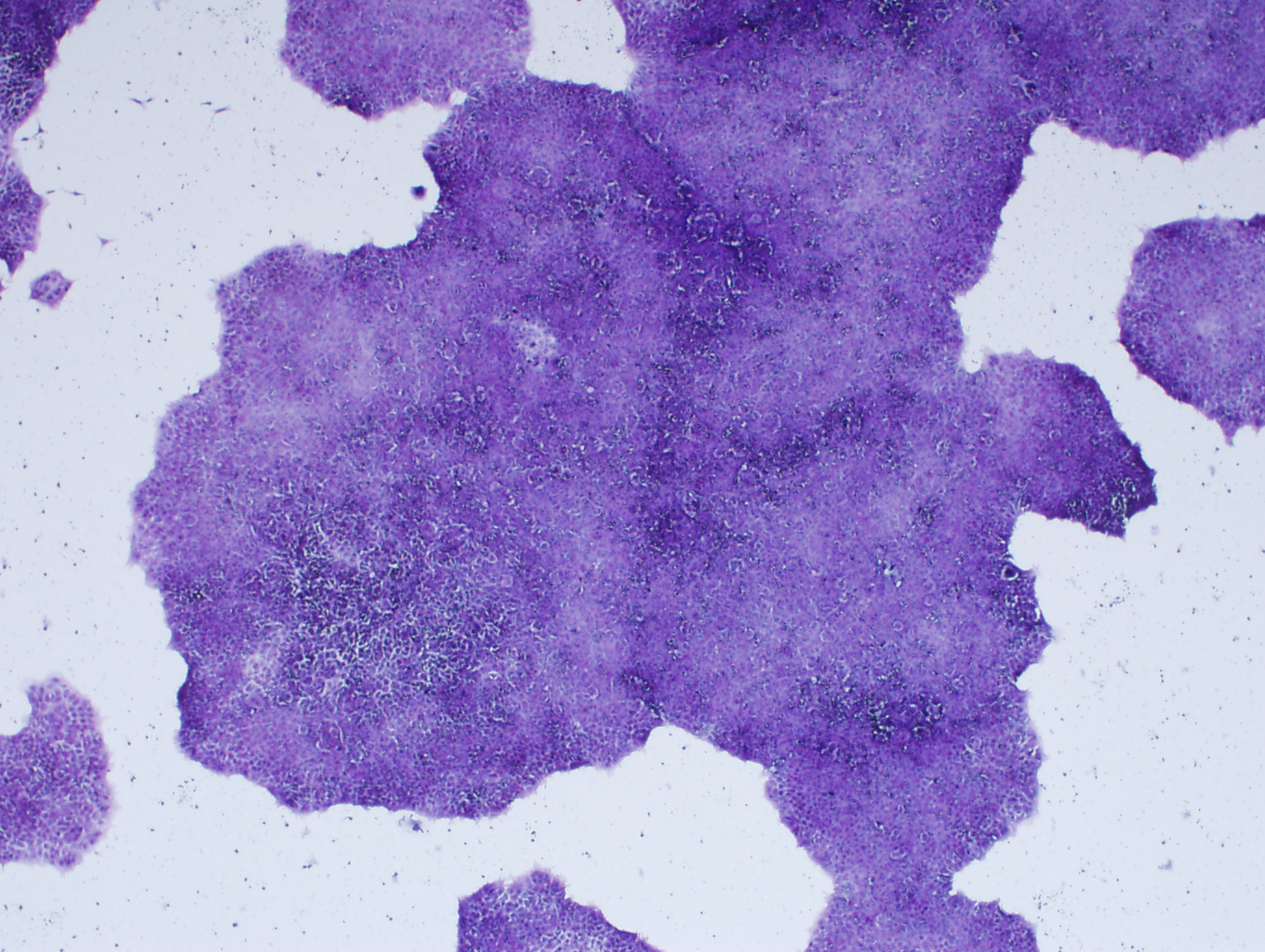

Supplement: Supplementary file 12 — Source Data for Figure 1E [file EMMM-12-e12013-s010.zip › Source_data_Fig_1E/ALX1_165L_165F_ALP.tif]

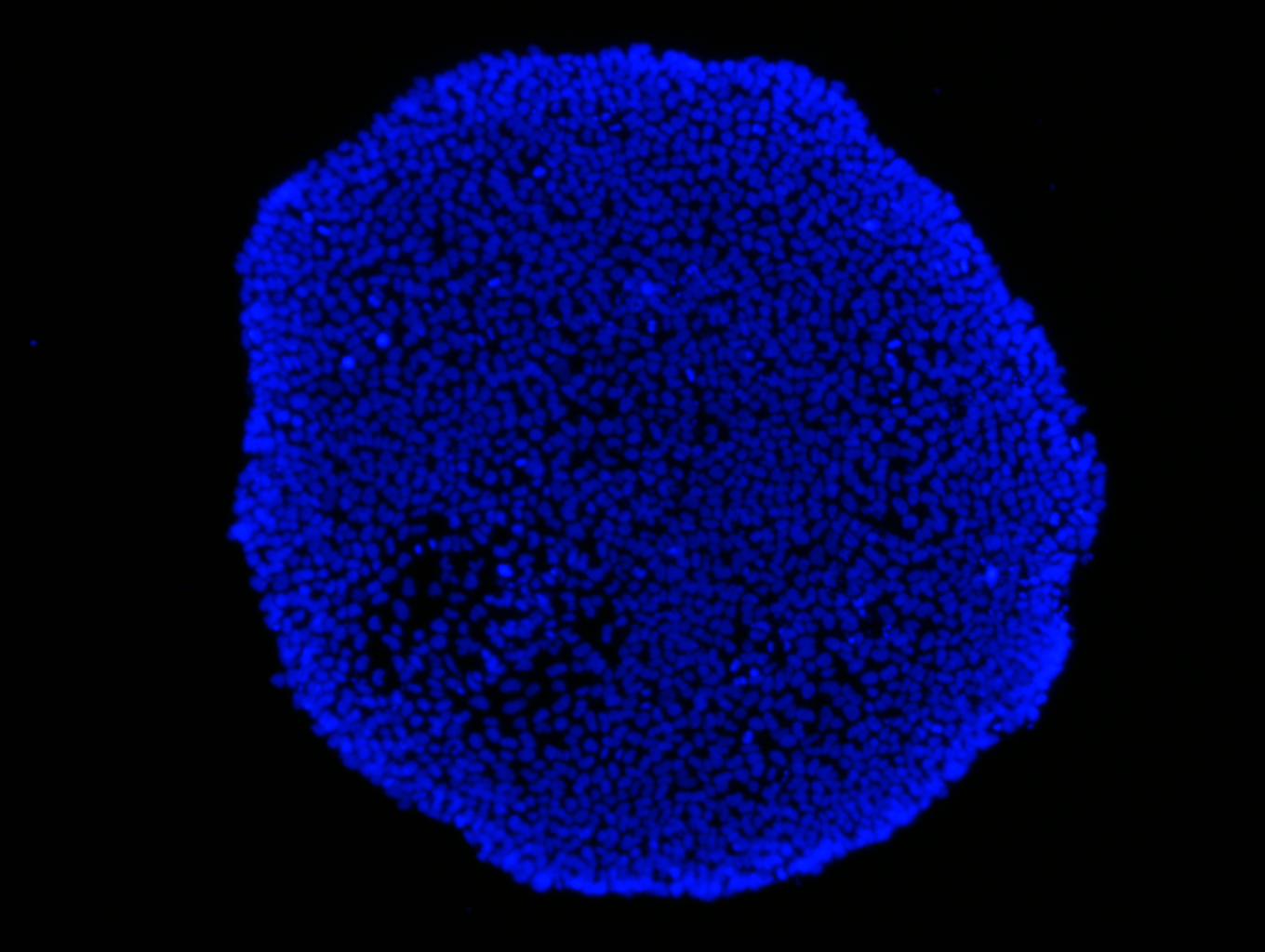

Supplement: Supplementary file 12 — Source Data for Figure 1E [file EMMM-12-e12013-s010.zip › Source_data_Fig_1E/ALX1_165F_165F_DAPI.tif]

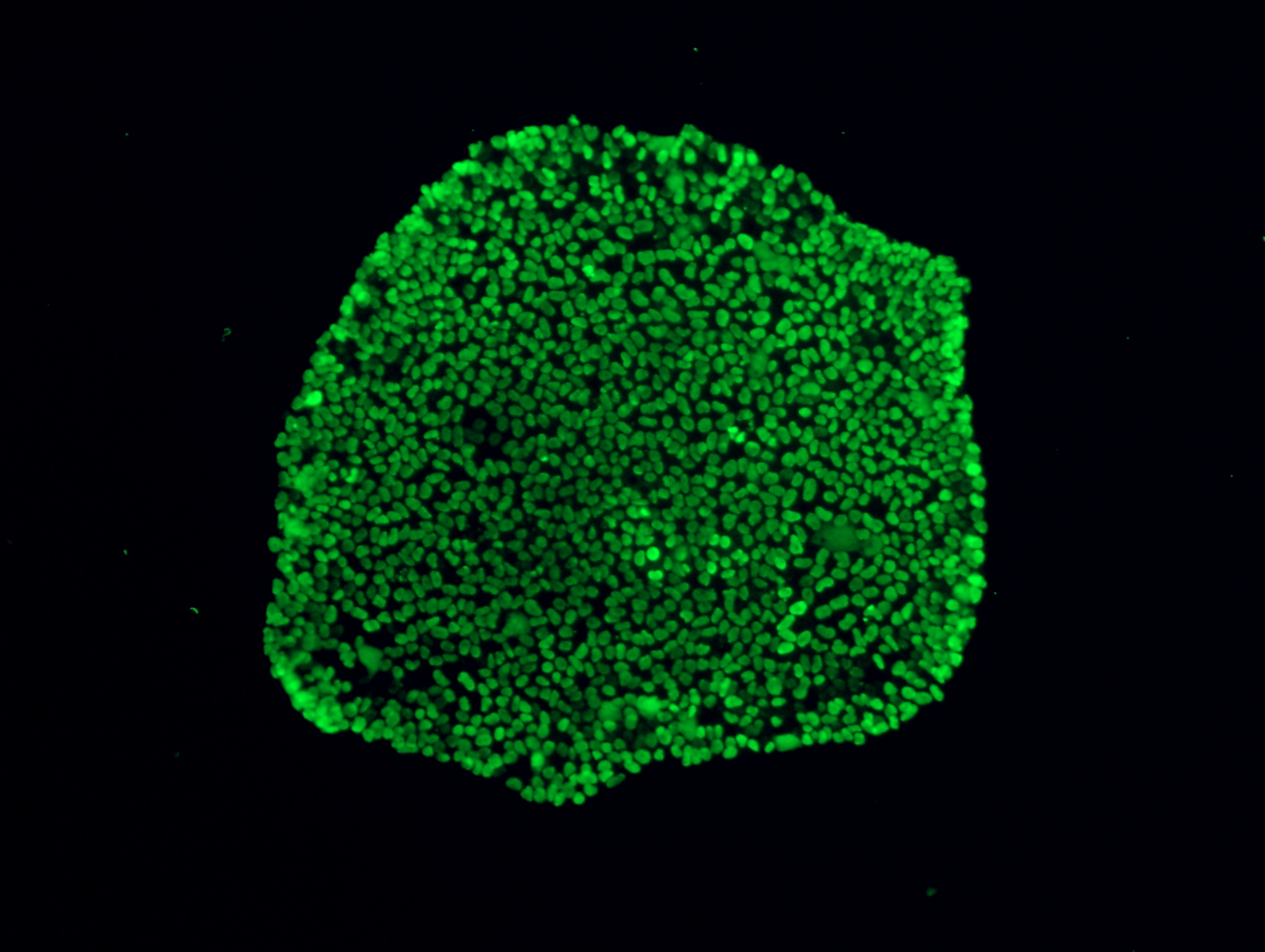

Supplement: Supplementary file 12 — Source Data for Figure 1E [file EMMM-12-e12013-s010.zip › Source_data_Fig_1E/ALX1_165F_165F_SOX2.tif]

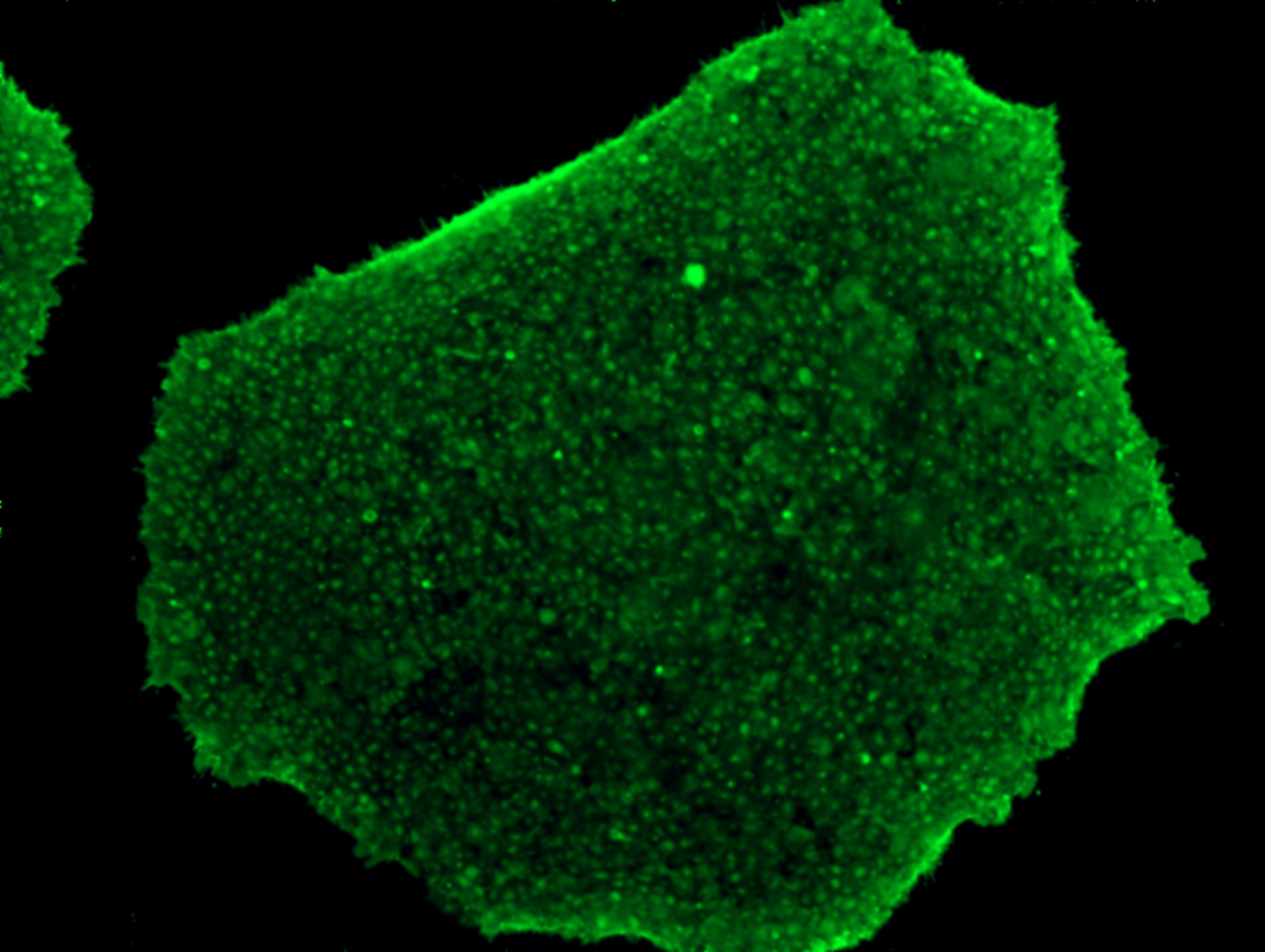

Supplement: Supplementary file 12 — Source Data for Figure 1E [file EMMM-12-e12013-s010.zip › Source_data_Fig_1E/ALX1_165L_165F_SSEA4.tif]

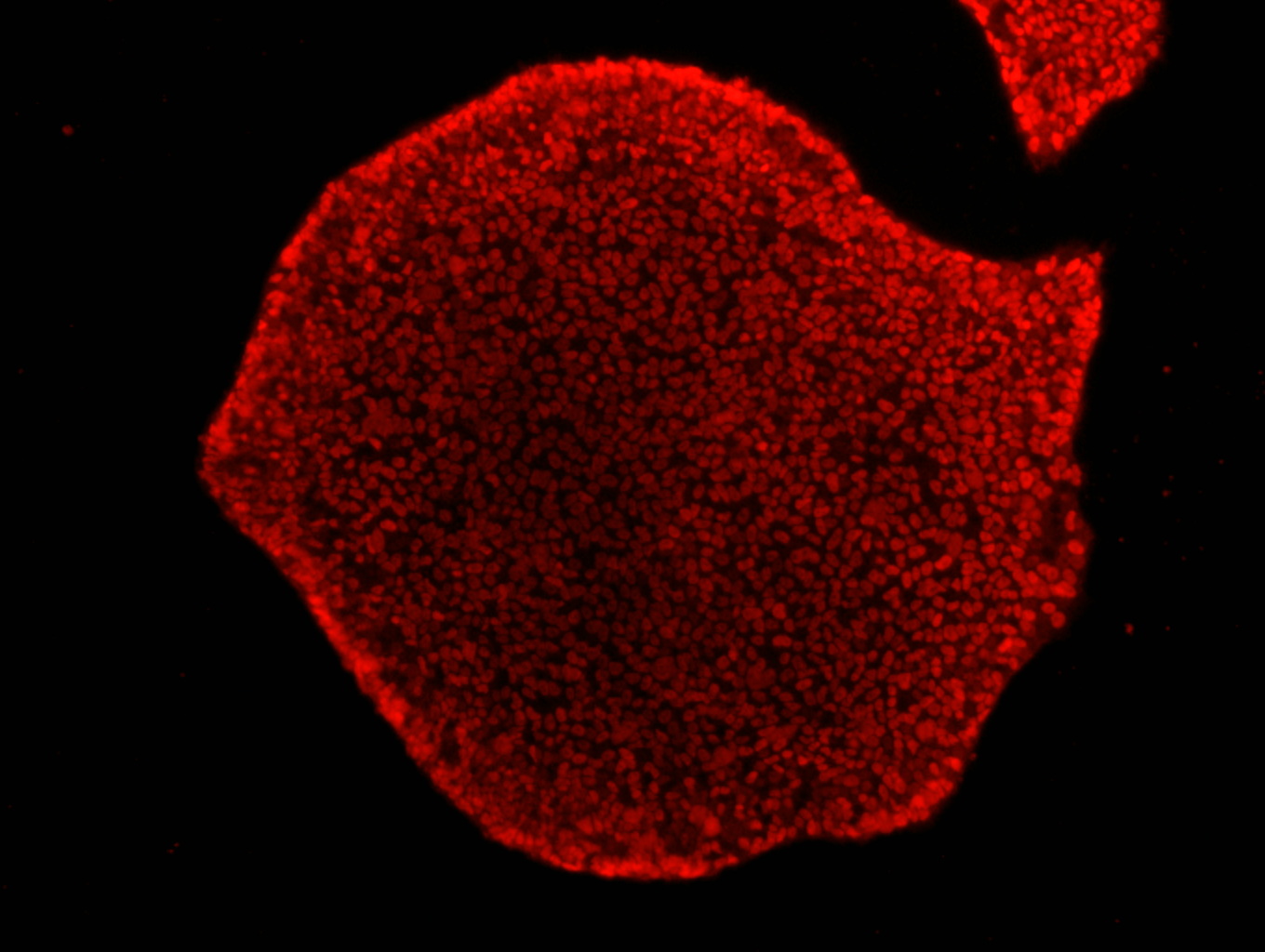

Supplement: Supplementary file 12 — Source Data for Figure 1E [file EMMM-12-e12013-s010.zip › Source_data_Fig_1E/Control_OCT4.tif]

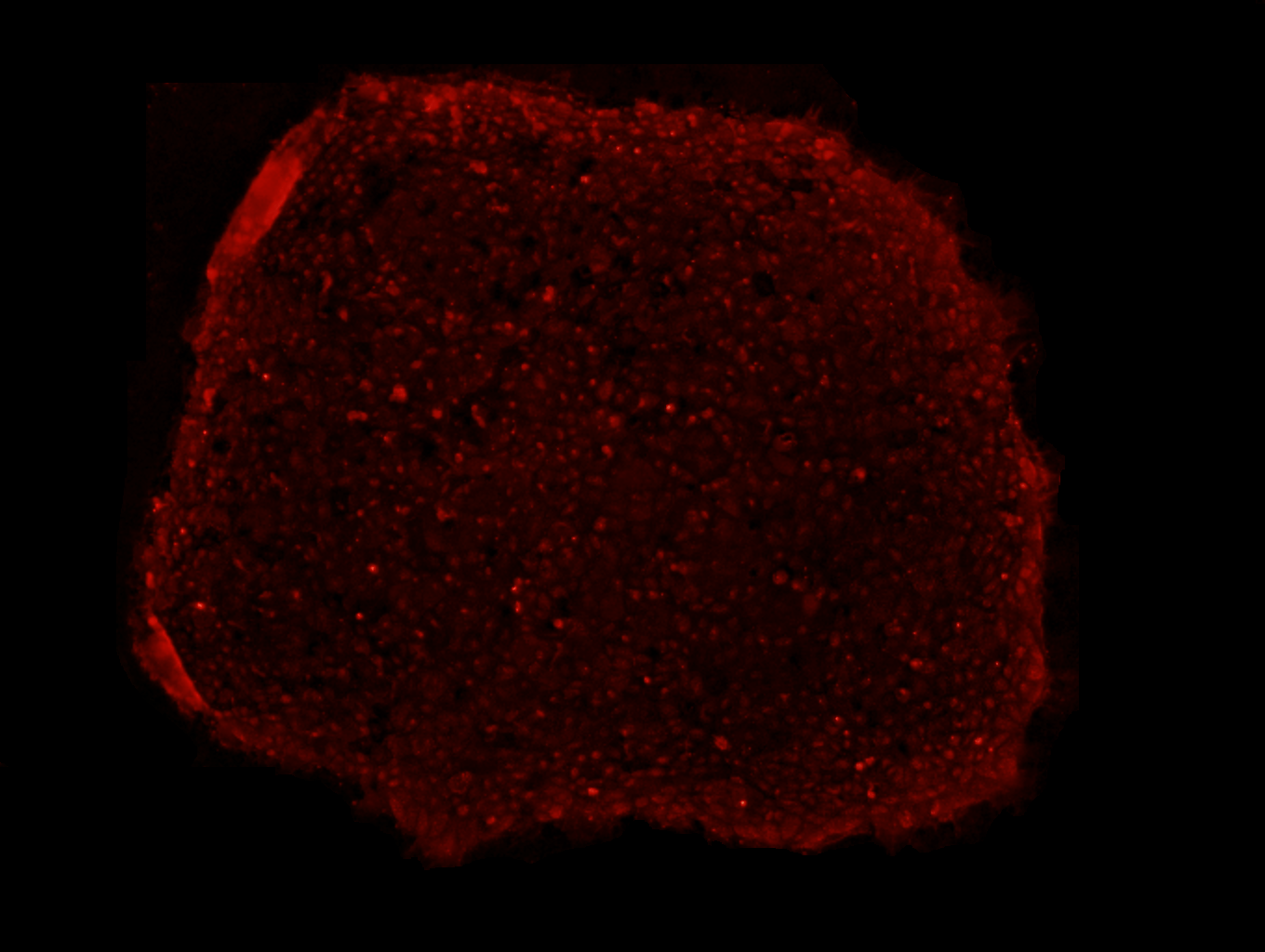

Supplement: Supplementary file 12 — Source Data for Figure 1E [file EMMM-12-e12013-s010.zip › Source_data_Fig_1E/ALX1_165L_165F_TRA-1-60.tif]

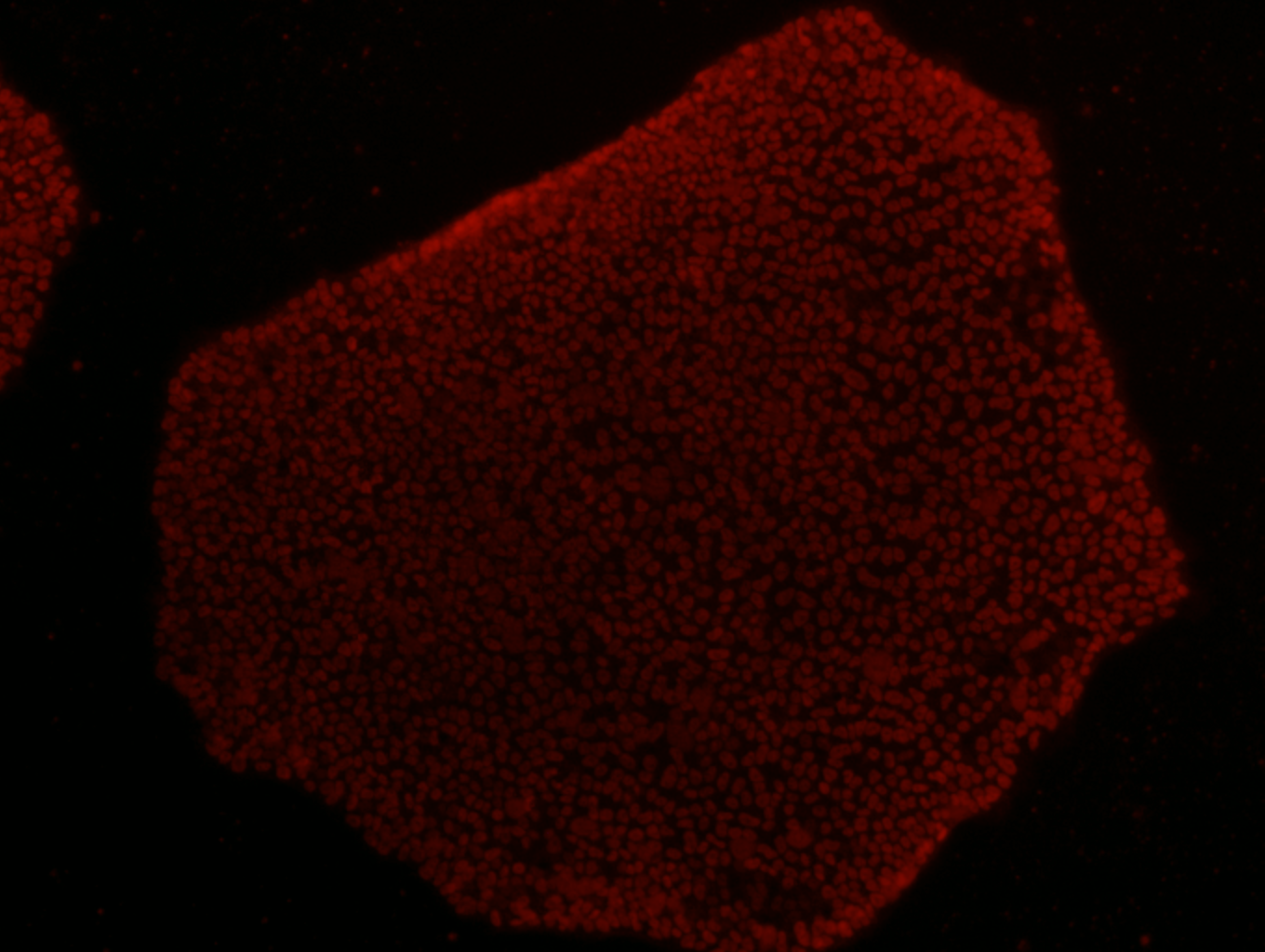

Supplement: Supplementary file 12 — Source Data for Figure 1E [file EMMM-12-e12013-s010.zip › Source_data_Fig_1E/ALX1_165L_165F_OCT4.tif]

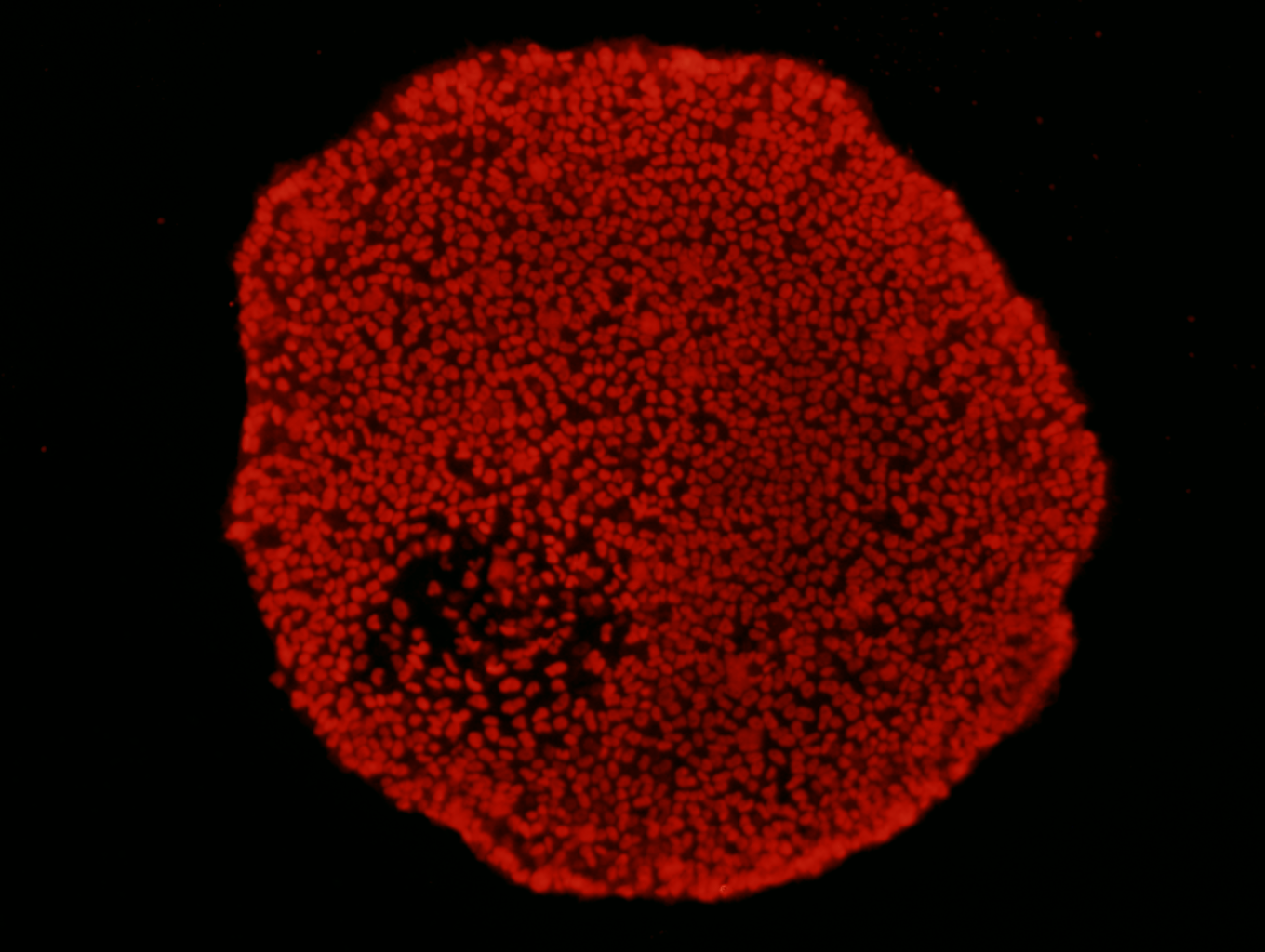

Supplement: Supplementary file 12 — Source Data for Figure 1E [file EMMM-12-e12013-s010.zip › Source_data_Fig_1E/ALX1_165F_165F_OCT4.tif]

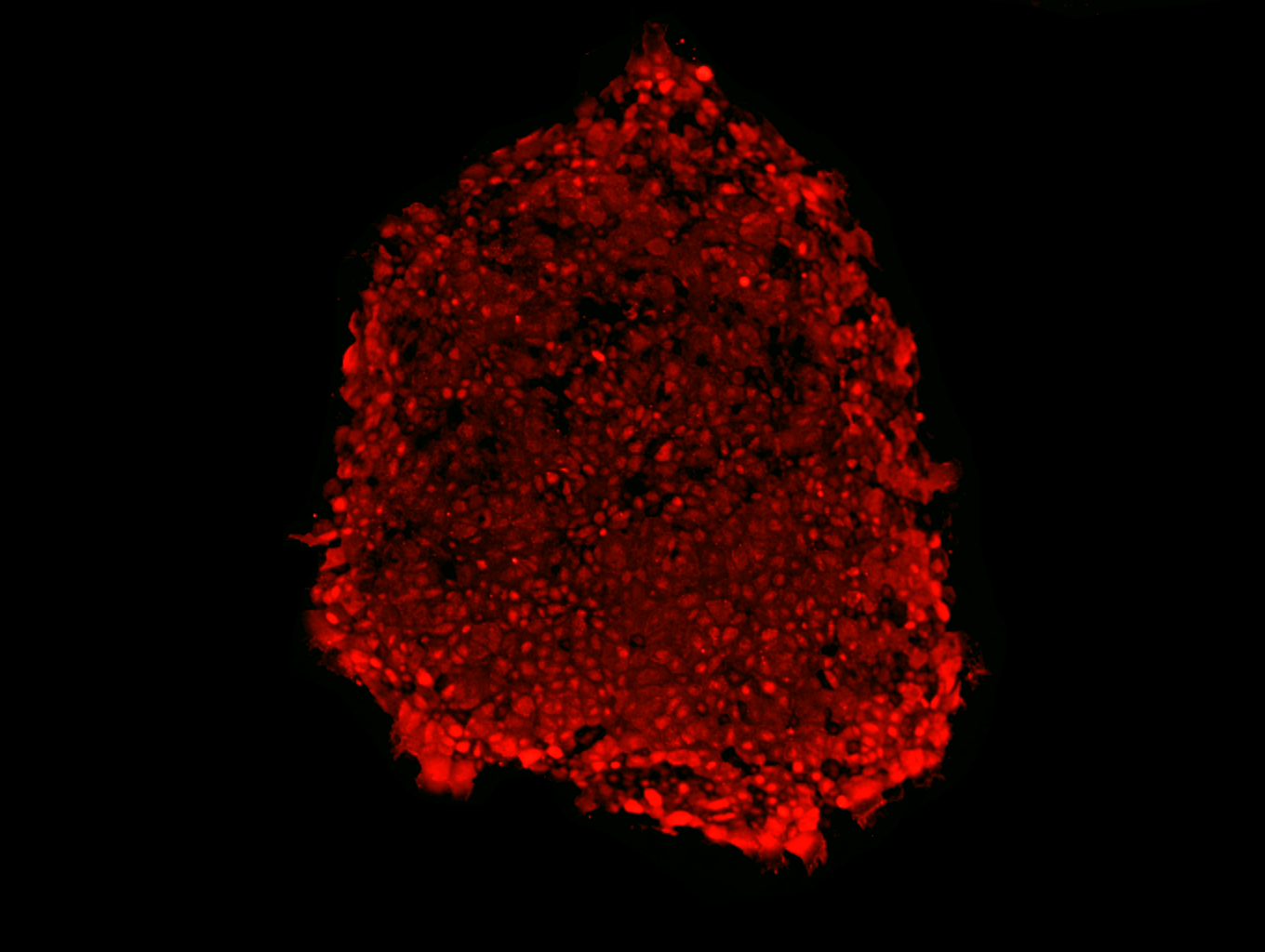

Supplement: Supplementary file 12 — Source Data for Figure 1E [file EMMM-12-e12013-s010.zip › Source_data_Fig_1E/Control_TRA-1-60.tif]

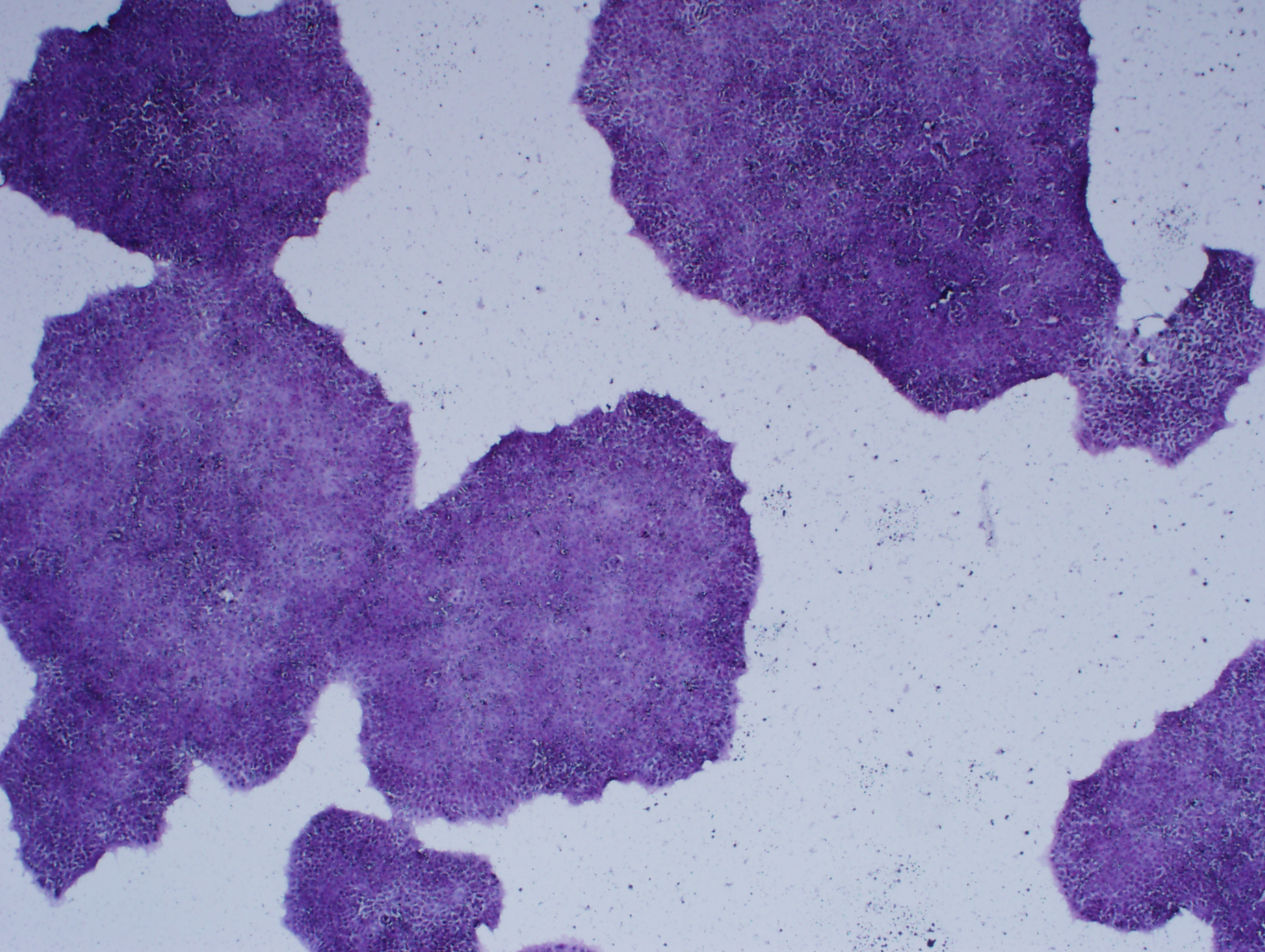

Supplement: Supplementary file 12 — Source Data for Figure 1E [file EMMM-12-e12013-s010.zip › Source_data_Fig_1E/Control_ALP.tif]

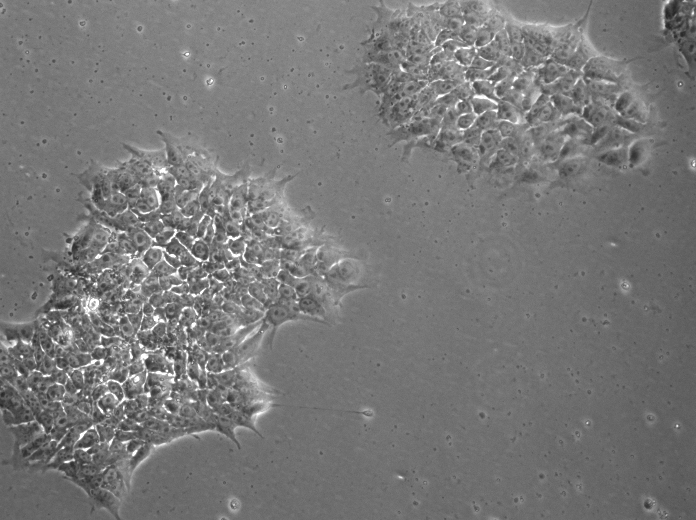

Supplement: Supplementary file 13 — Source Data for Figure 2B [file EMMM-12-e12013-s011.zip › Source_data_Fig_2B/ALX165F_165F_Day_0.TIF]

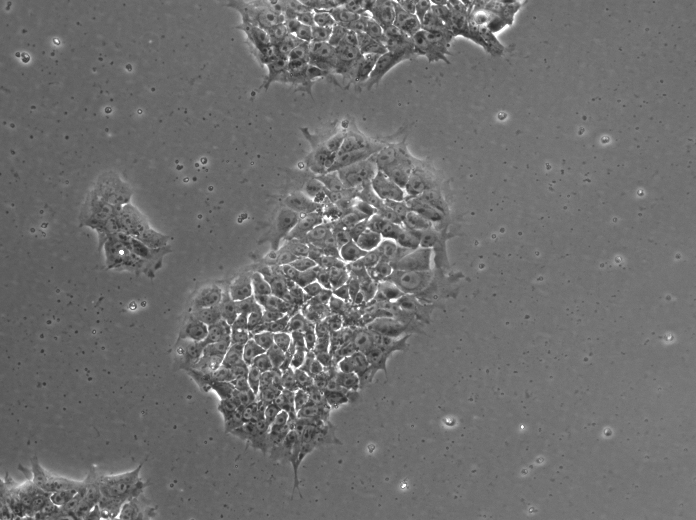

Supplement: Supplementary file 13 — Source Data for Figure 2B [file EMMM-12-e12013-s011.zip › Source_data_Fig_2B/Conrol_Day_0.TIF]

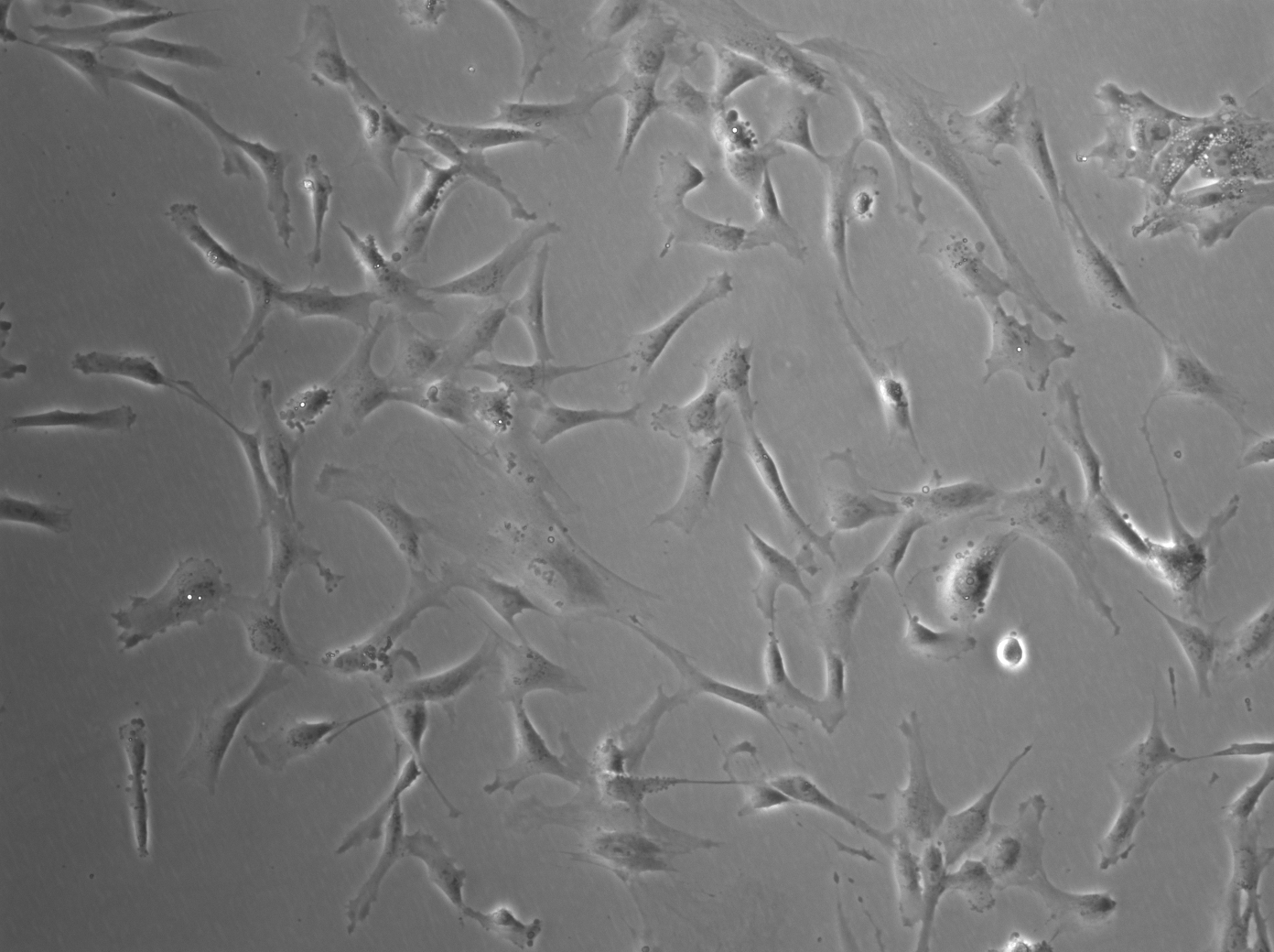

Supplement: Supplementary file 13 — Source Data for Figure 2B [file EMMM-12-e12013-s011.zip › Source_data_Fig_2B/Control_Day_14.TIF]

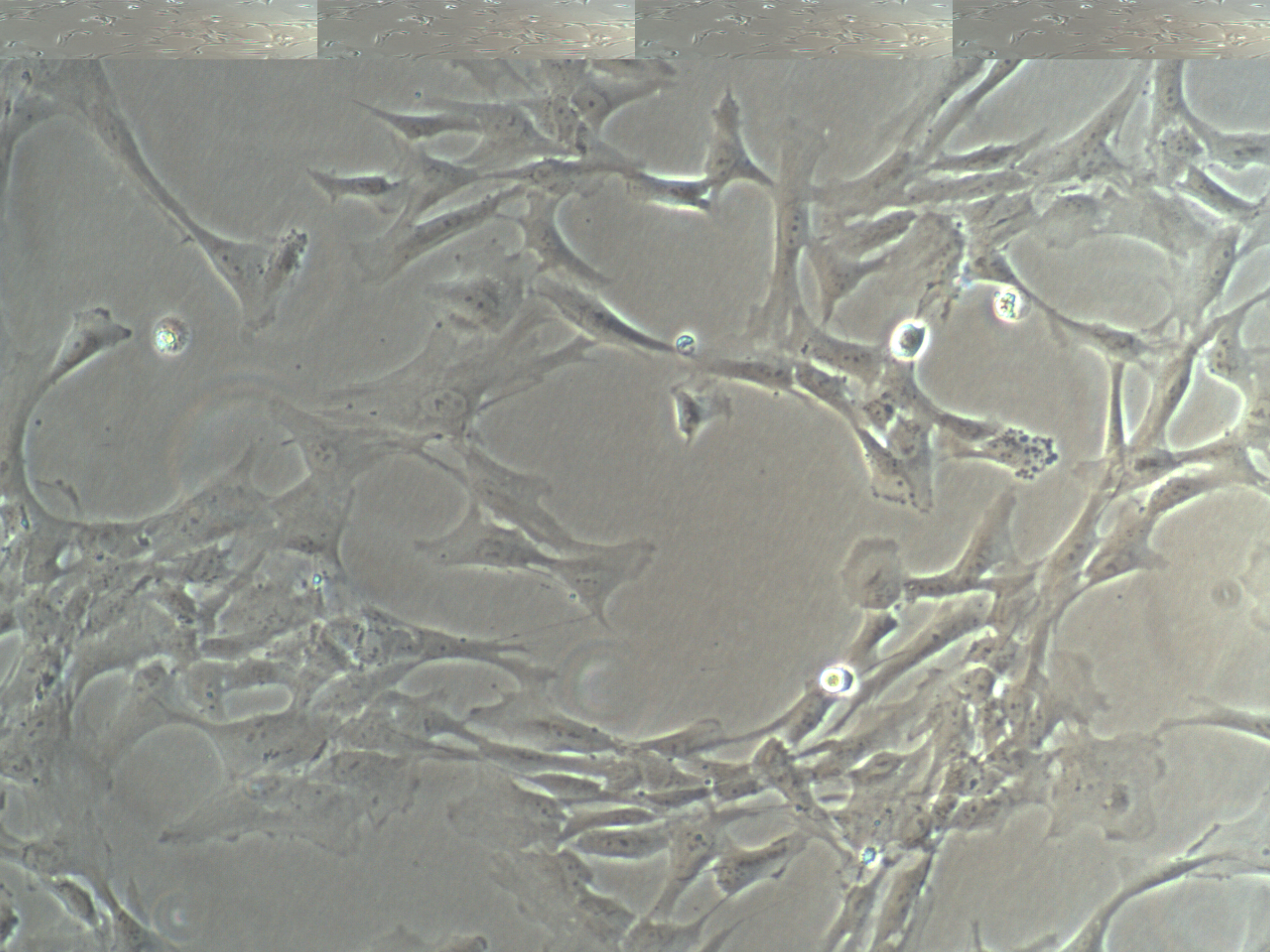

Supplement: Supplementary file 13 — Source Data for Figure 2B [file EMMM-12-e12013-s011.zip › Source_data_Fig_2B/Control_passage_4.TIF]

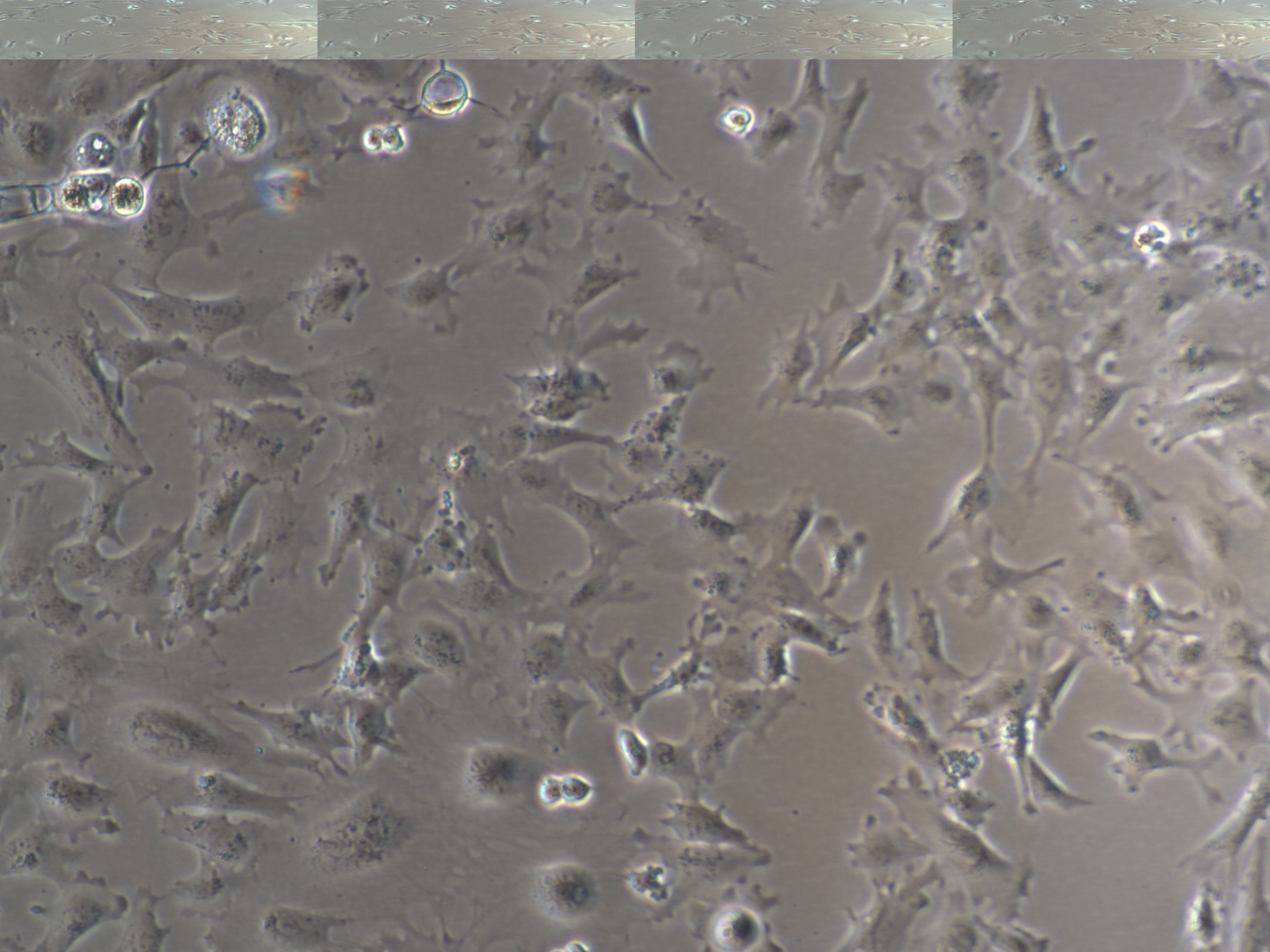

Supplement: Supplementary file 13 — Source Data for Figure 2B [file EMMM-12-e12013-s011.zip › Source_data_Fig_2B/ALX1_165F_165f_passage_4.TIF]

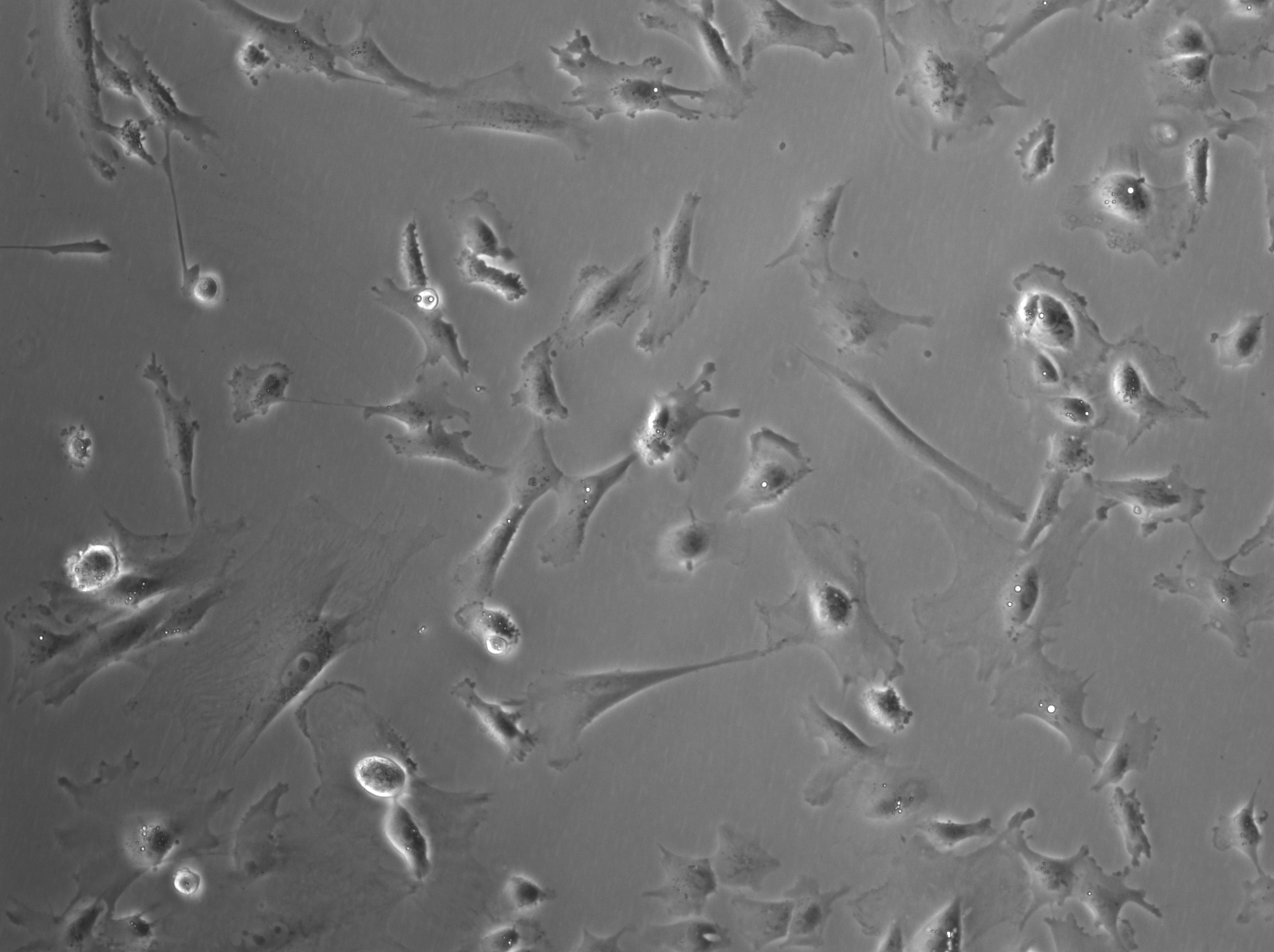

Supplement: Supplementary file 13 — Source Data for Figure 2B [file EMMM-12-e12013-s011.zip › Source_data_Fig_2B/ALX1_165F_165F_Day_14.TIF]

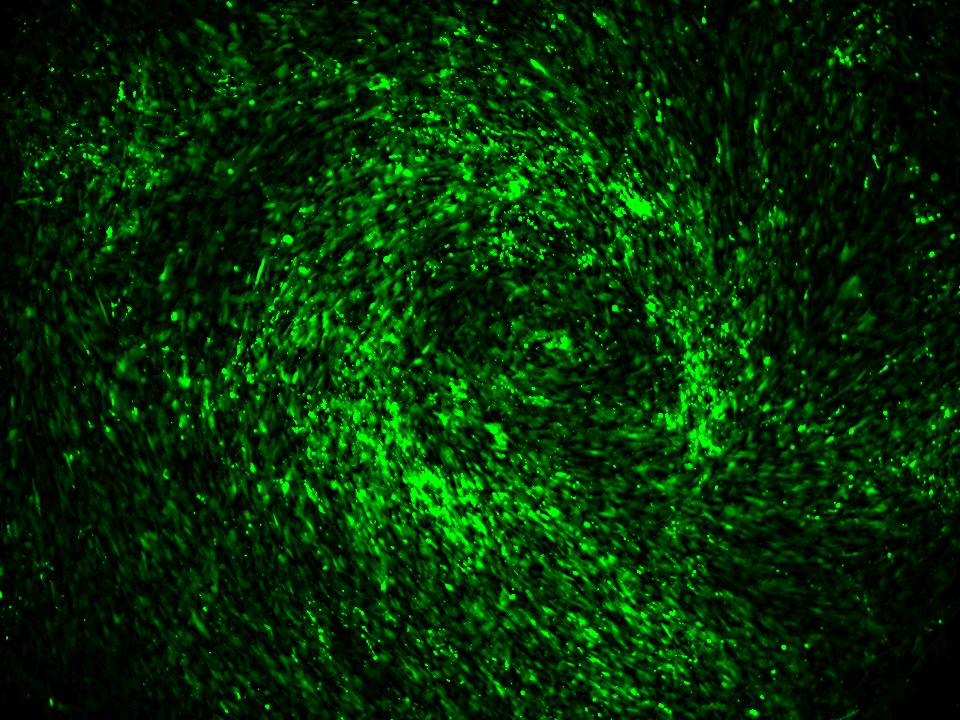

Supplement: Supplementary file 14 — Source Data for Figure 5 [file EMMM-12-e12013-s012.zip › Source_data_Fig_5/5A/Control_24h-1.tif]

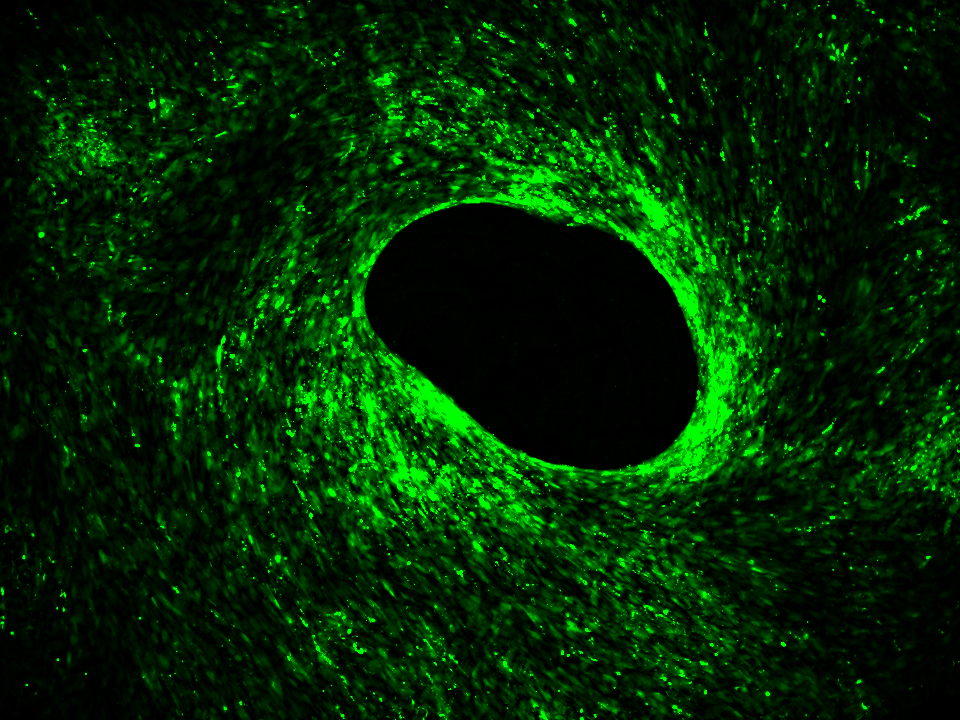

Supplement: Supplementary file 14 — Source Data for Figure 5 [file EMMM-12-e12013-s012.zip › Source_data_Fig_5/5A/Control_0h-1.tif]

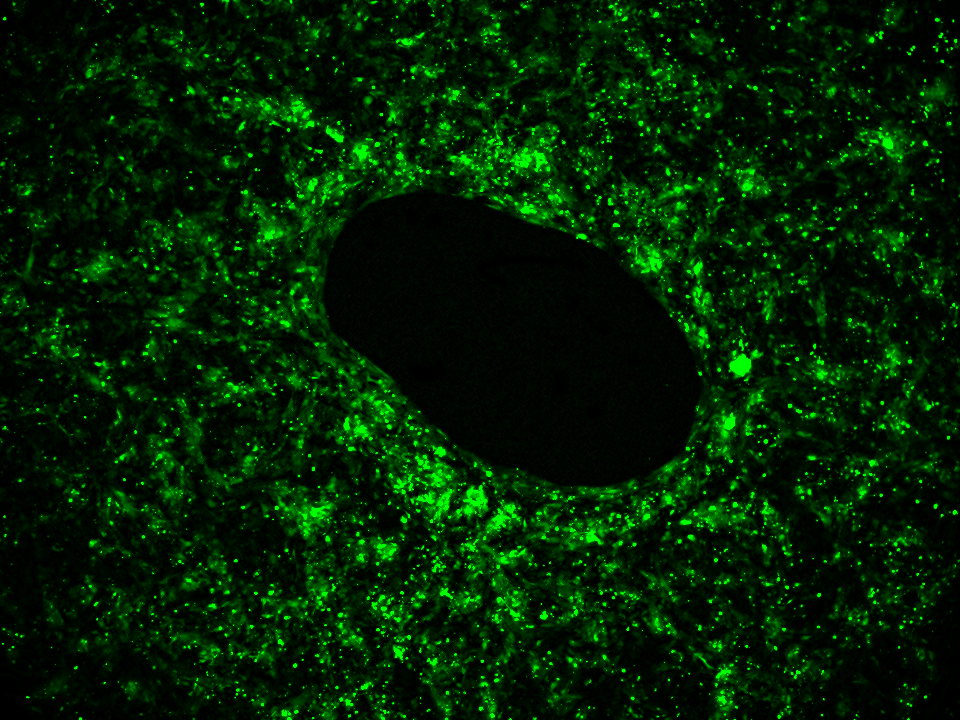

Supplement: Supplementary file 14 — Source Data for Figure 5 [file EMMM-12-e12013-s012.zip › Source_data_Fig_5/5A/Patient_0h.tif]

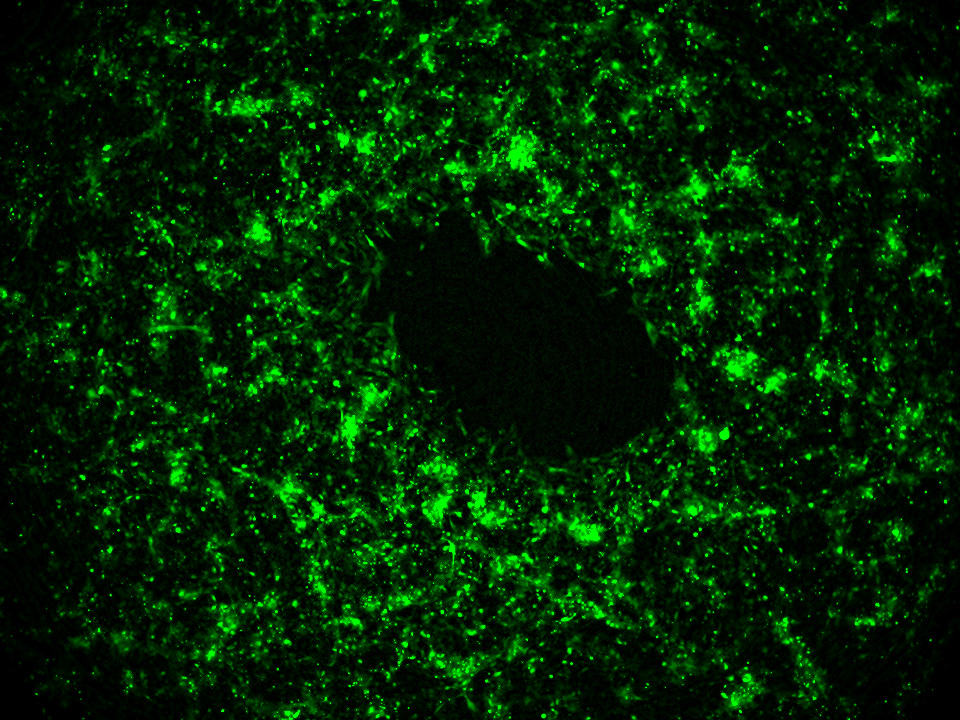

Supplement: Supplementary file 14 — Source Data for Figure 5 [file EMMM-12-e12013-s012.zip › Source_data_Fig_5/5A/Patient_24h.tif]

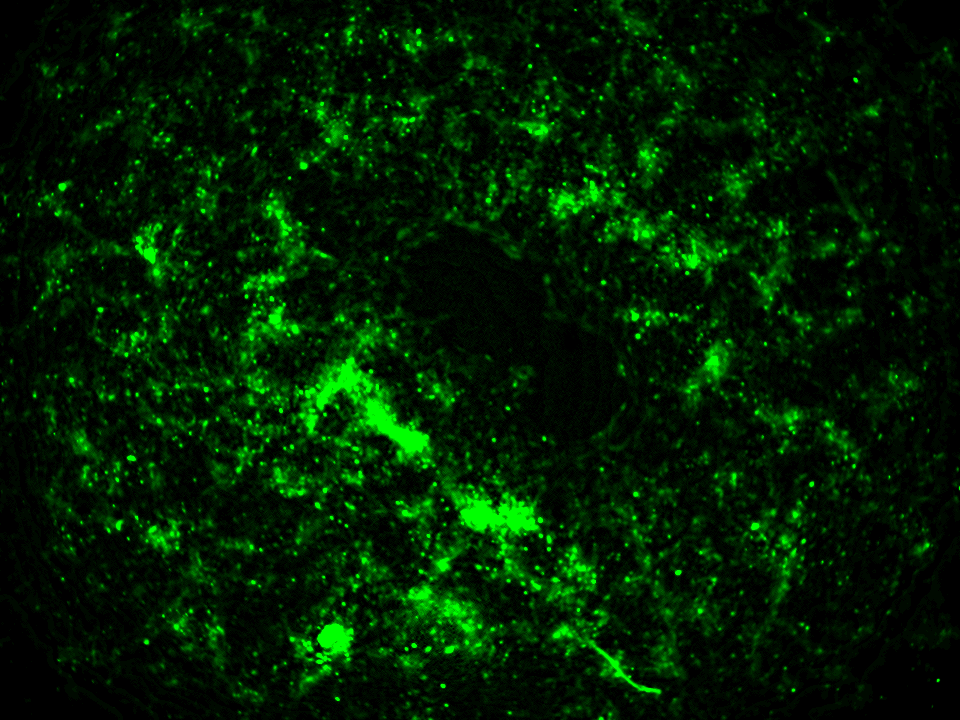

Supplement: Supplementary file 14 — Source Data for Figure 5 [file EMMM-12-e12013-s012.zip › Source_data_Fig_5/5C/BMP2_24h.tif]

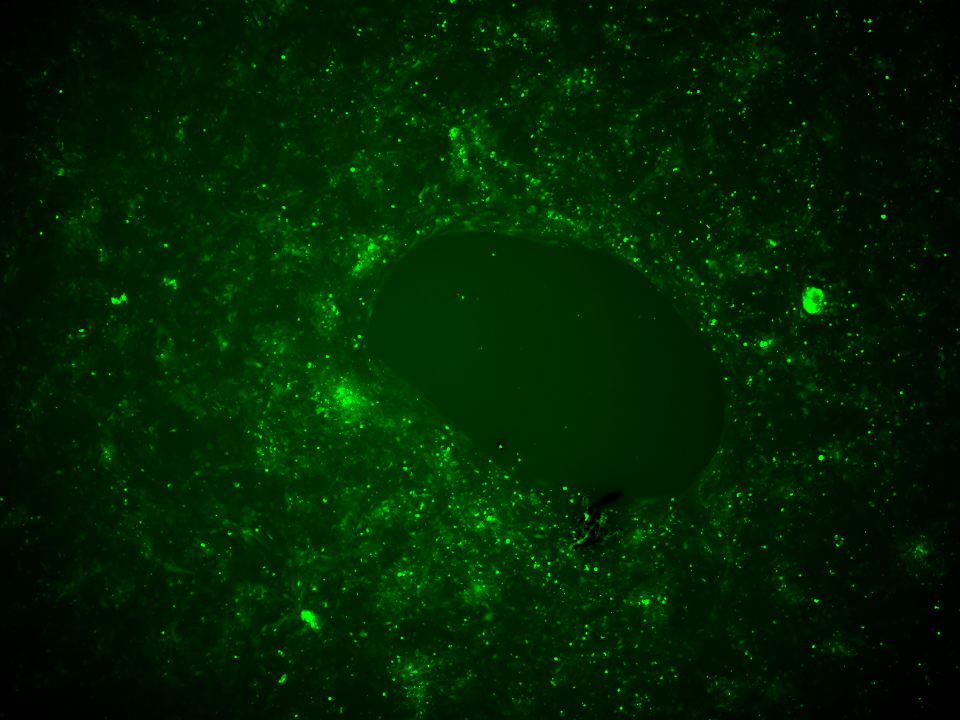

Supplement: Supplementary file 14 — Source Data for Figure 5 [file EMMM-12-e12013-s012.zip › Source_data_Fig_5/5C/CV2_50ng_ml_0h.tif]

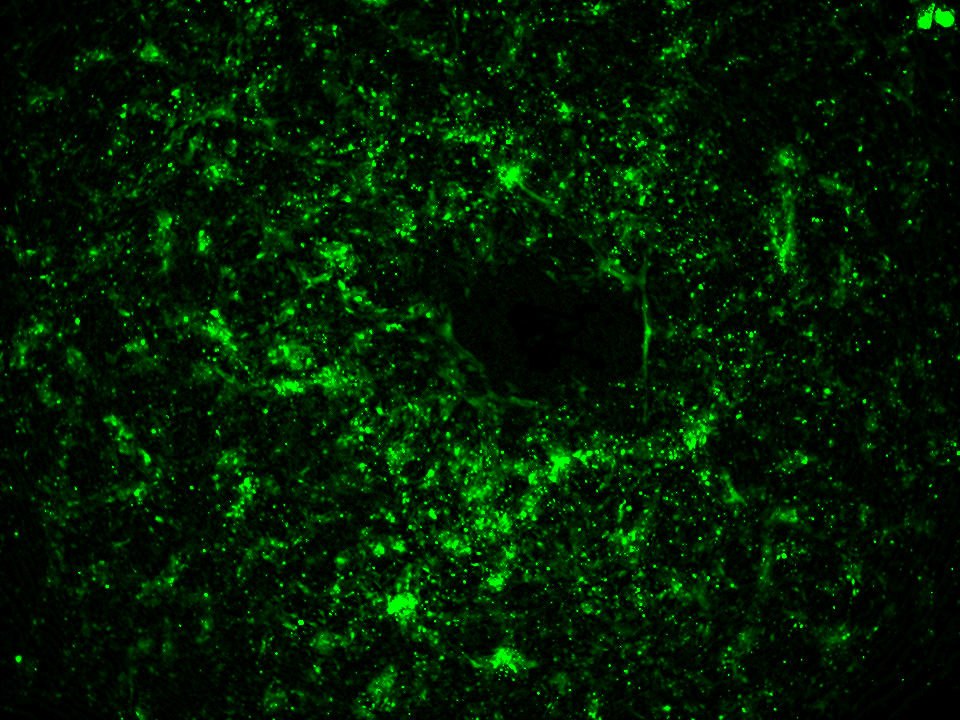

Supplement: Supplementary file 14 — Source Data for Figure 5 [file EMMM-12-e12013-s012.zip › Source_data_Fig_5/5C/BMP2_CV2_24h.tif]

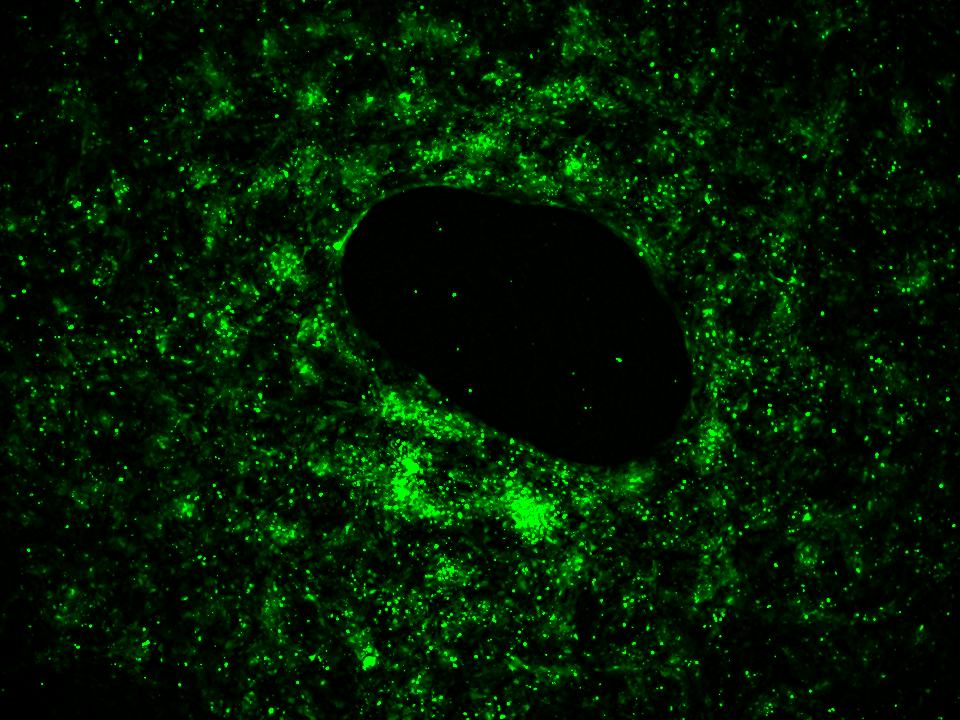

Supplement: Supplementary file 14 — Source Data for Figure 5 [file EMMM-12-e12013-s012.zip › Source_data_Fig_5/5C/BMP2_10ng_ml_0h.tif]

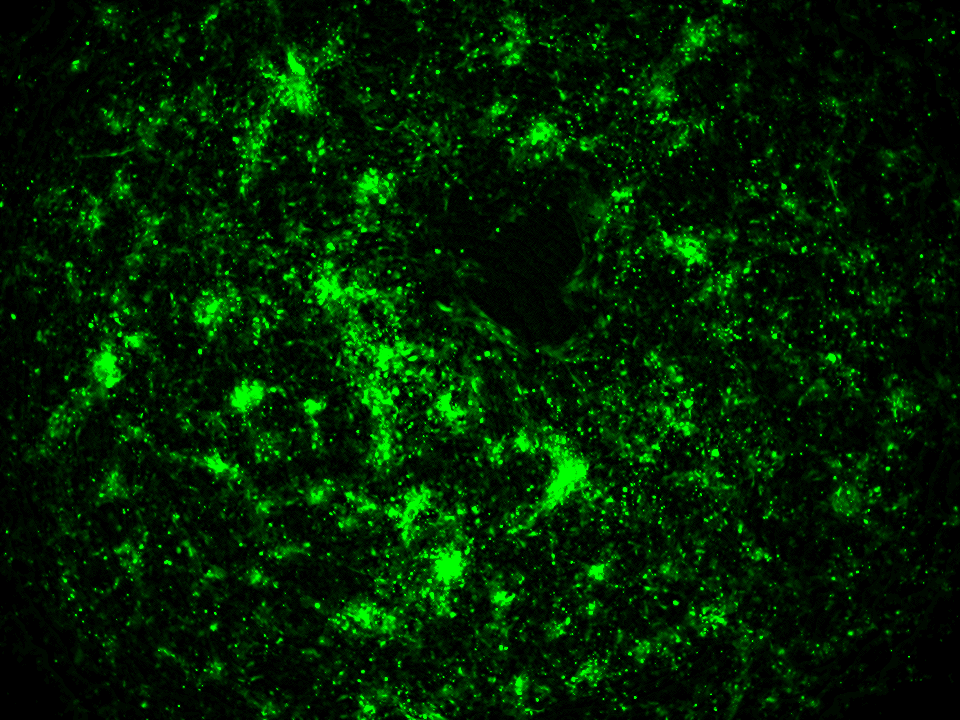

Supplement: Supplementary file 14 — Source Data for Figure 5 [file EMMM-12-e12013-s012.zip › Source_data_Fig_5/5C/CV2_100ng_ml_24h.tif]

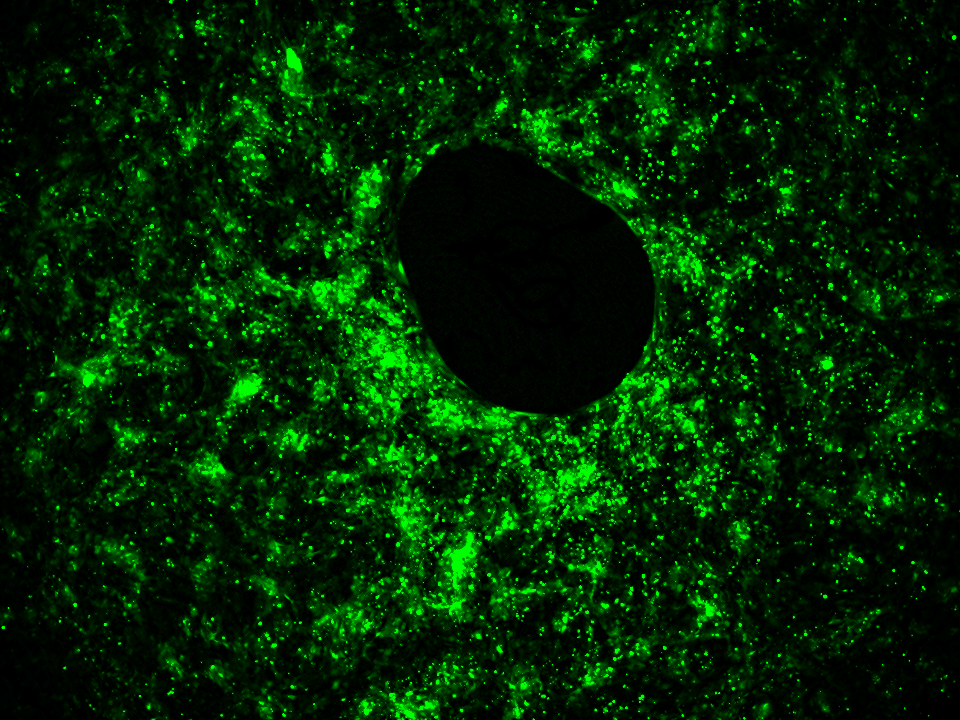

Supplement: Supplementary file 14 — Source Data for Figure 5 [file EMMM-12-e12013-s012.zip › Source_data_Fig_5/5C/CV2_100ng_ml_0h.tif]

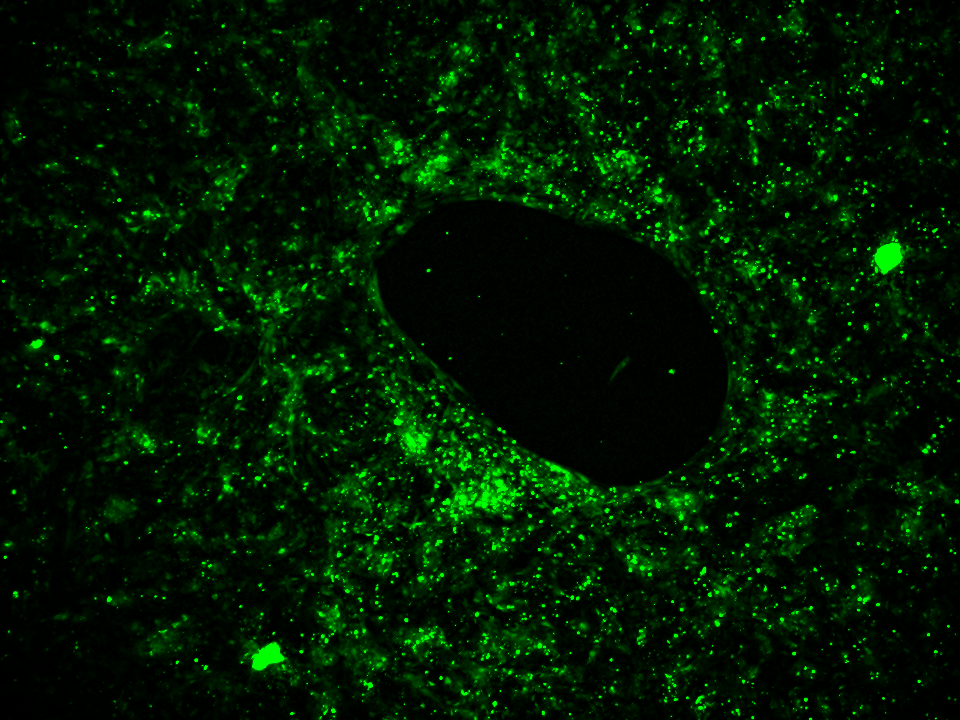

Supplement: Supplementary file 14 — Source Data for Figure 5 [file EMMM-12-e12013-s012.zip › Source_data_Fig_5/5C/BMP2_50ng_ml_0h.tif]

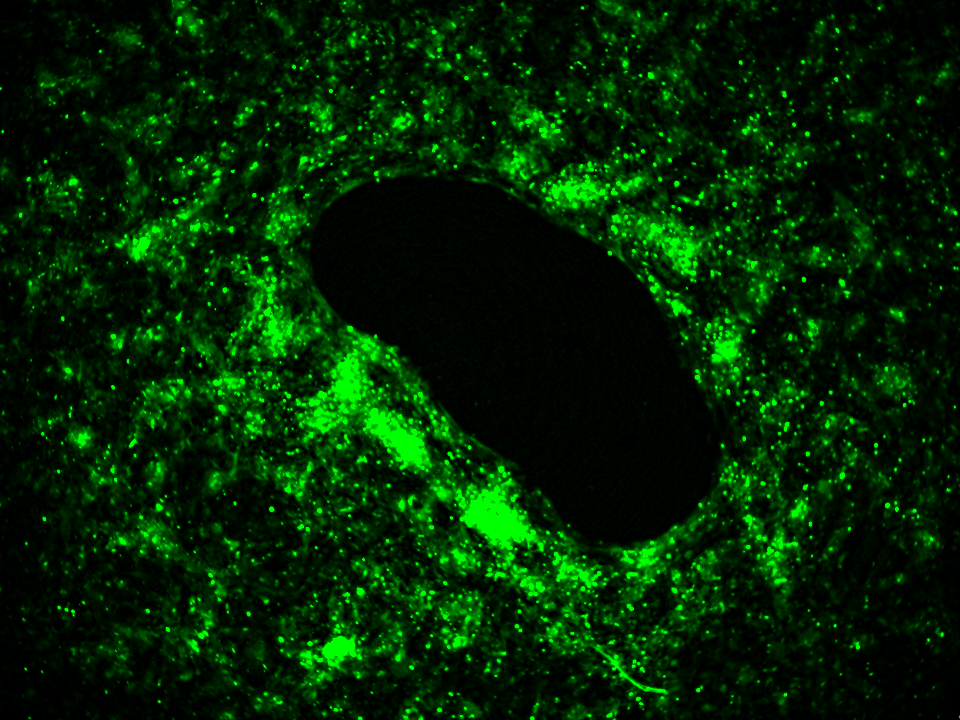

Supplement: Supplementary file 14 — Source Data for Figure 5 [file EMMM-12-e12013-s012.zip › Source_data_Fig_5/5C/BMP2_0h.tif]

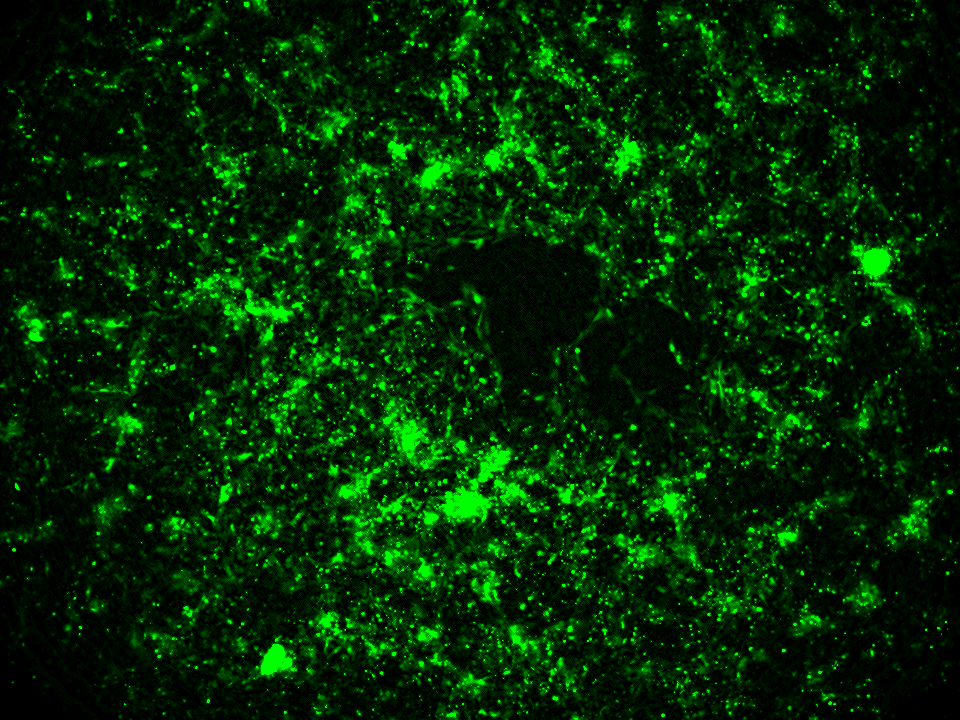

Supplement: Supplementary file 14 — Source Data for Figure 5 [file EMMM-12-e12013-s012.zip › Source_data_Fig_5/5C/BMP2_50ng_ml_24h.tif]

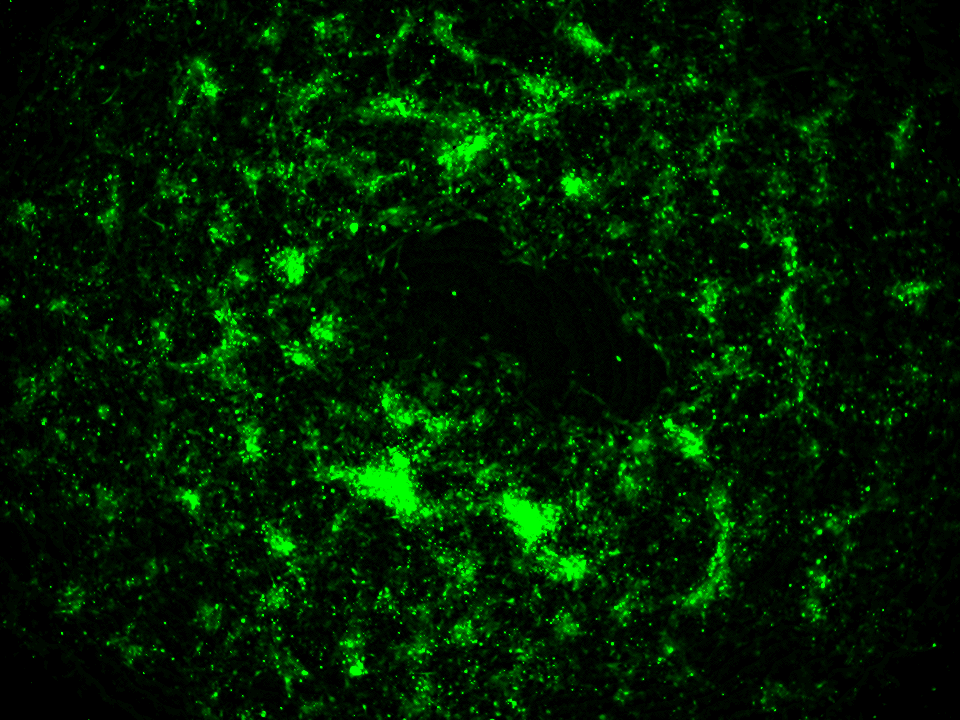

Supplement: Supplementary file 14 — Source Data for Figure 5 [file EMMM-12-e12013-s012.zip › Source_data_Fig_5/5C/BMP2_10ng_ml_24h.tif]

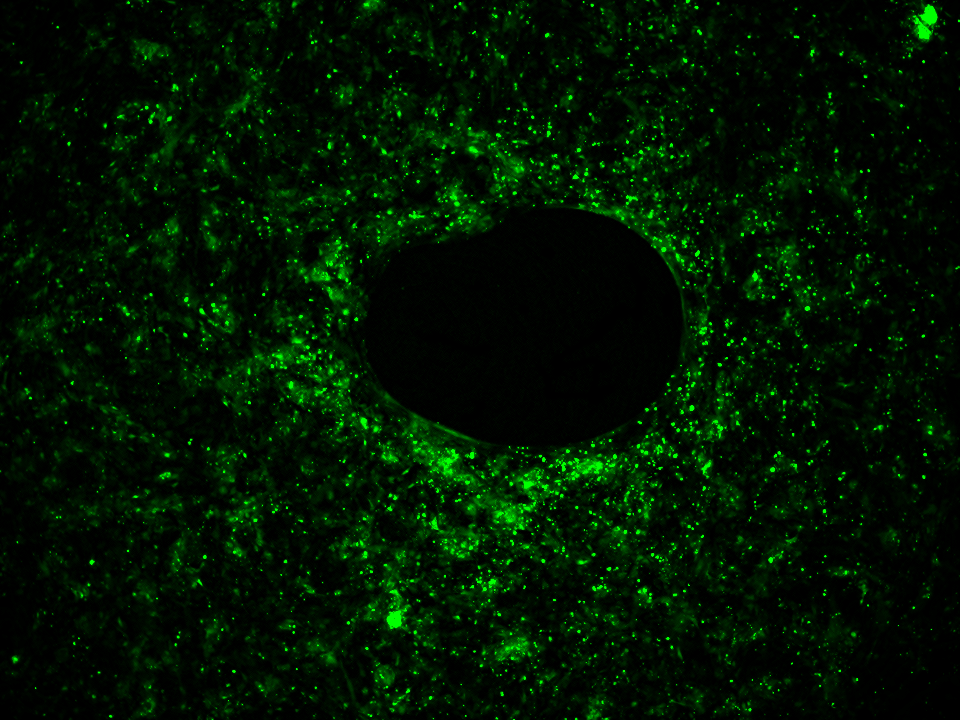

Supplement: Supplementary file 14 — Source Data for Figure 5 [file EMMM-12-e12013-s012.zip › Source_data_Fig_5/5C/BMP2_CV2_0h.tif]

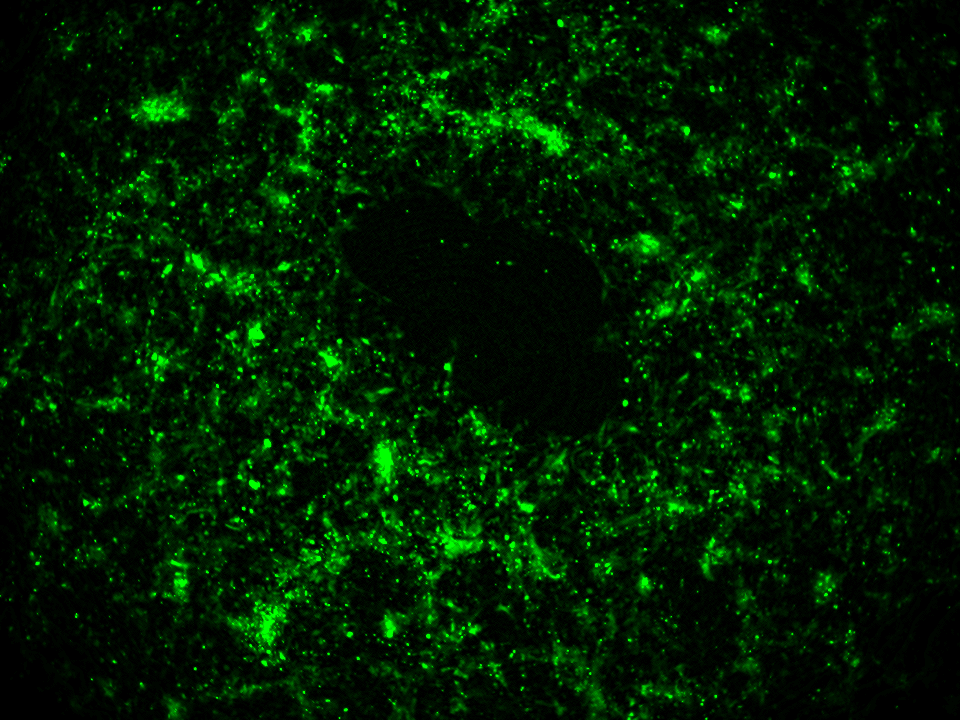

Supplement: Supplementary file 14 — Source Data for Figure 5 [file EMMM-12-e12013-s012.zip › Source_data_Fig_5/5C/Patient_Untreated_24h-2.tif]

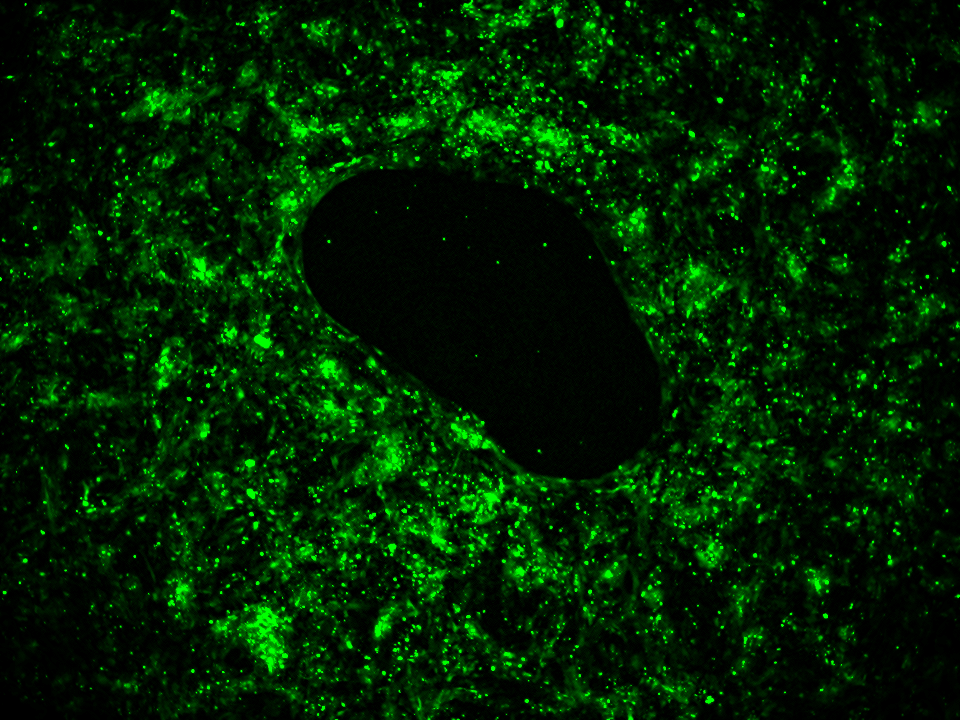

Supplement: Supplementary file 14 — Source Data for Figure 5 [file EMMM-12-e12013-s012.zip › Source_data_Fig_5/5C/Patient_Untreated_0h-2.tif]

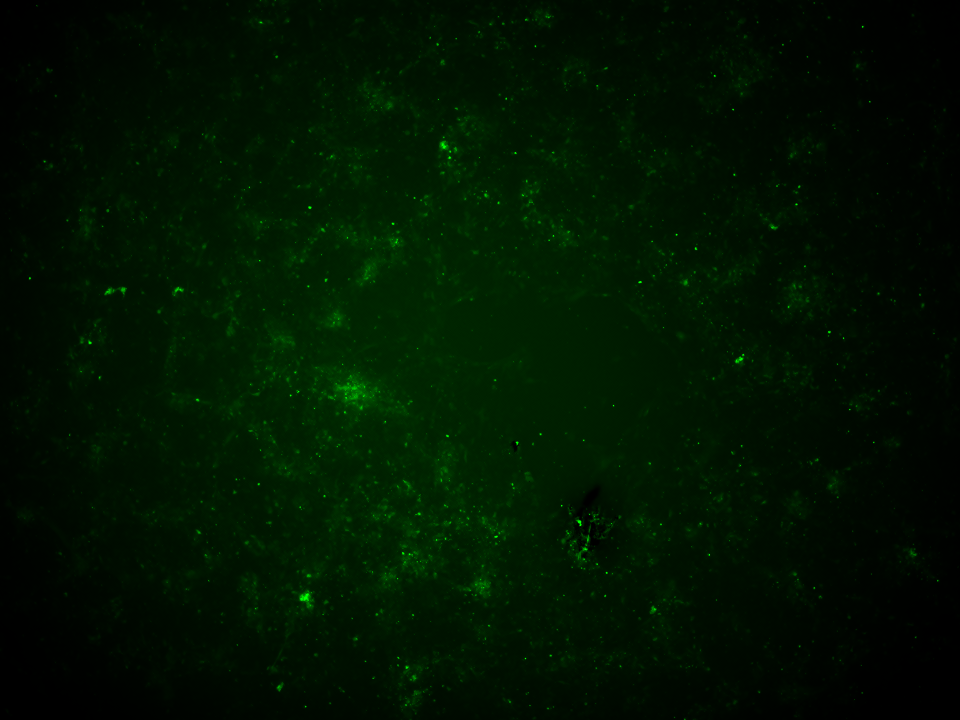

Supplement: Supplementary file 14 — Source Data for Figure 5 [file EMMM-12-e12013-s012.zip › Source_data_Fig_5/5C/CV2_50ng_ml_24h.tif]

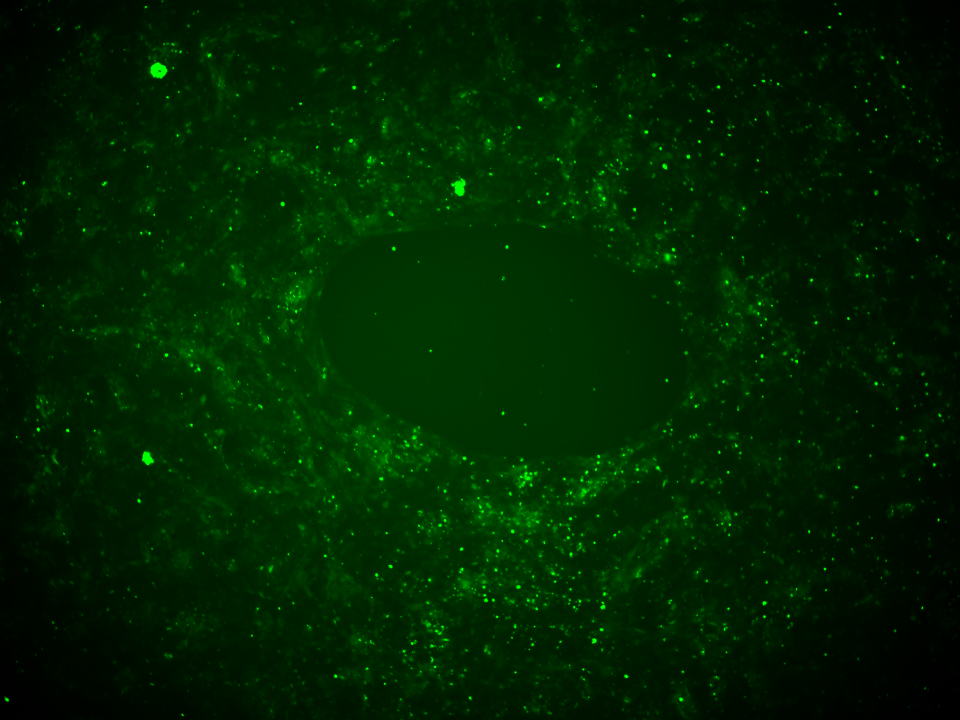

Supplement: Supplementary file 14 — Source Data for Figure 5 [file EMMM-12-e12013-s012.zip › Source_data_Fig_5/5C/CV2_10ng_ml_0h.tif]

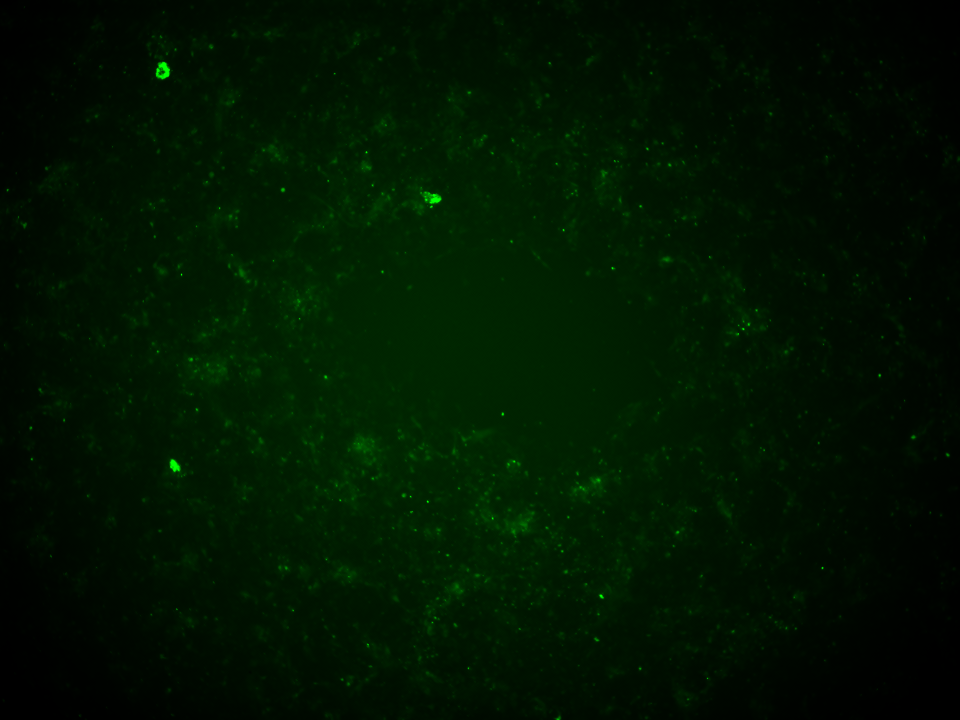

Supplement: Supplementary file 14 — Source Data for Figure 5 [file EMMM-12-e12013-s012.zip › Source_data_Fig_5/5C/CV2_10ng_ml_24h.tif]

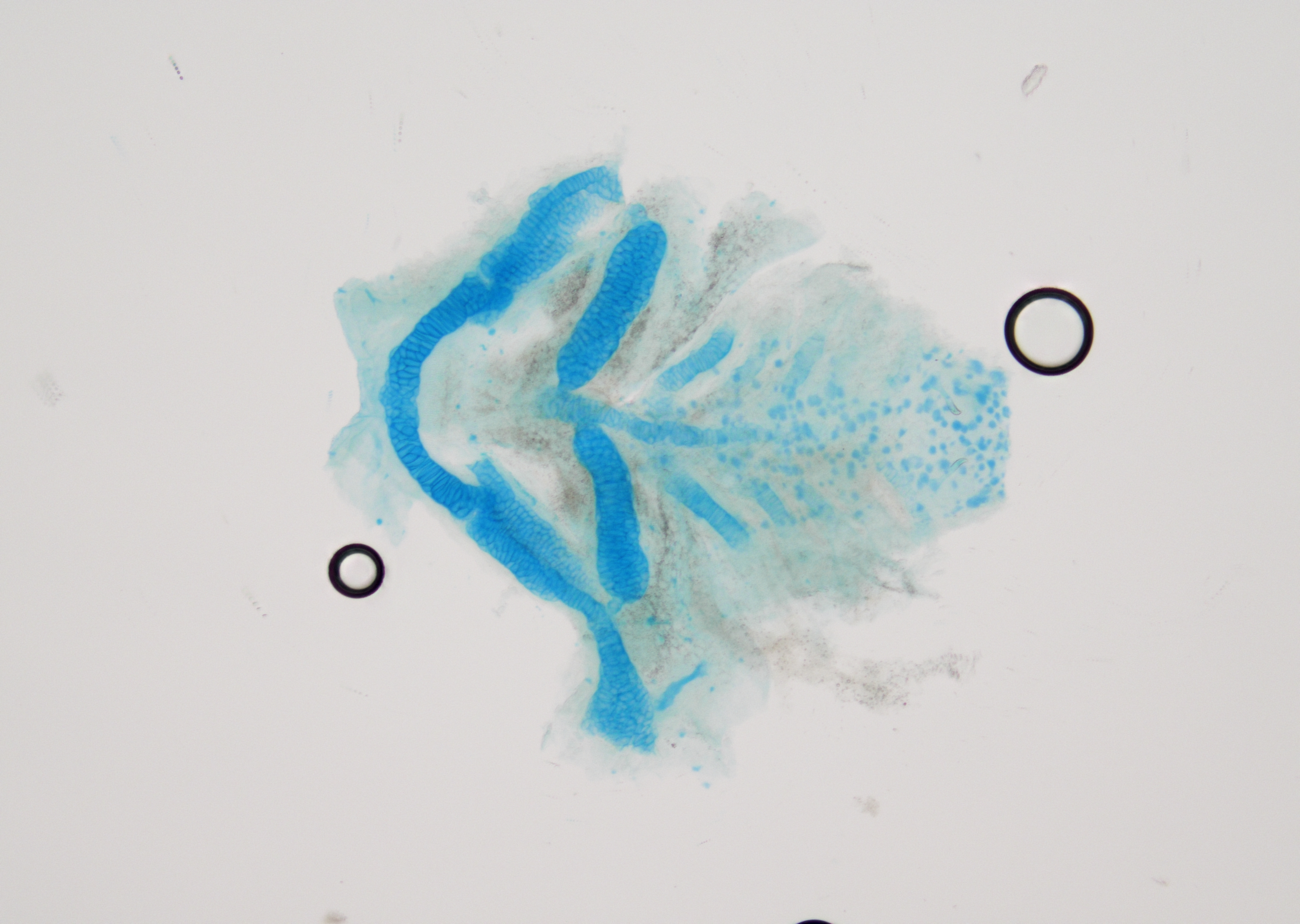

Supplement: Supplementary file 15 — Source Data for Figure 6 [file EMMM-12-e12013-s013.zip › Figure 6A/Alx1KO_Meckels_Mut_JPEG.jpg]

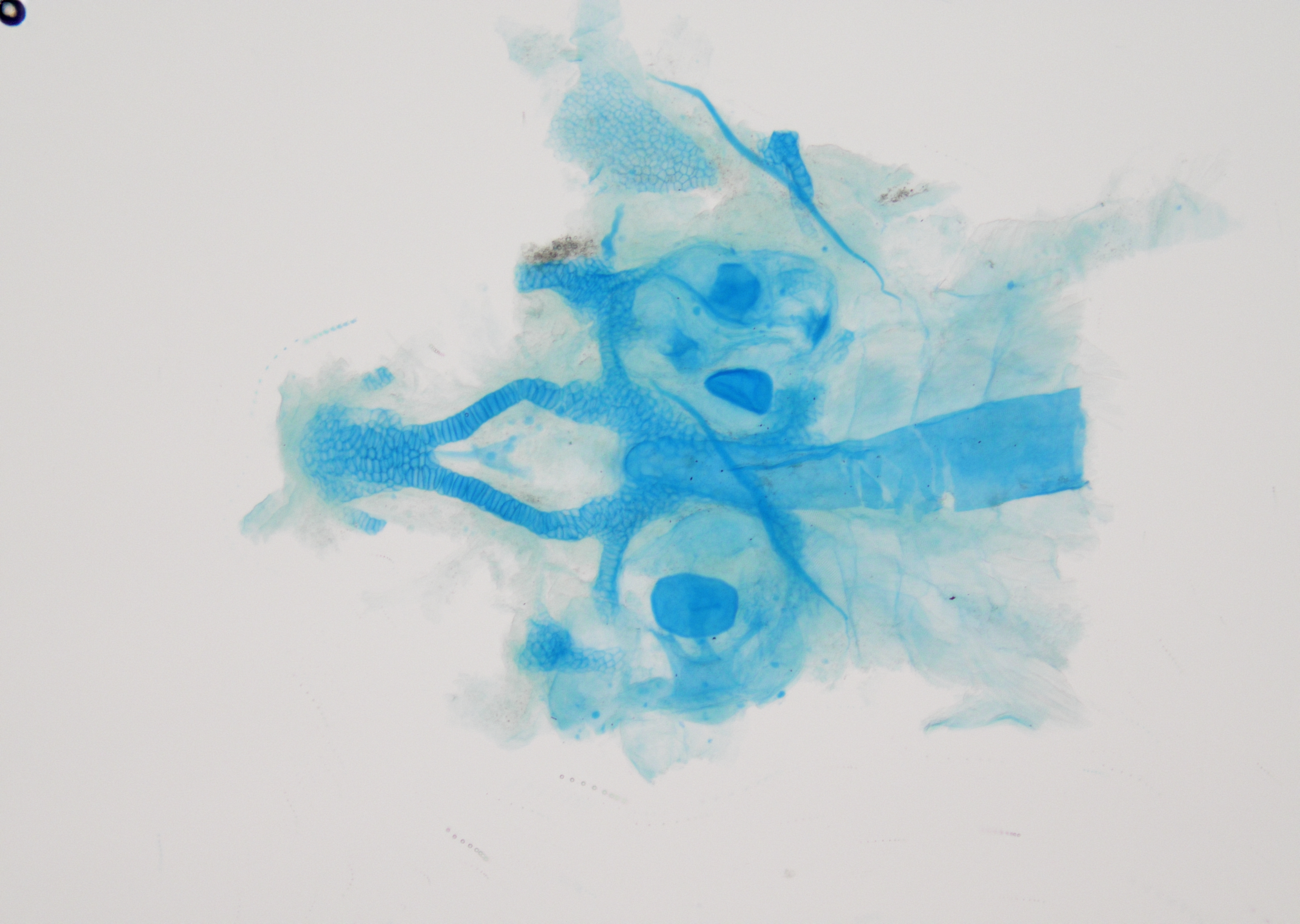

Supplement: Supplementary file 15 — Source Data for Figure 6 [file EMMM-12-e12013-s013.zip › Figure 6A/Alx1KO_Palate_Mut_JPEG.jpg]

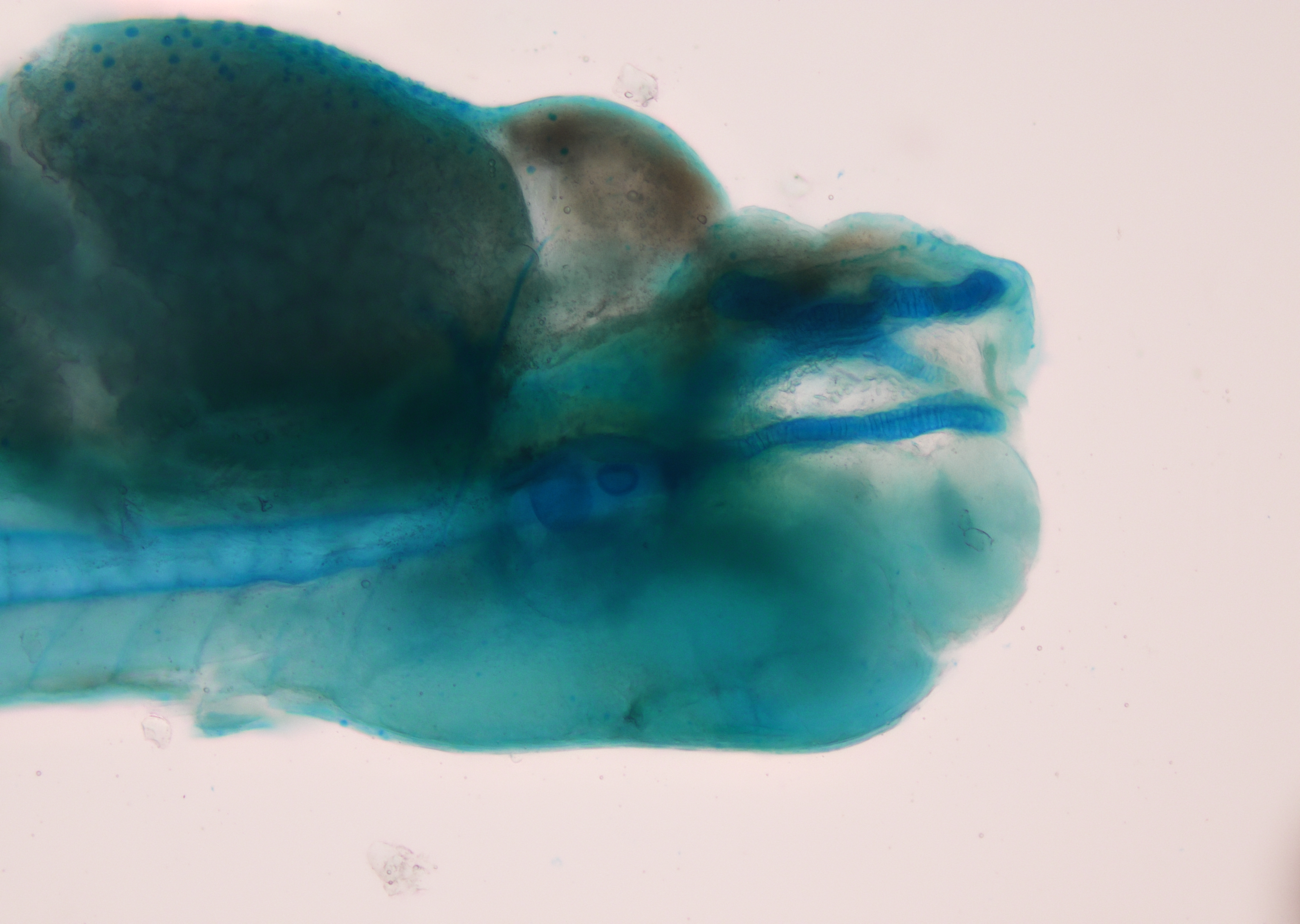

Supplement: Supplementary file 15 — Source Data for Figure 6 [file EMMM-12-e12013-s013.zip › Figure 6A/Alx1KO_Sagittal_Mut_JPEG.jpg]

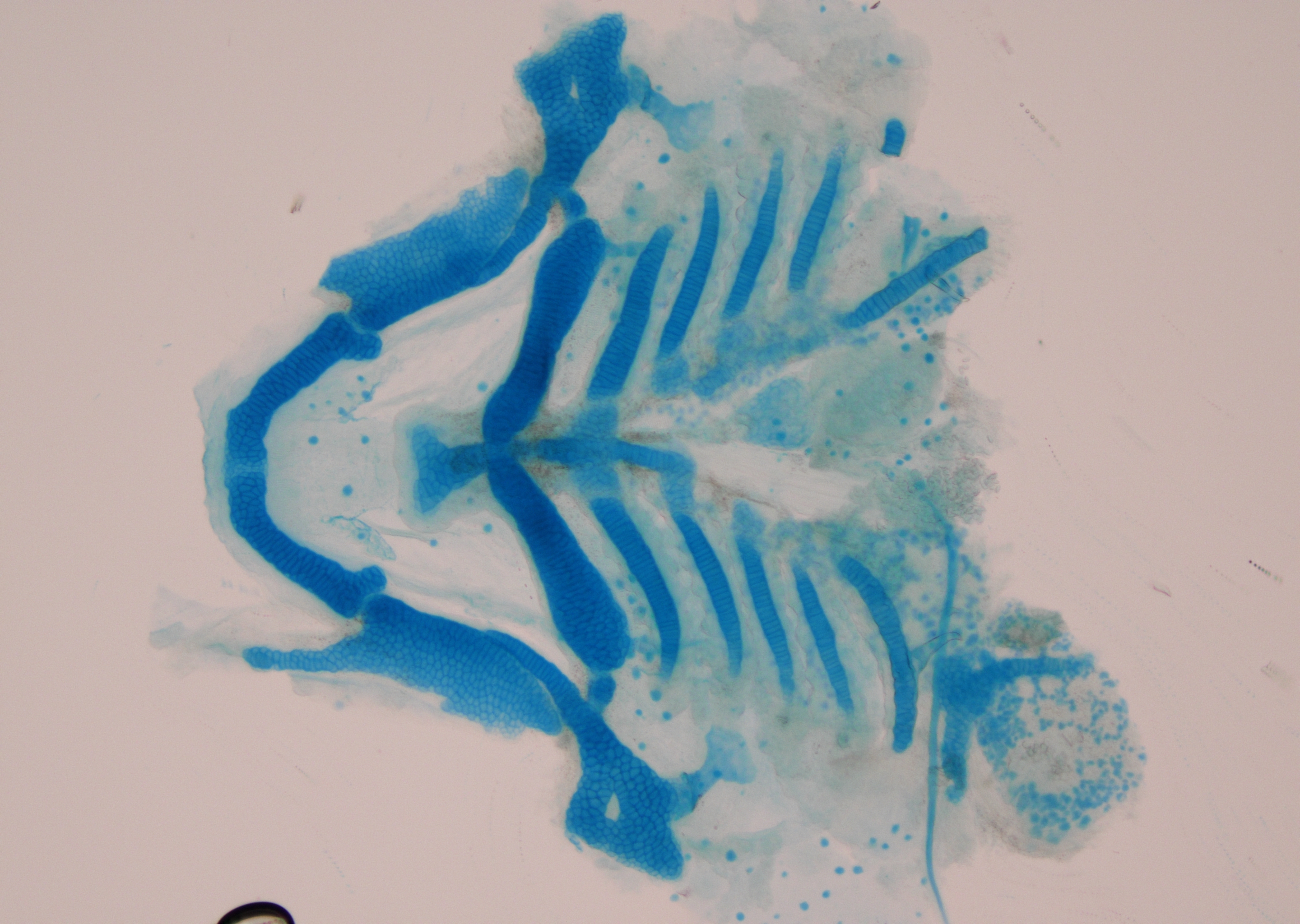

Supplement: Supplementary file 15 — Source Data for Figure 6 [file EMMM-12-e12013-s013.zip › Figure 6A/Alx1KO_uninjected_Meckels_JPEG.jpg]

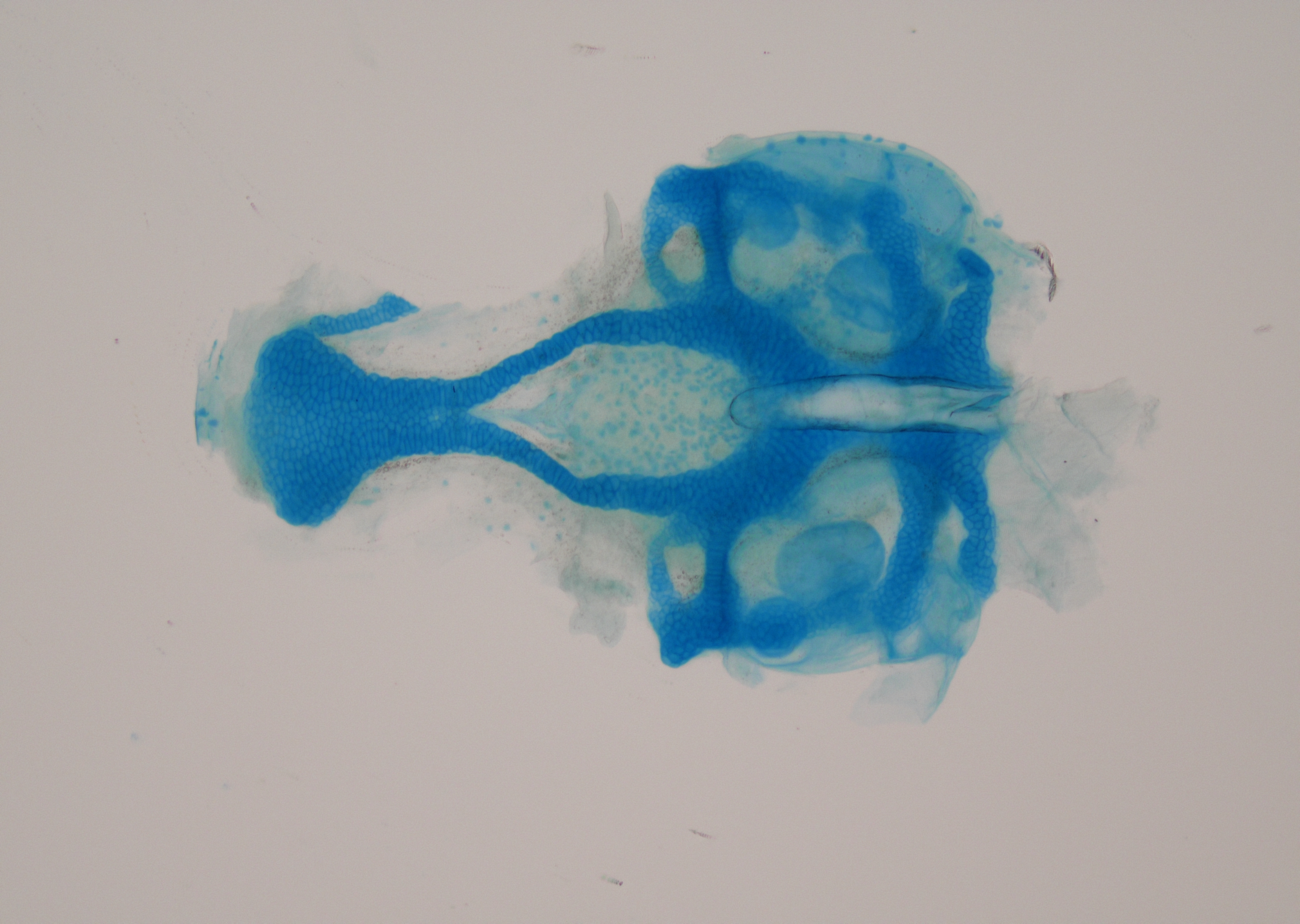

Supplement: Supplementary file 15 — Source Data for Figure 6 [file EMMM-12-e12013-s013.zip › Figure 6A/Alx1KO_uninjected_Palate_JPEG.jpg]

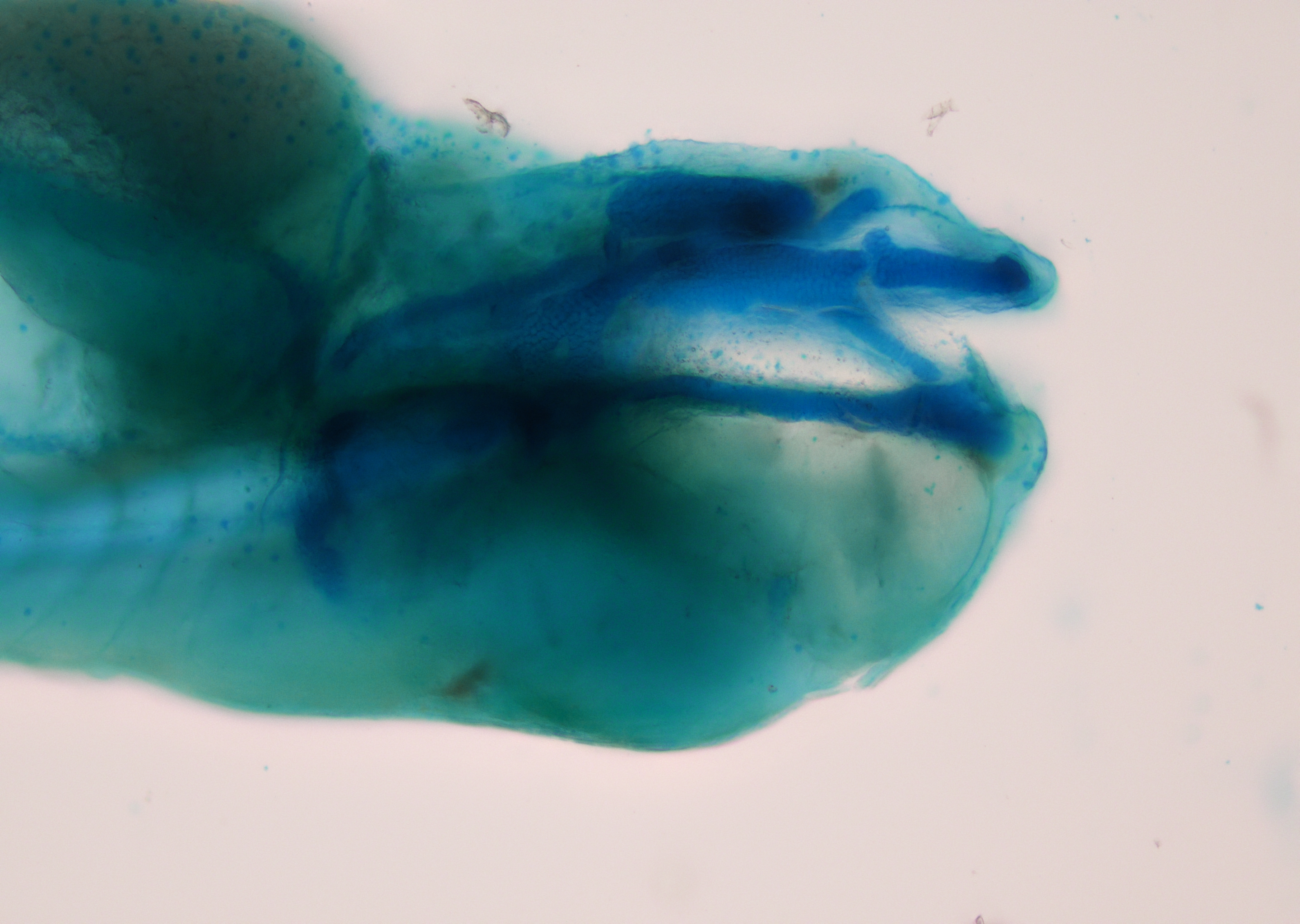

Supplement: Supplementary file 15 — Source Data for Figure 6 [file EMMM-12-e12013-s013.zip › Figure 6A/Alx1KO_uninjected_Sagittal_JPEG.jpg]

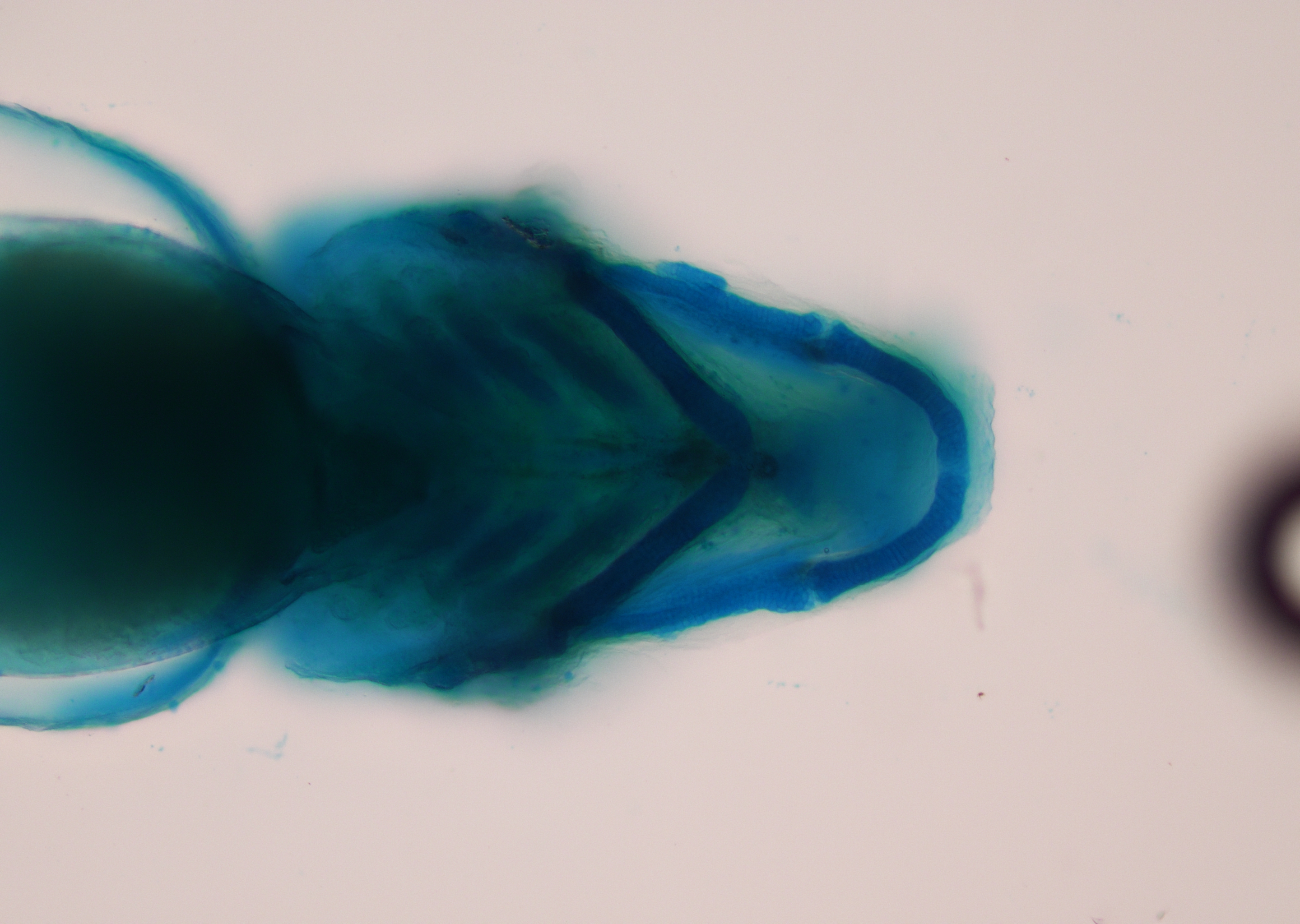

Supplement: Supplementary file 15 — Source Data for Figure 6 [file EMMM-12-e12013-s013.zip › Figure 6A/Alx1KO_uninjected_Ventral_JPEG.jpg]

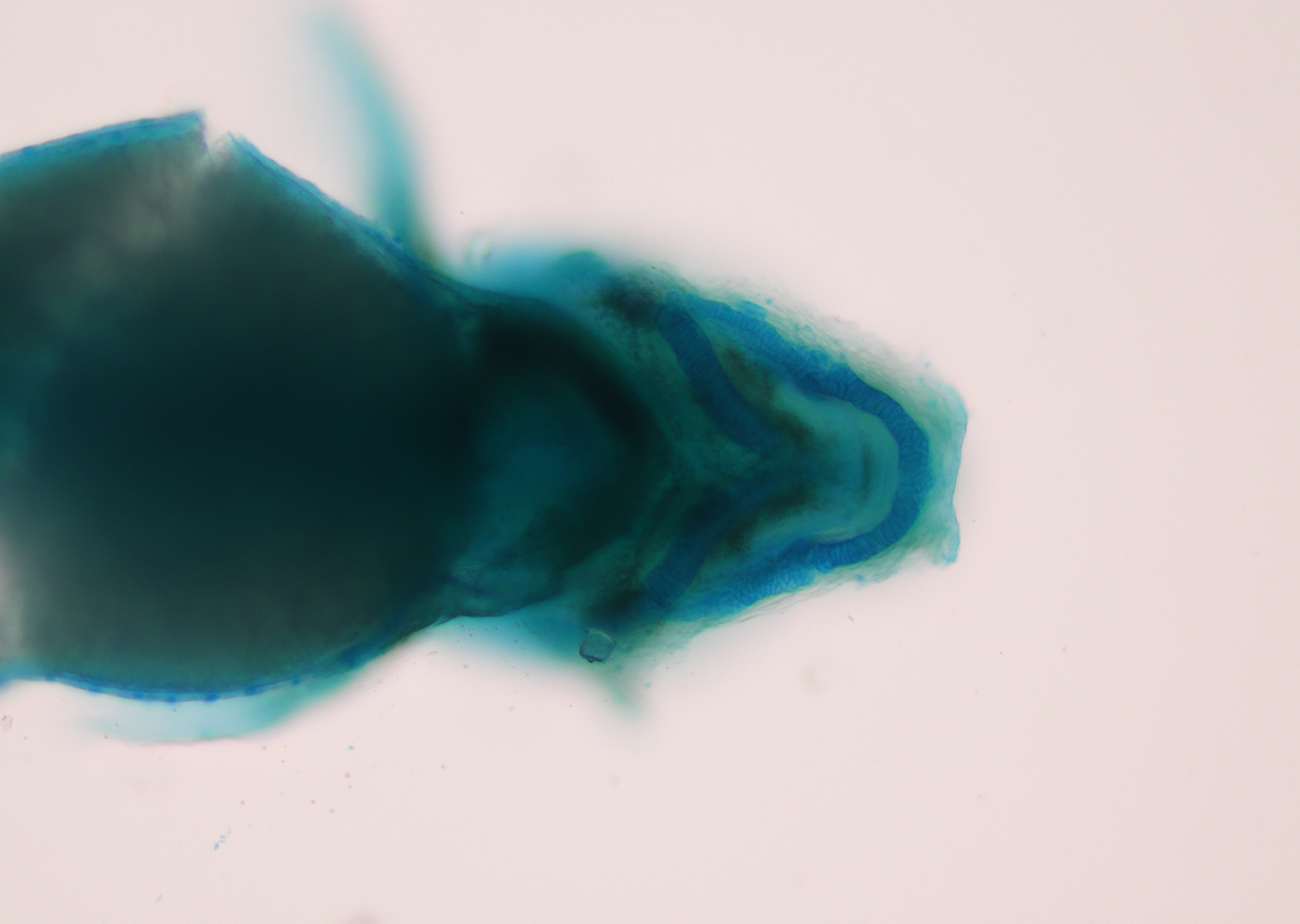

Supplement: Supplementary file 15 — Source Data for Figure 6 [file EMMM-12-e12013-s013.zip › Figure 6A/Alx1KO_Ventral_Mut_JPEG.jpg]

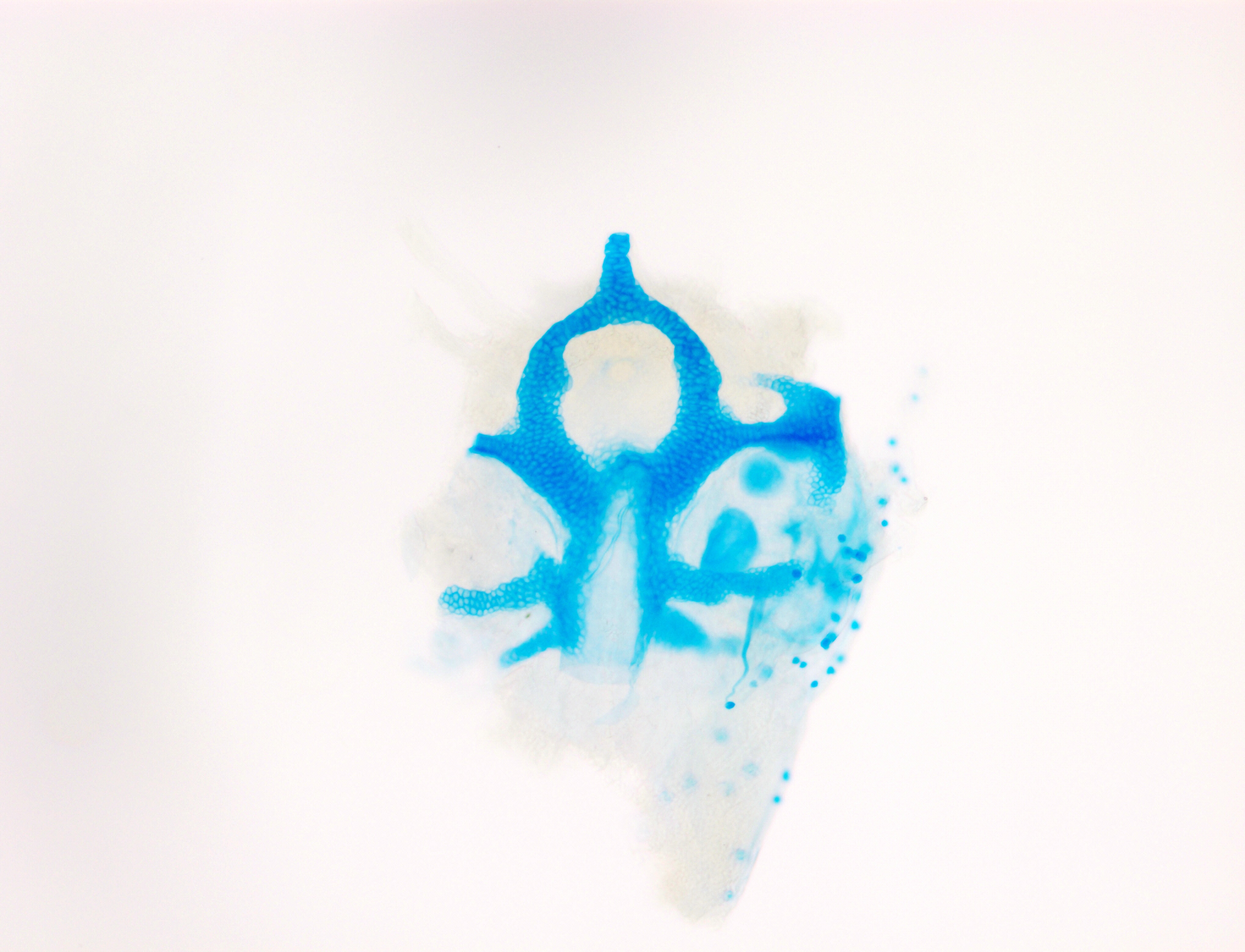

Supplement: Supplementary file 15 — Source Data for Figure 6 [file EMMM-12-e12013-s013.zip › Figure 6C/ALX1DN_ANC_JPEG.jpg]

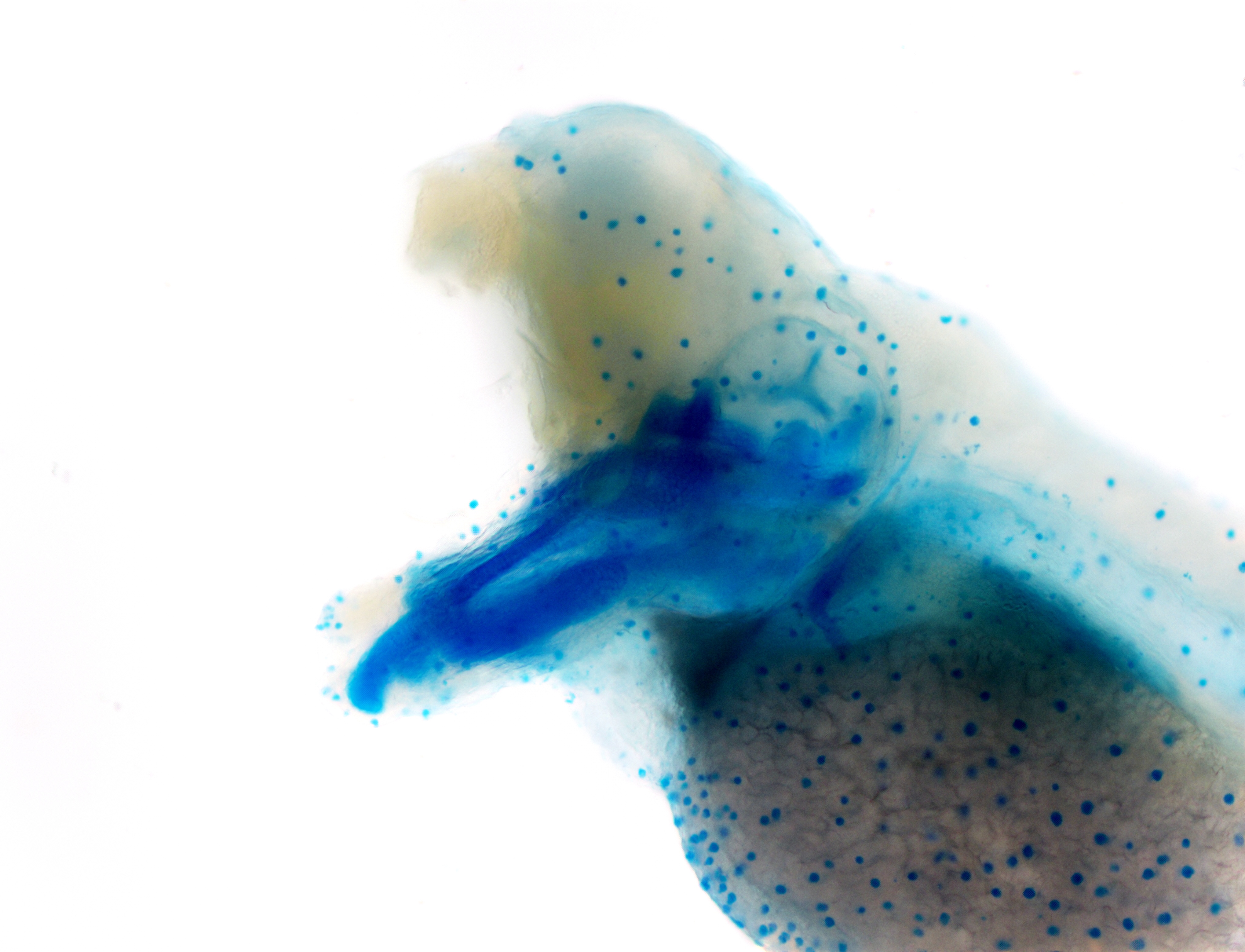

Supplement: Supplementary file 15 — Source Data for Figure 6 [file EMMM-12-e12013-s013.zip › Figure 6C/ALX1DN_lateral_JPEG.jpg]

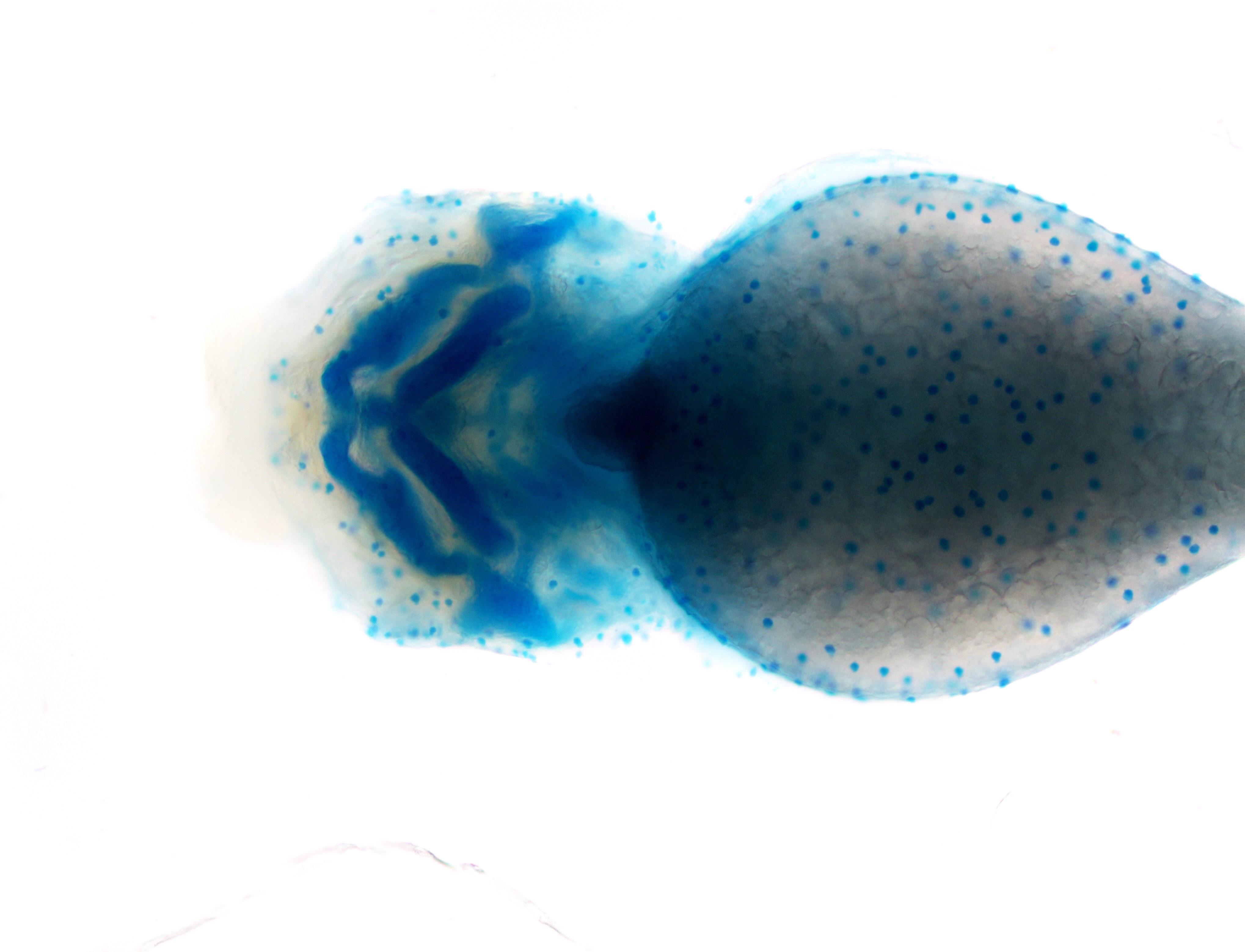

Supplement: Supplementary file 15 — Source Data for Figure 6 [file EMMM-12-e12013-s013.zip › Figure 6C/ALX1DN_ventral_1_JPEG.jpg]

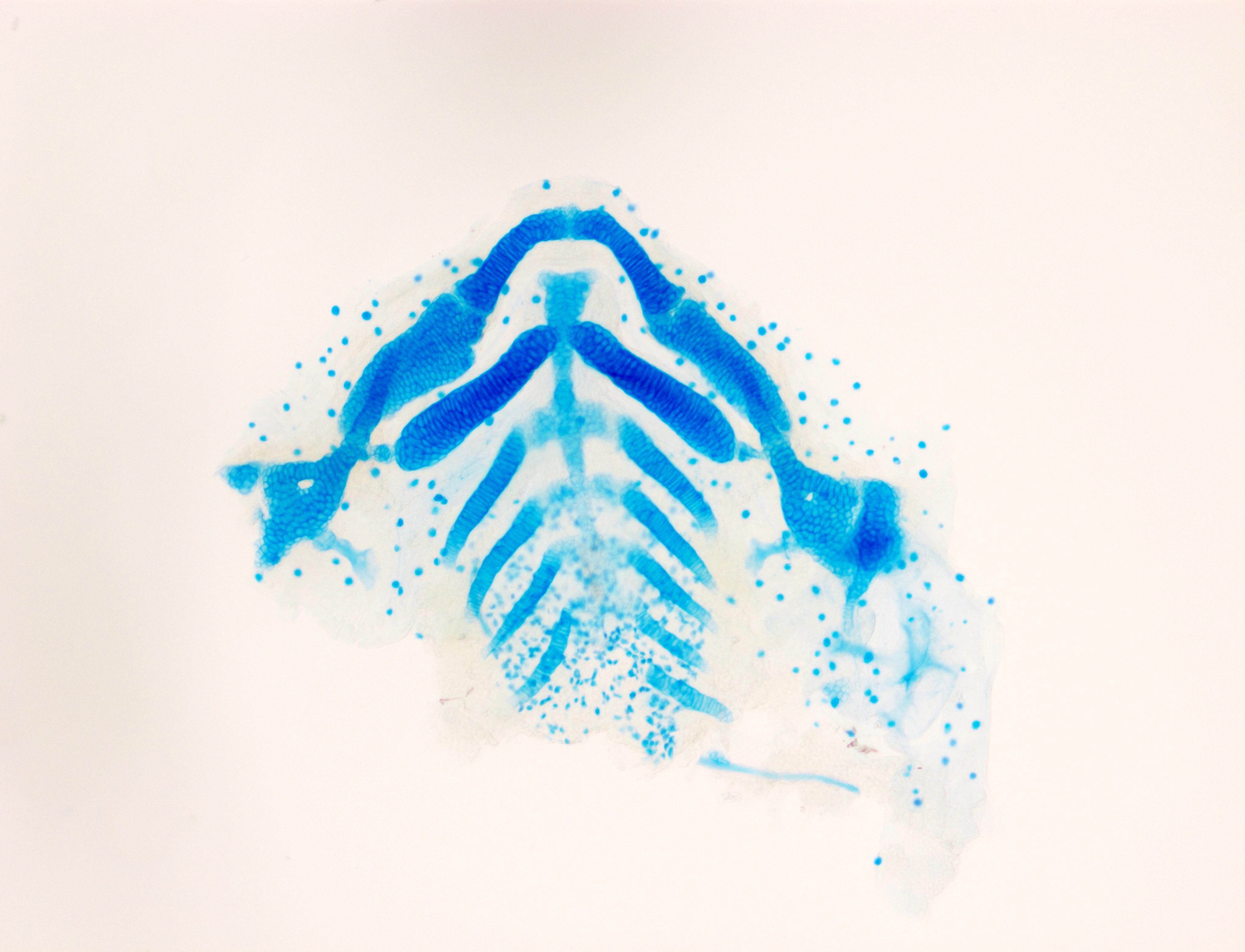

Supplement: Supplementary file 15 — Source Data for Figure 6 [file EMMM-12-e12013-s013.zip › Figure 6C/ALX1DN_ventral_2_JPEG.jpg]

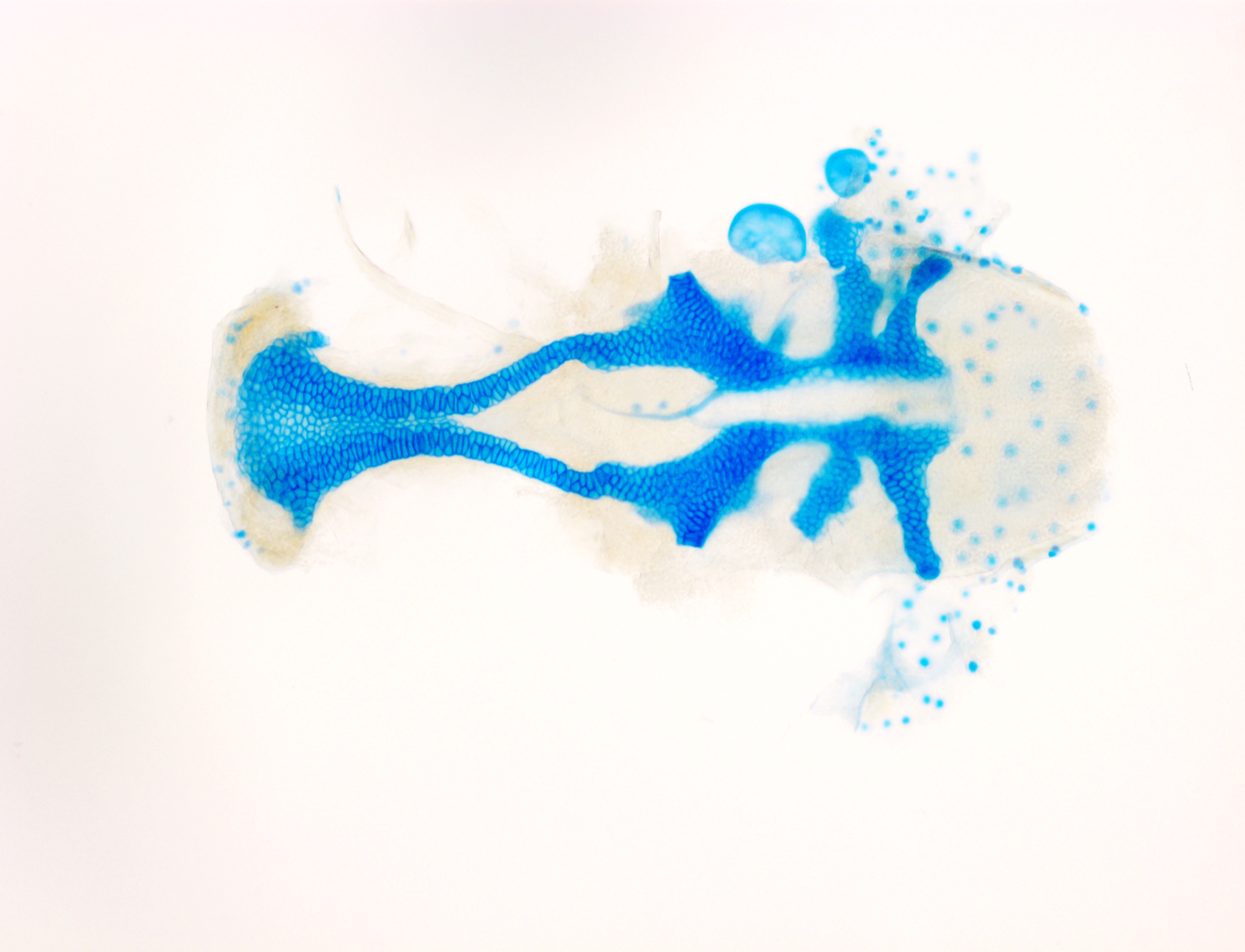

Supplement: Supplementary file 15 — Source Data for Figure 6 [file EMMM-12-e12013-s013.zip › Figure 6C/Control_ANC_JPEG.jpg]

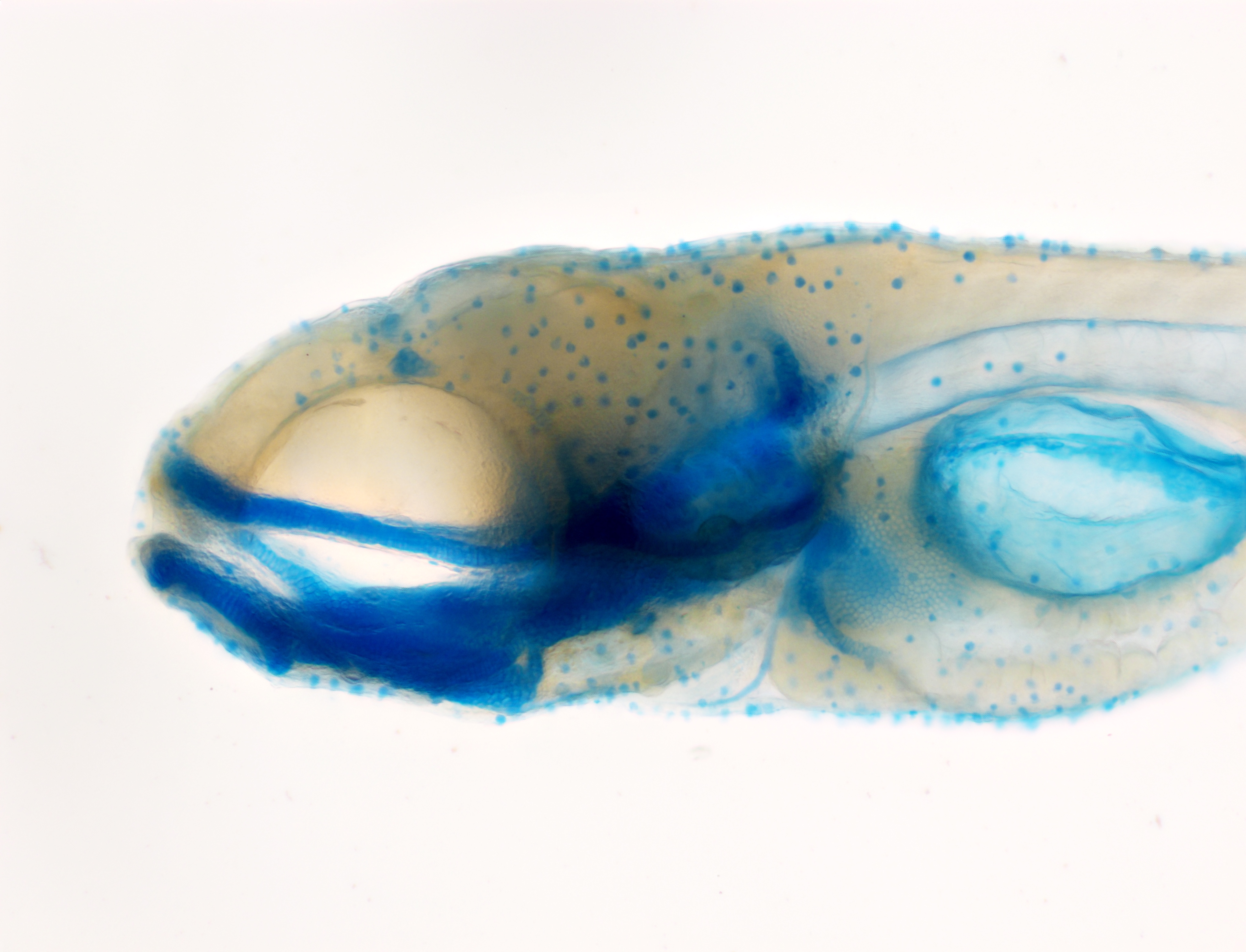

Supplement: Supplementary file 15 — Source Data for Figure 6 [file EMMM-12-e12013-s013.zip › Figure 6C/Control_lateral_JPEG.jpg]

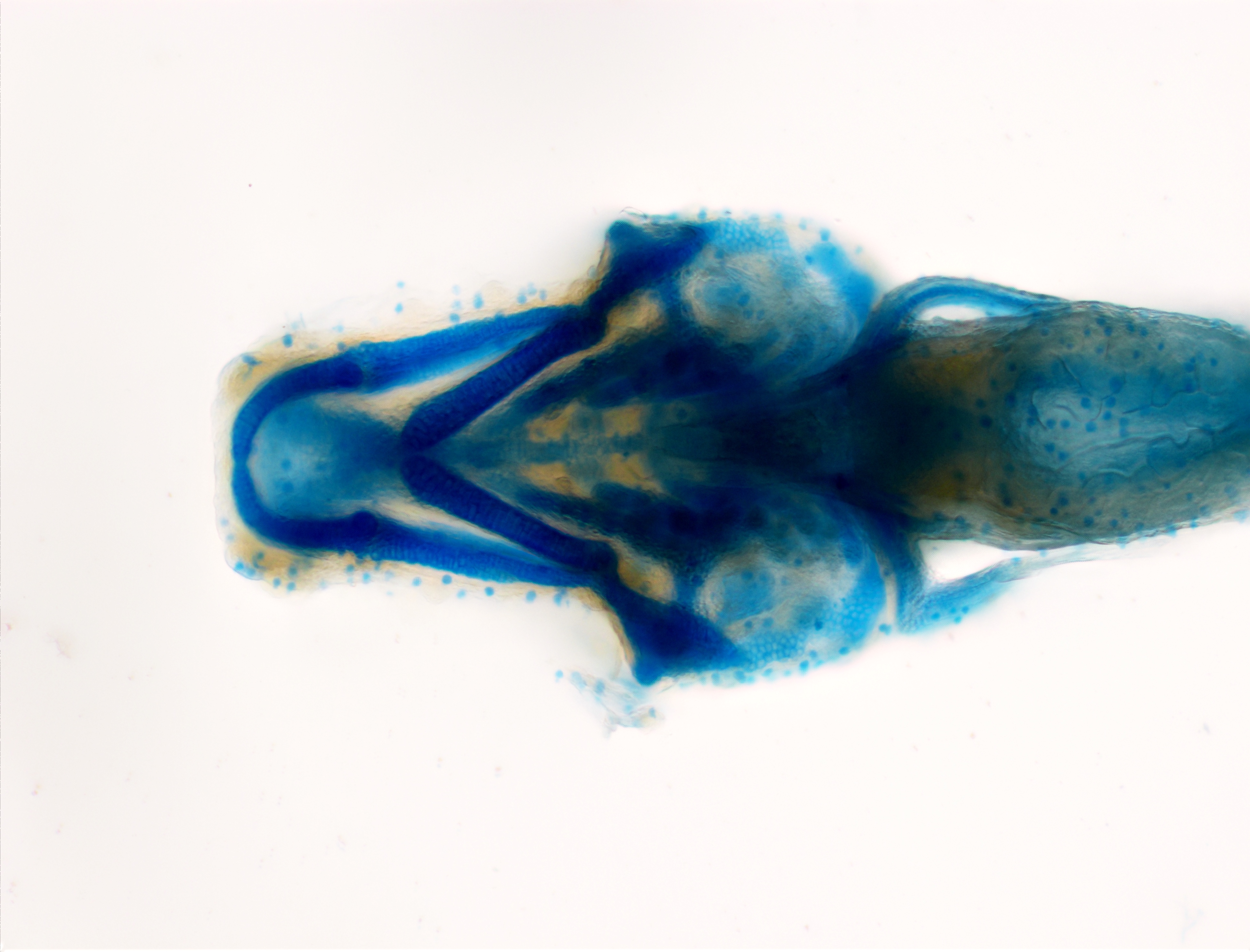

Supplement: Supplementary file 15 — Source Data for Figure 6 [file EMMM-12-e12013-s013.zip › Figure 6C/Control_ventral_1_JPEG.jpg]

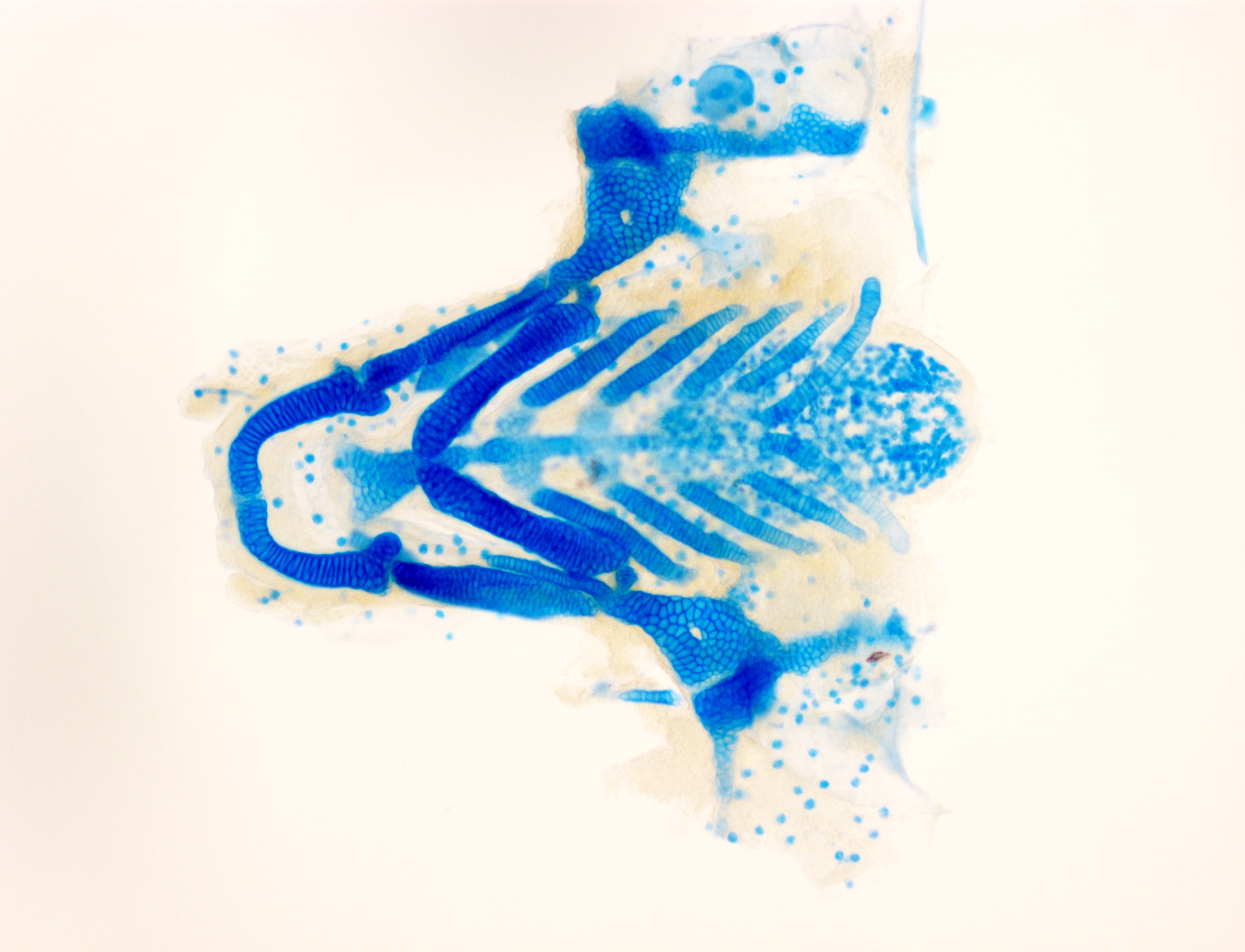

Supplement: Supplementary file 15 — Source Data for Figure 6 [file EMMM-12-e12013-s013.zip › Figure 6C/Control_ventral_2_JPEG.jpg]

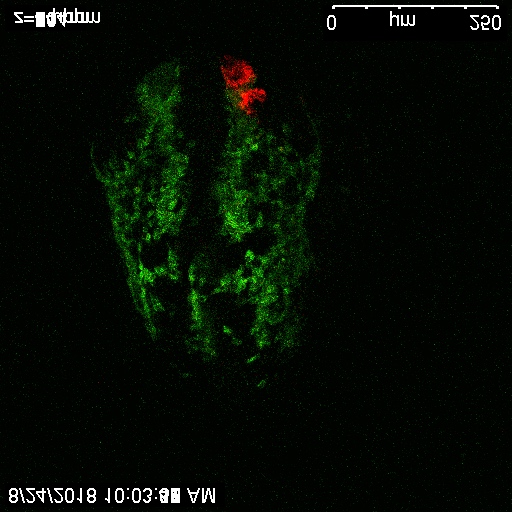

Supplement: Supplementary file 15 — Source Data for Figure 6 [file EMMM-12-e12013-s013.zip › Figure 6D/Alx1DN_10_somites_JPEG.jpg]

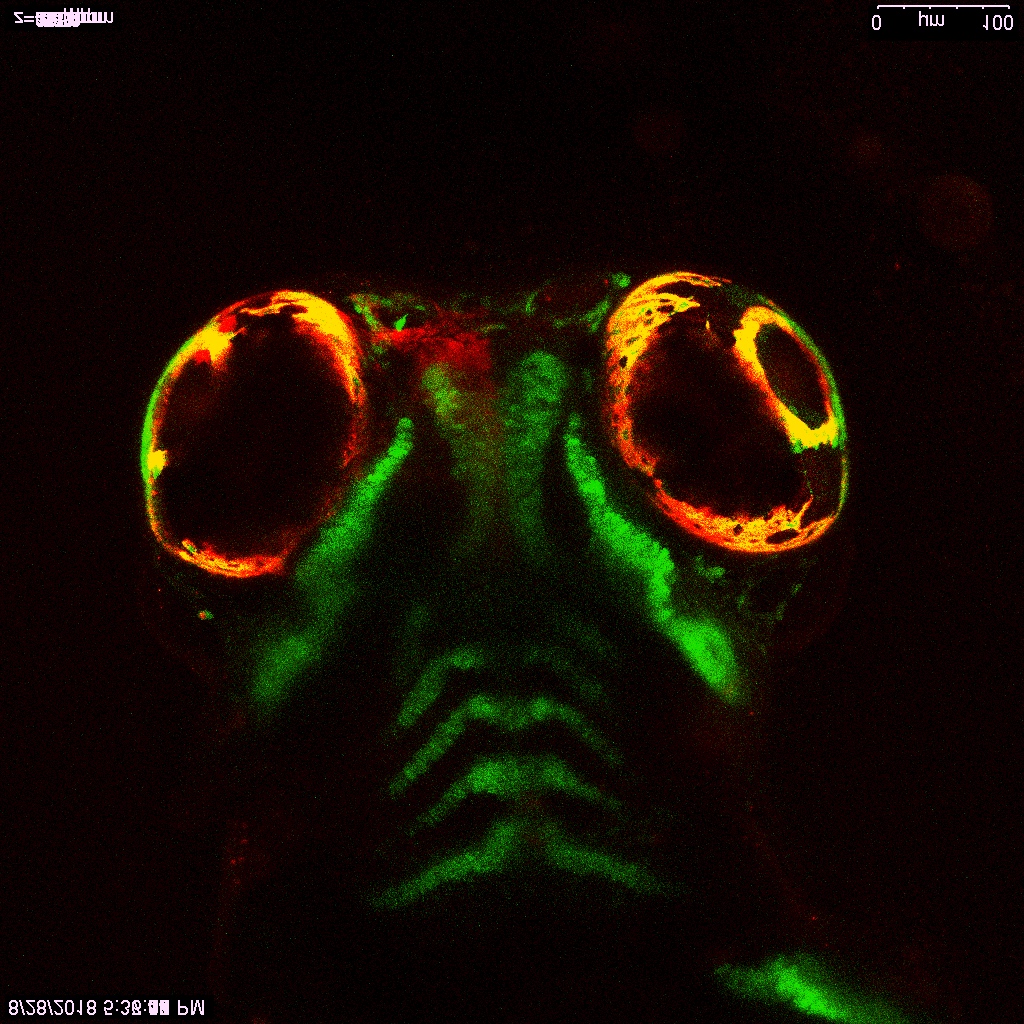

Supplement: Supplementary file 15 — Source Data for Figure 6 [file EMMM-12-e12013-s013.zip › Figure 6D/Alx1DN_4_dpf_JPEG.jpg]

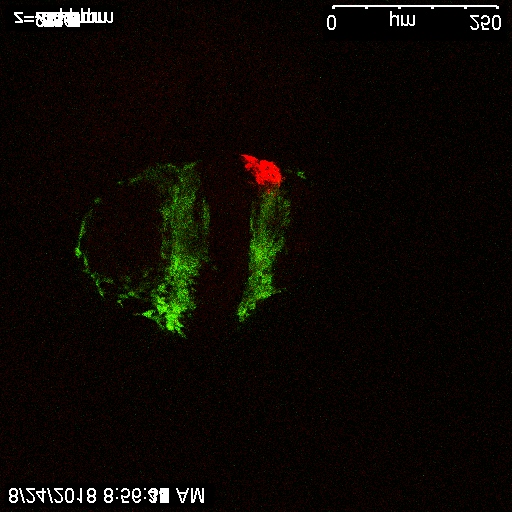

Supplement: Supplementary file 15 — Source Data for Figure 6 [file EMMM-12-e12013-s013.zip › Figure 6D/Control_10_somites_JPEG.jpg]

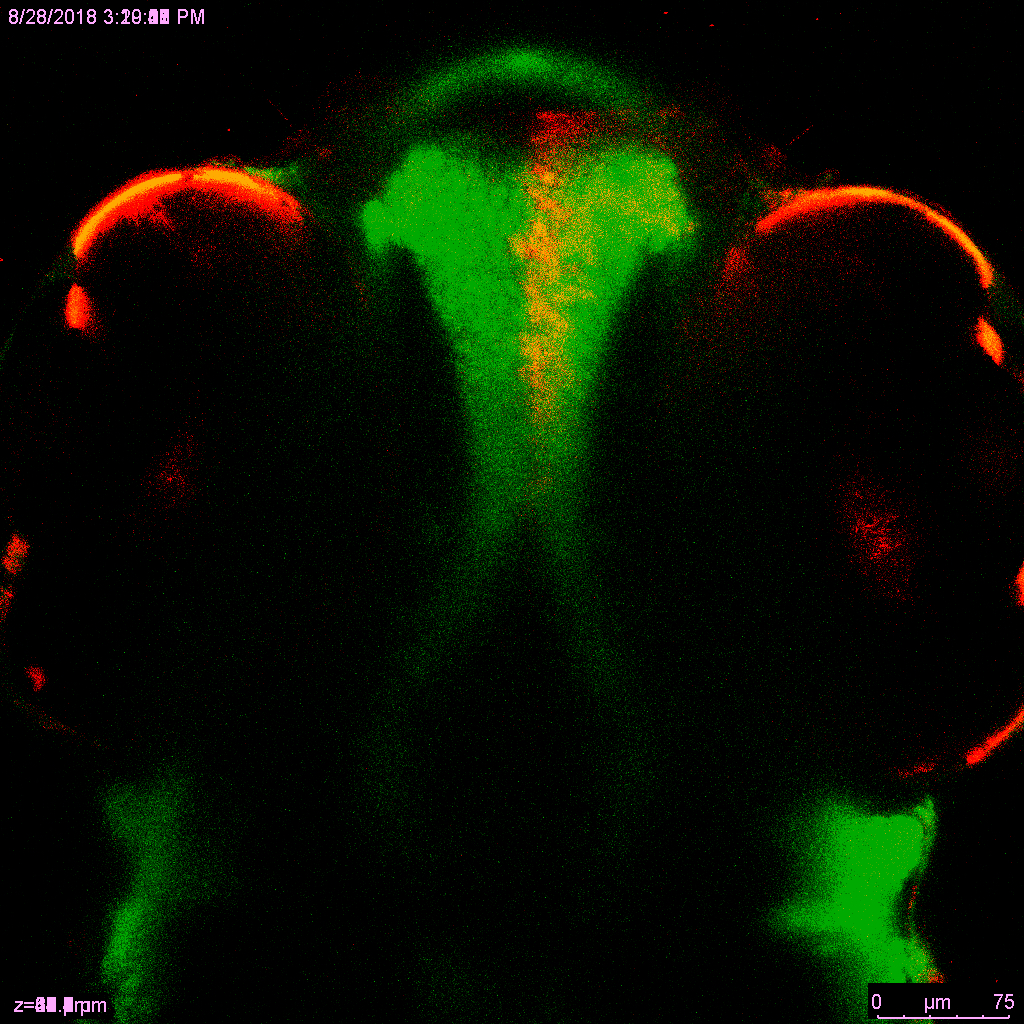

Supplement: Supplementary file 15 — Source Data for Figure 6 [file EMMM-12-e12013-s013.zip › Figure 6D/Control_4_dpf_JPEG.jpg]
