# Supplementary material for: ALX1‐related frontonasal dysplasia results from defective neural crest cell development and migration
Source: EMBO Mol Med. 2020 Sep 11;12(10):e12013. doi: 10.15252/emmm.202012013 (PMC7539331; doi:10.15252/emmm.202012013)

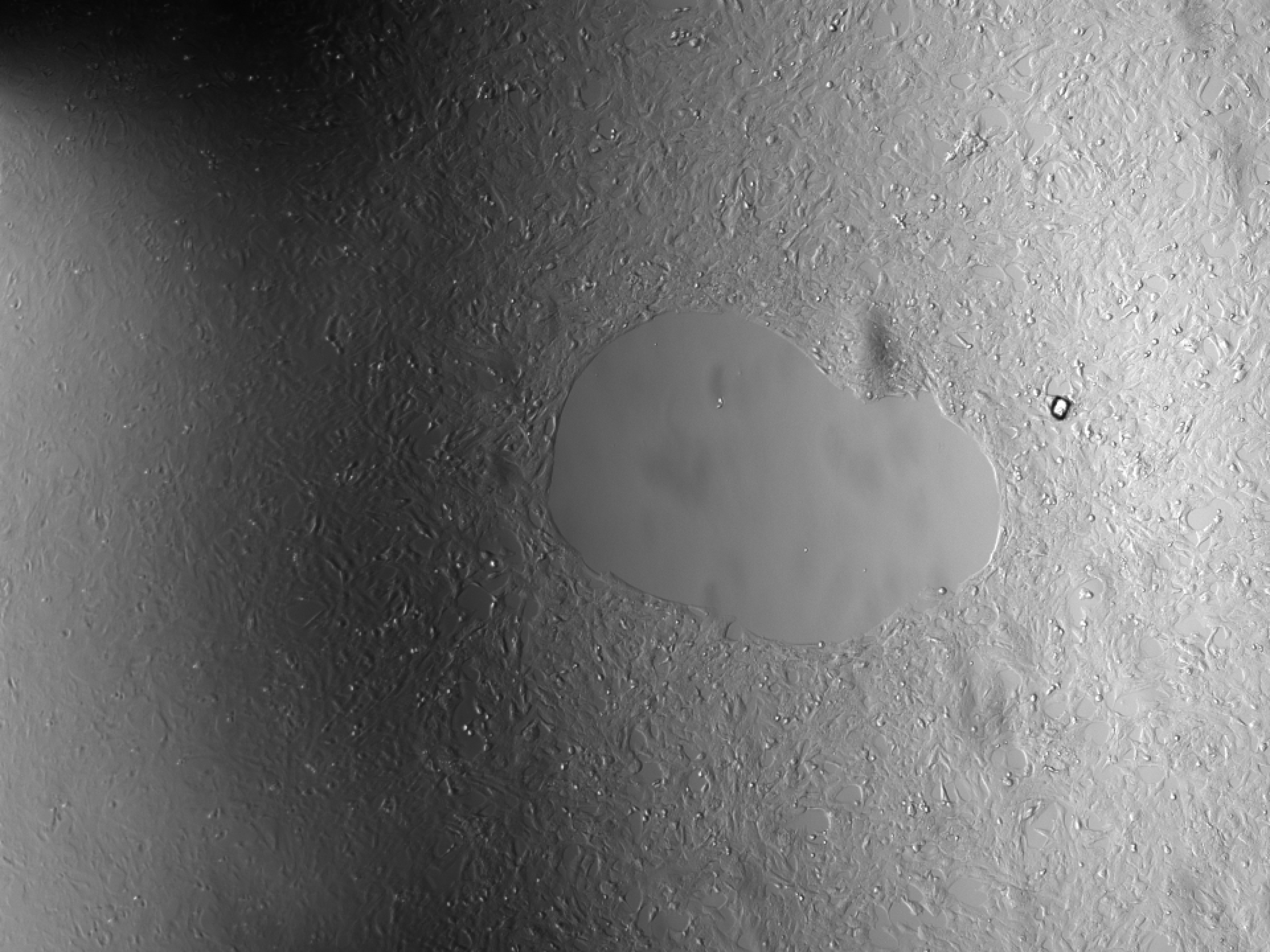

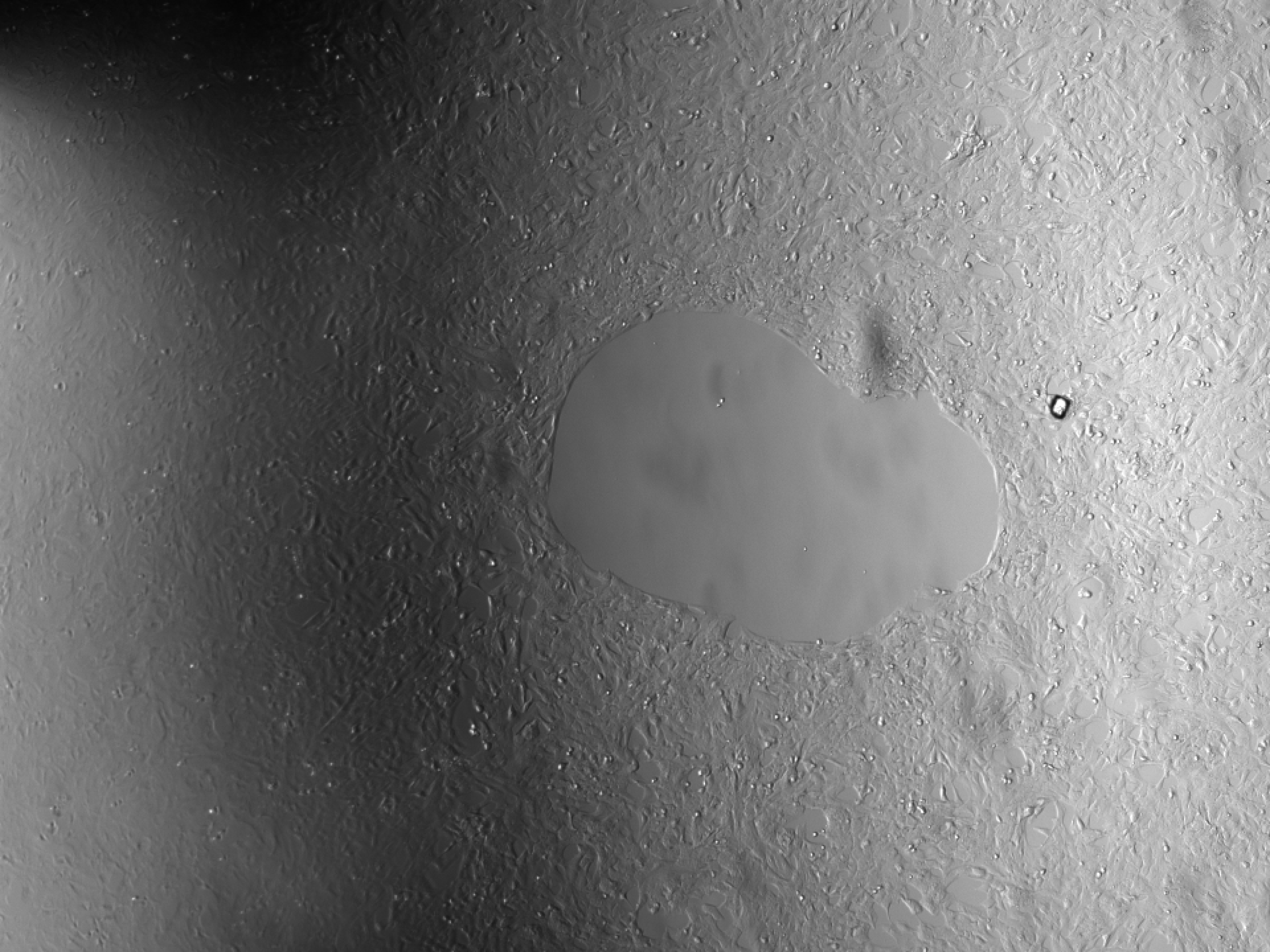

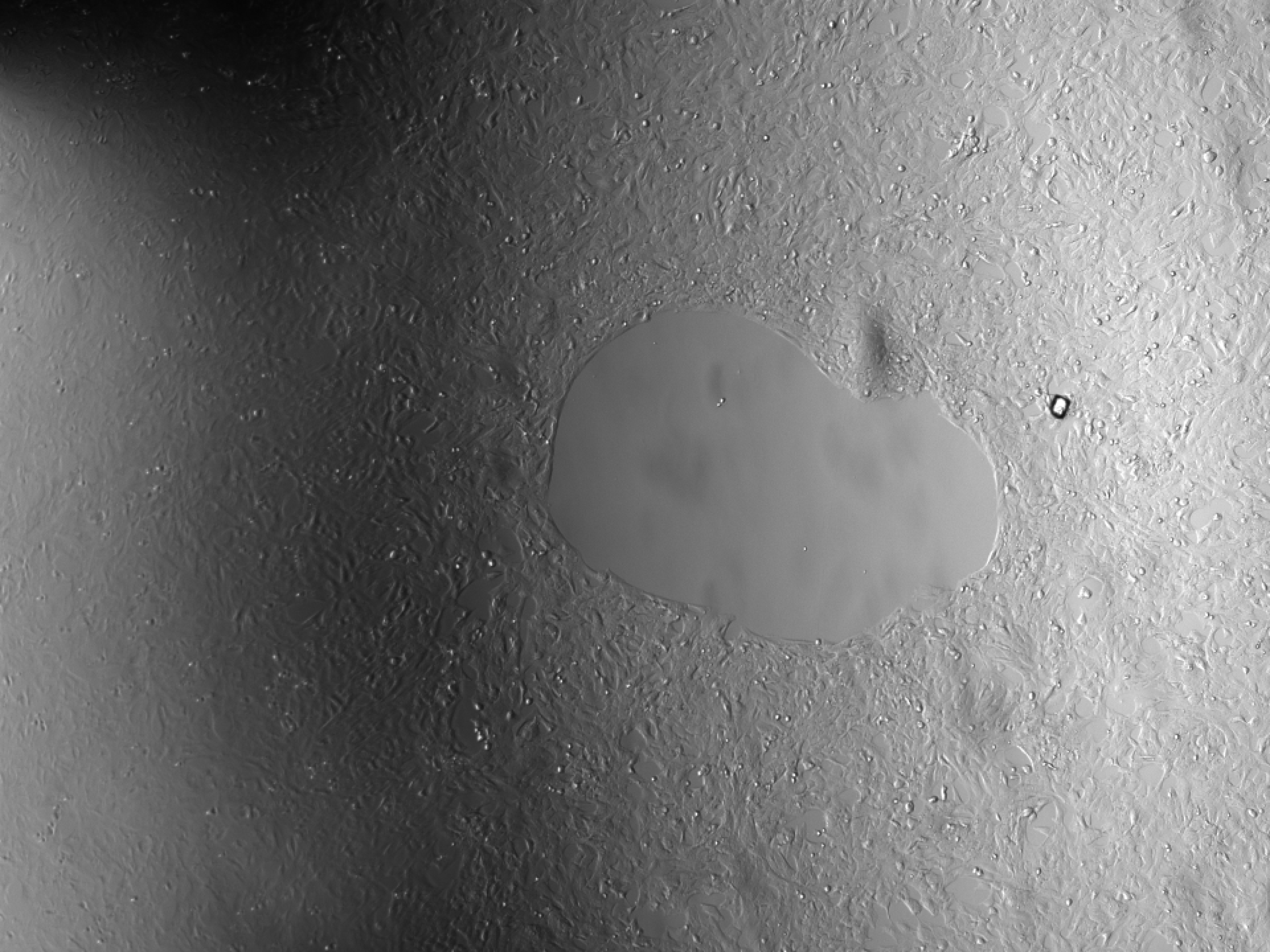

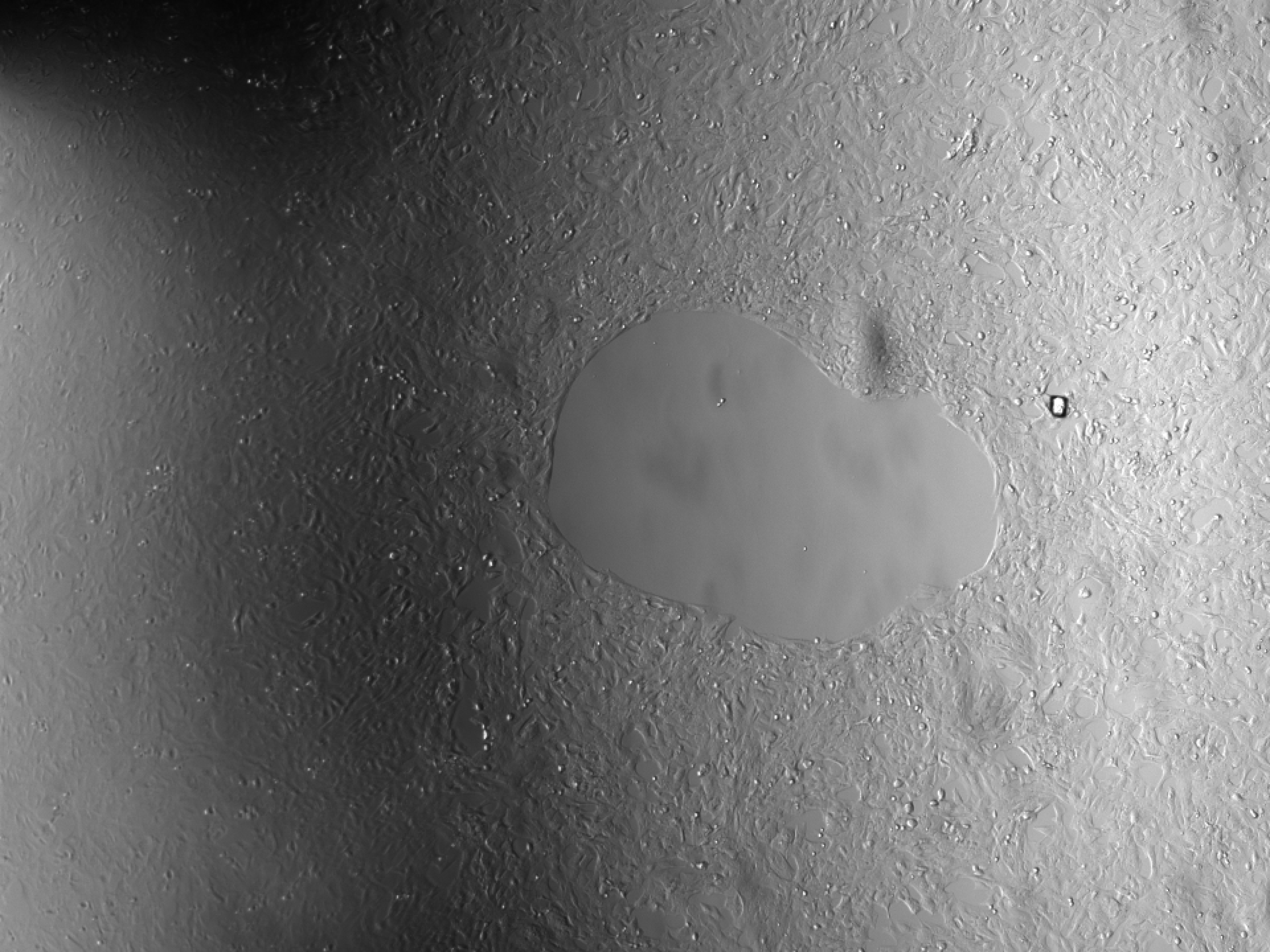

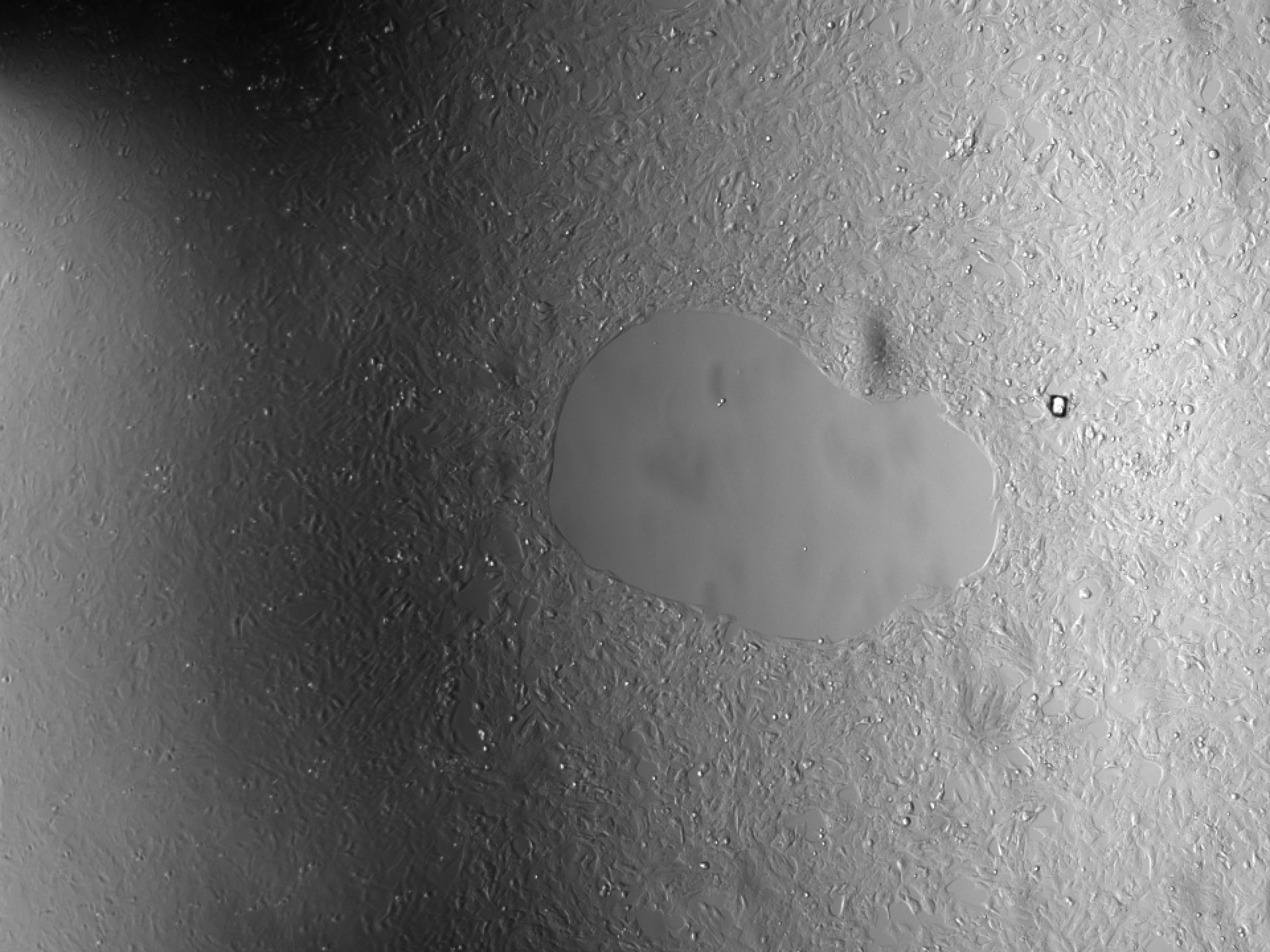

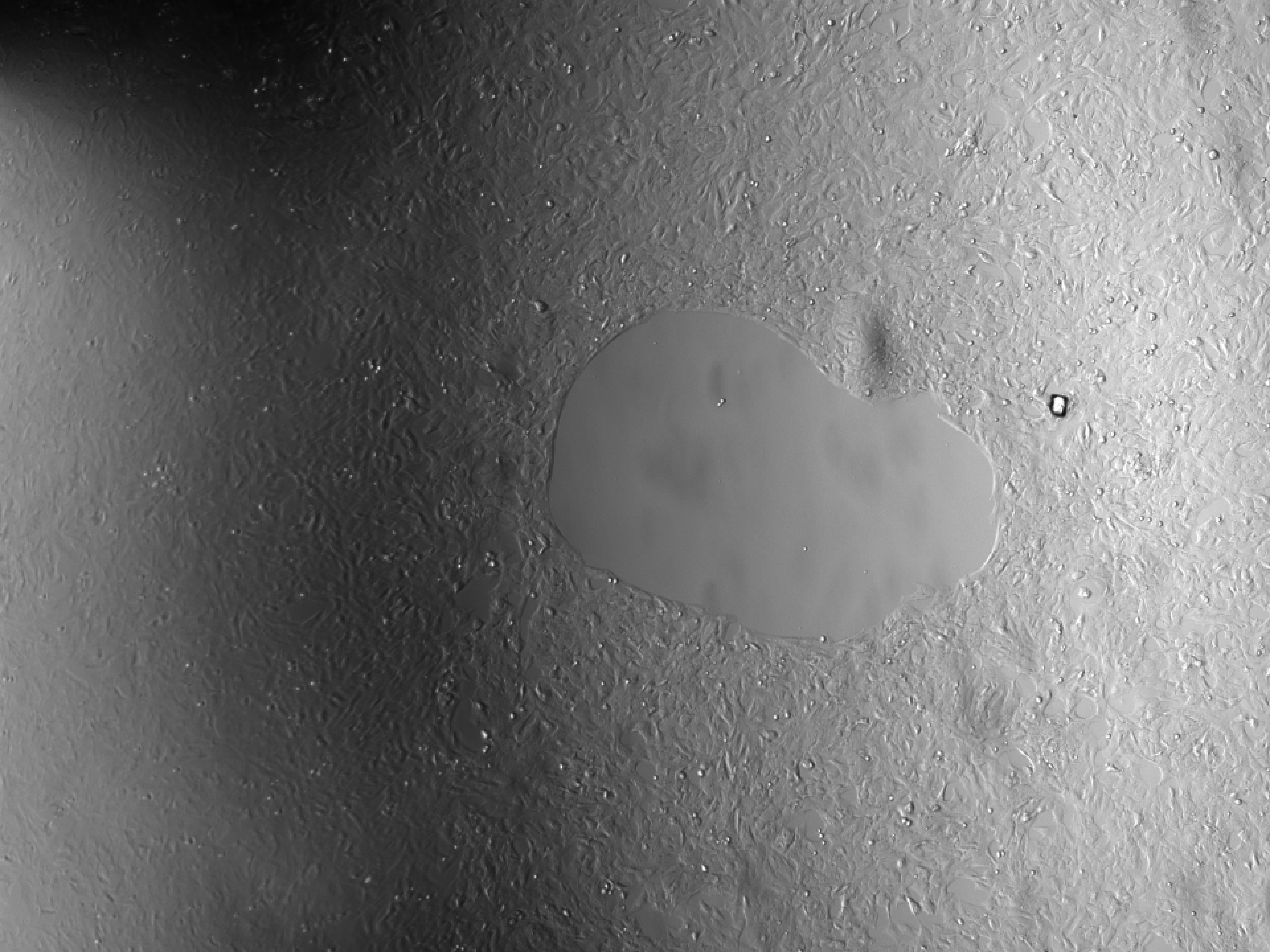

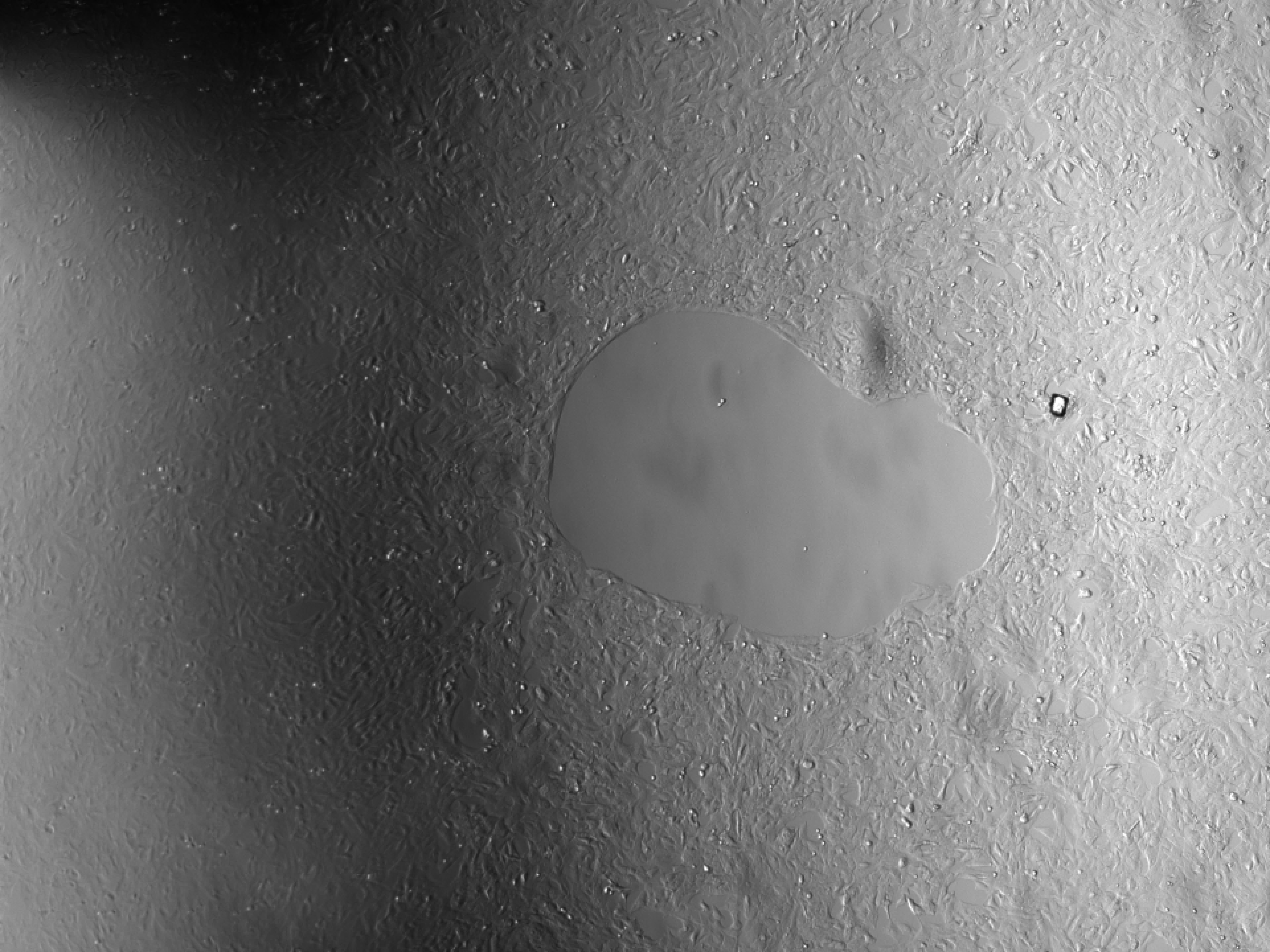

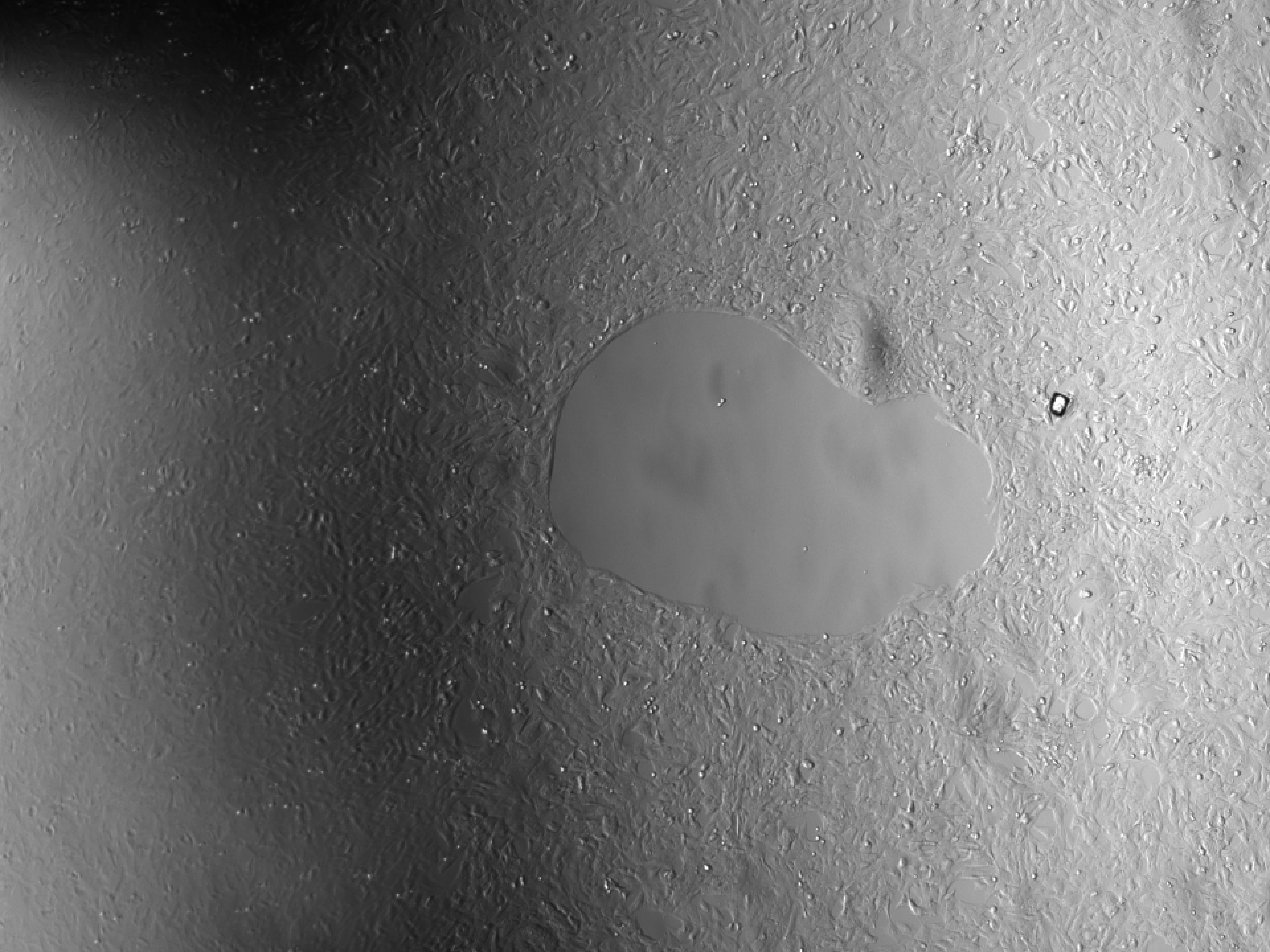

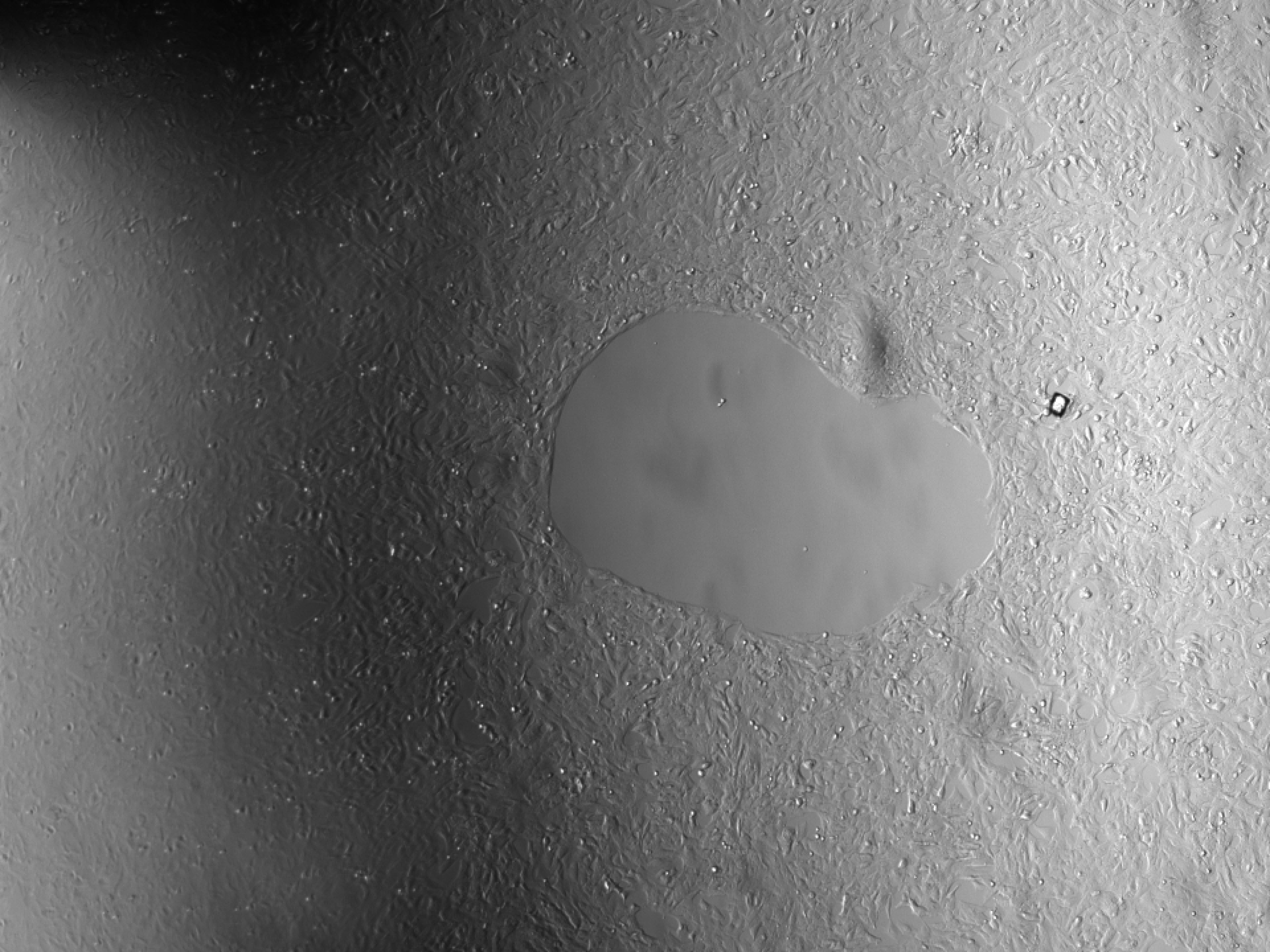

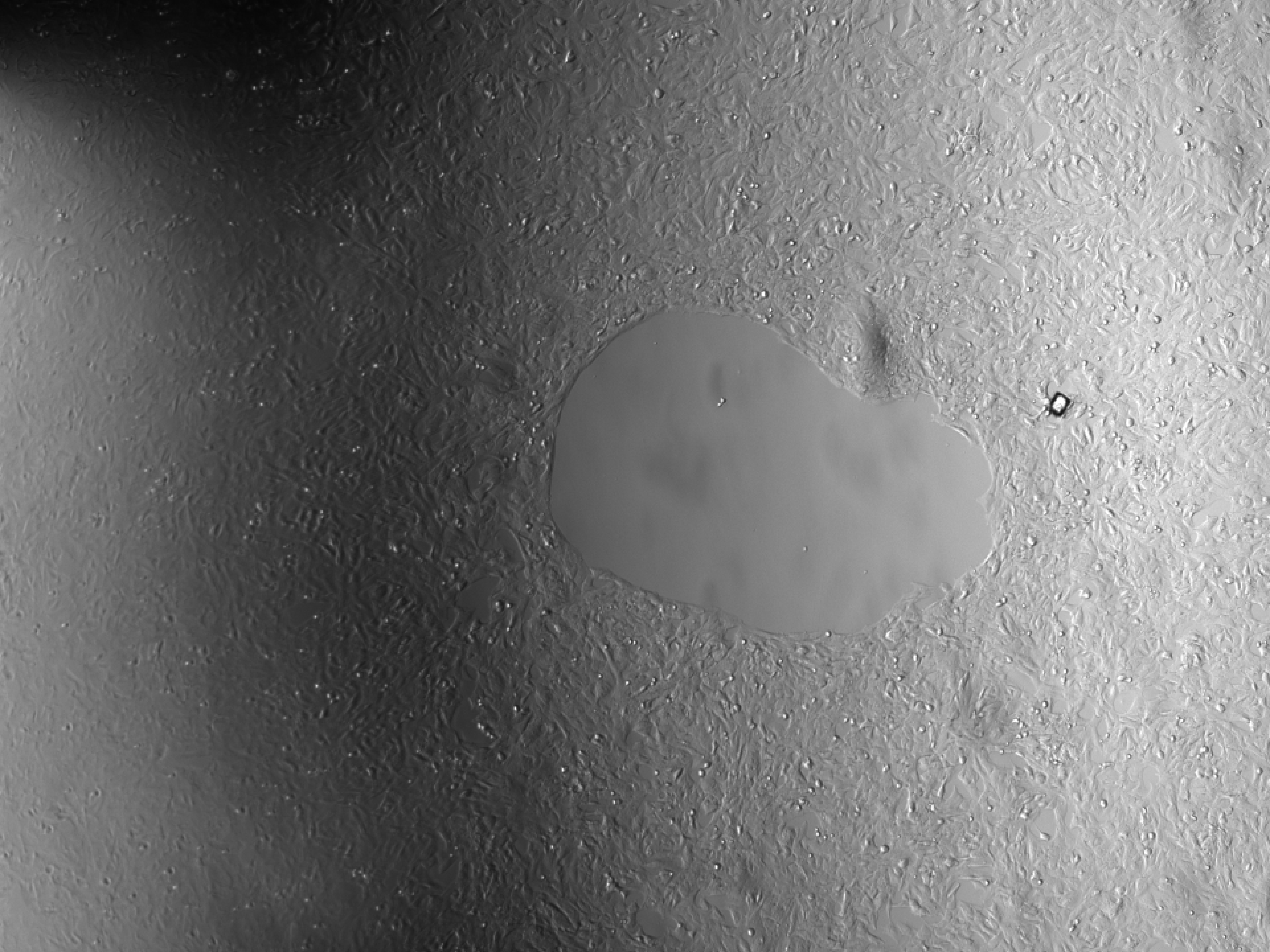

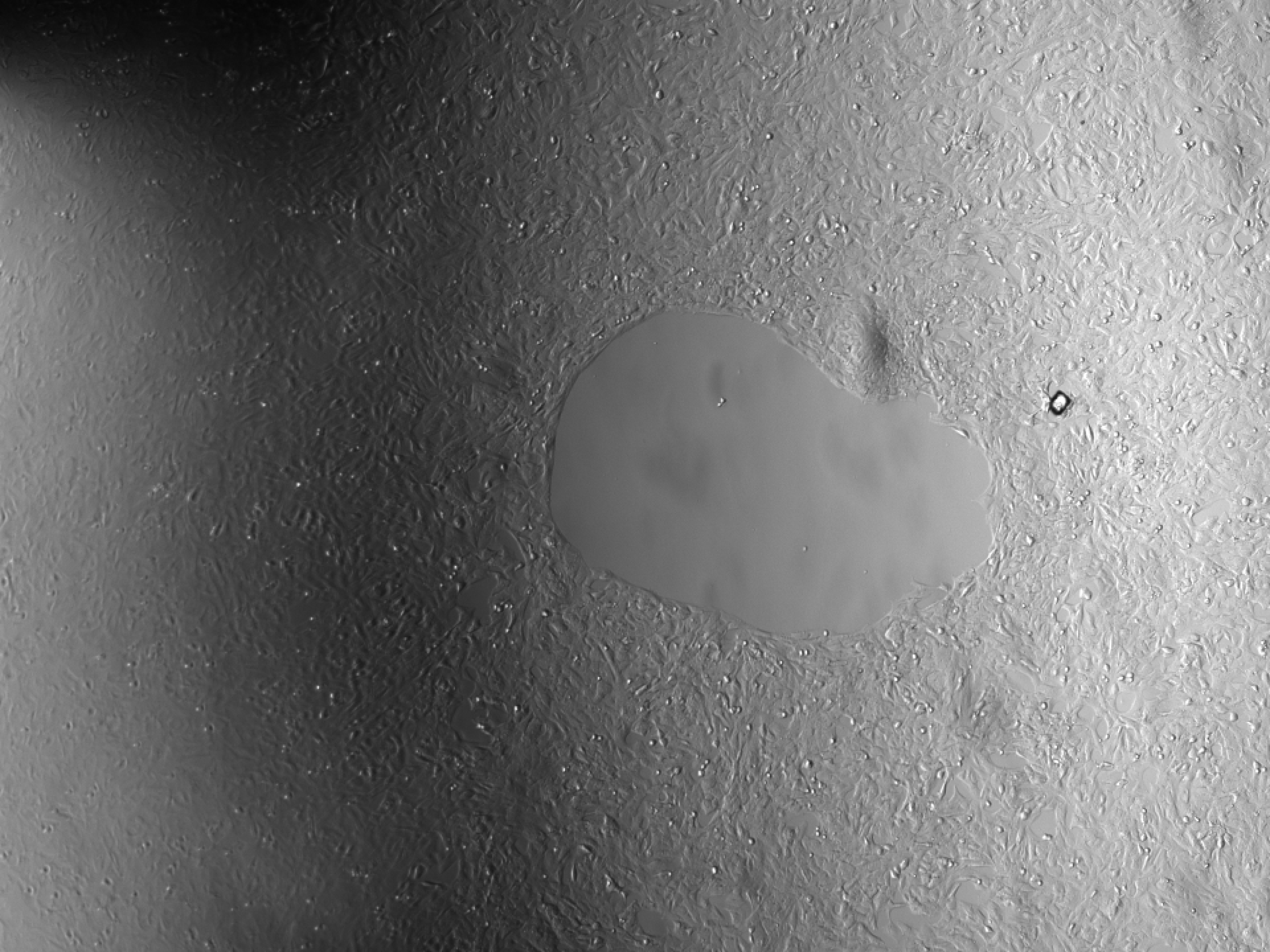

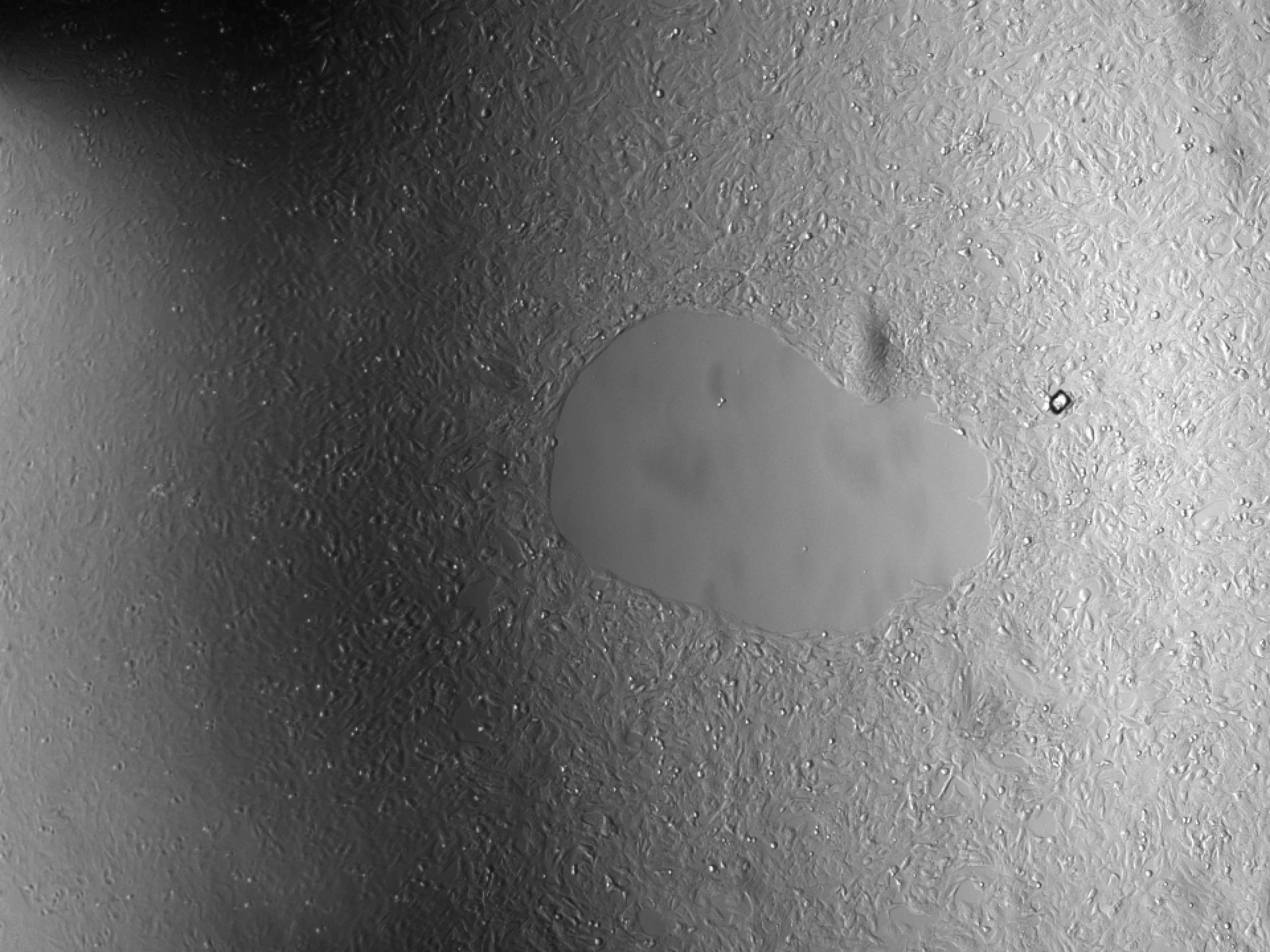

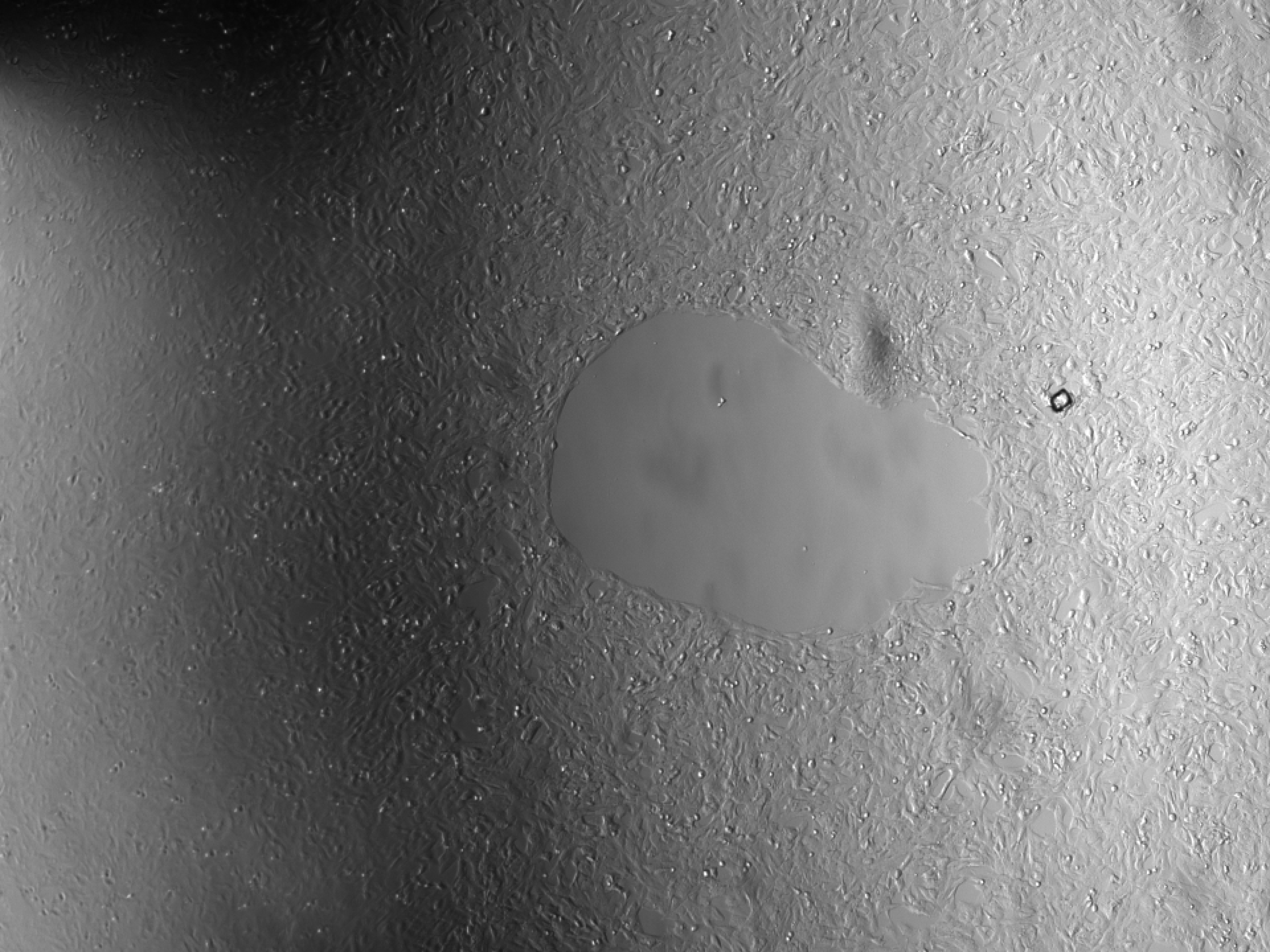

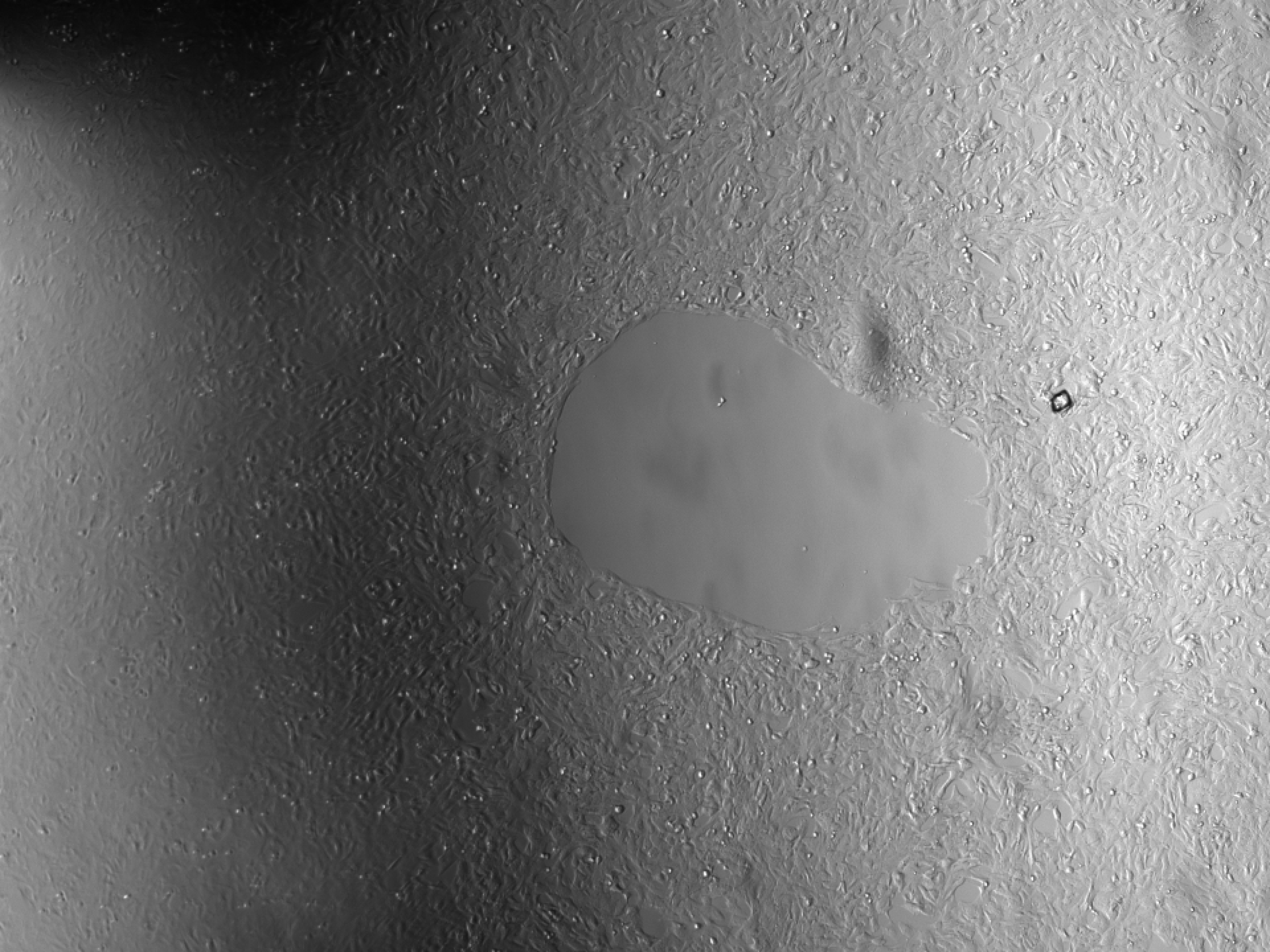

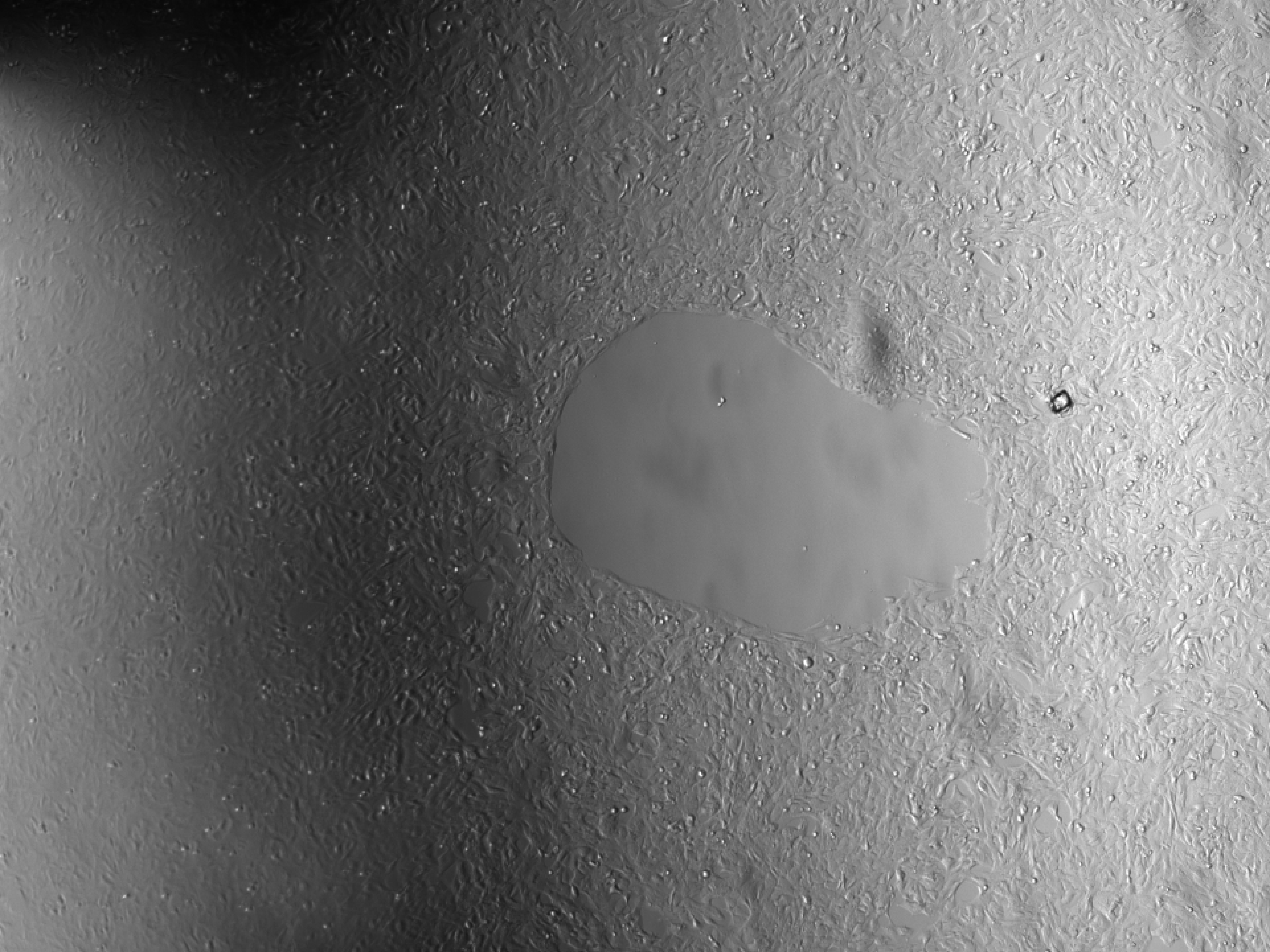

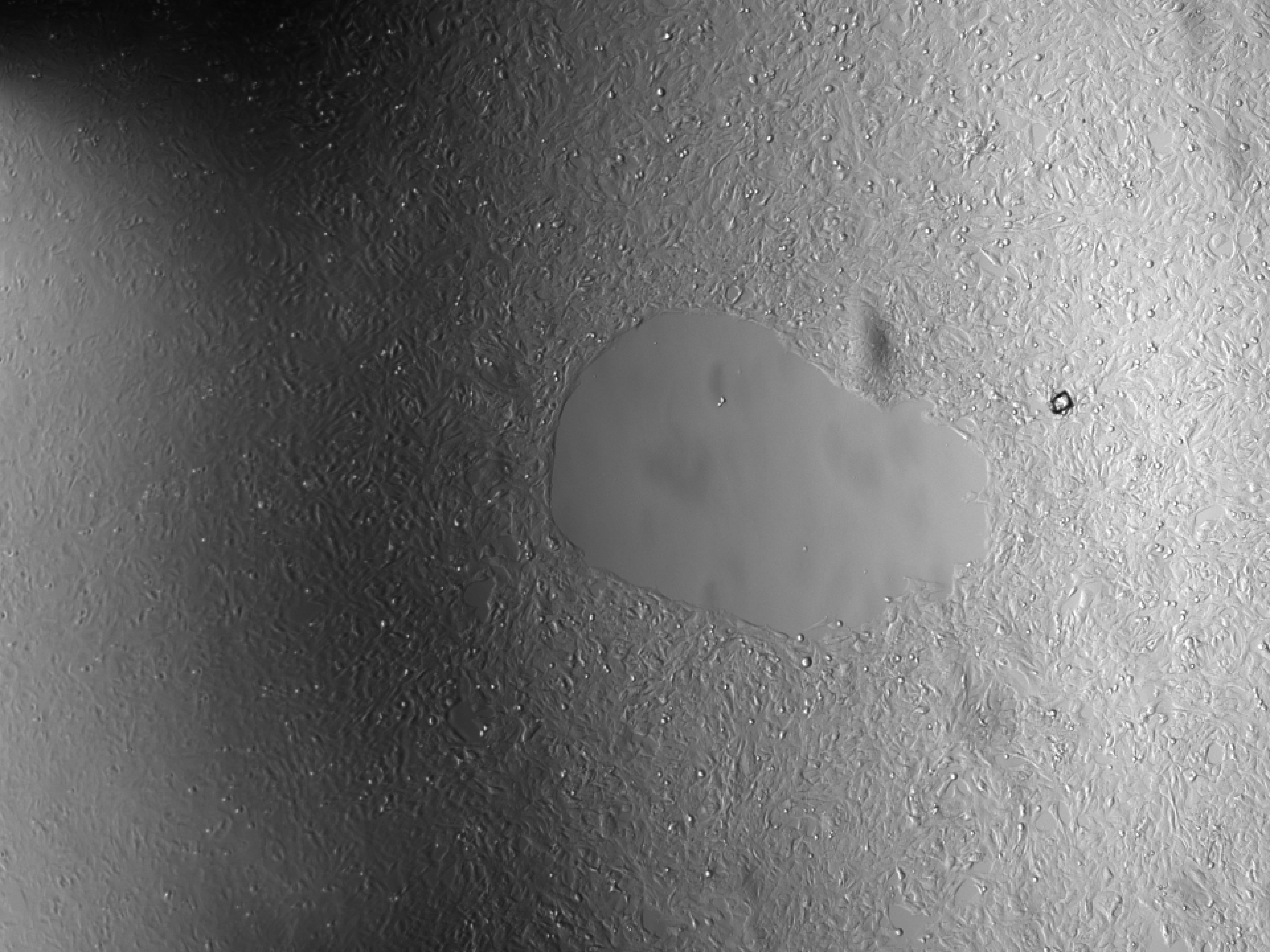

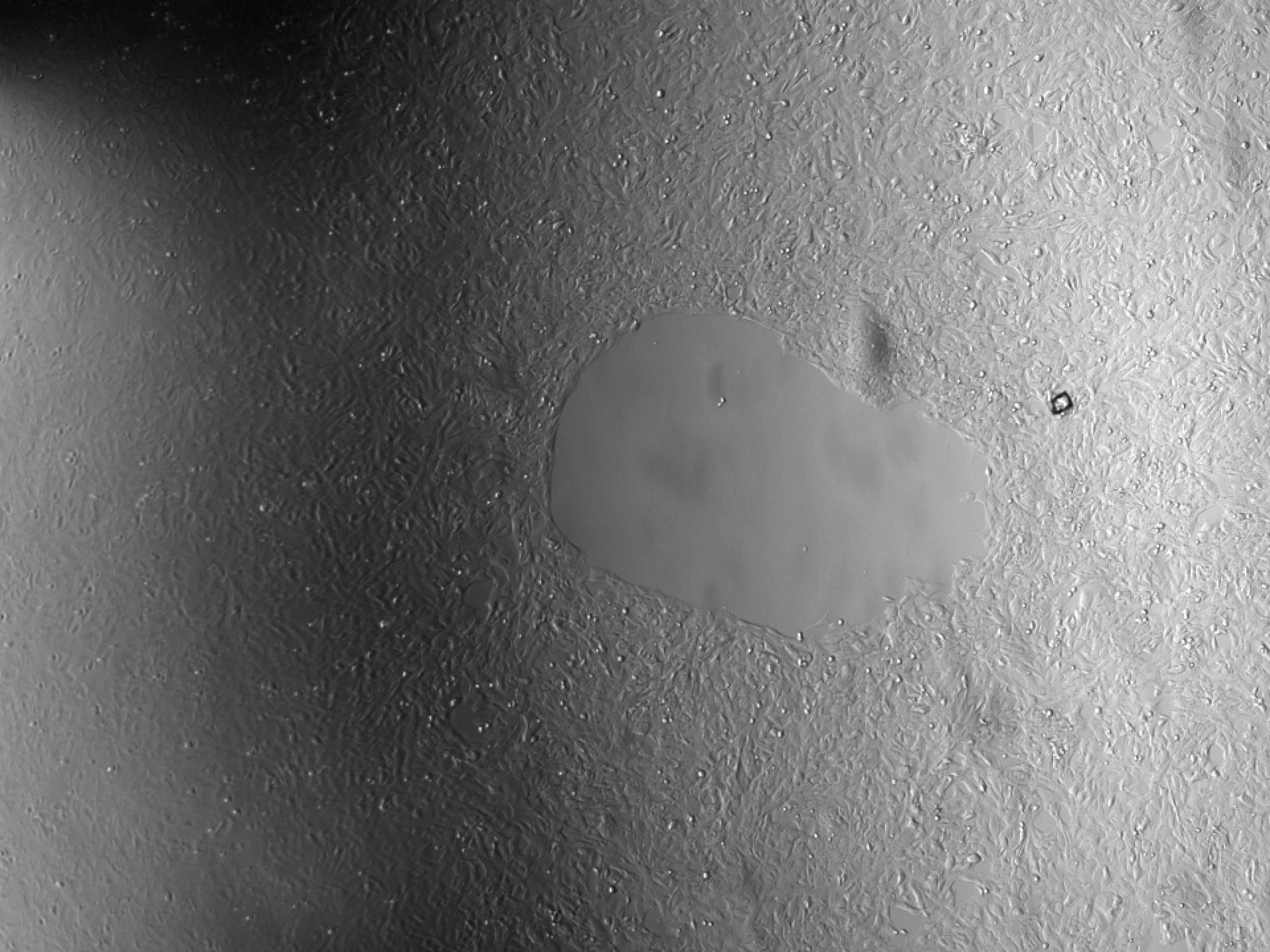

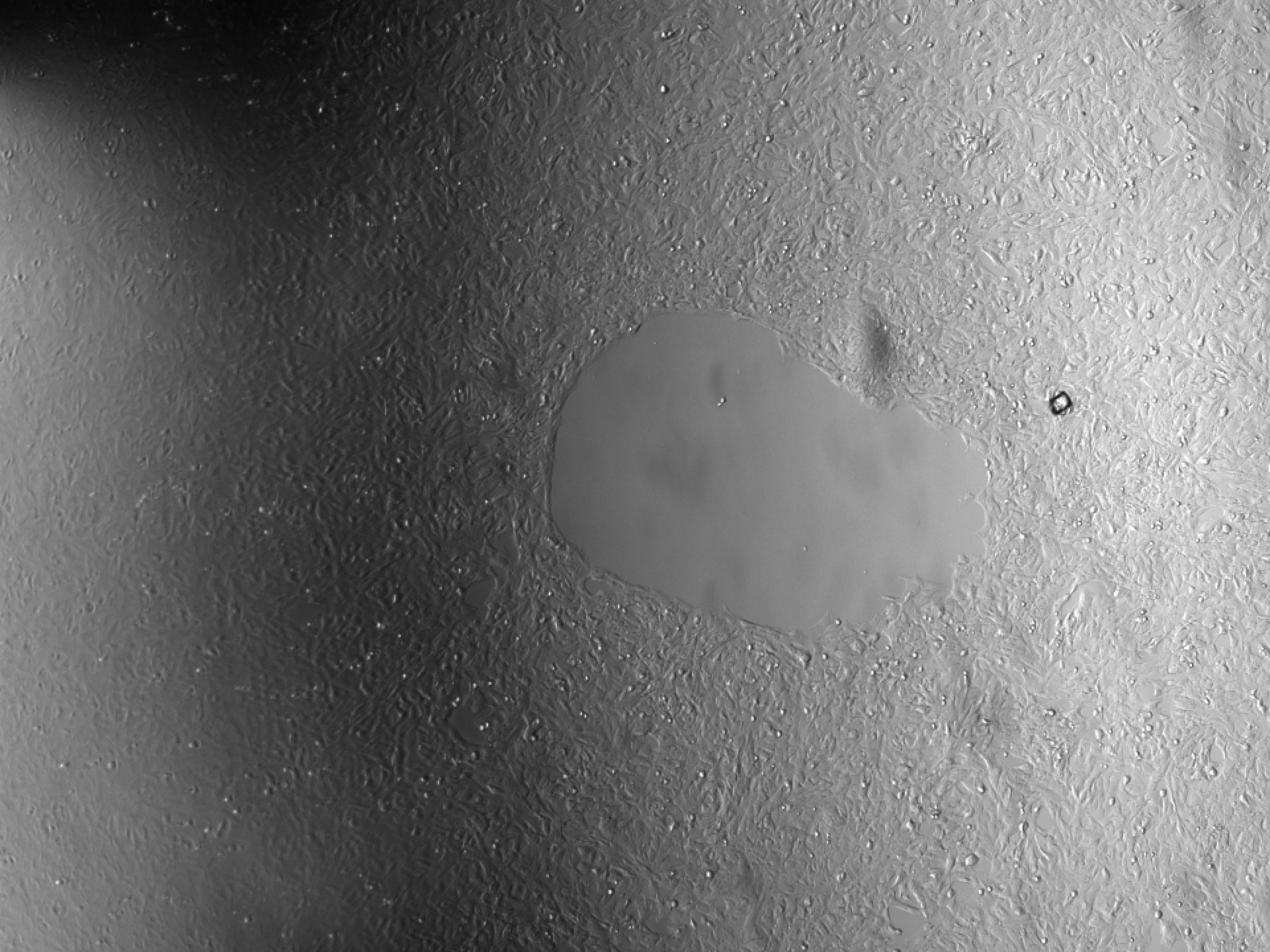

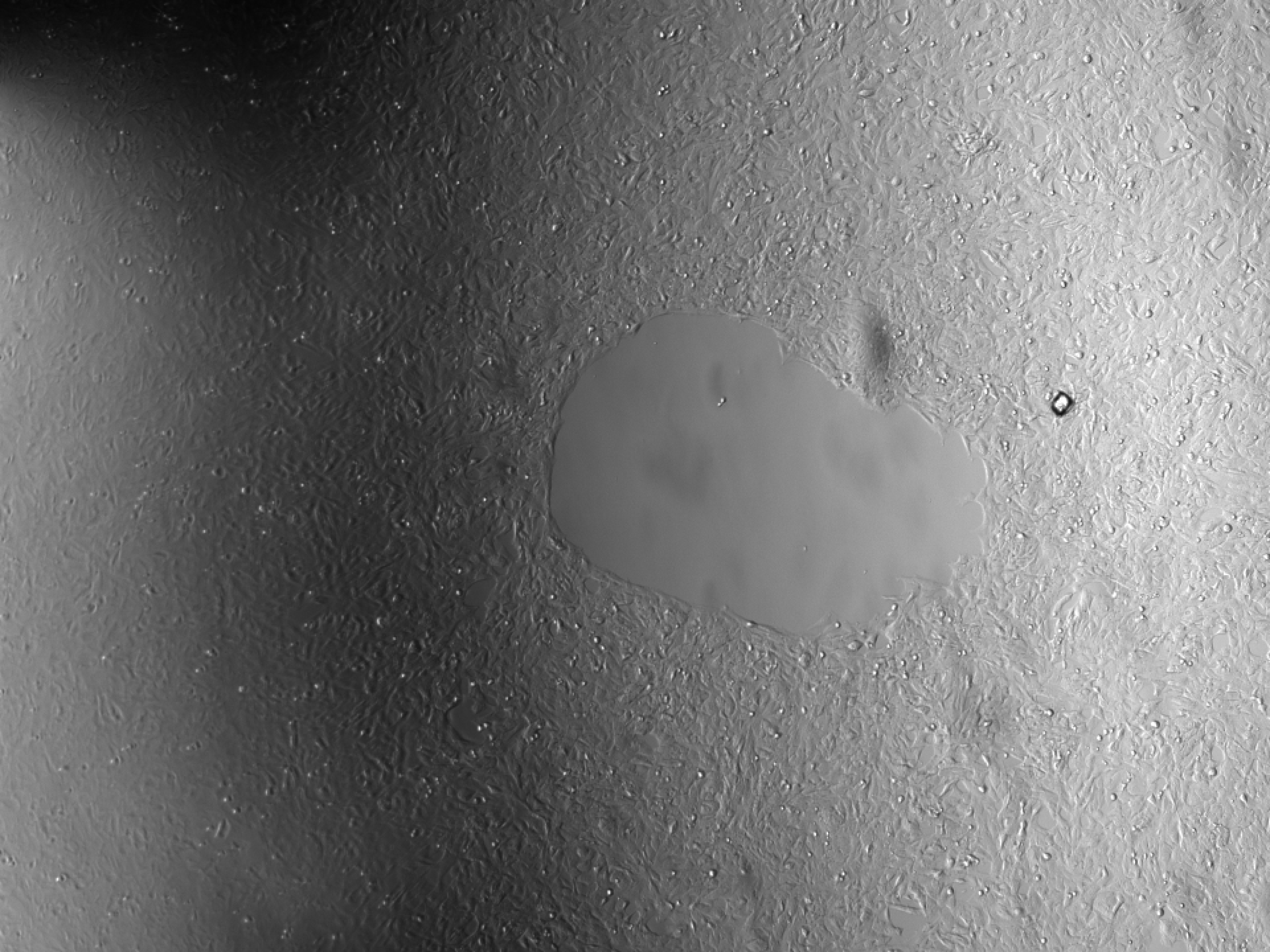

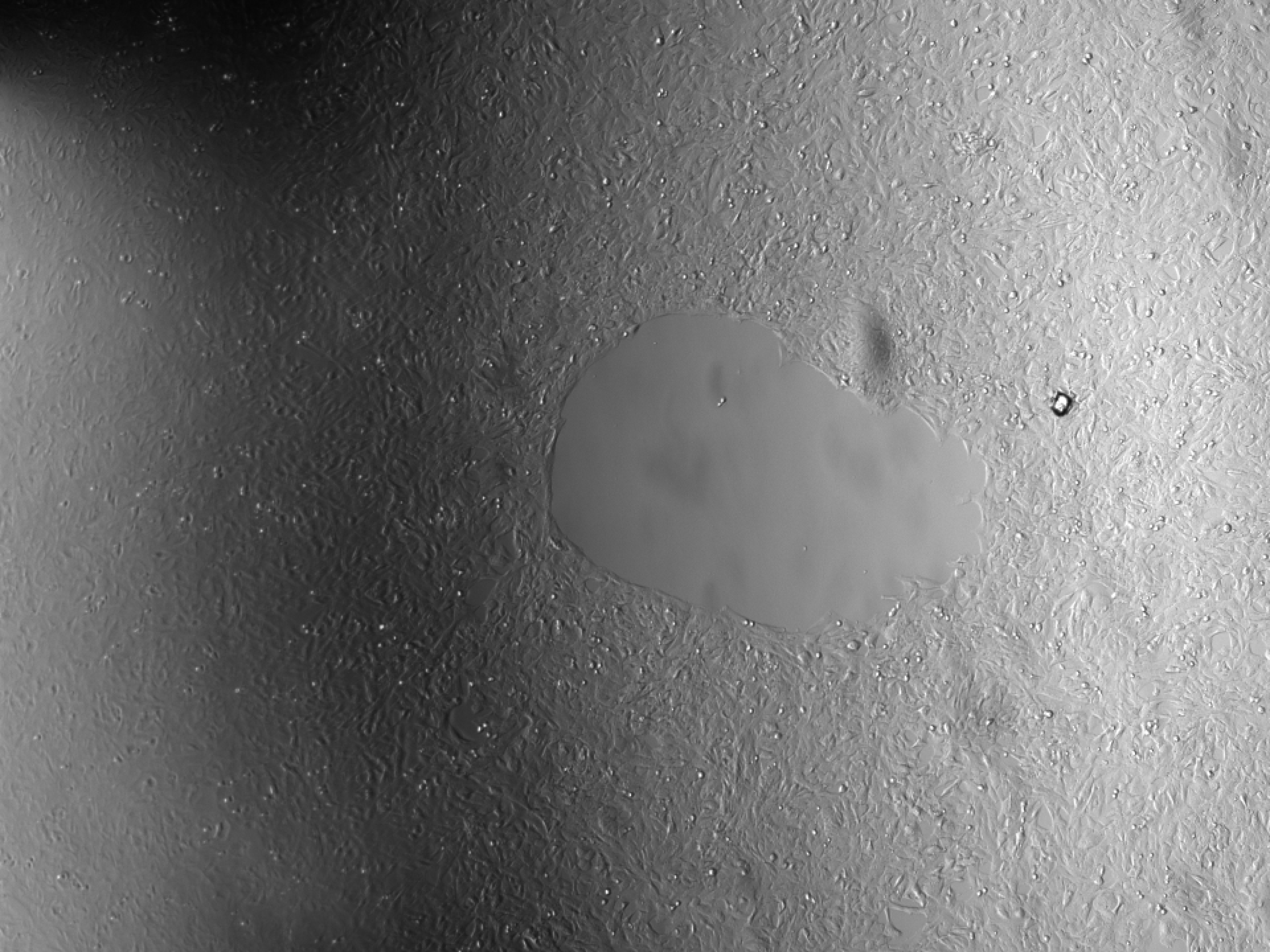

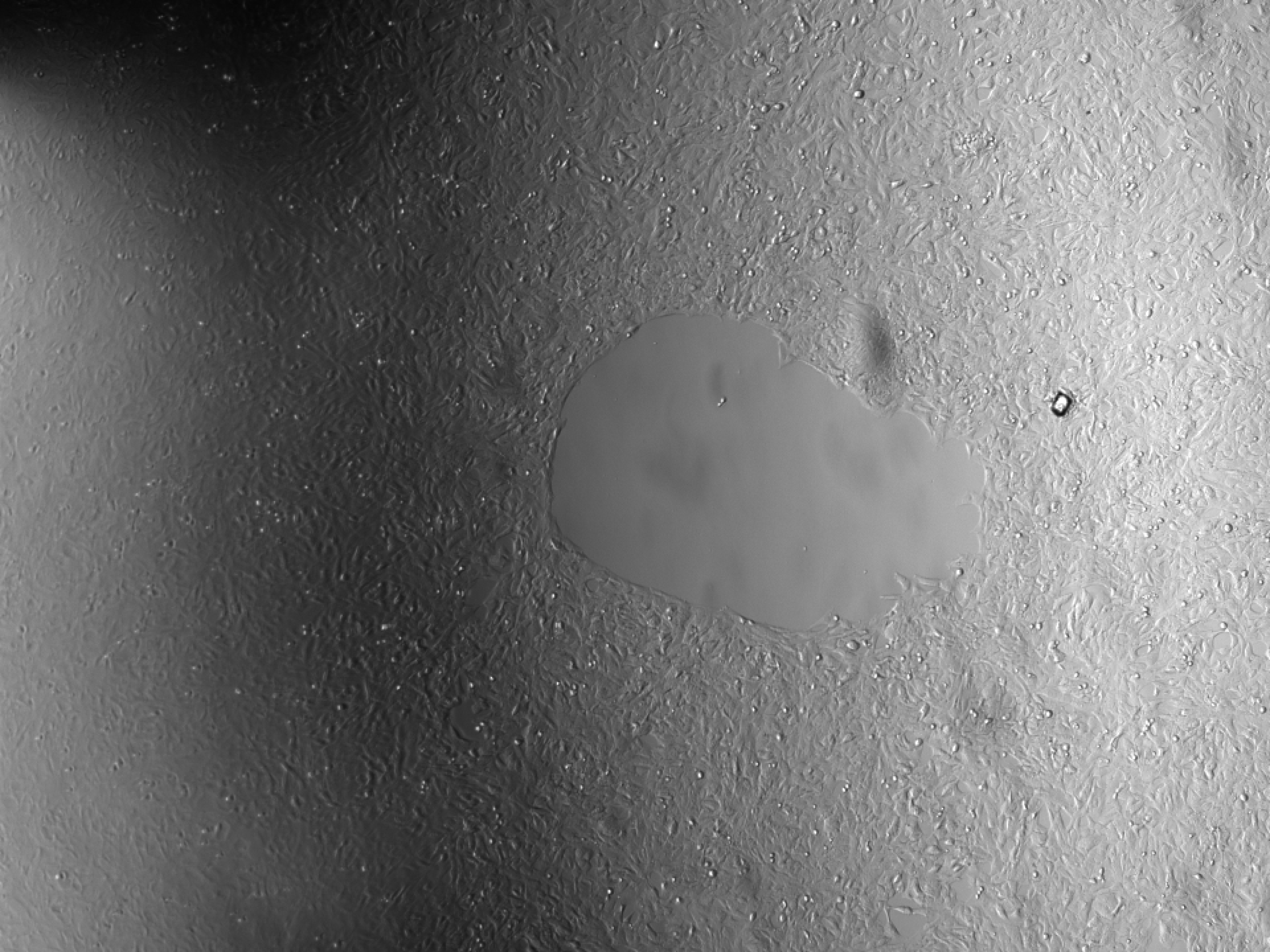

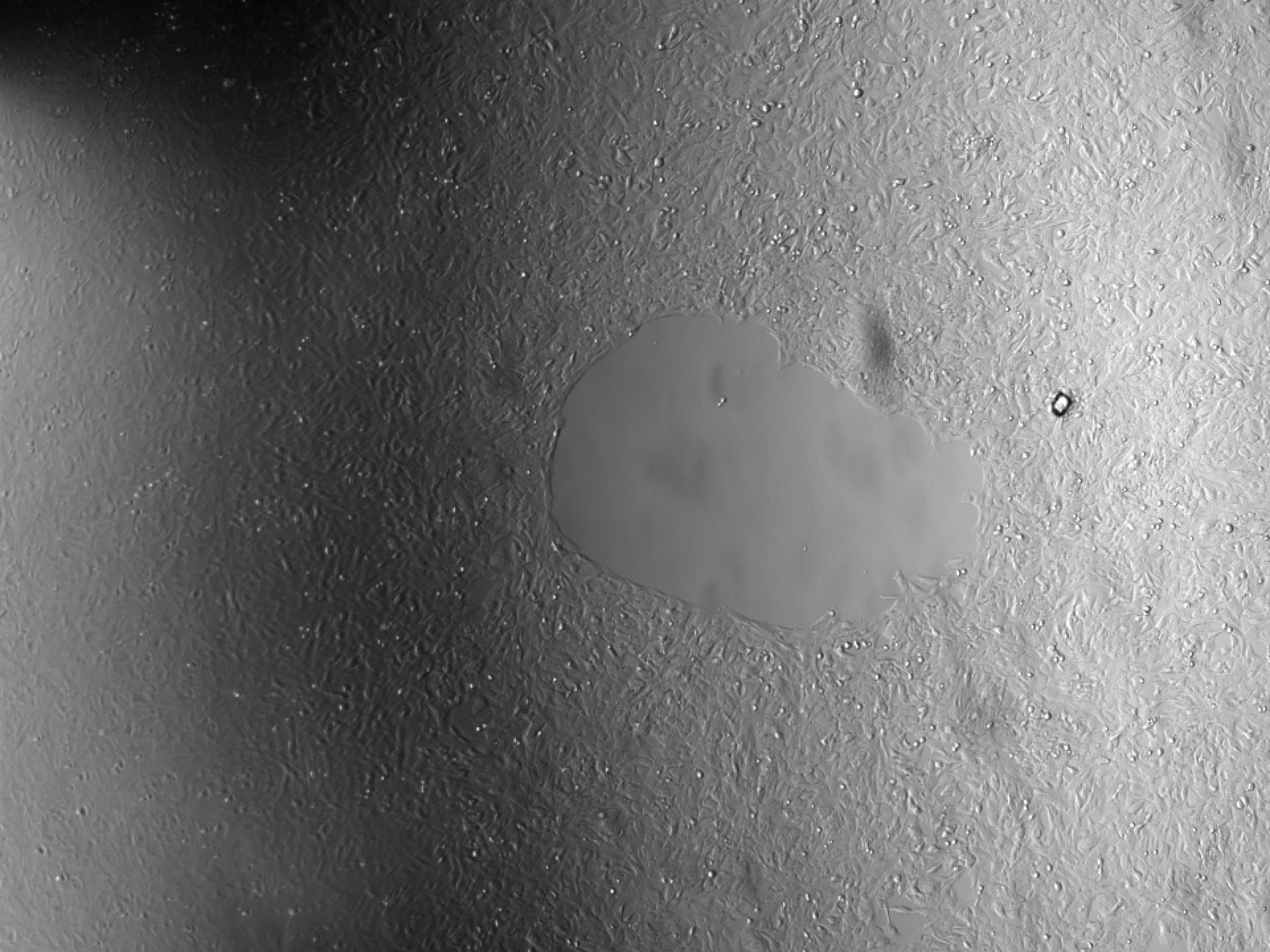

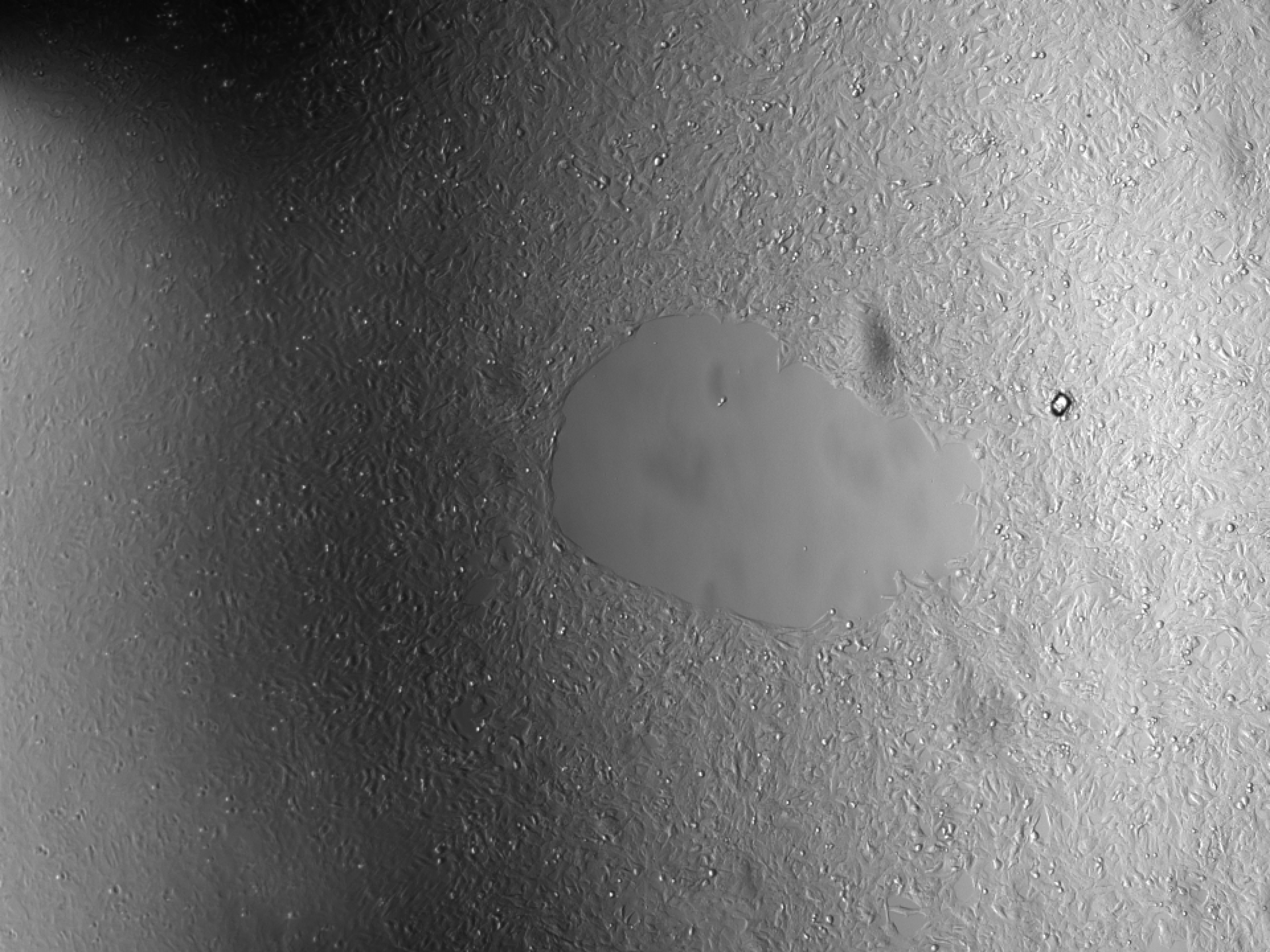

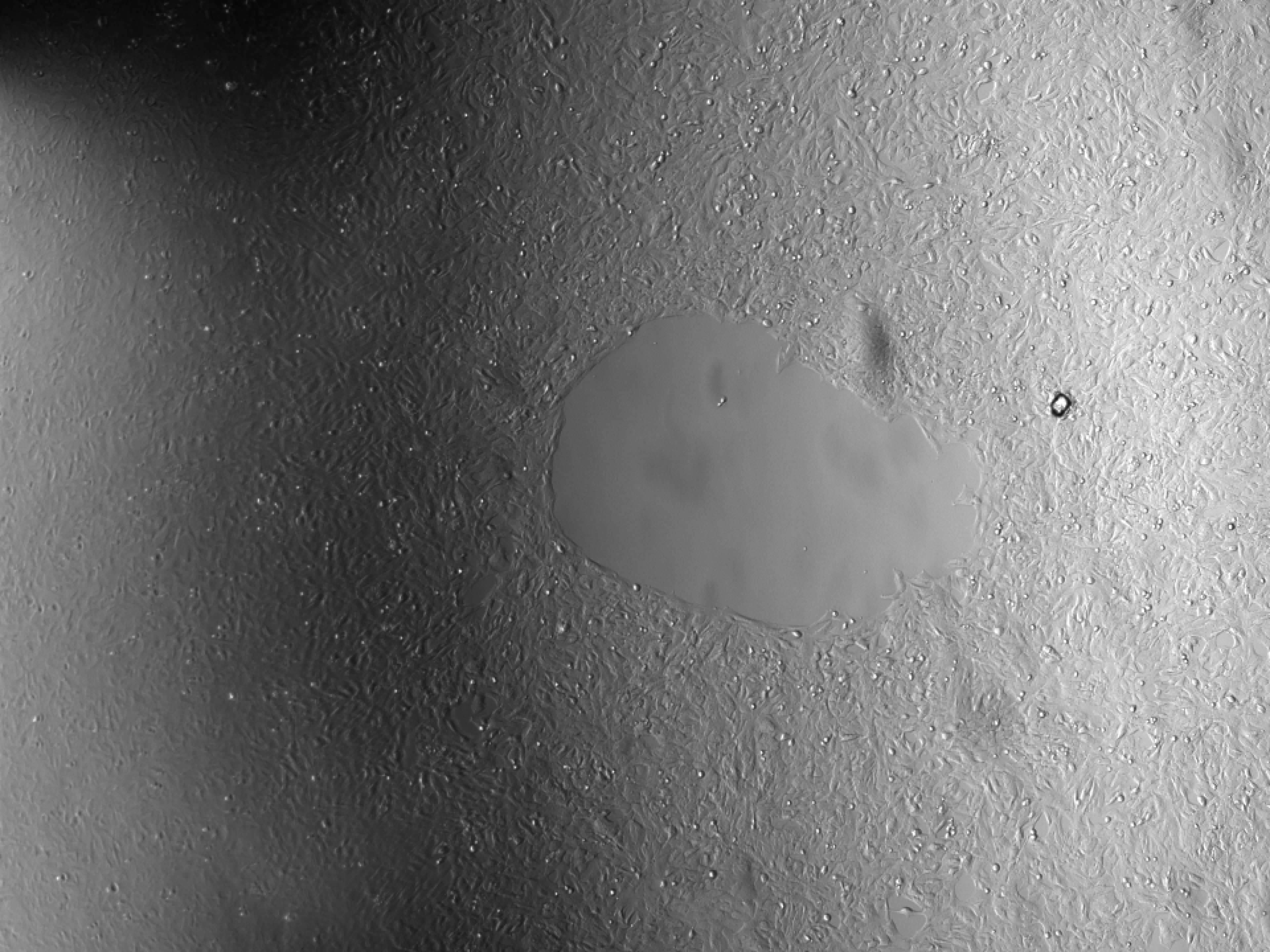

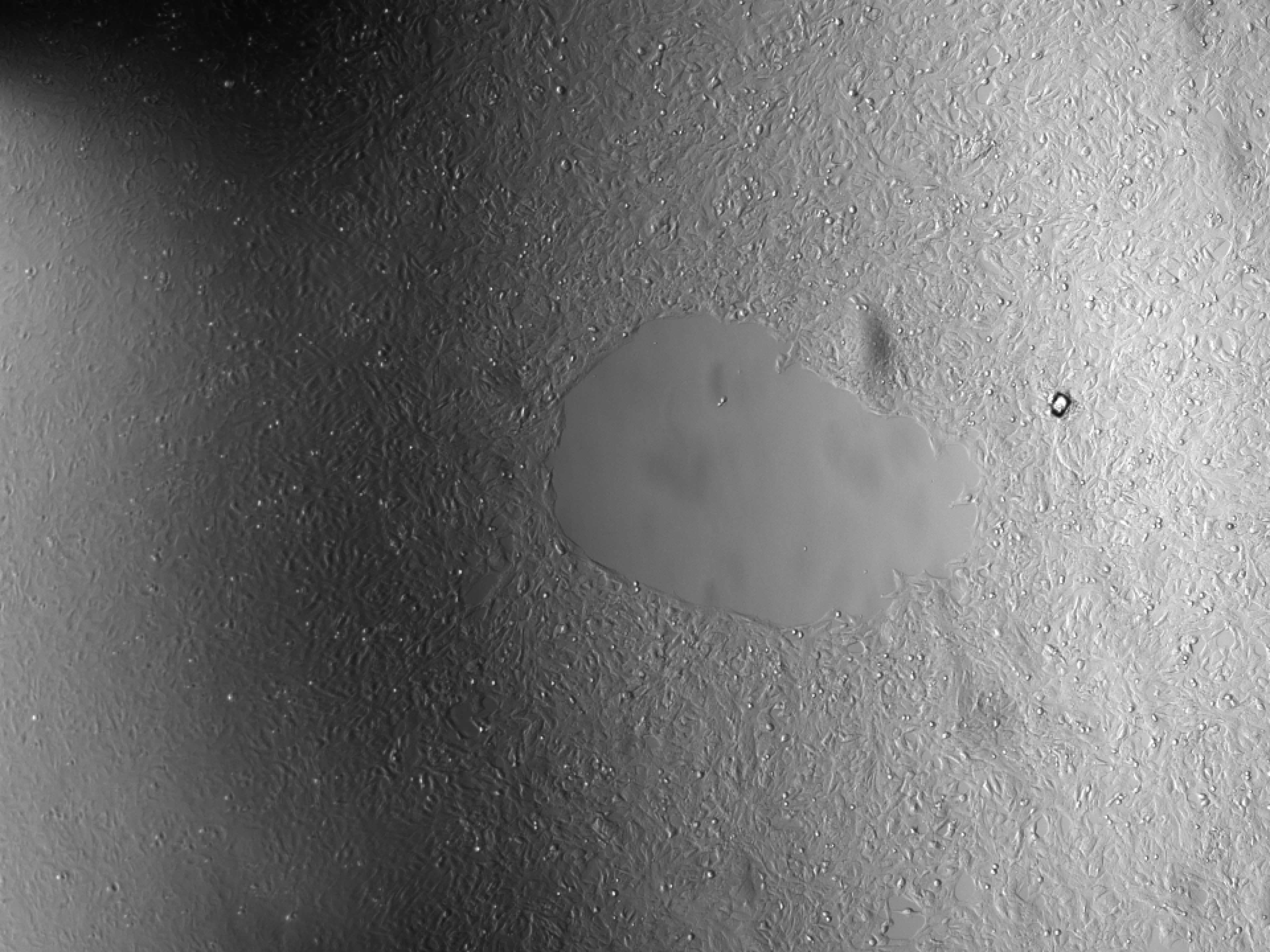

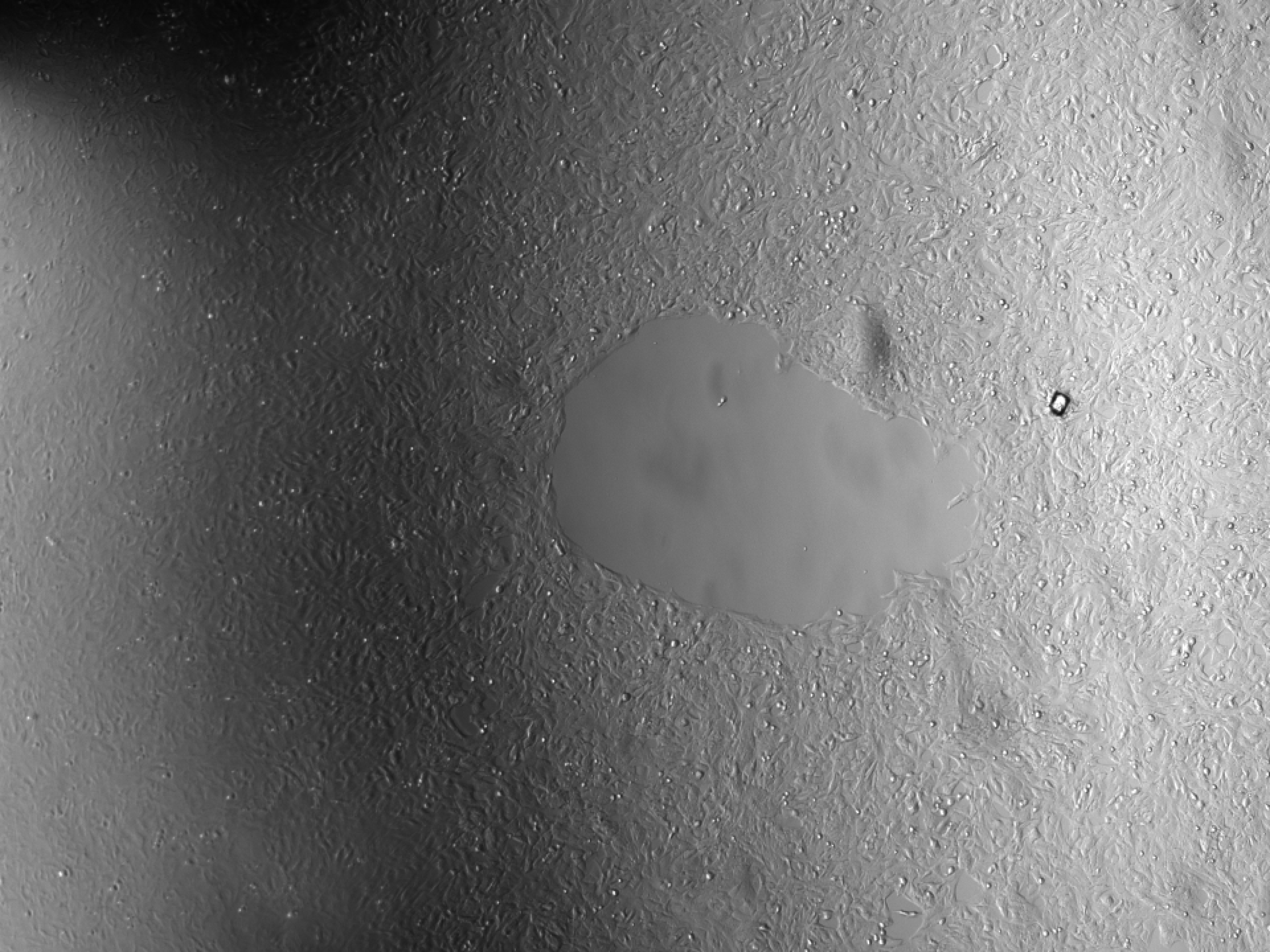

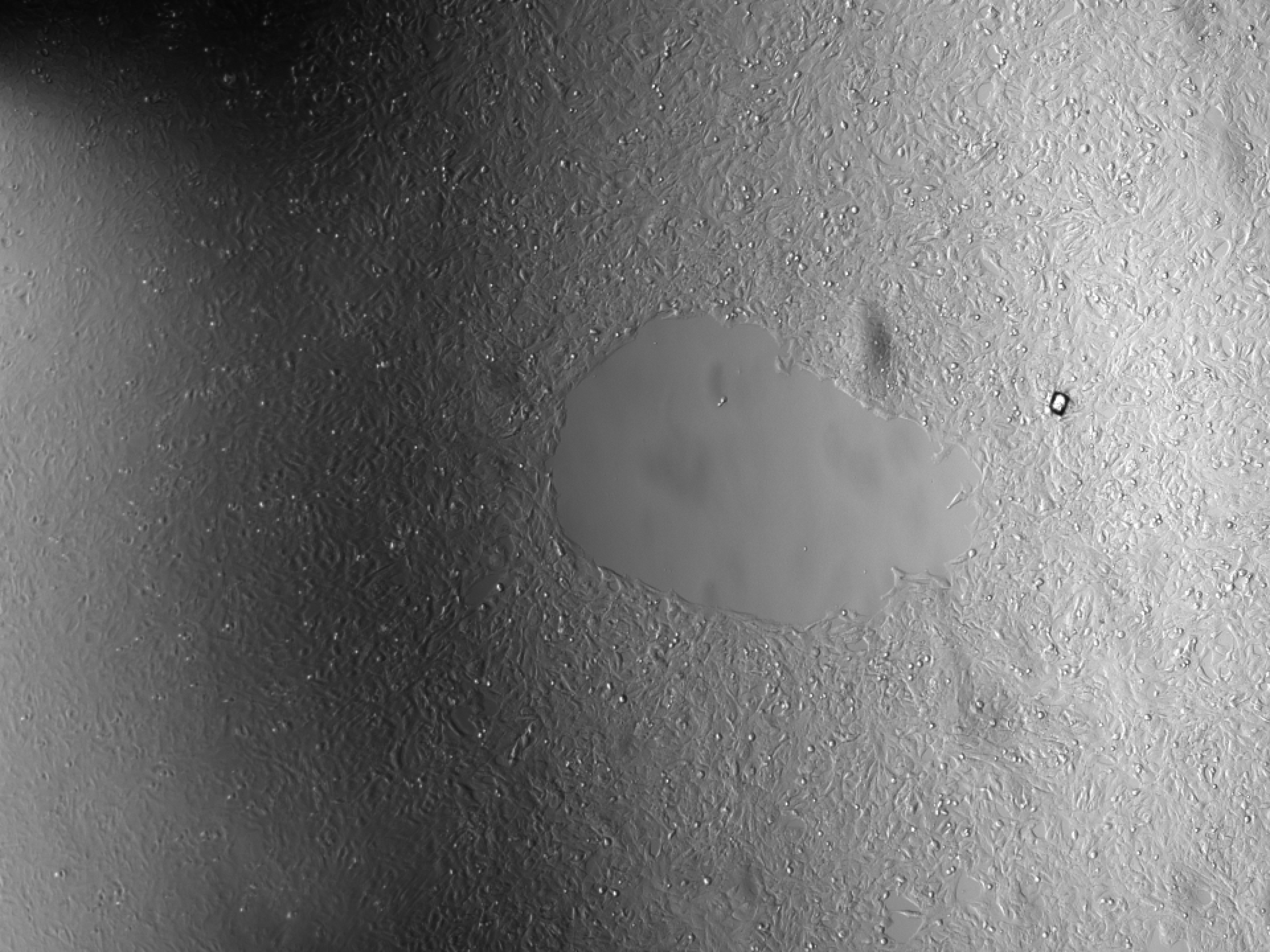

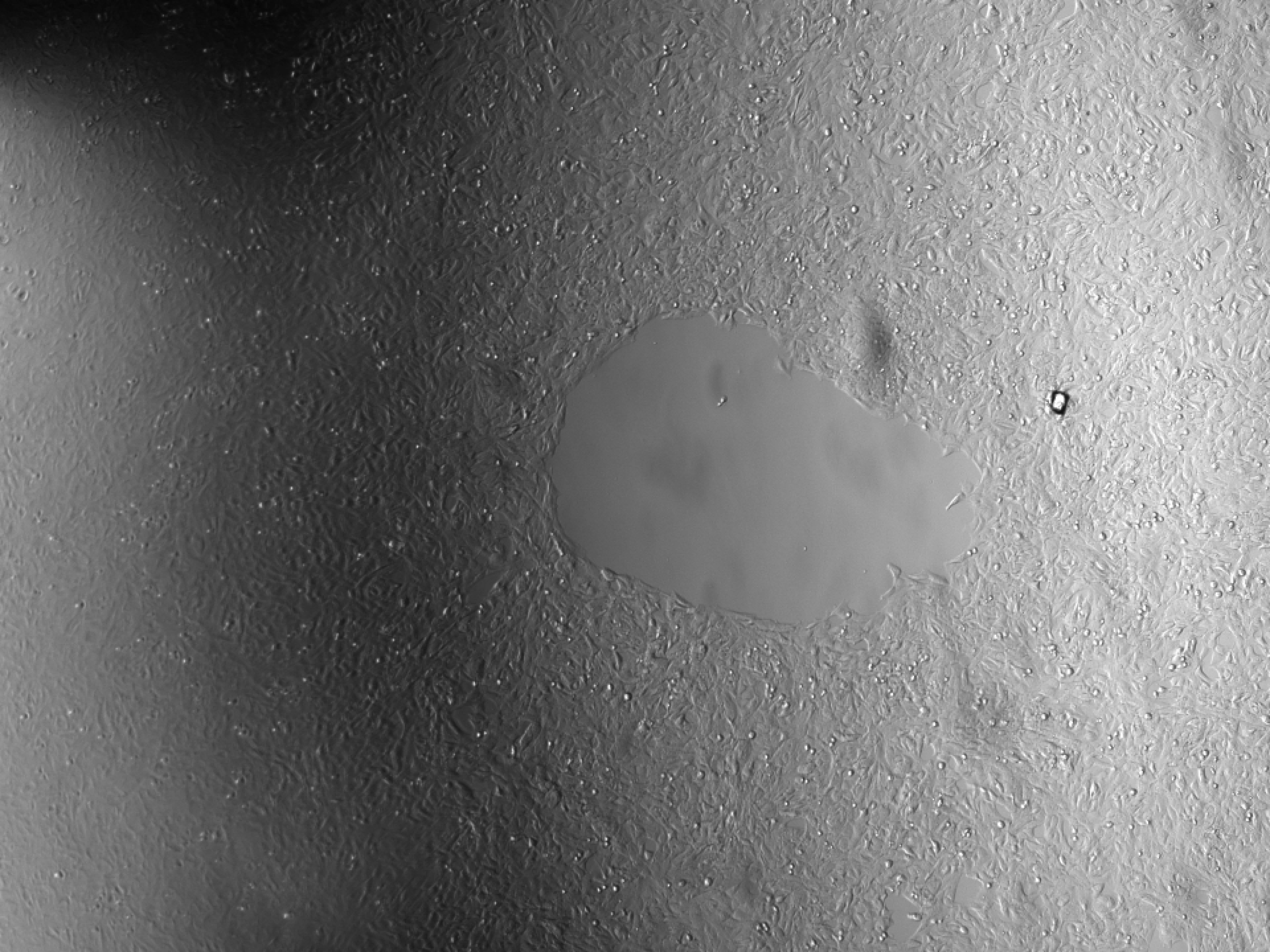

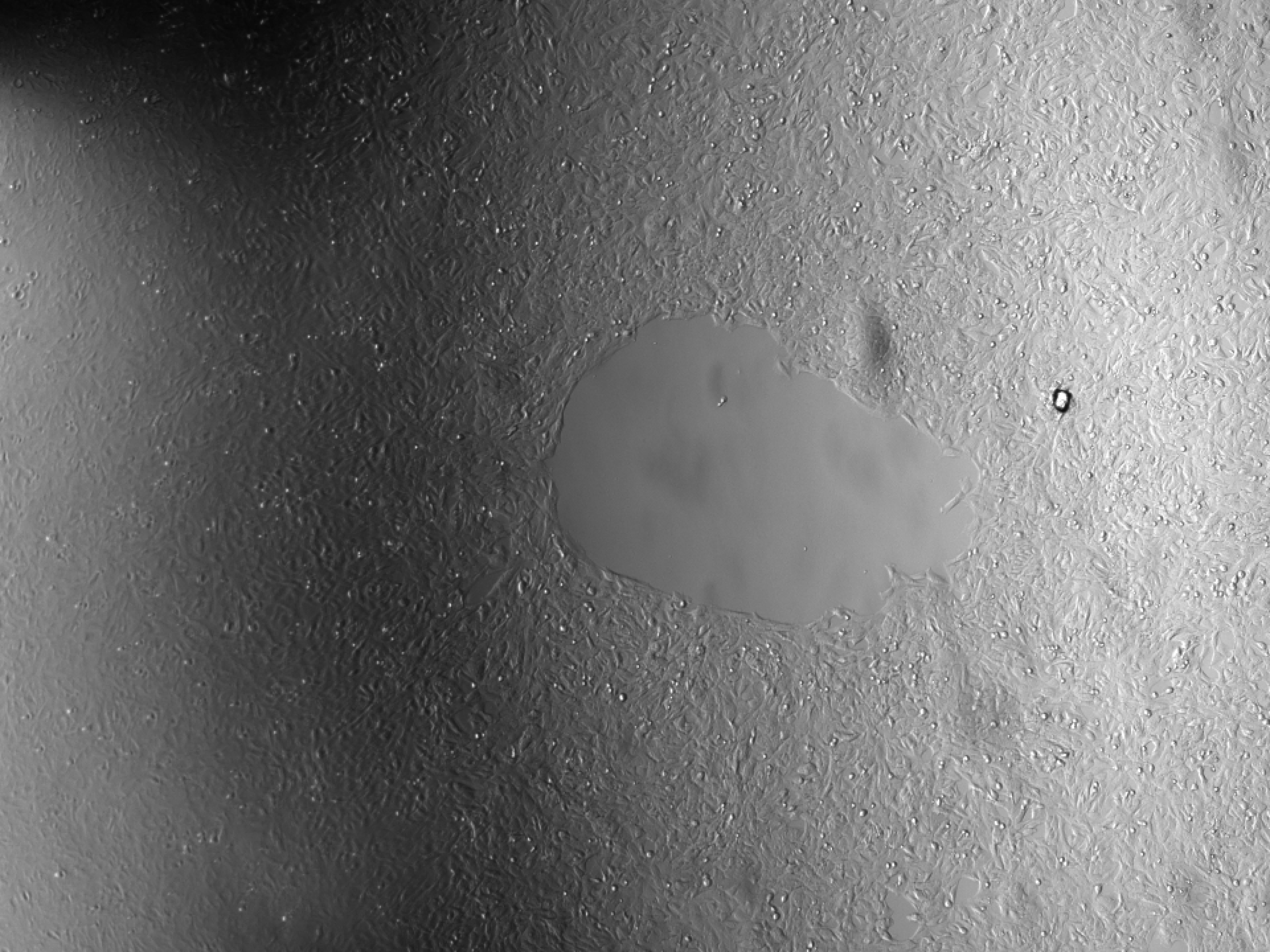

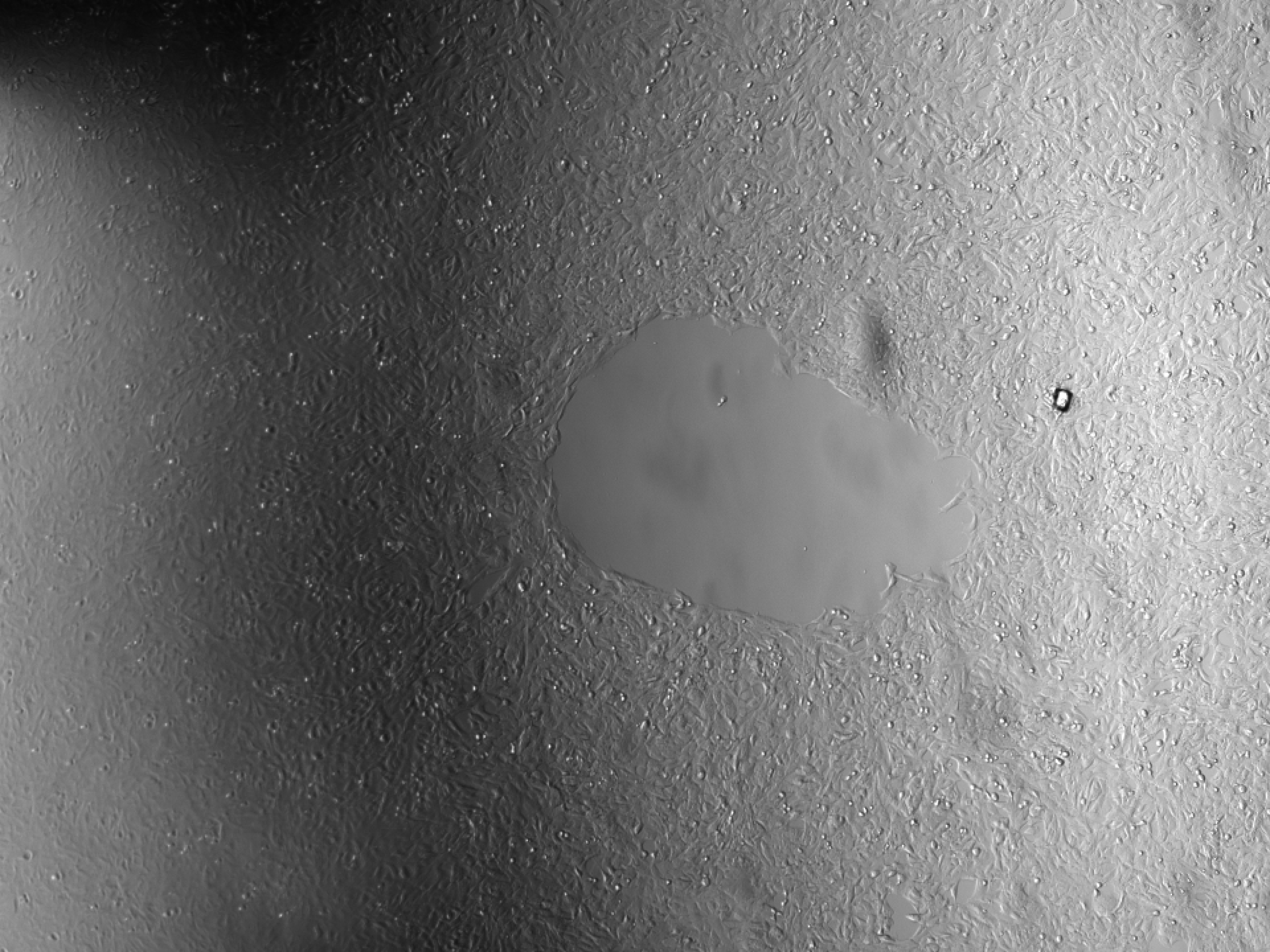

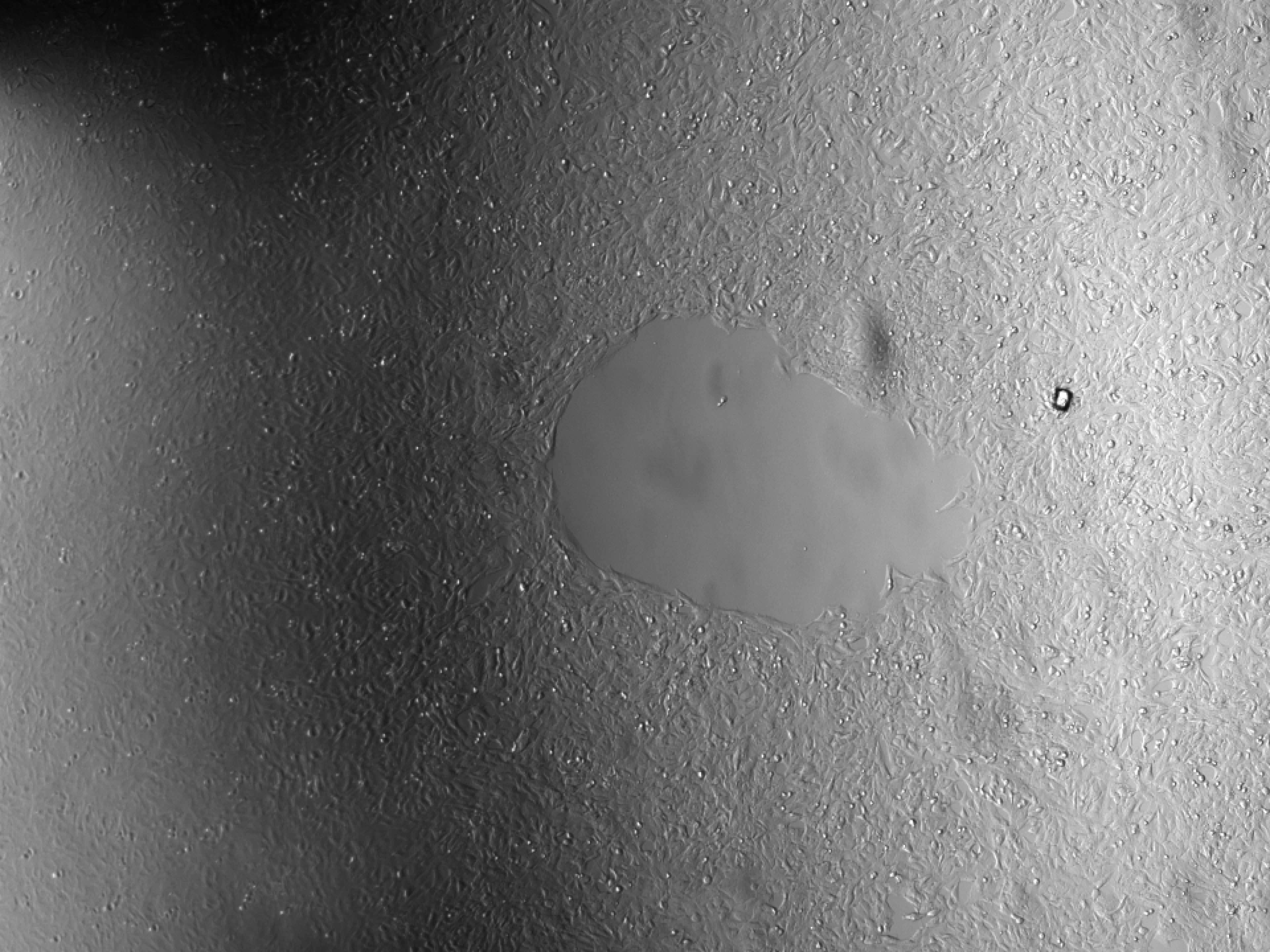

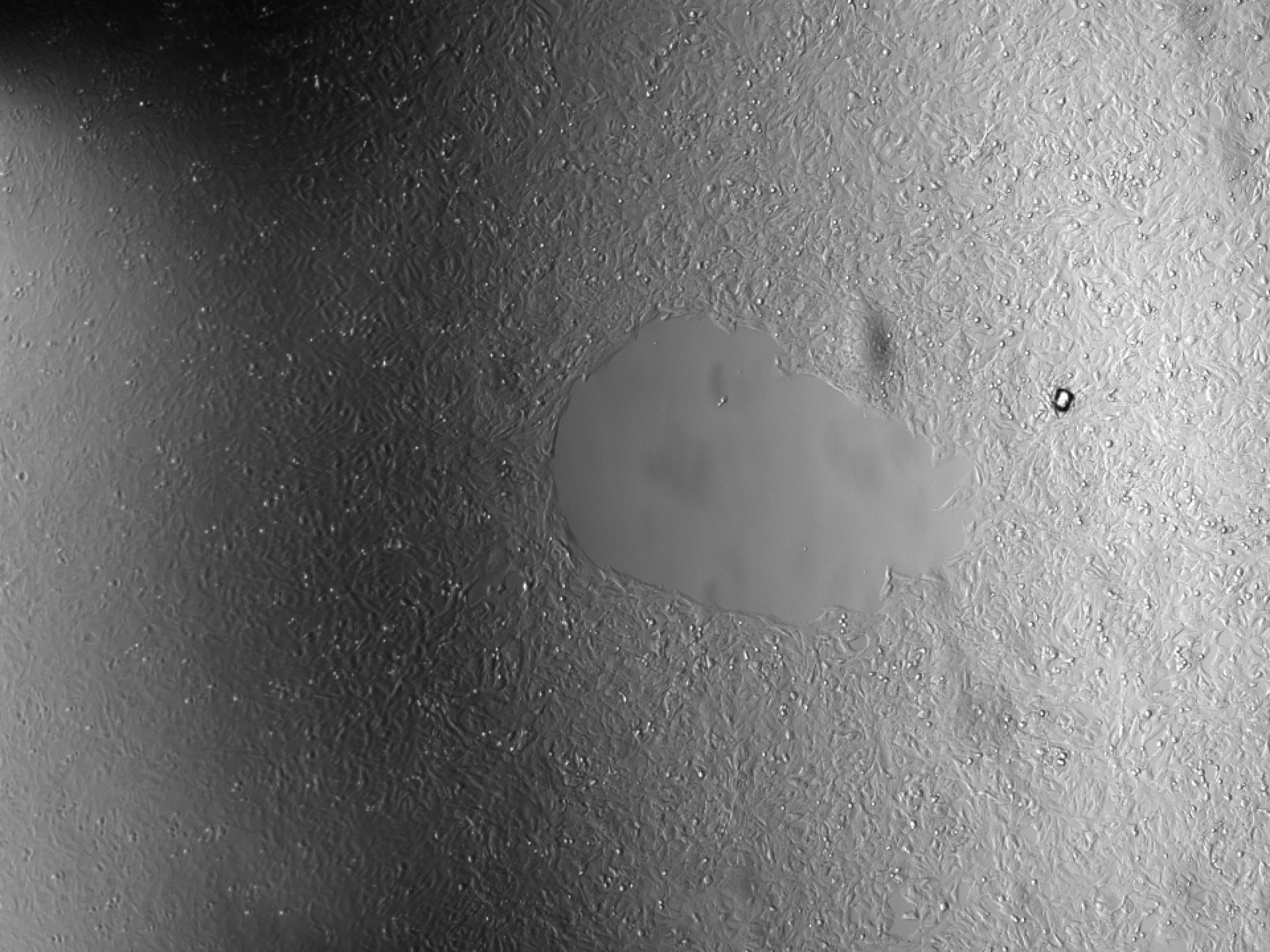

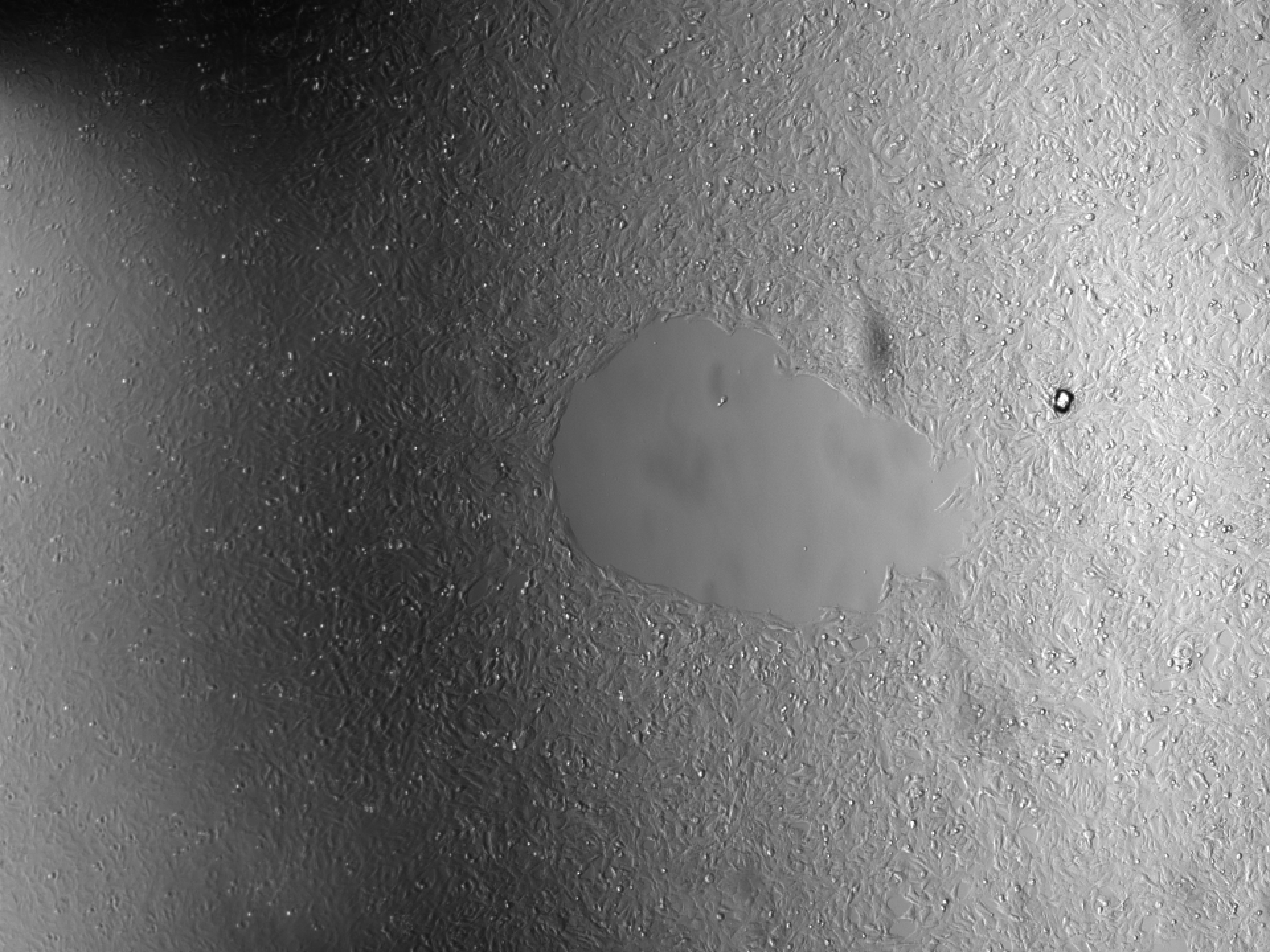

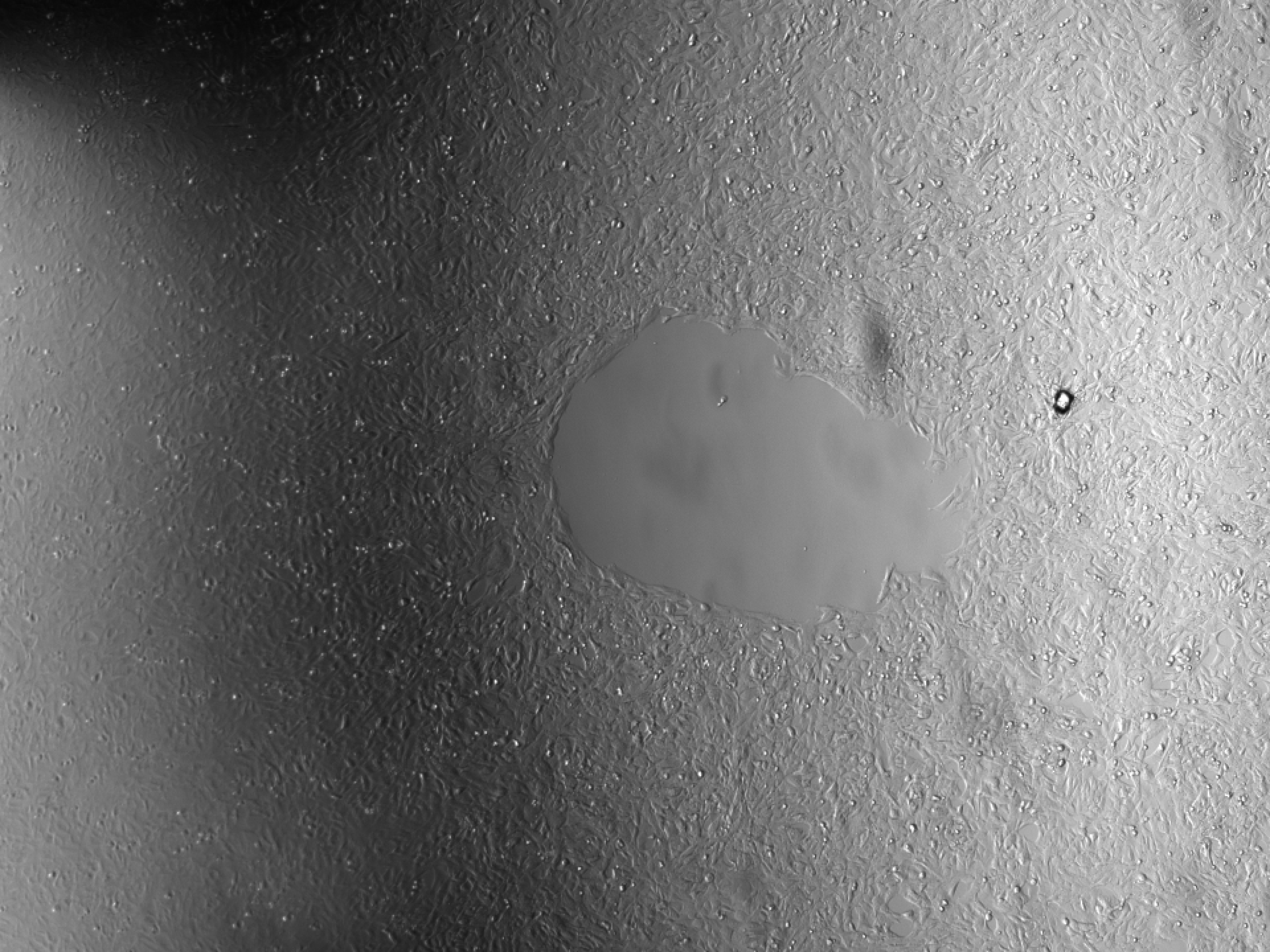

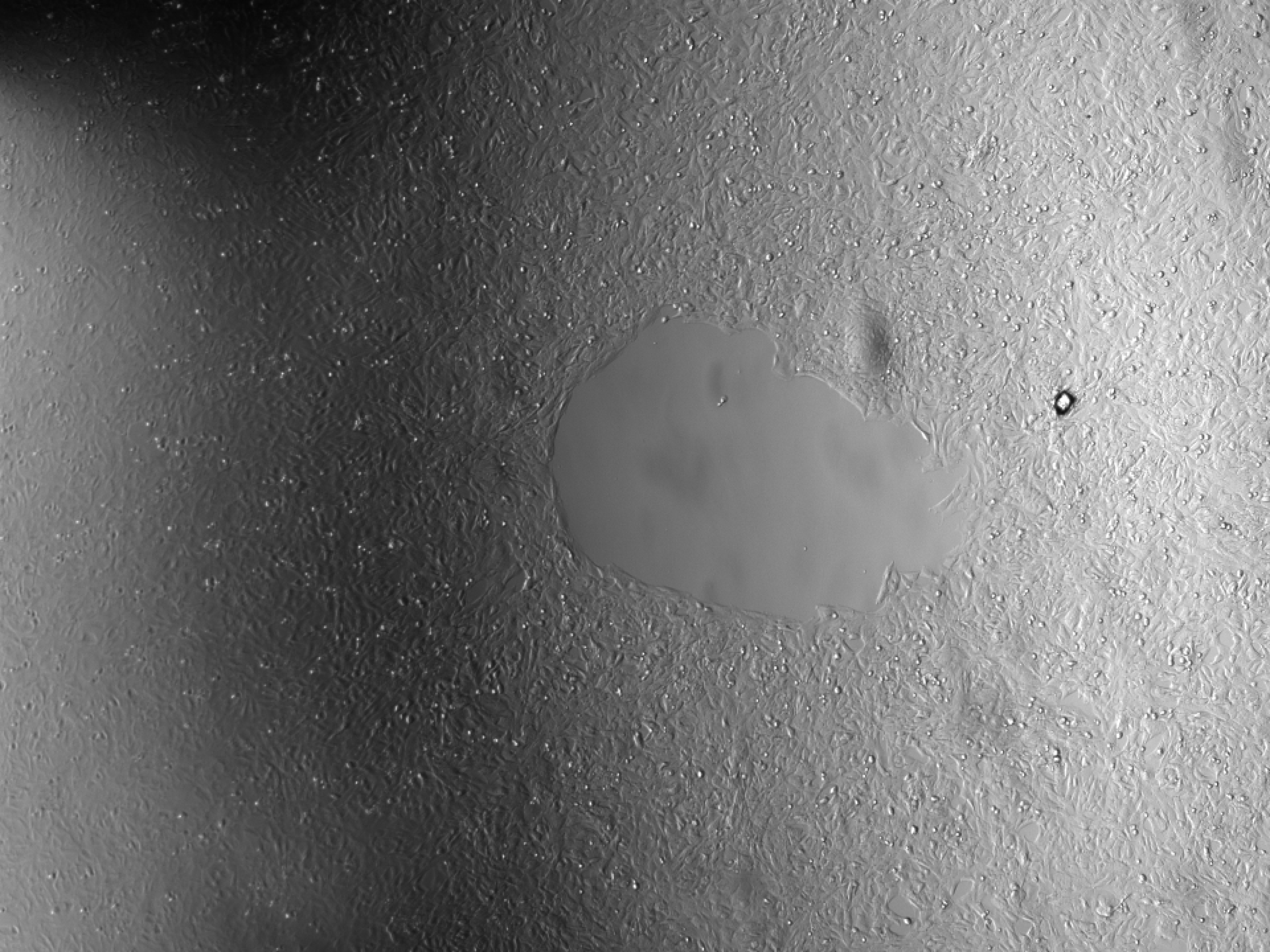

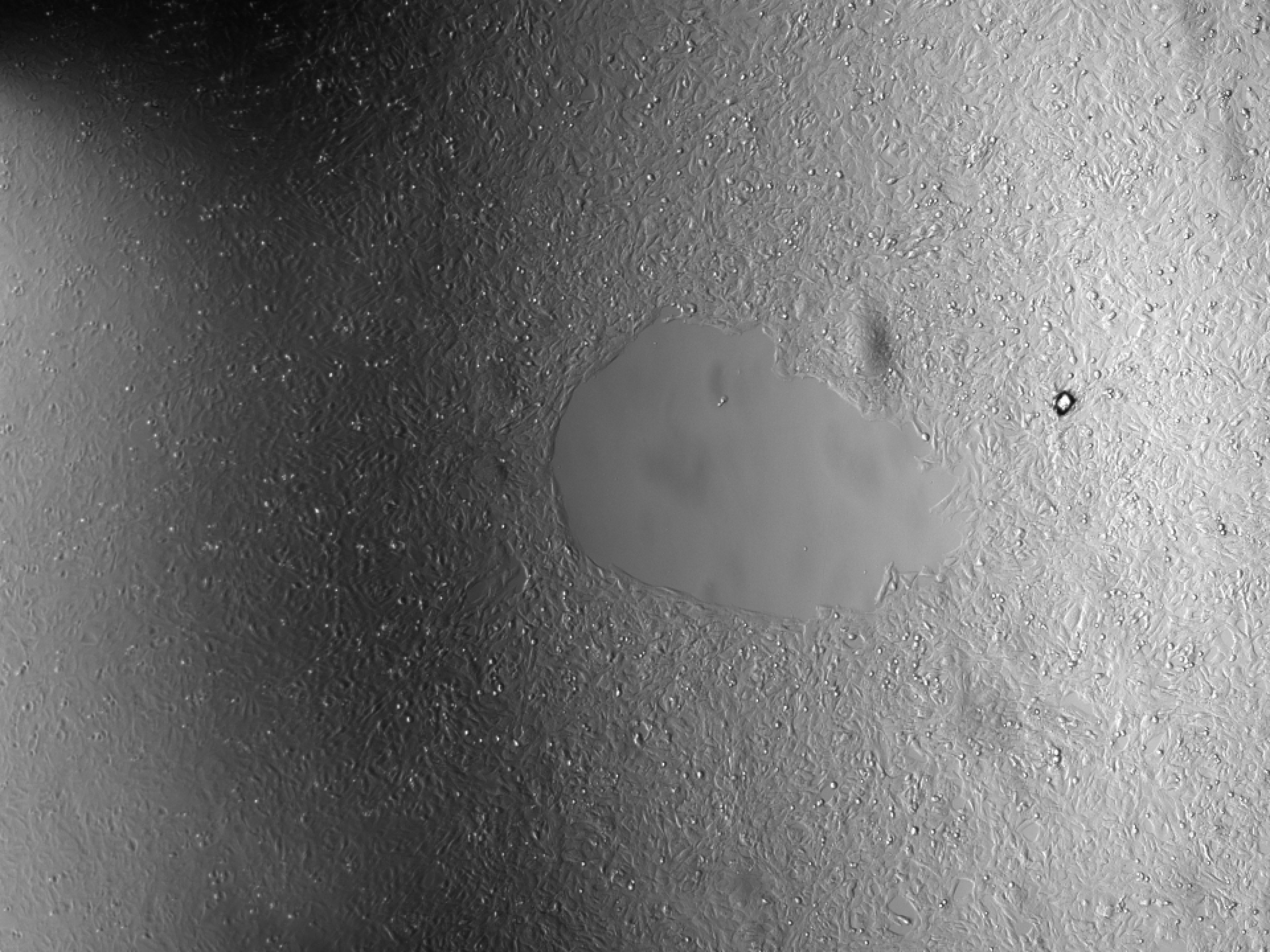

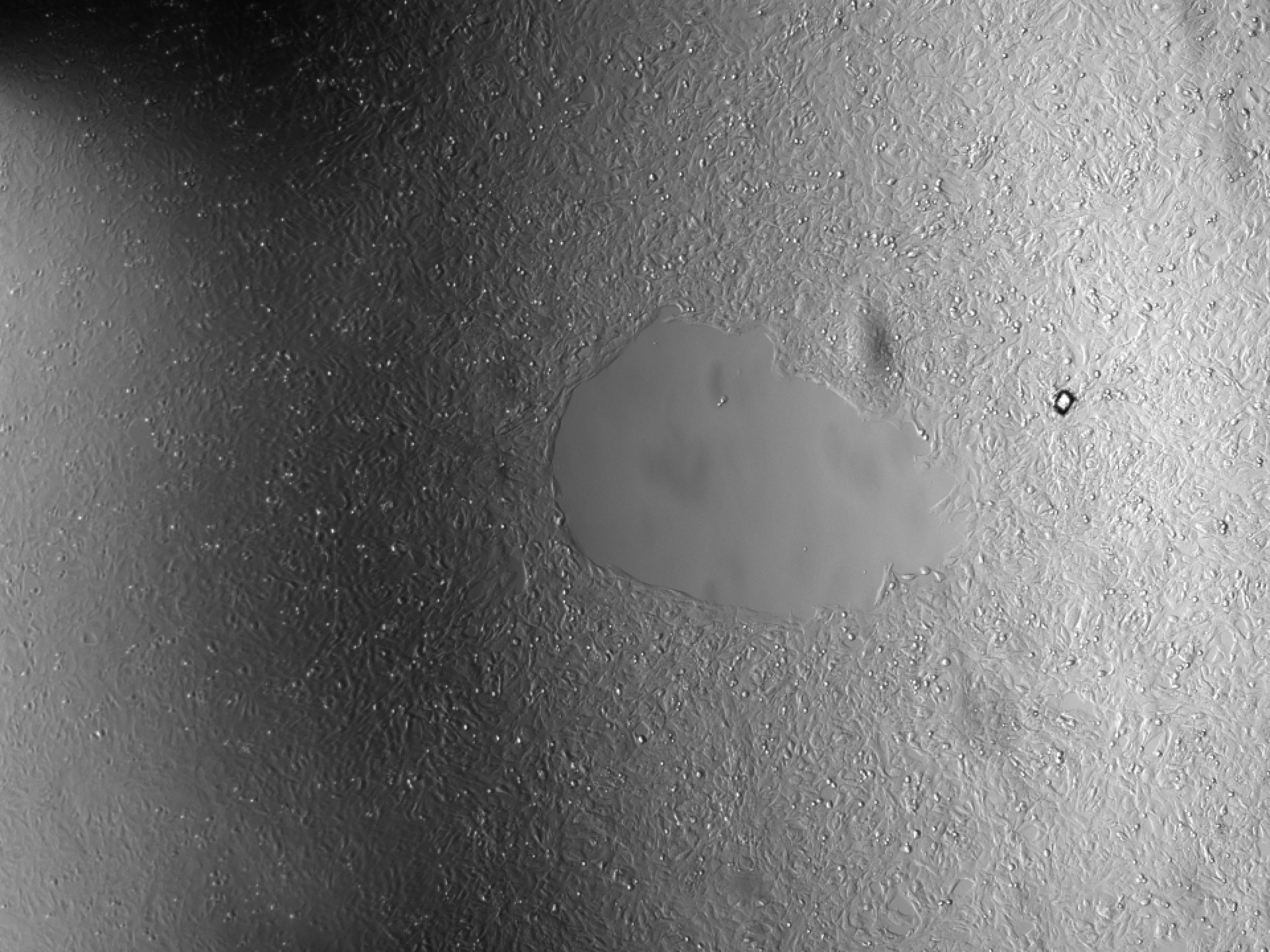

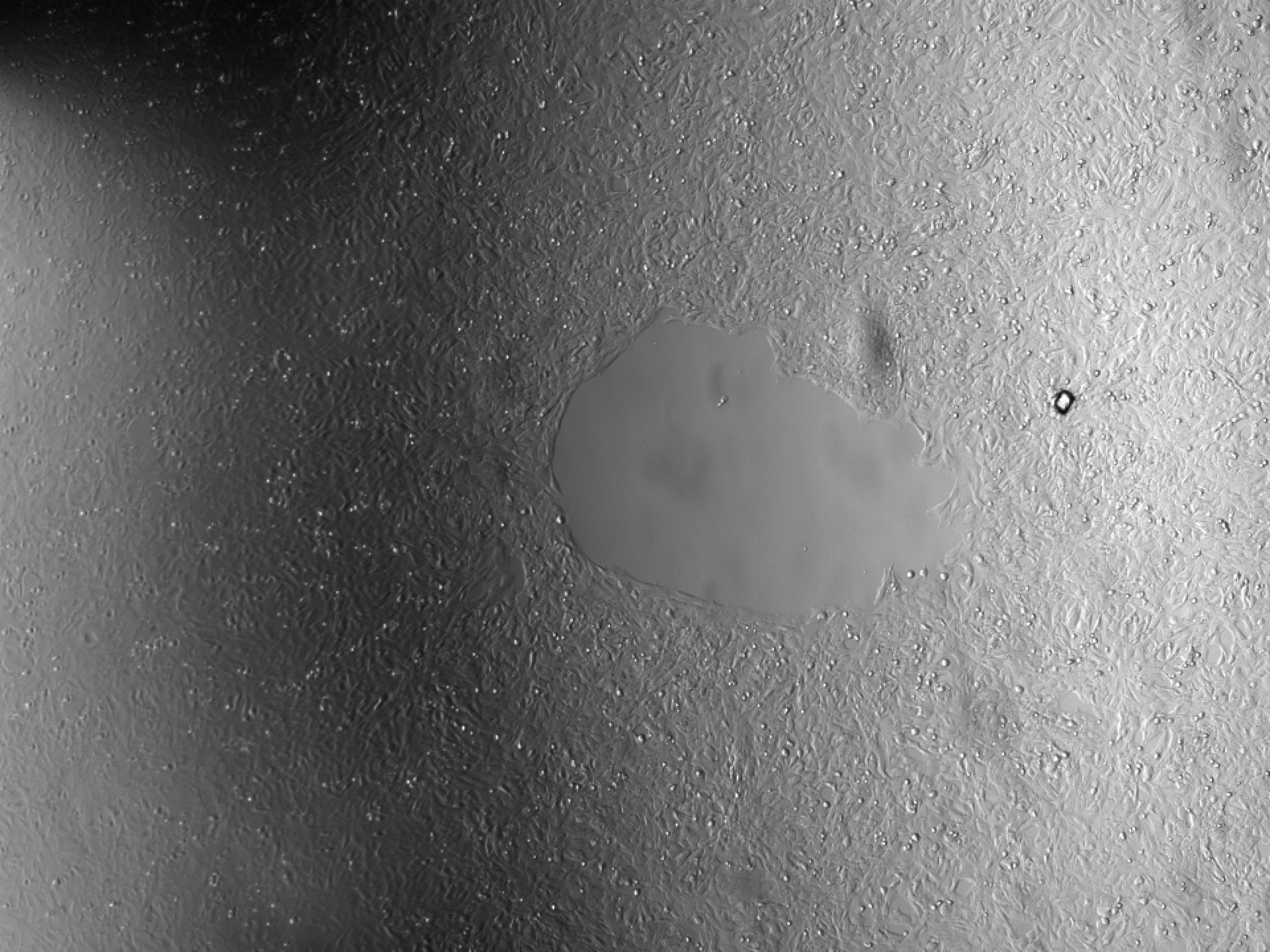

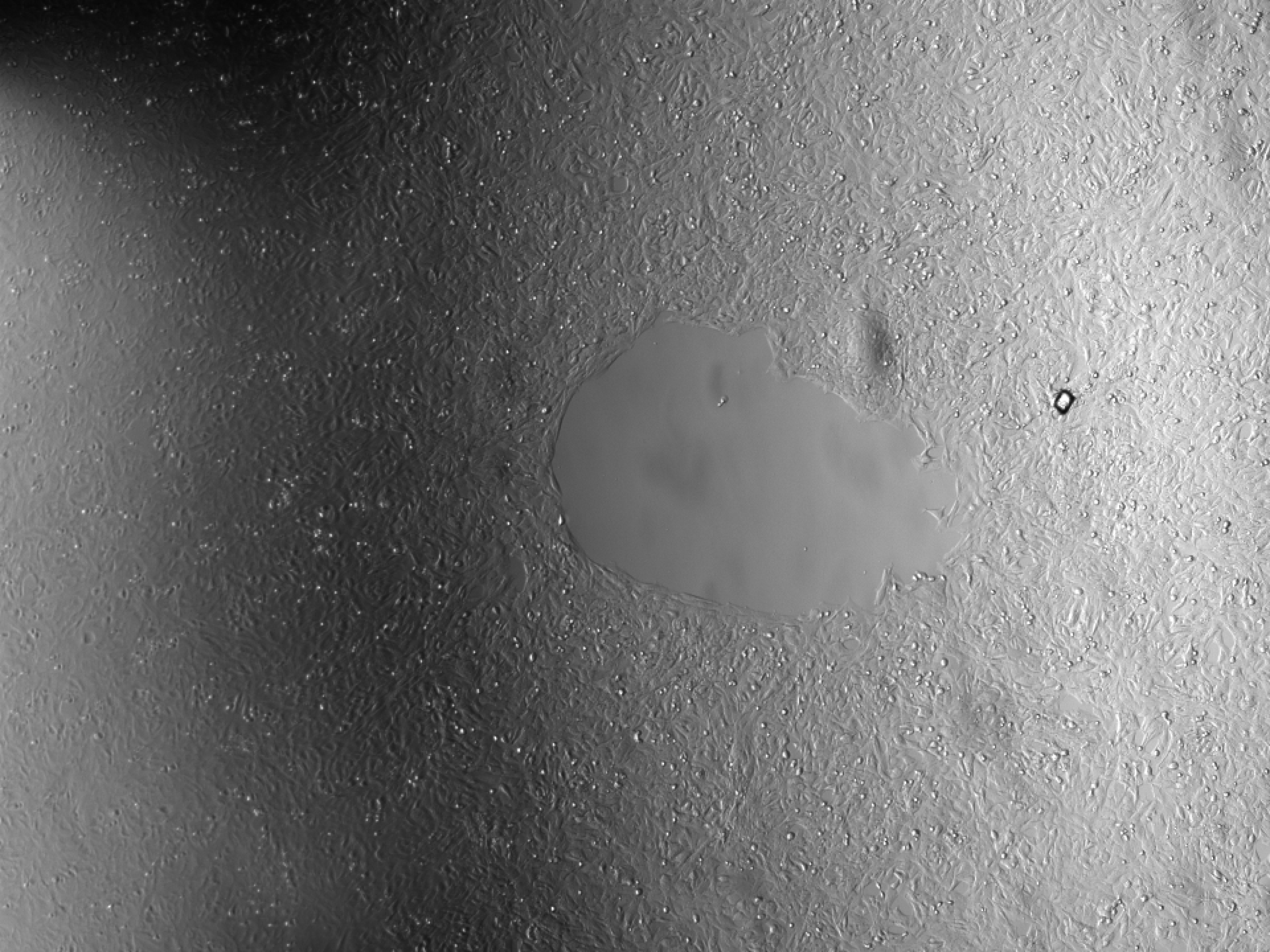

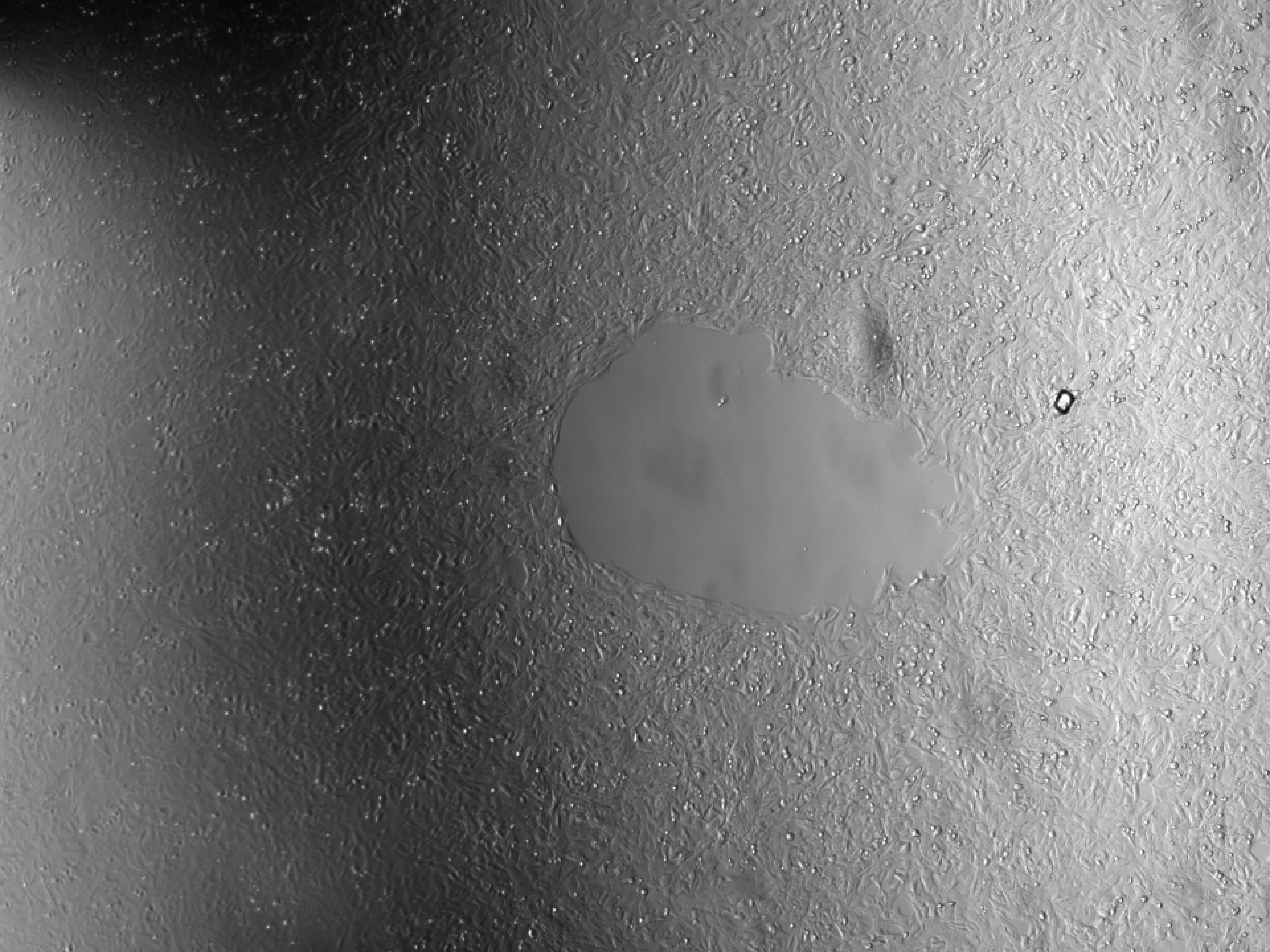

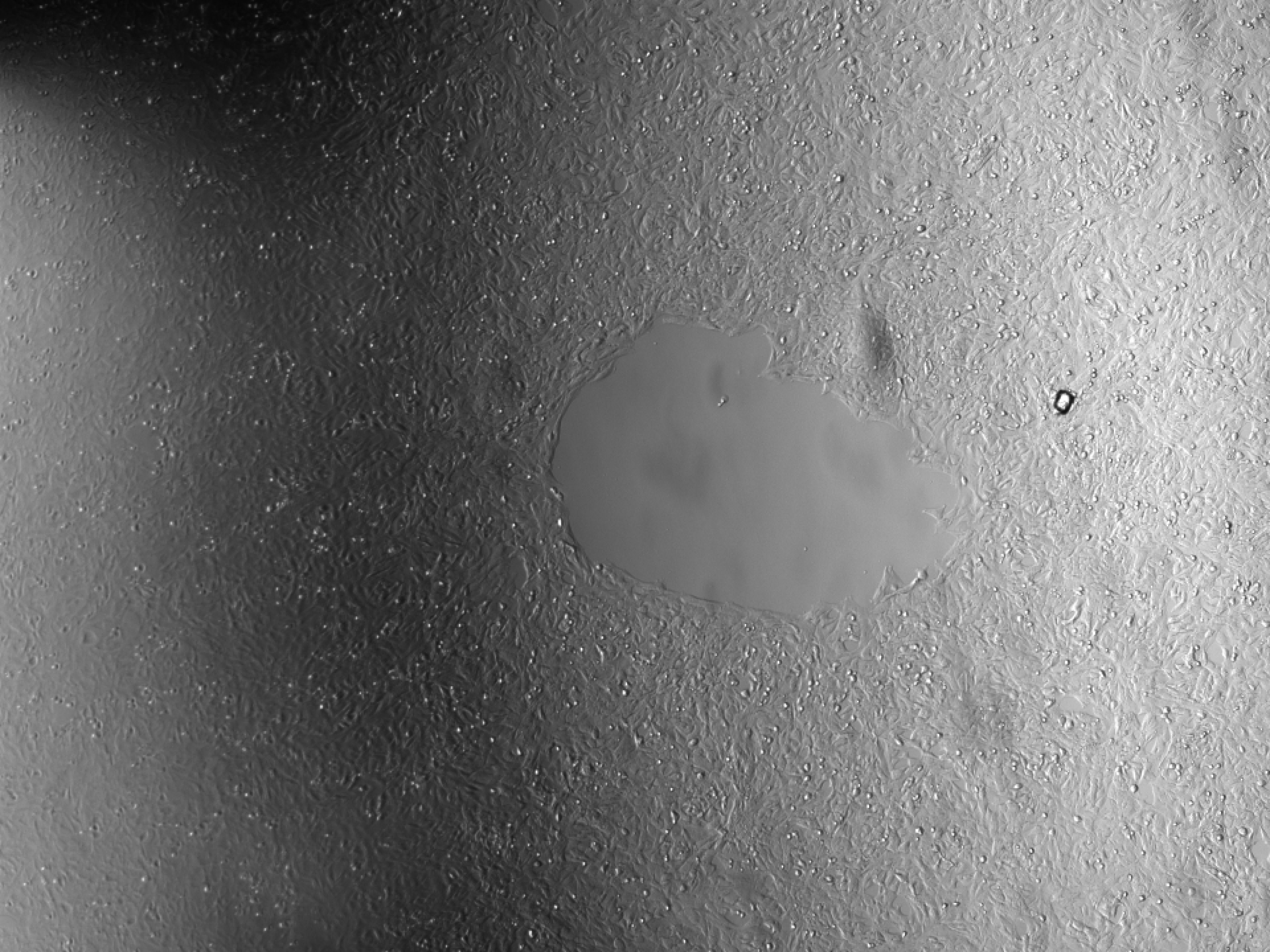

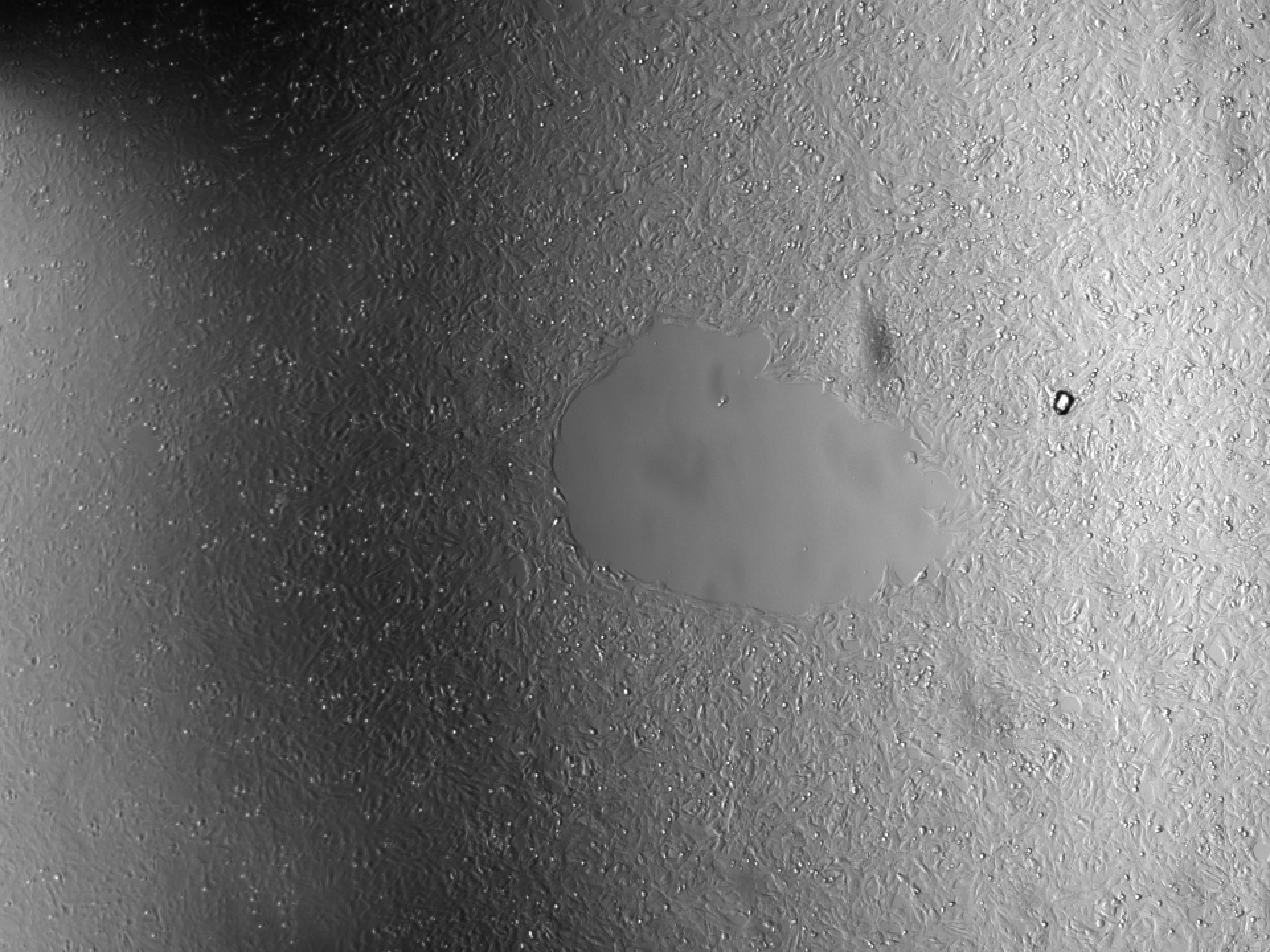

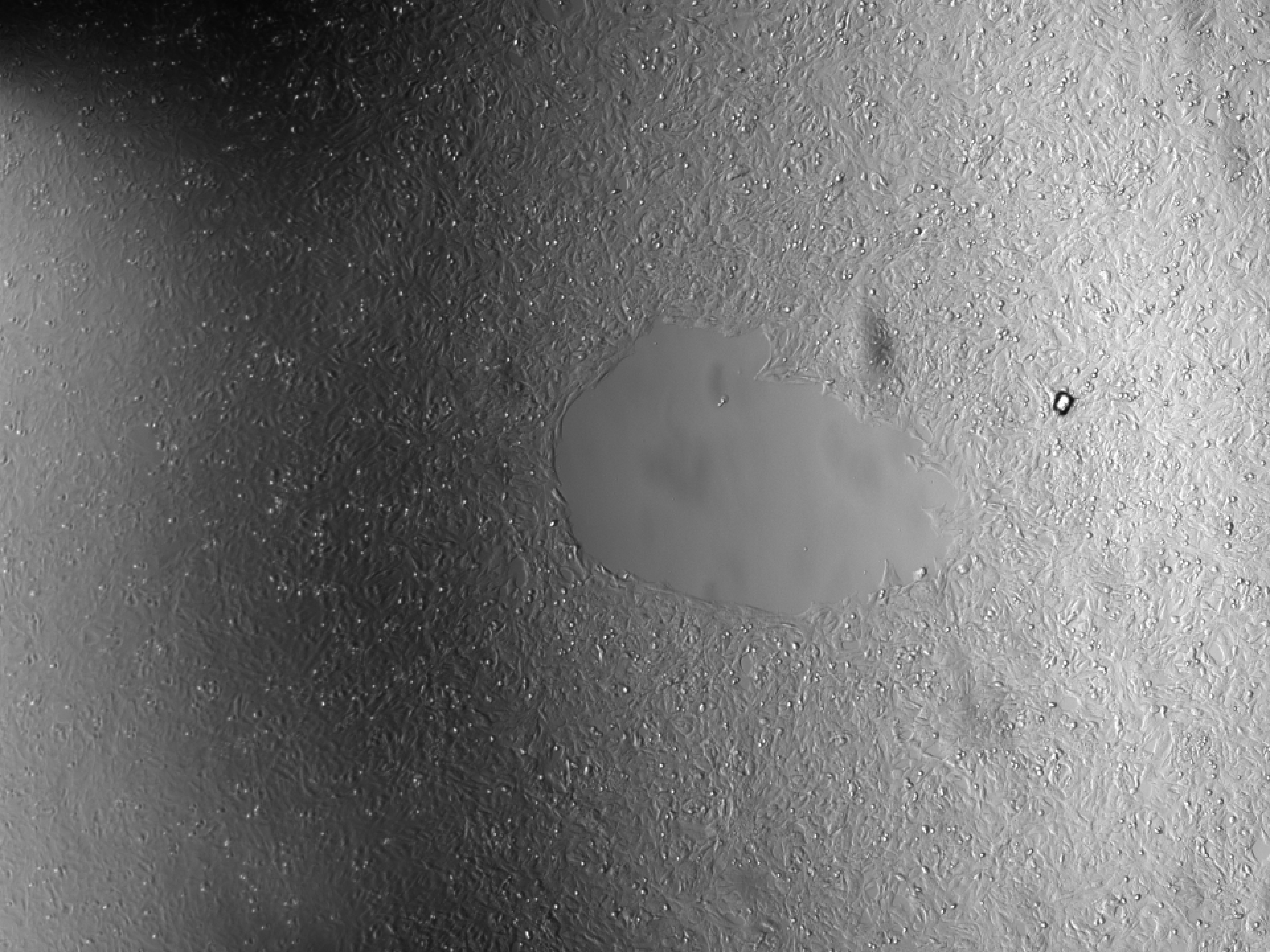

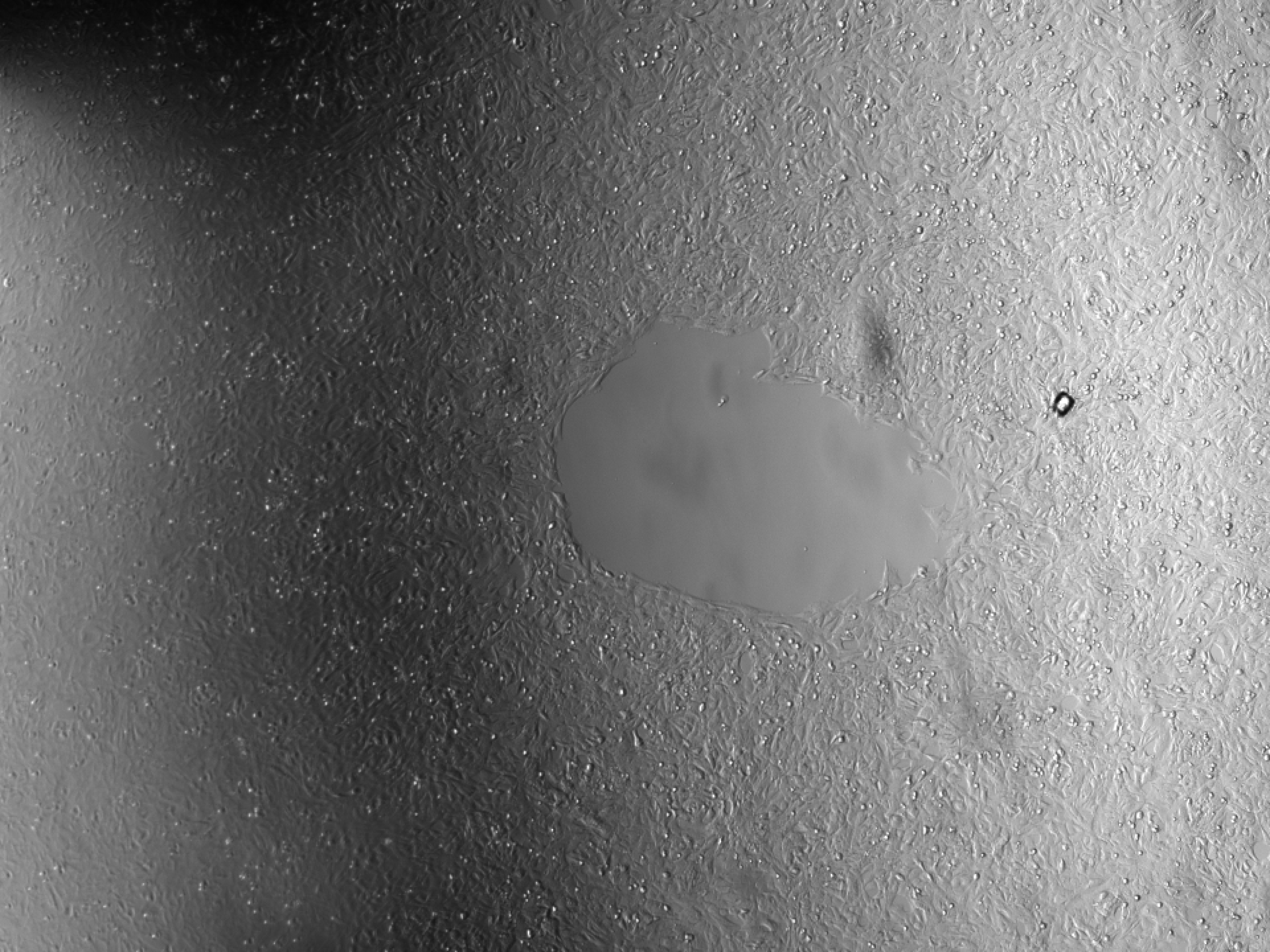

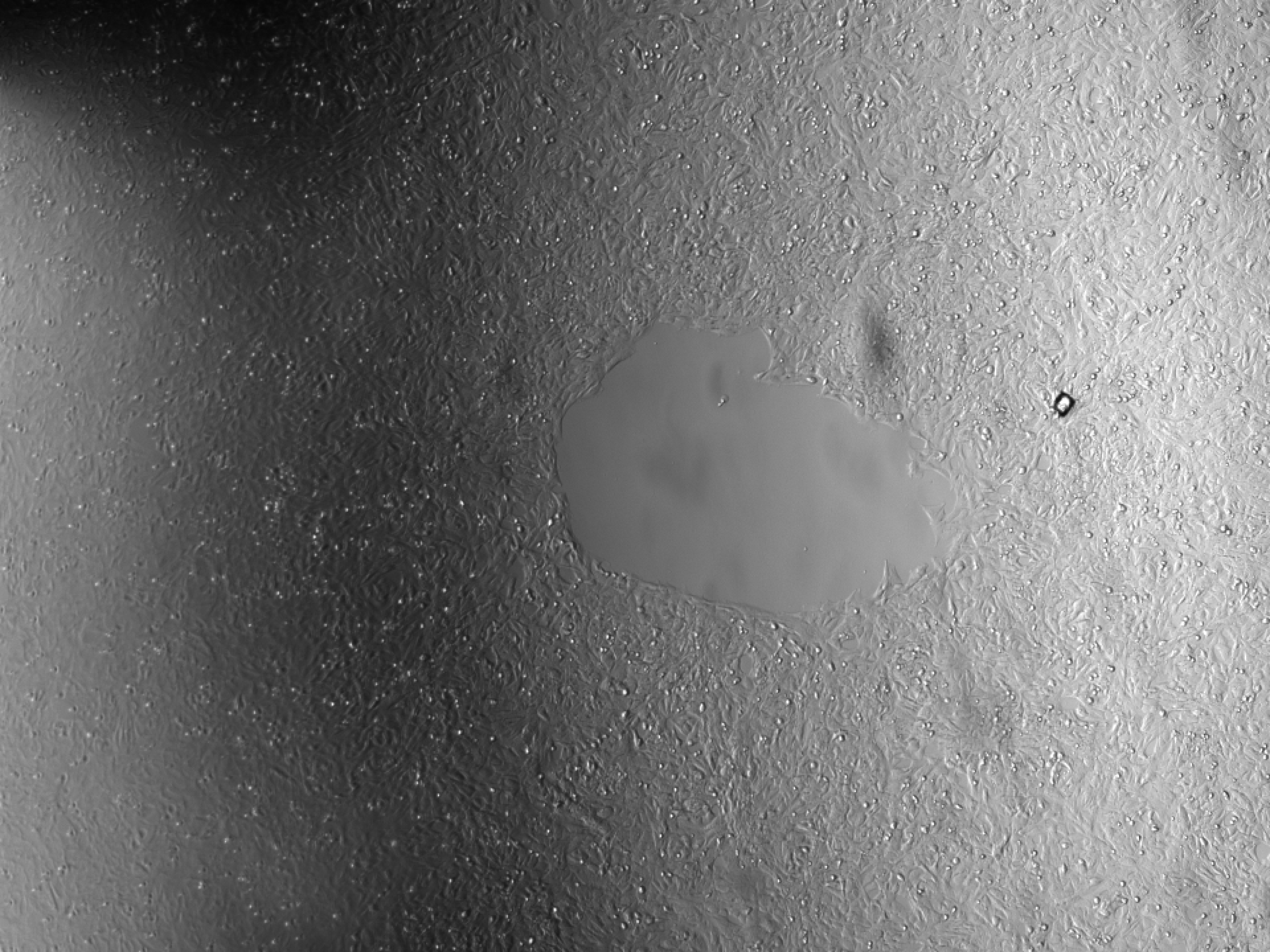

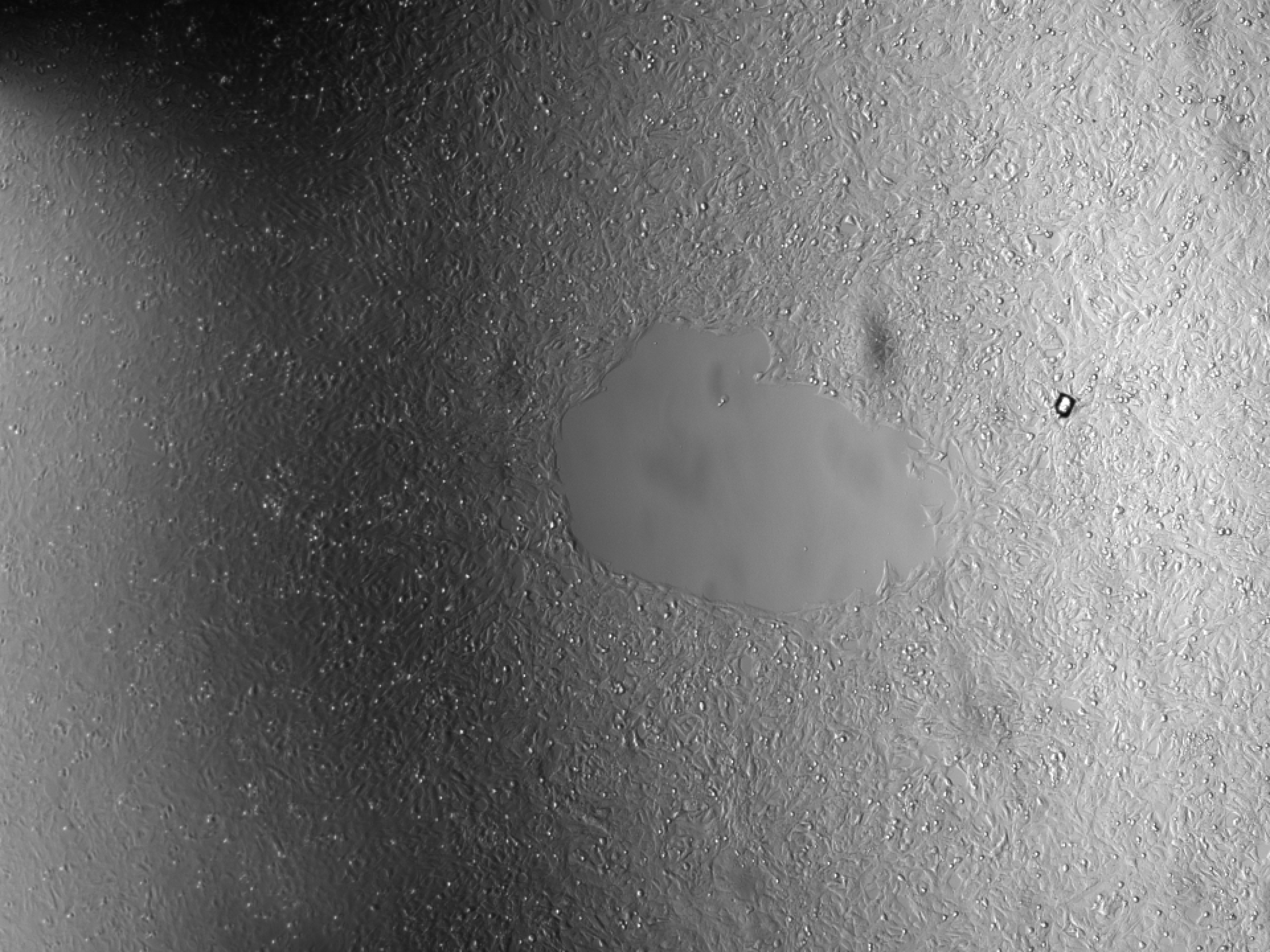

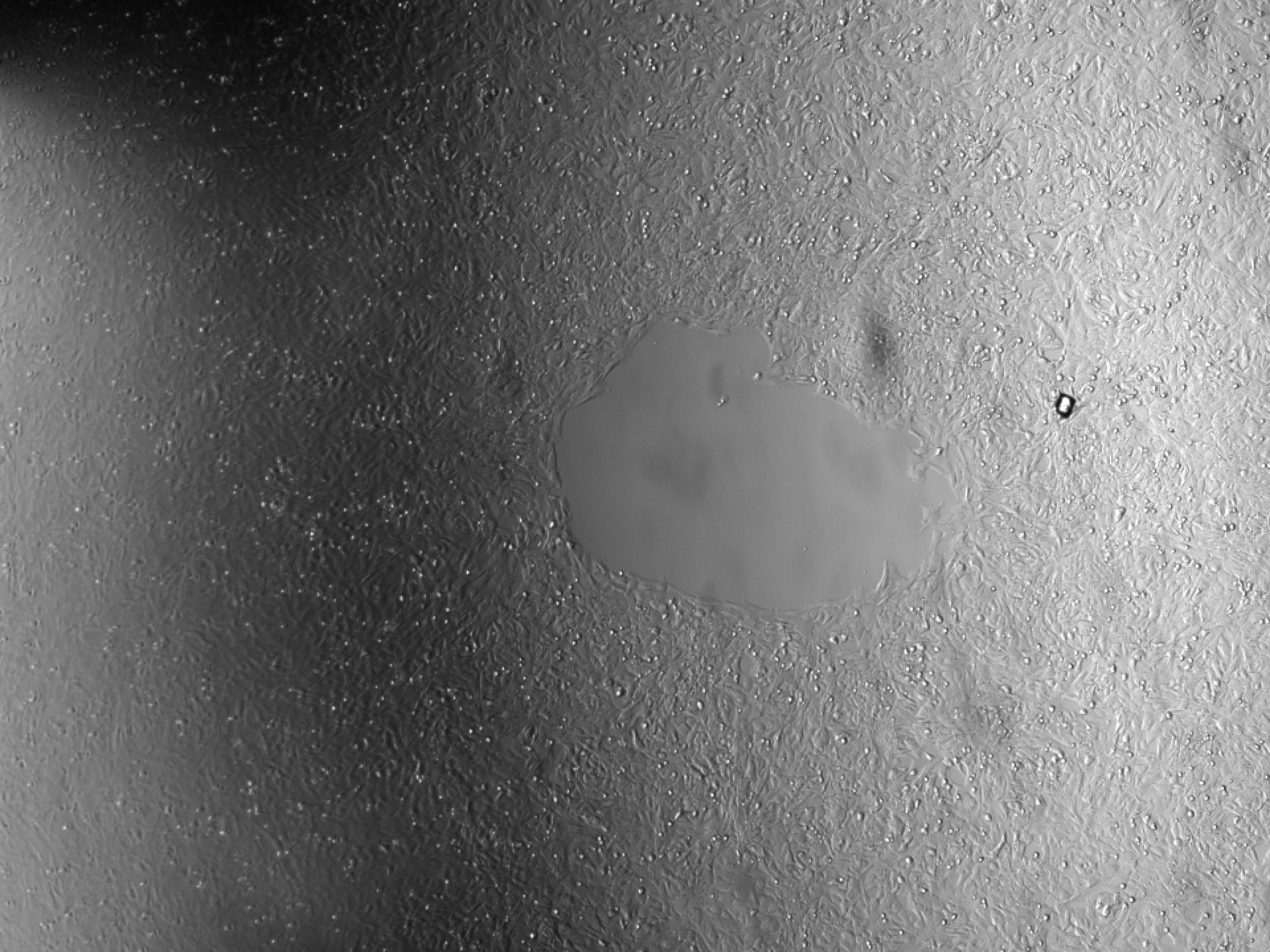

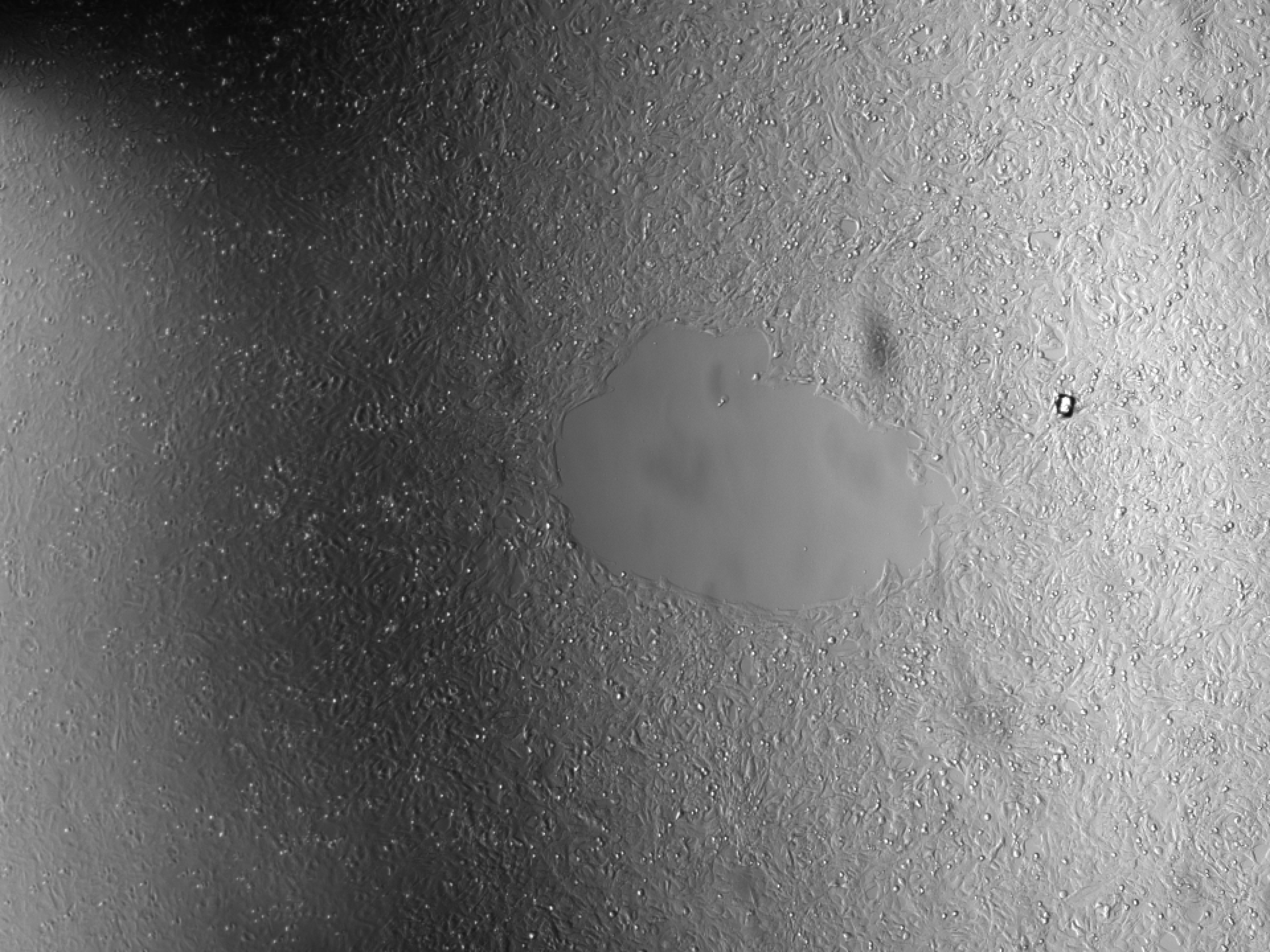

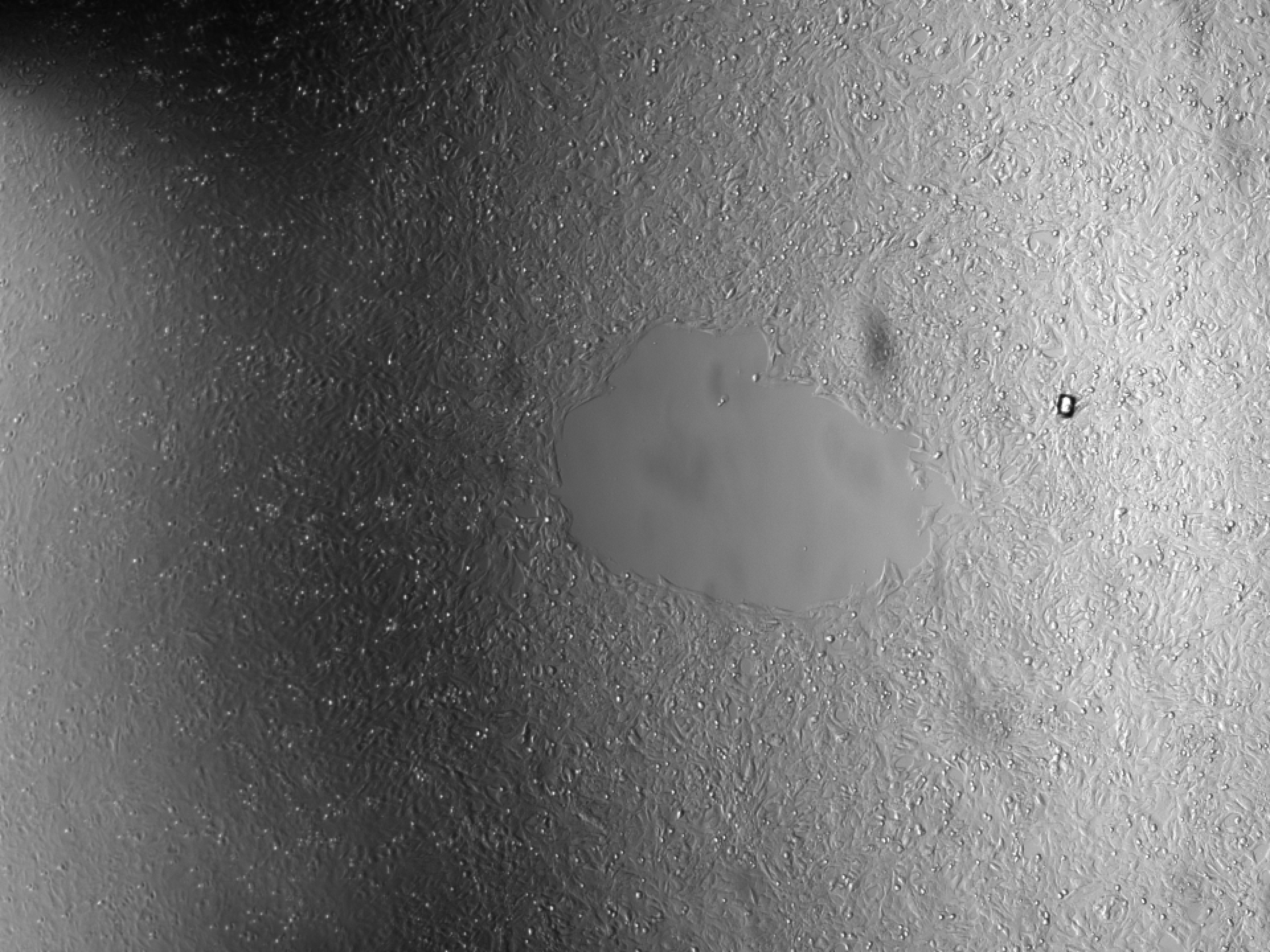

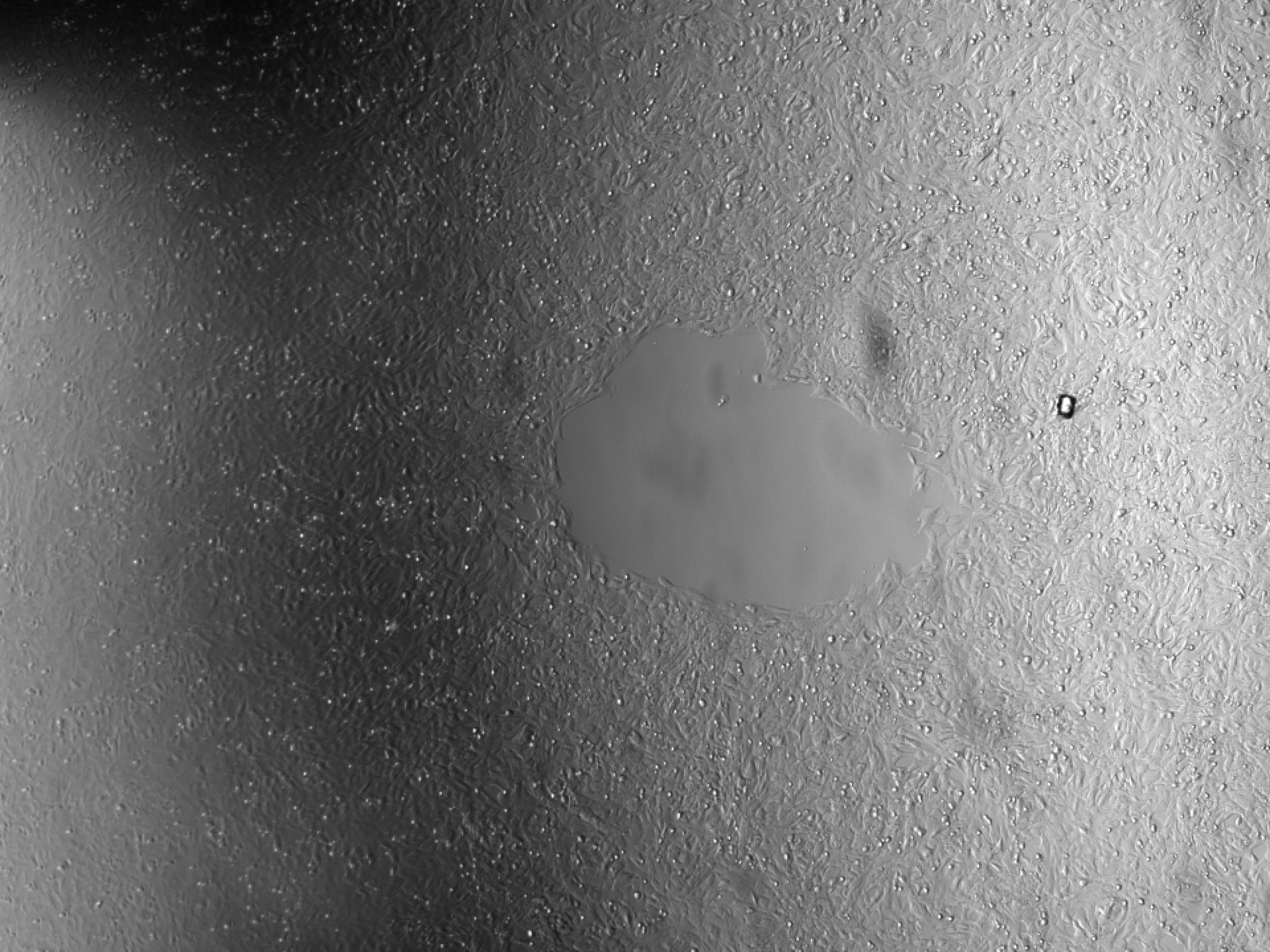

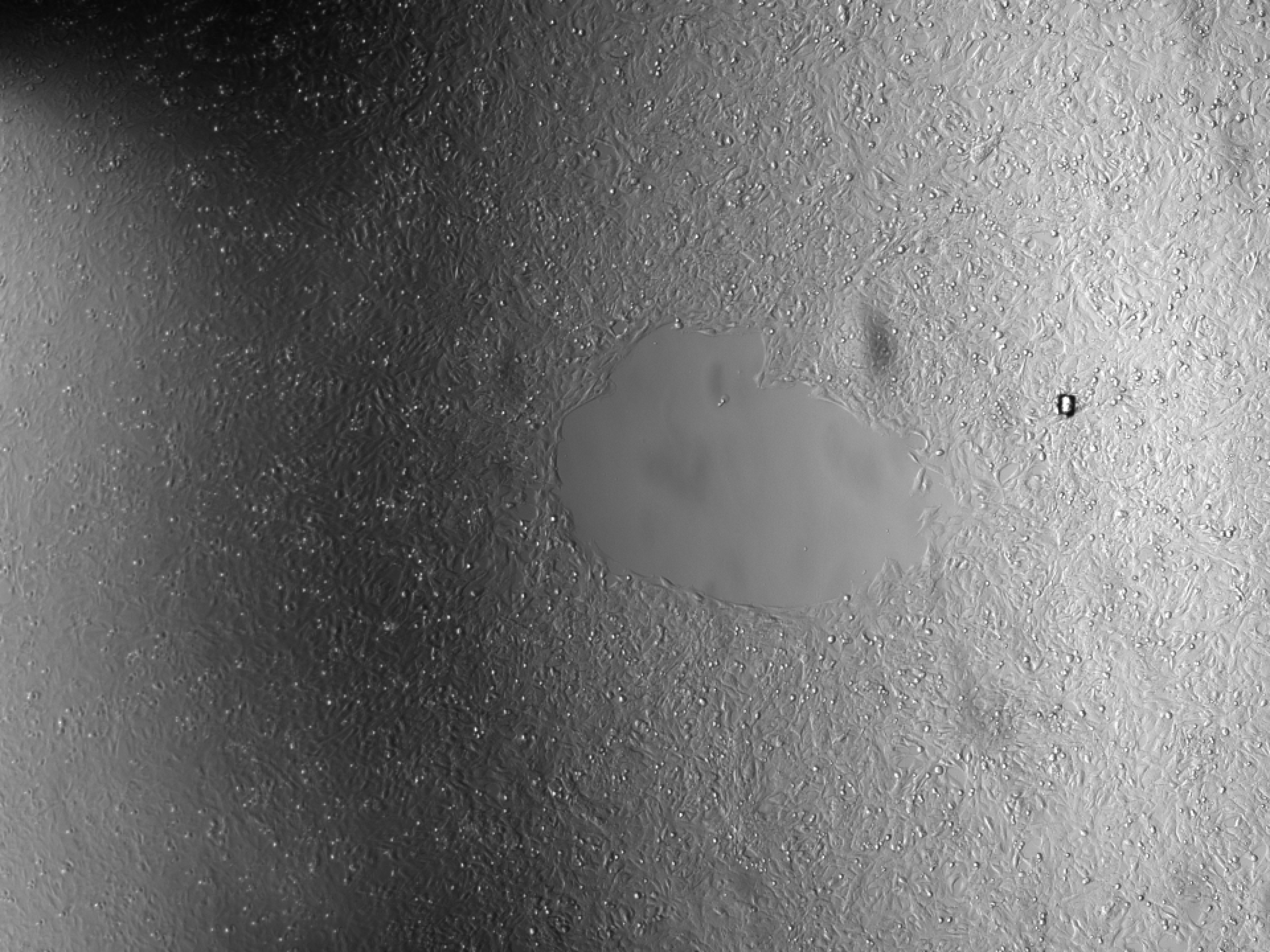

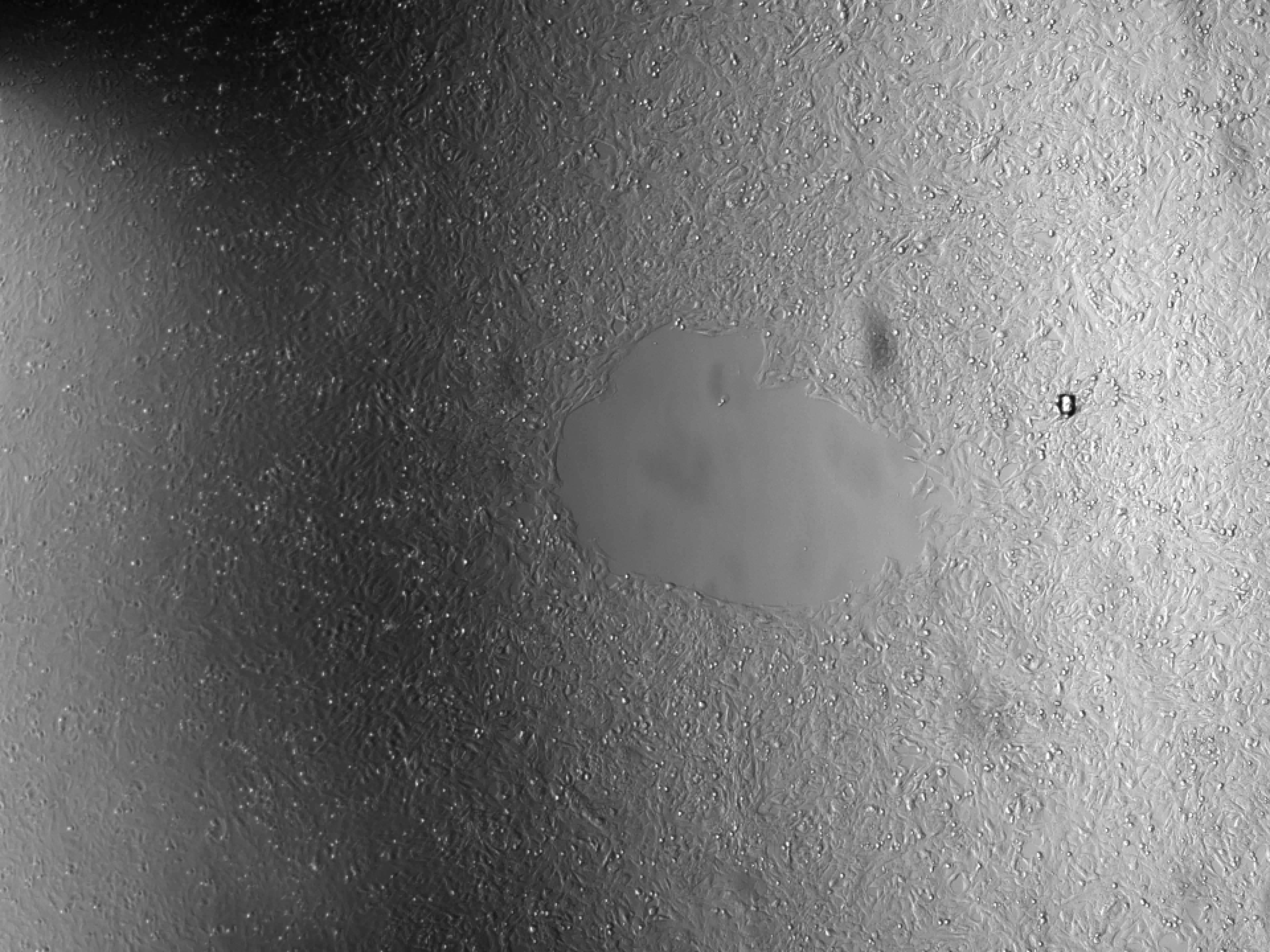

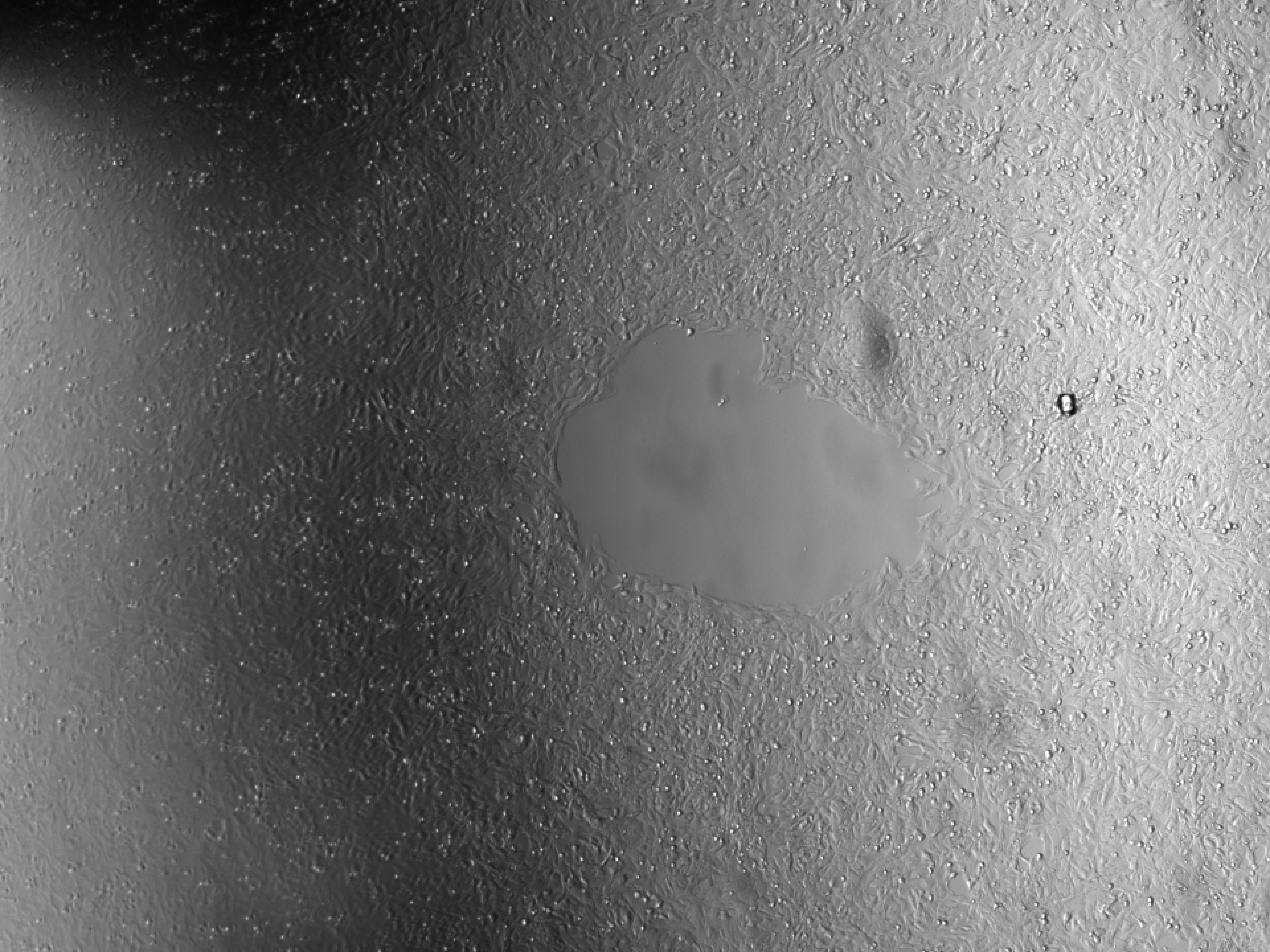

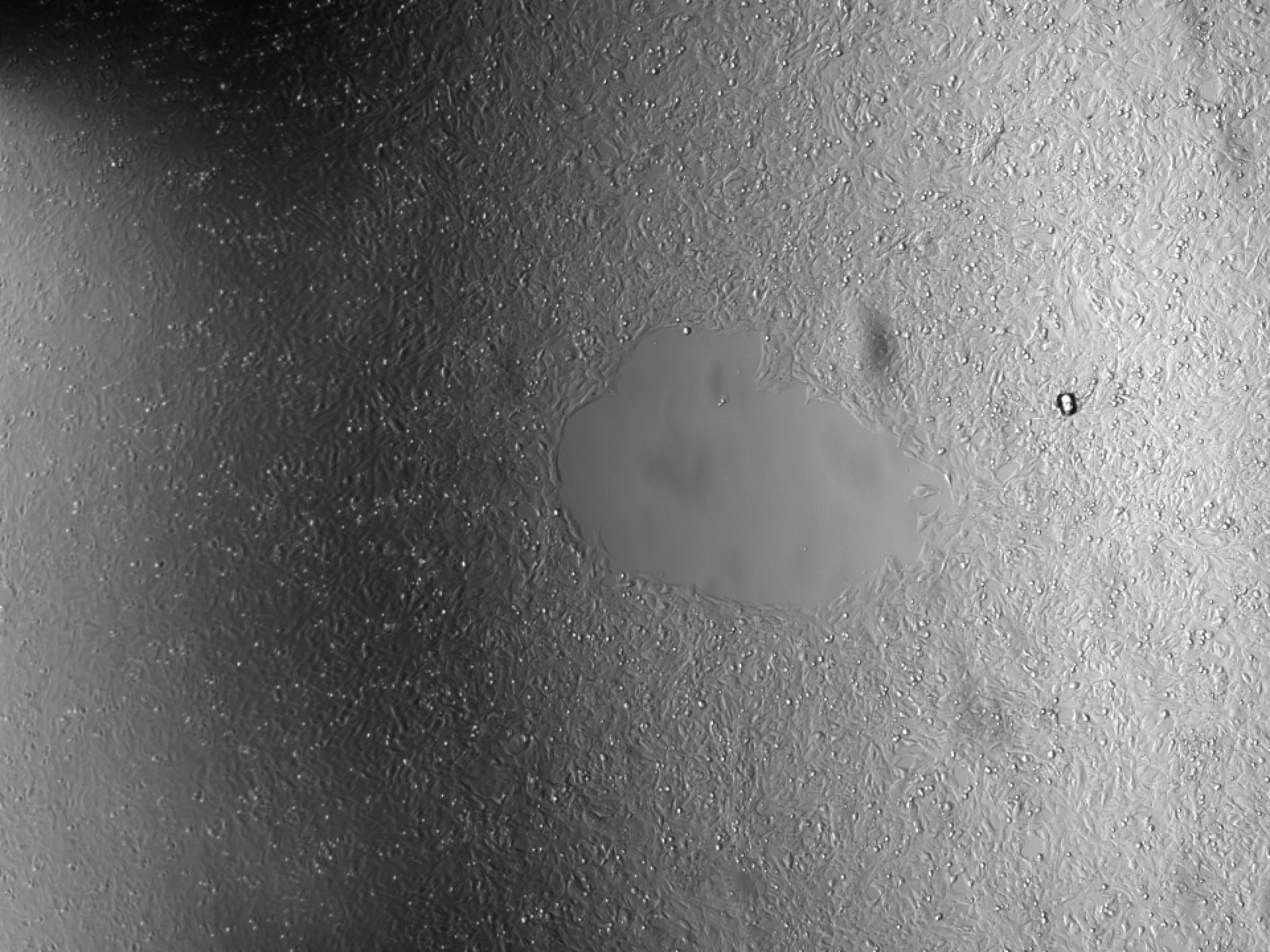

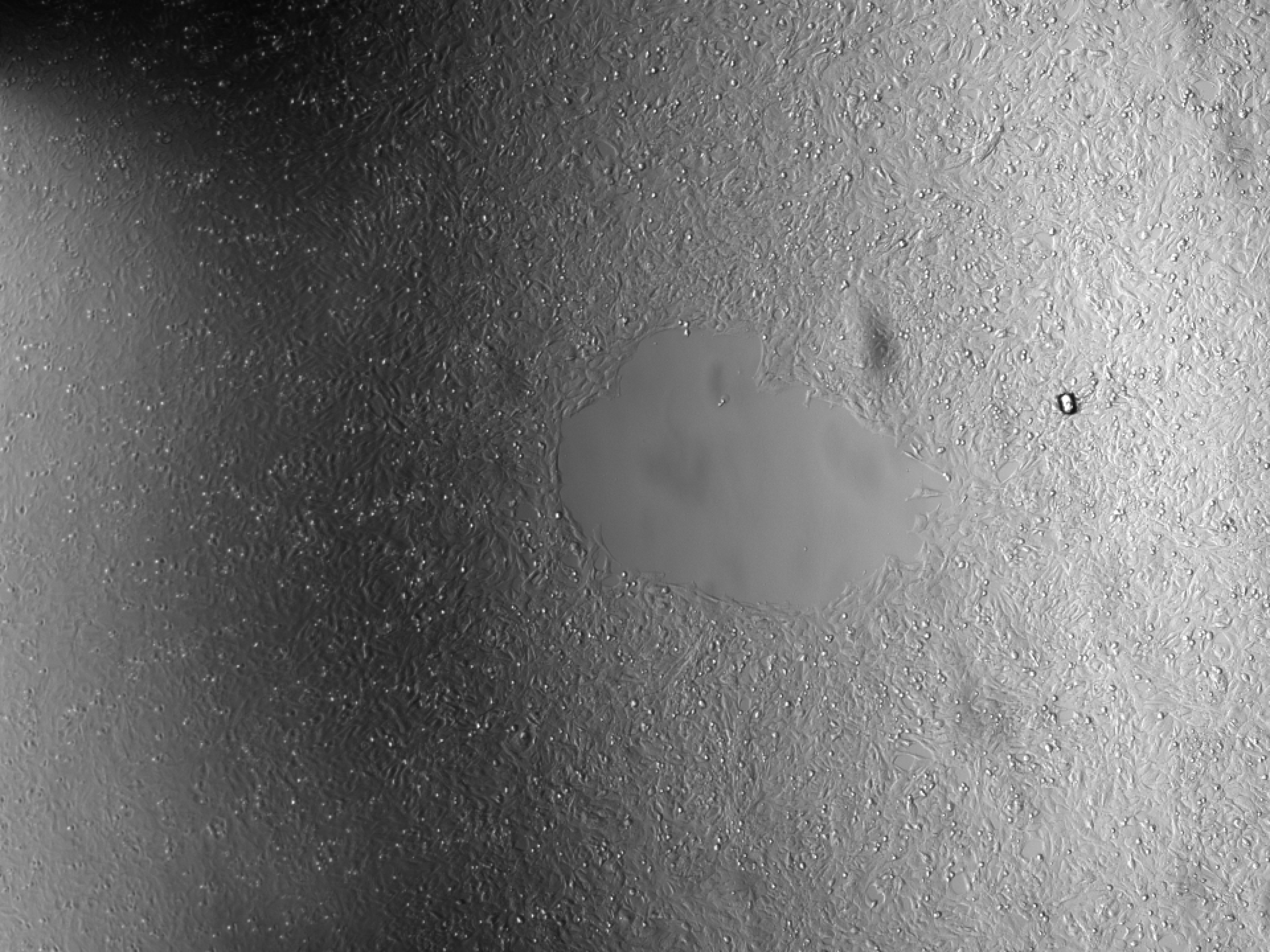

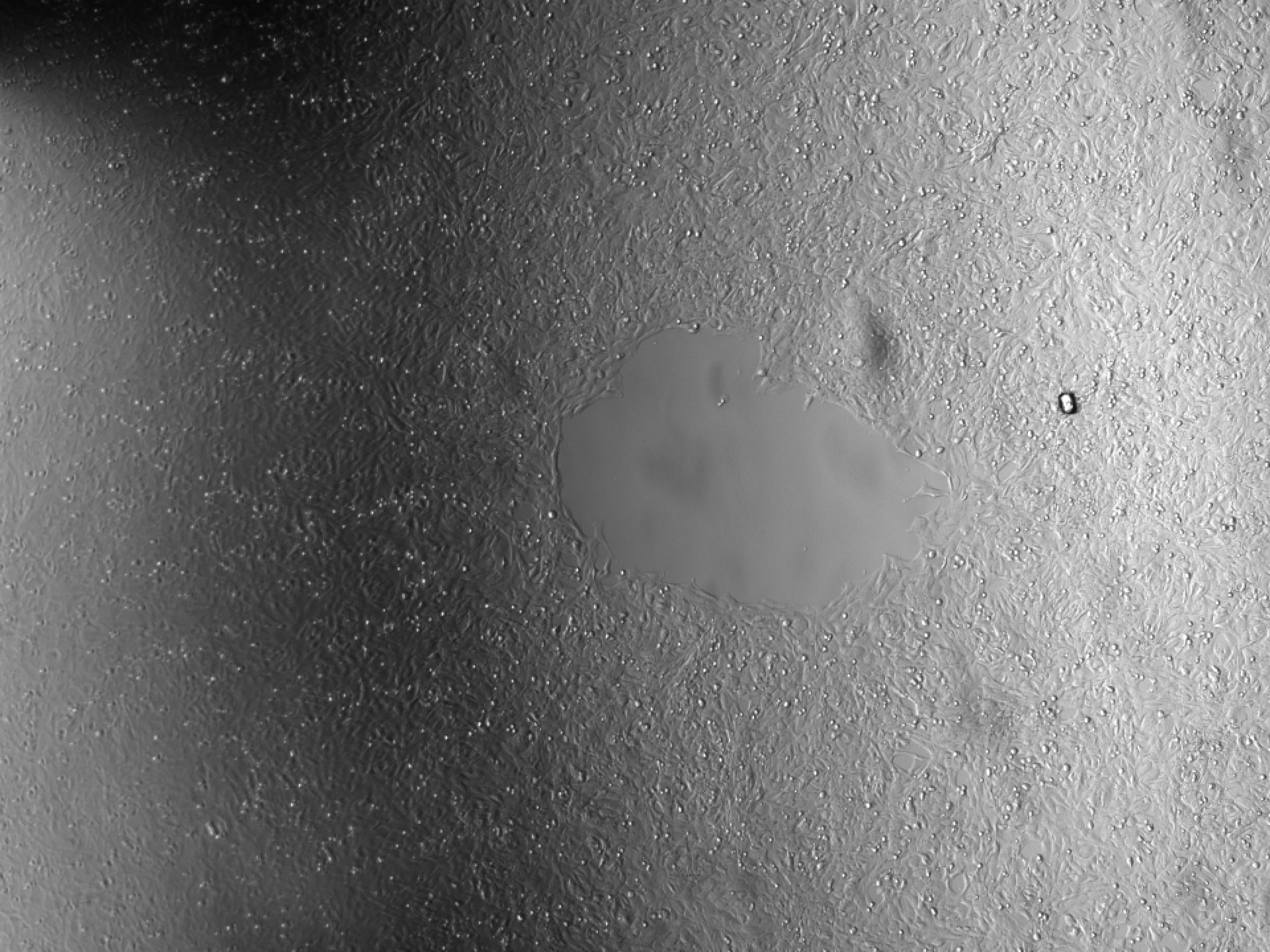

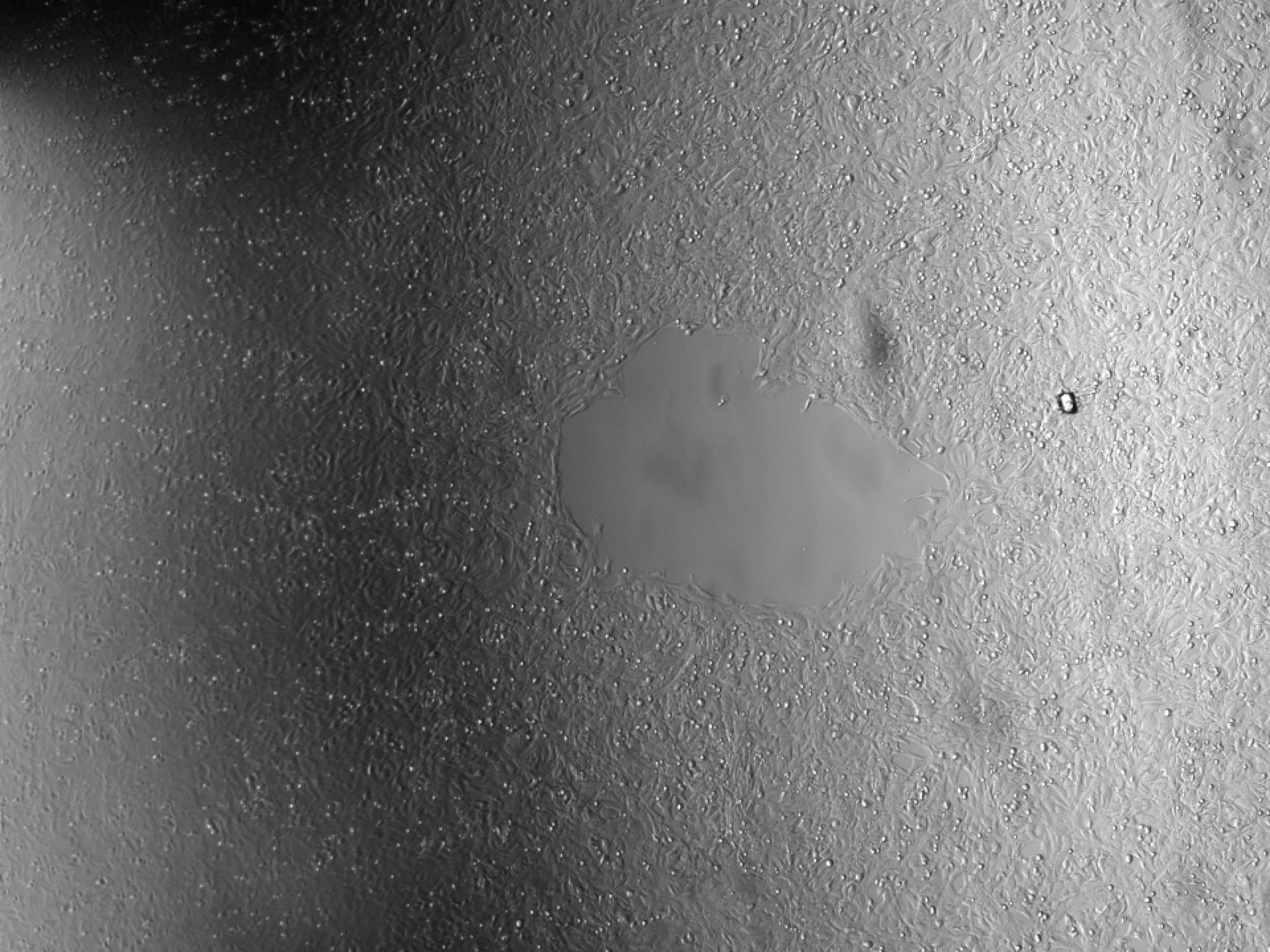

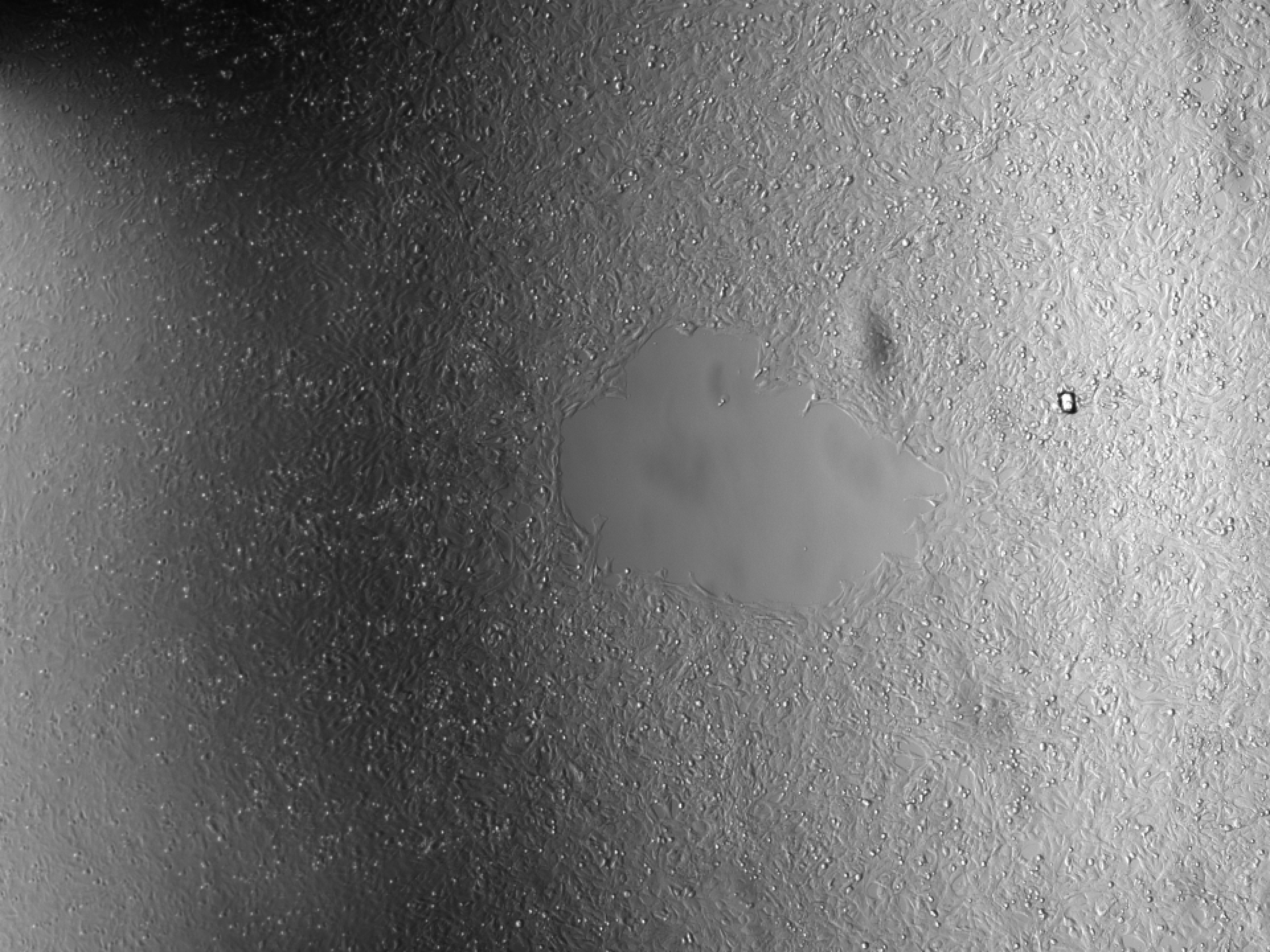

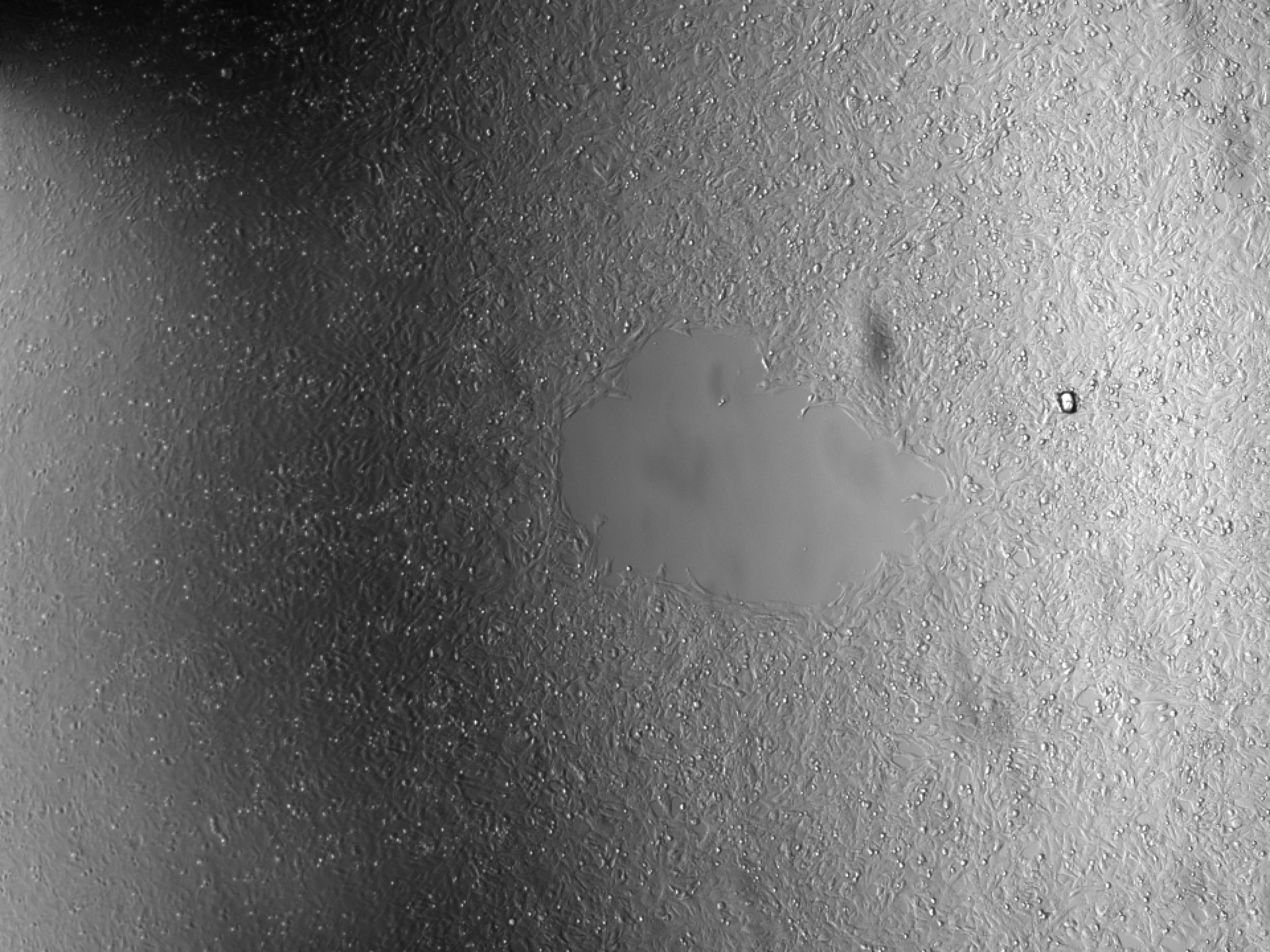

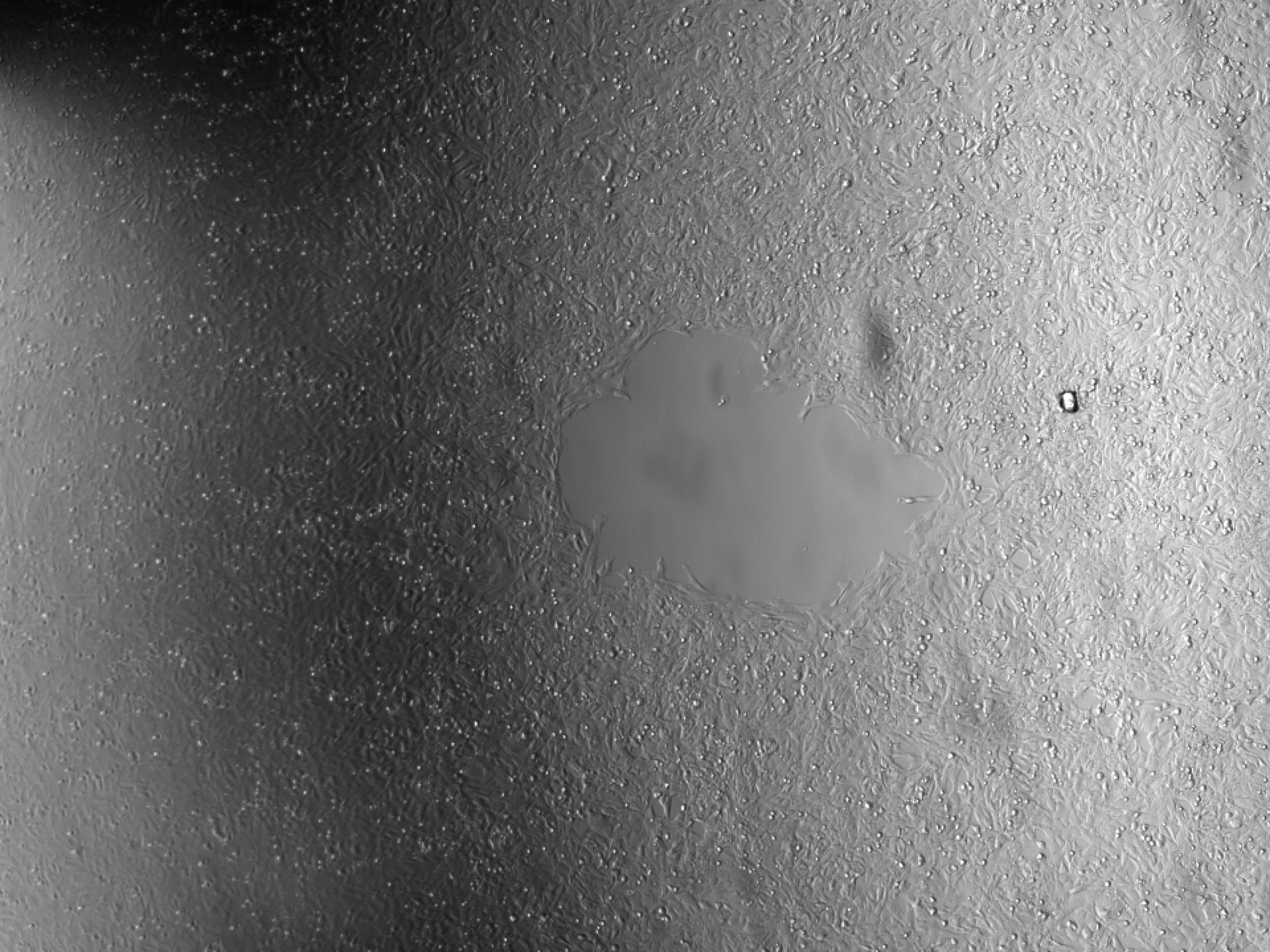

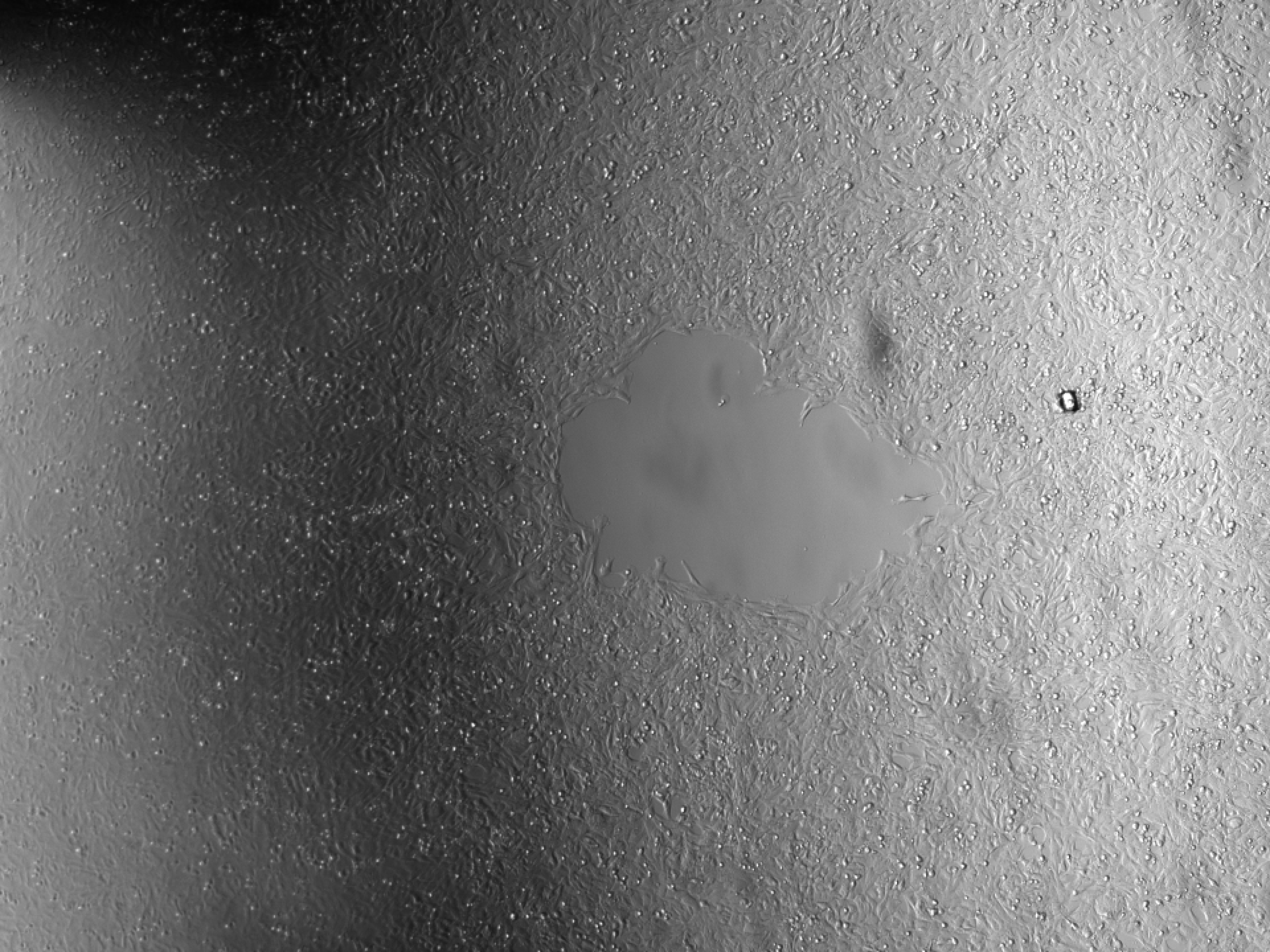

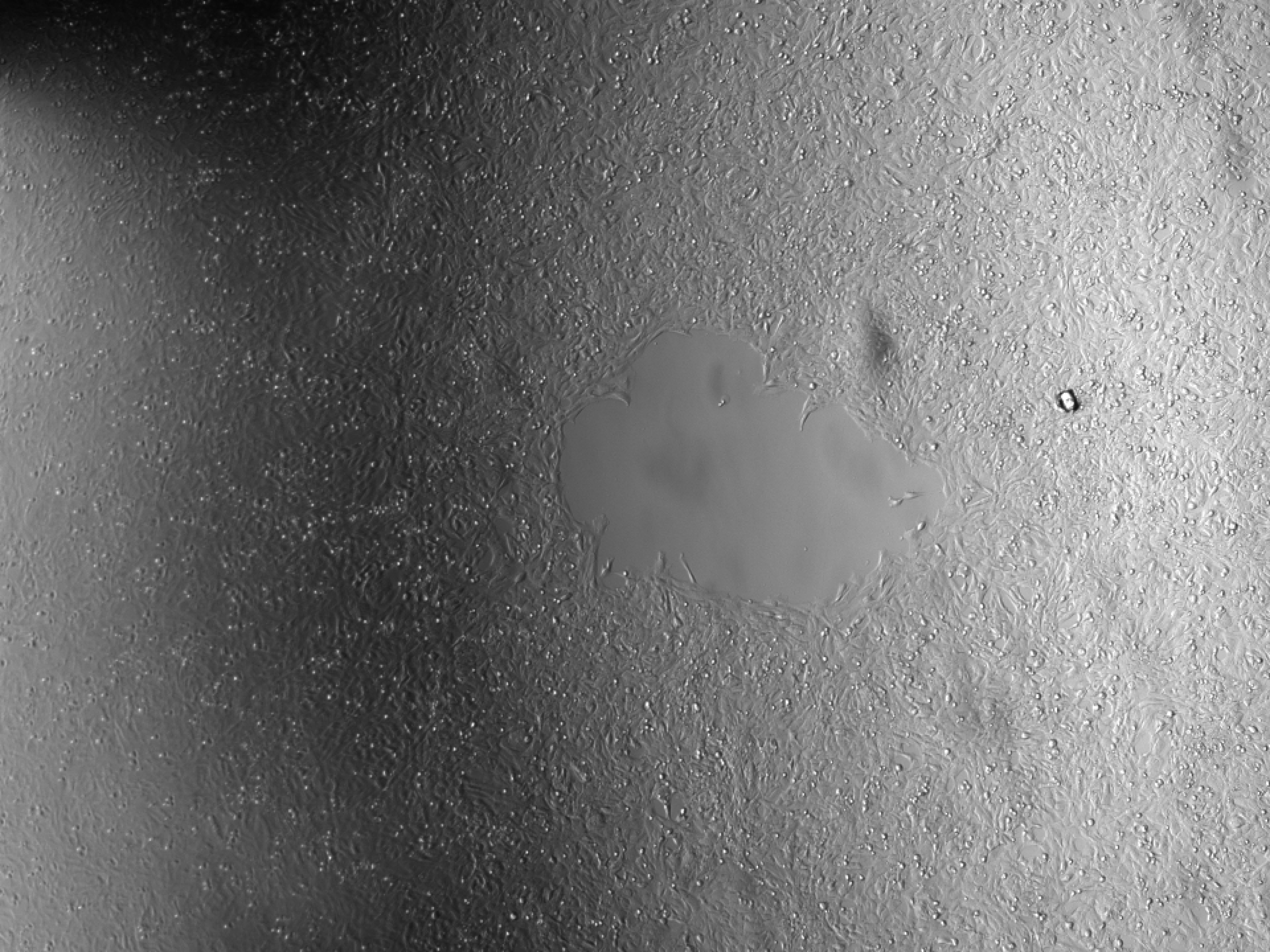

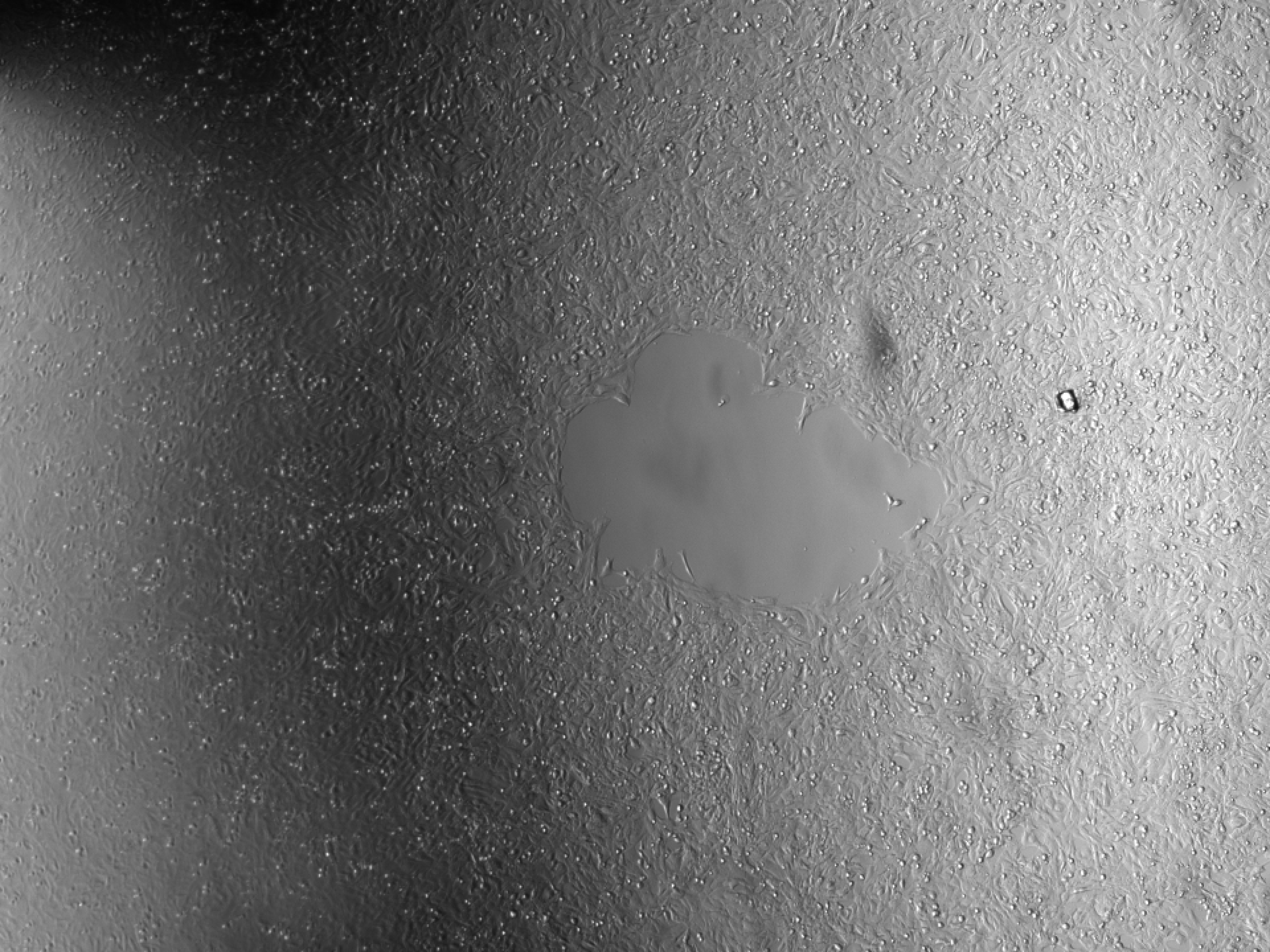

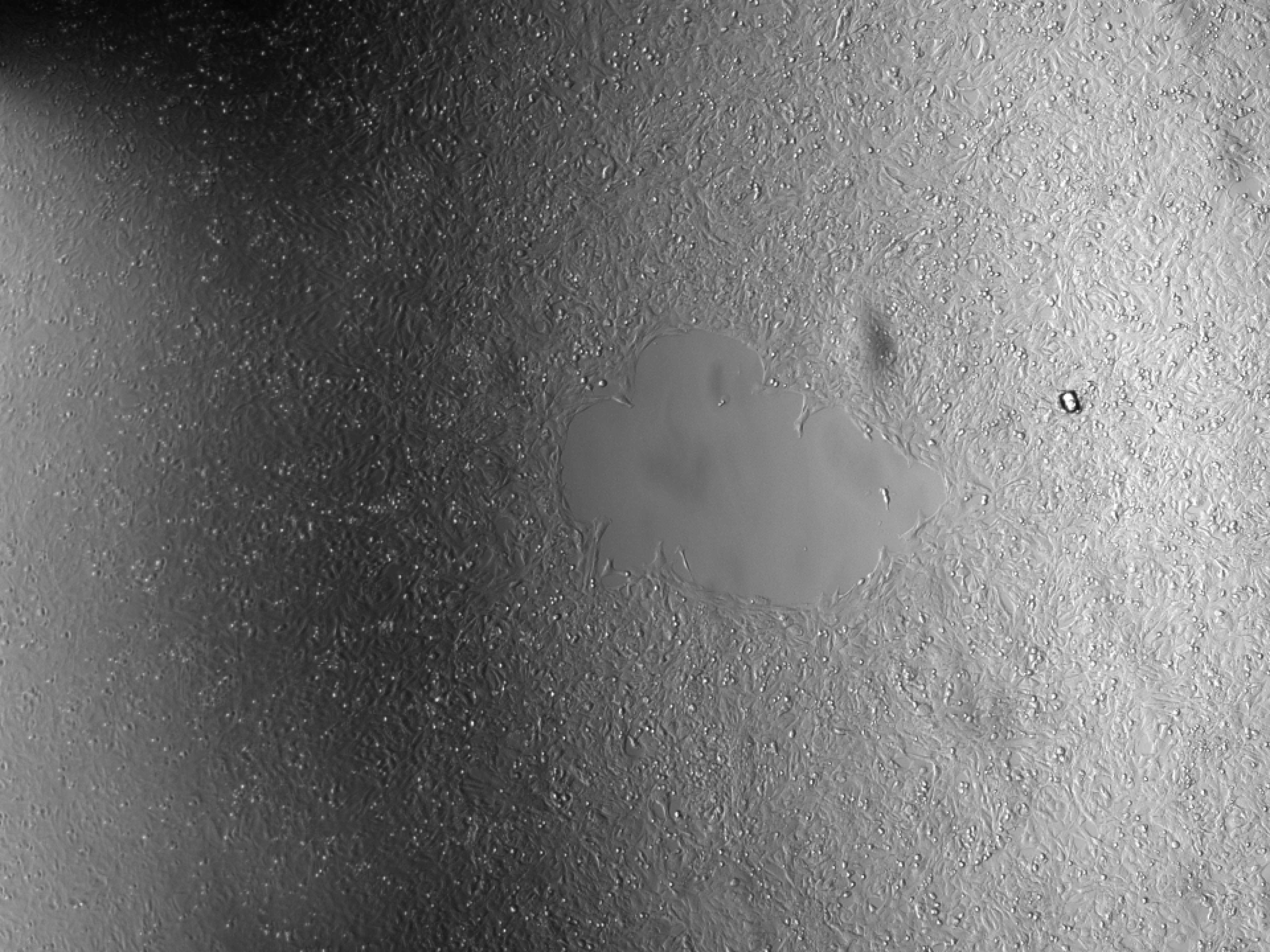

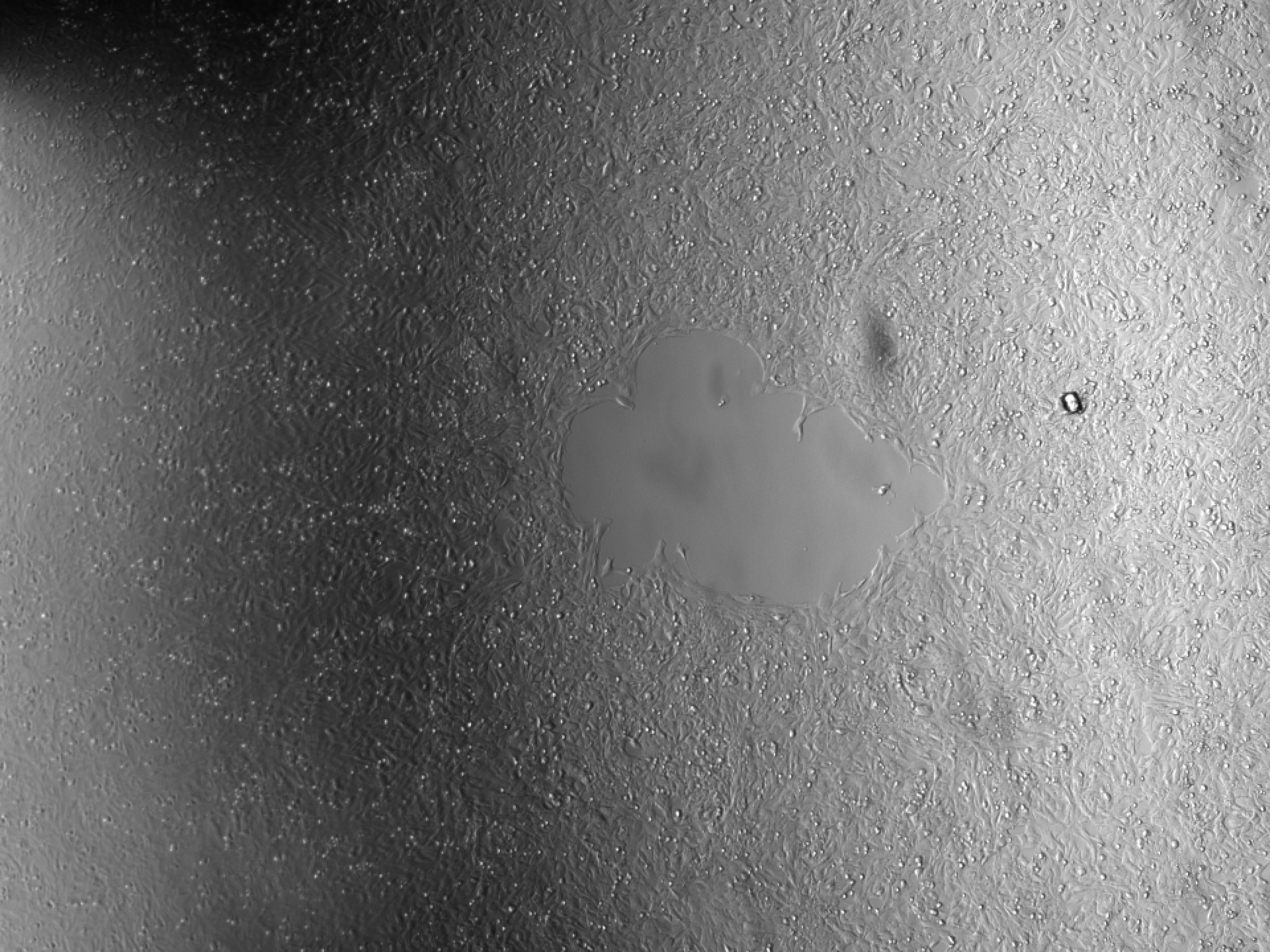

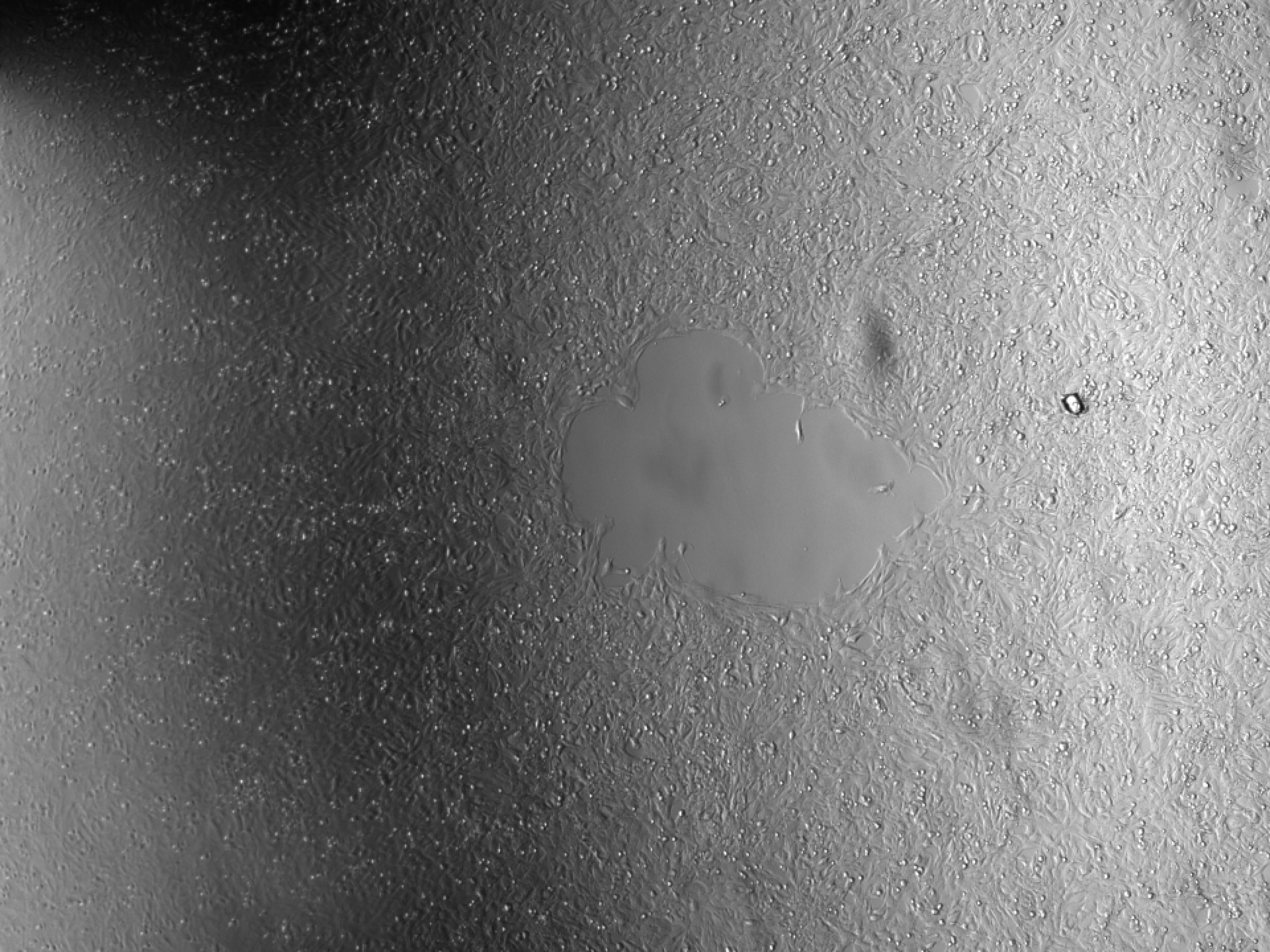

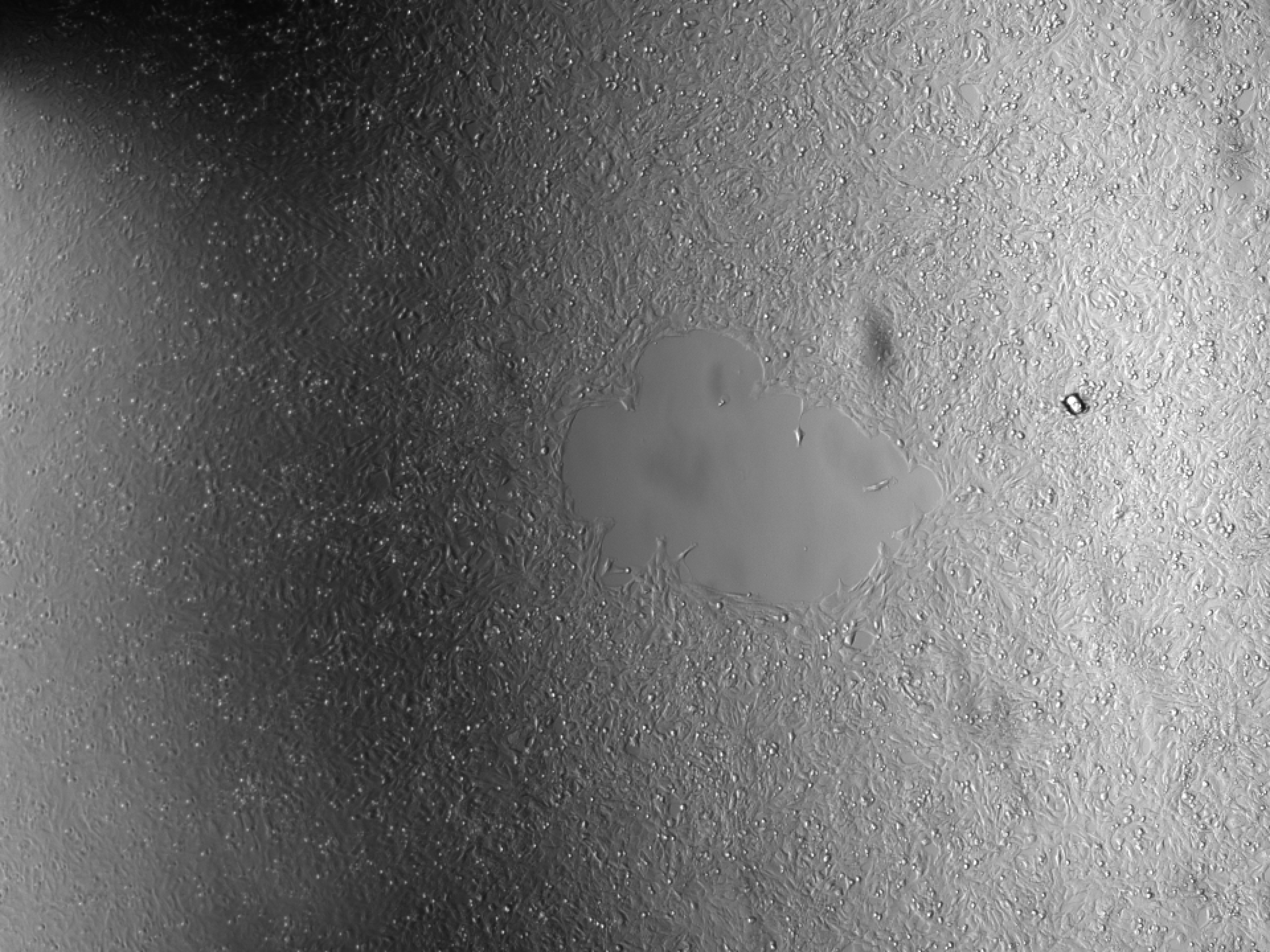

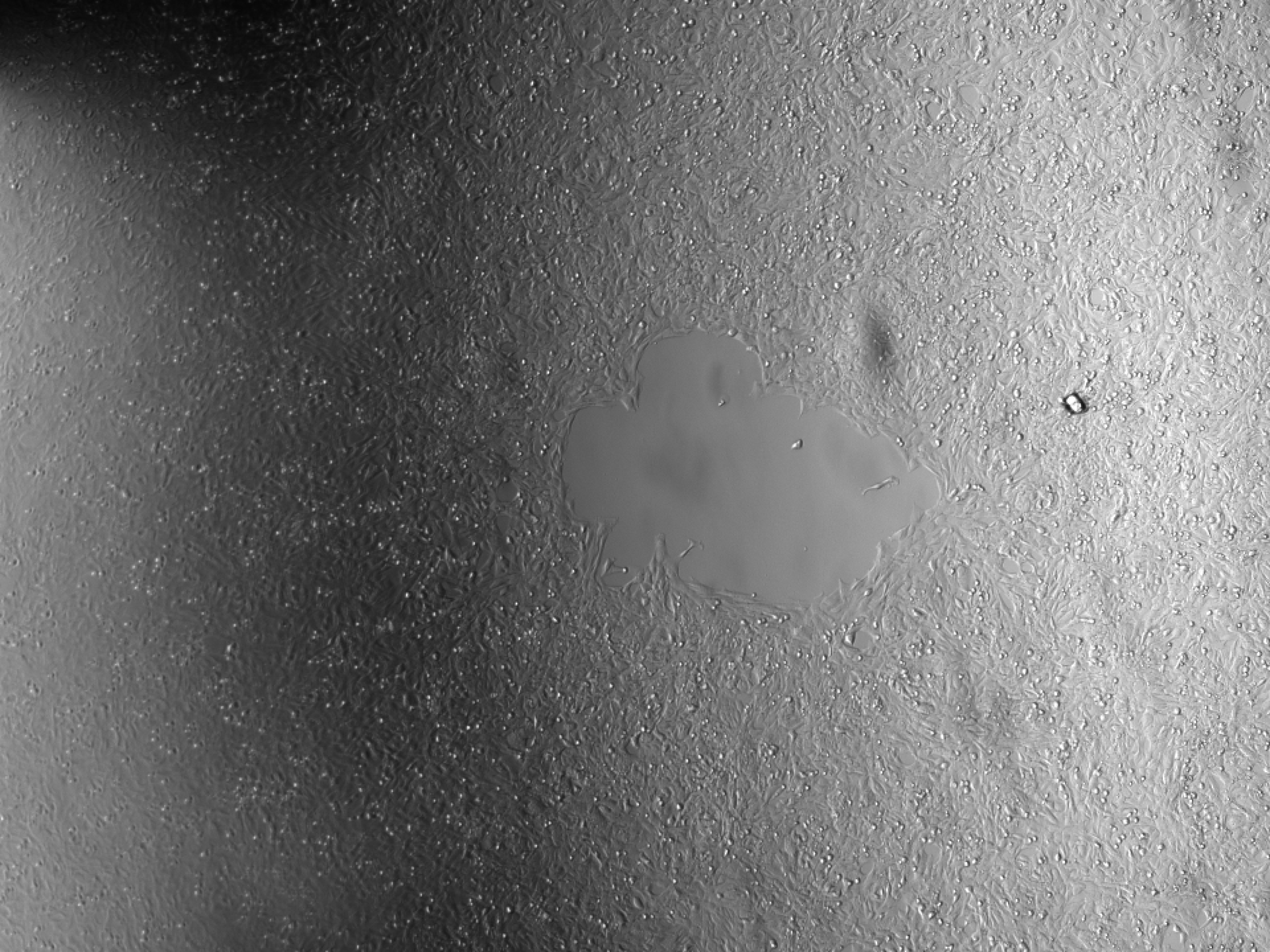

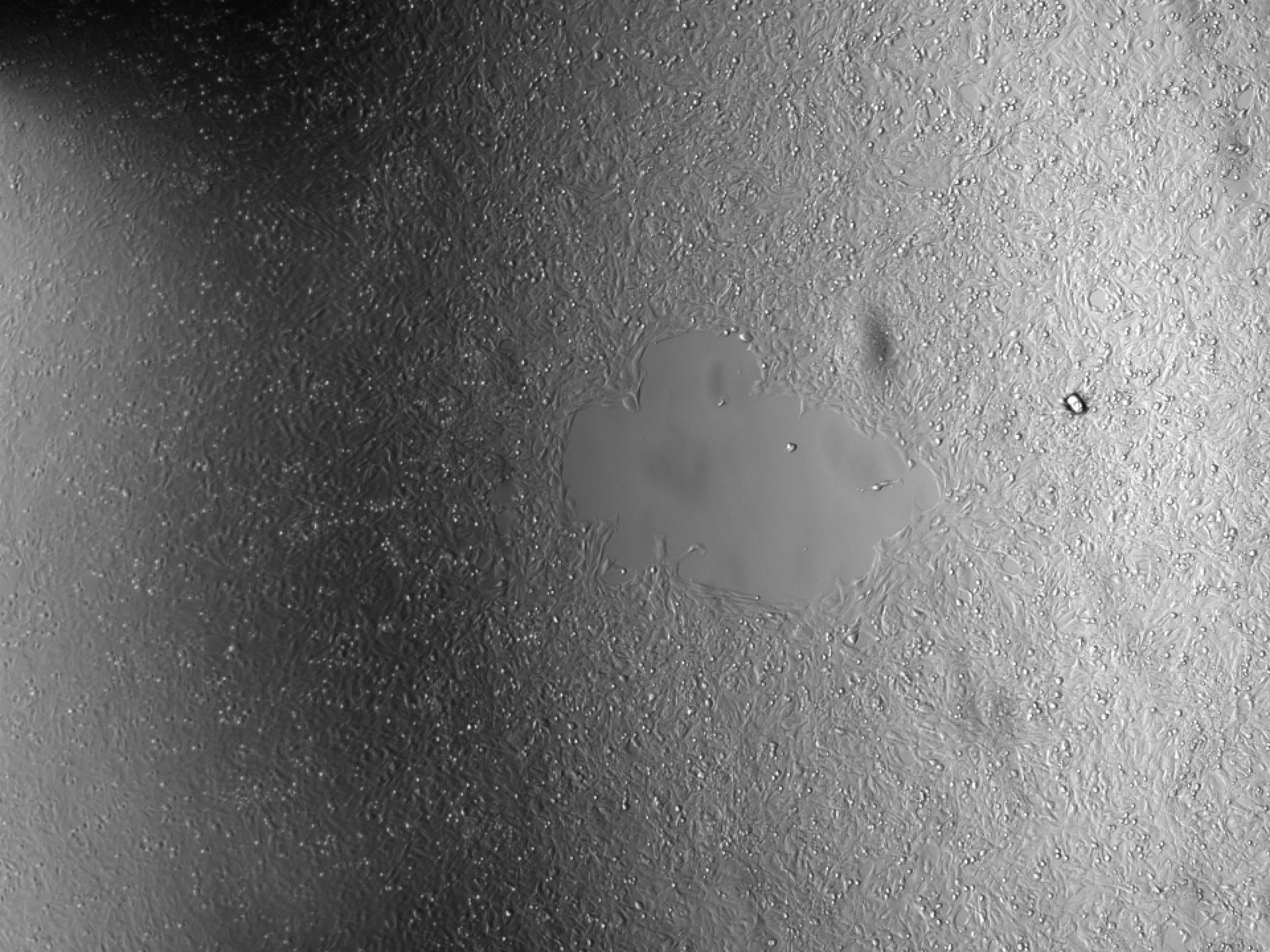

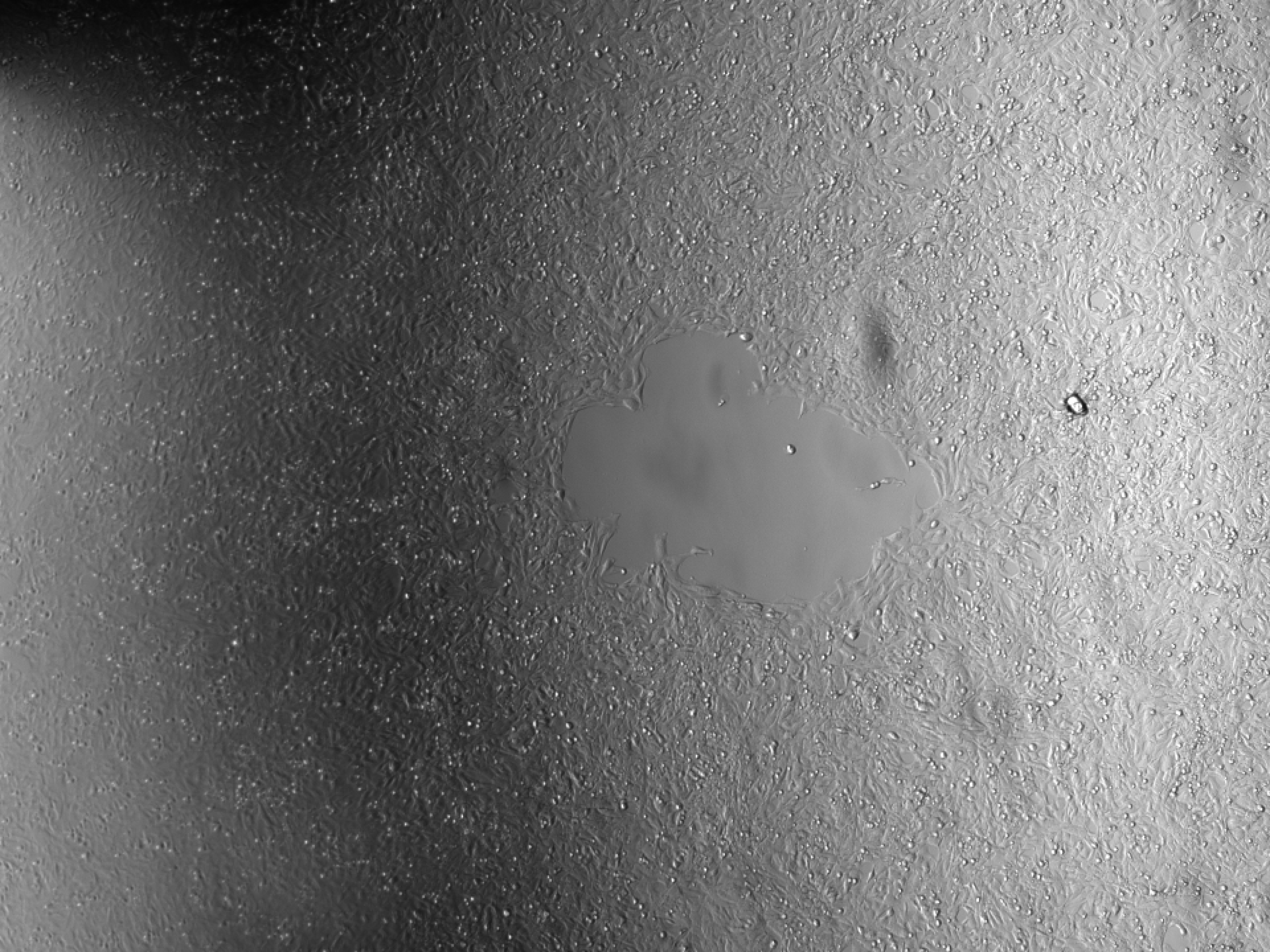

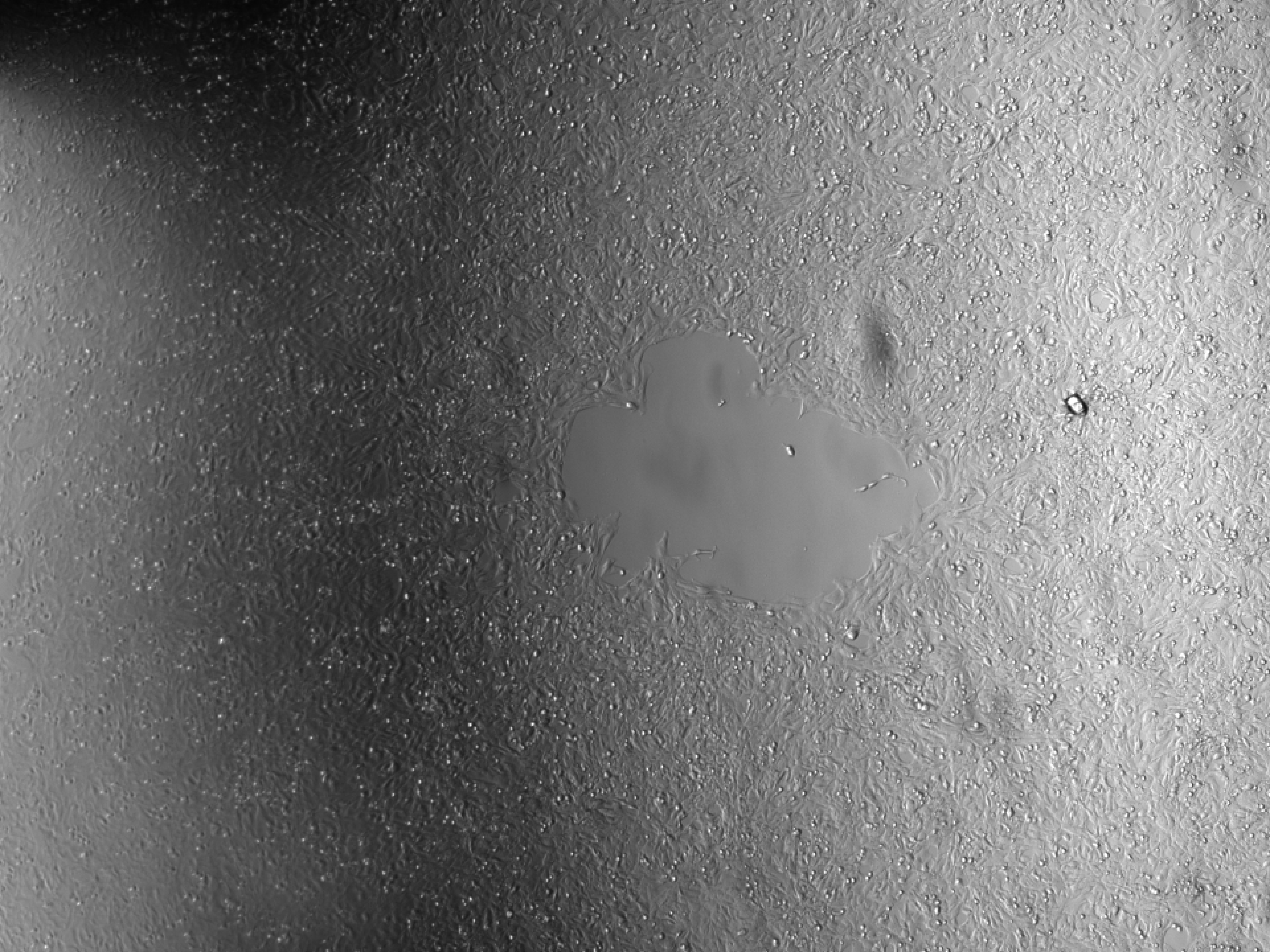

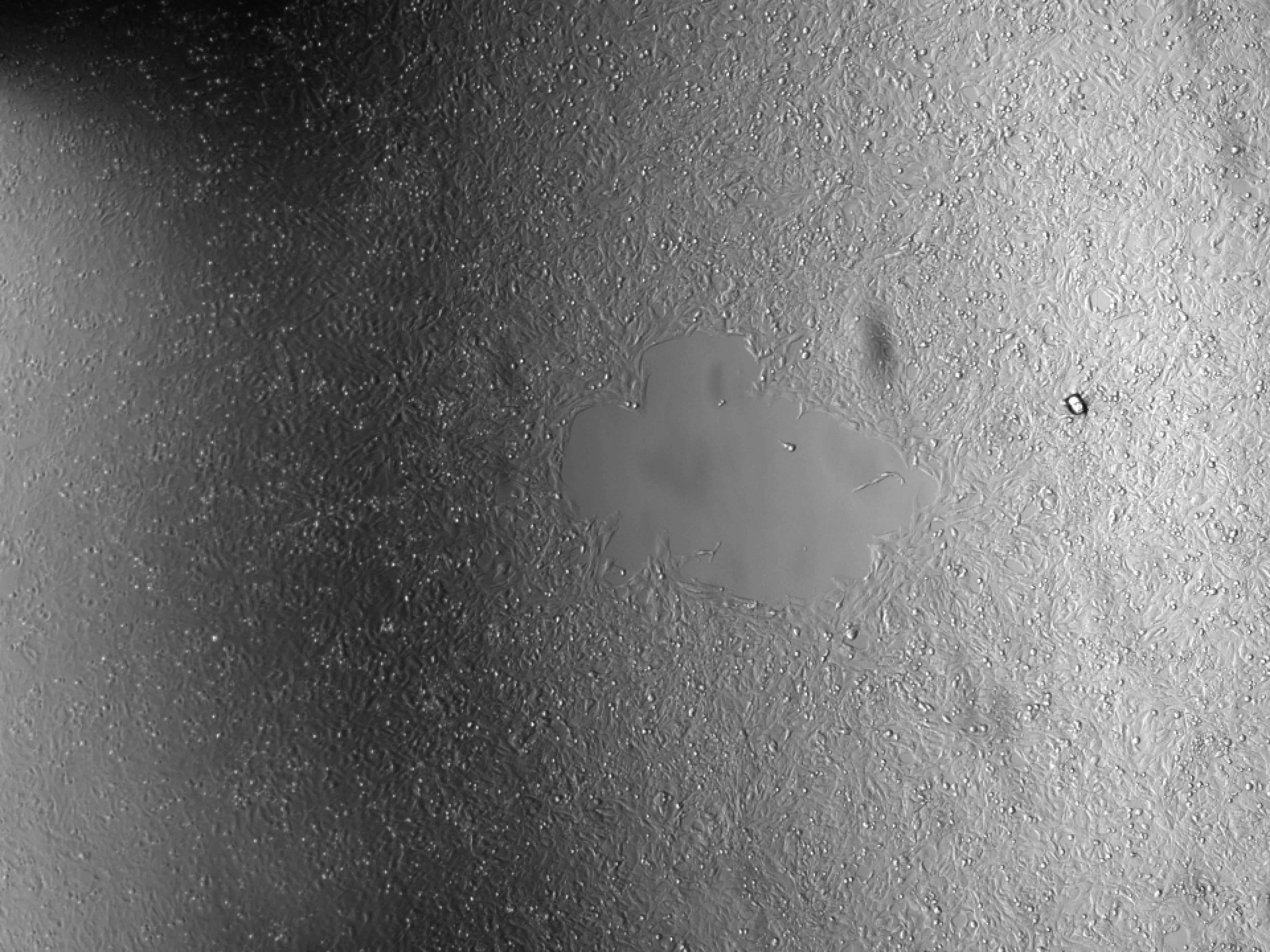

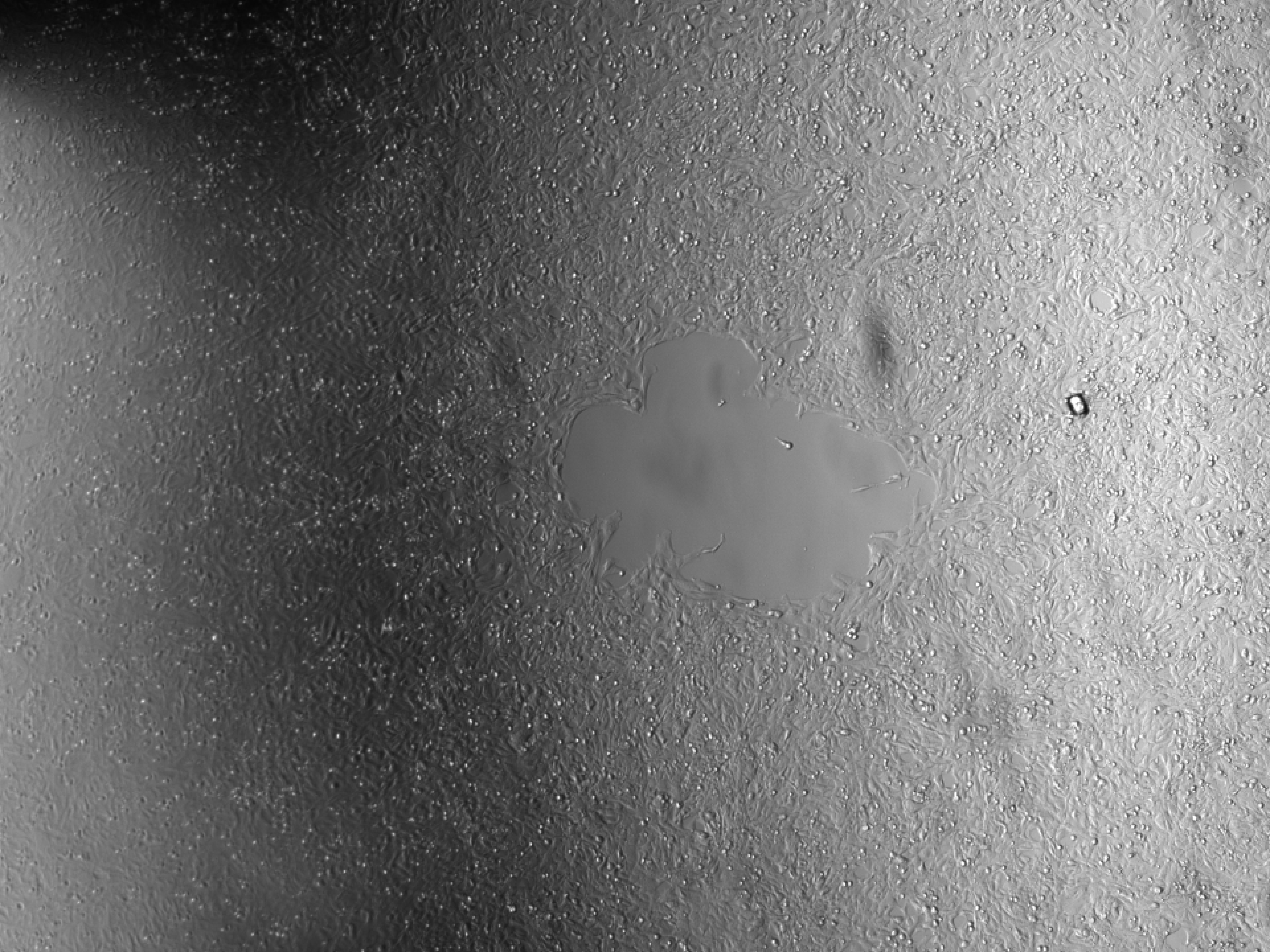

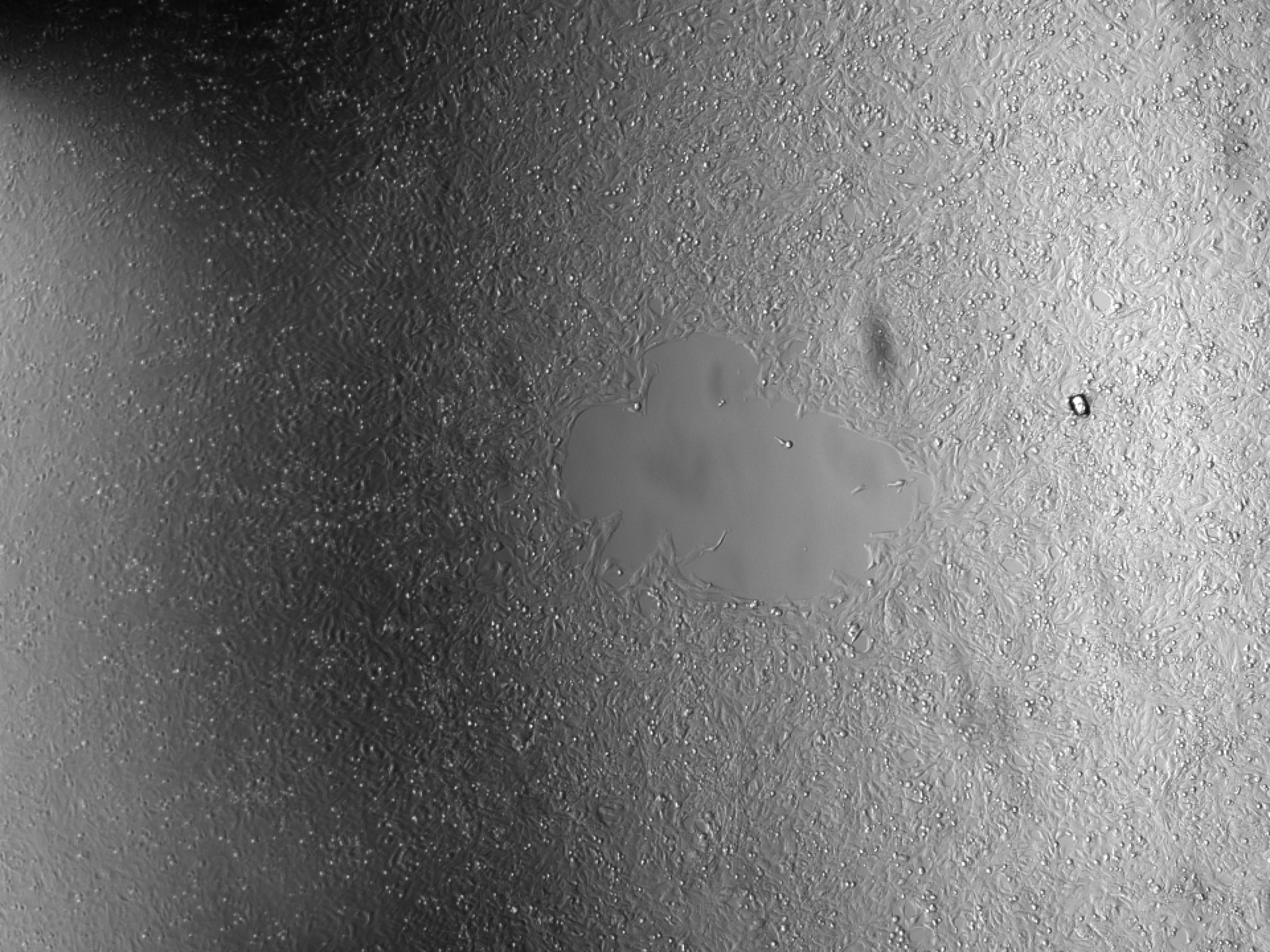

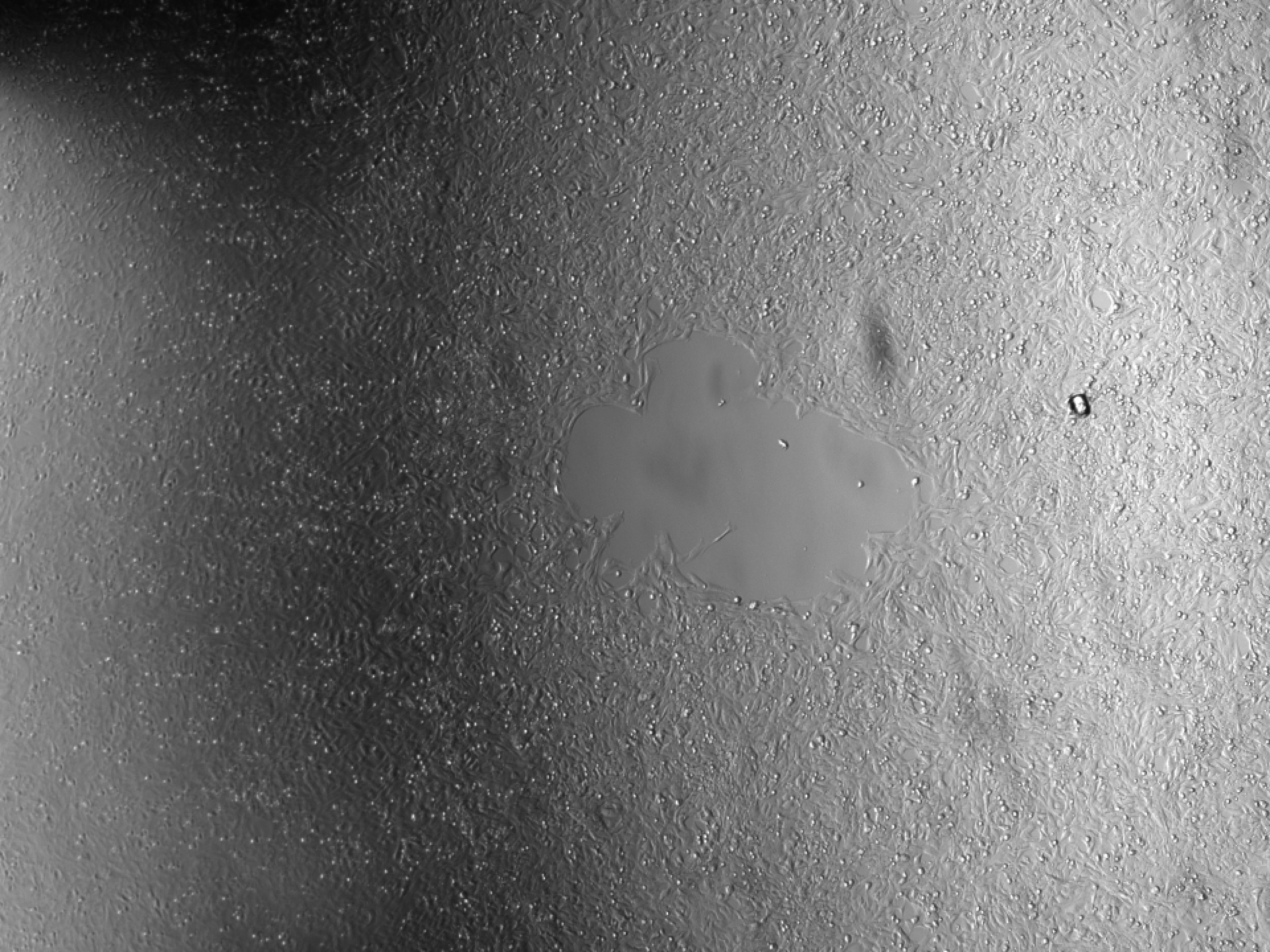

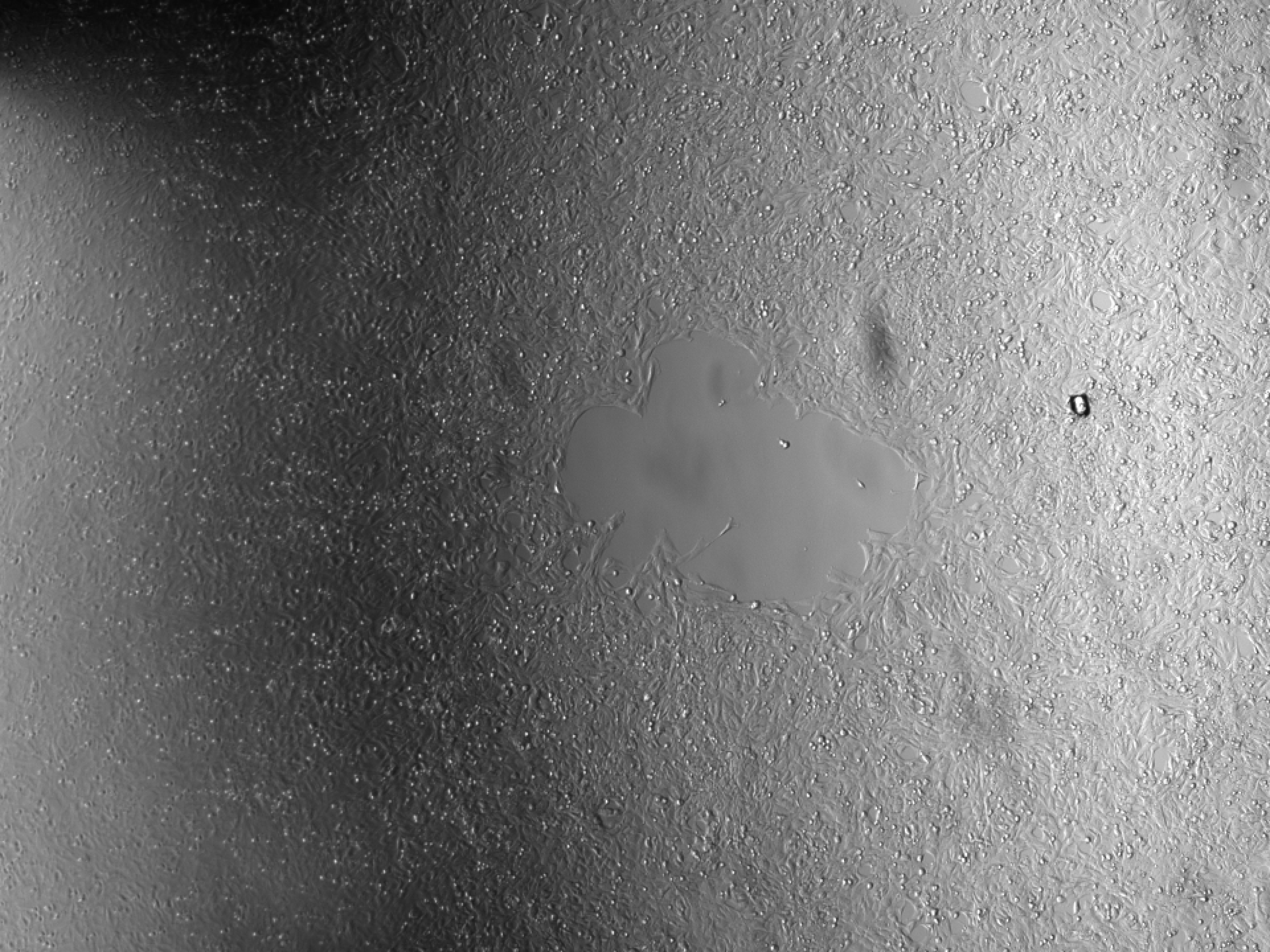

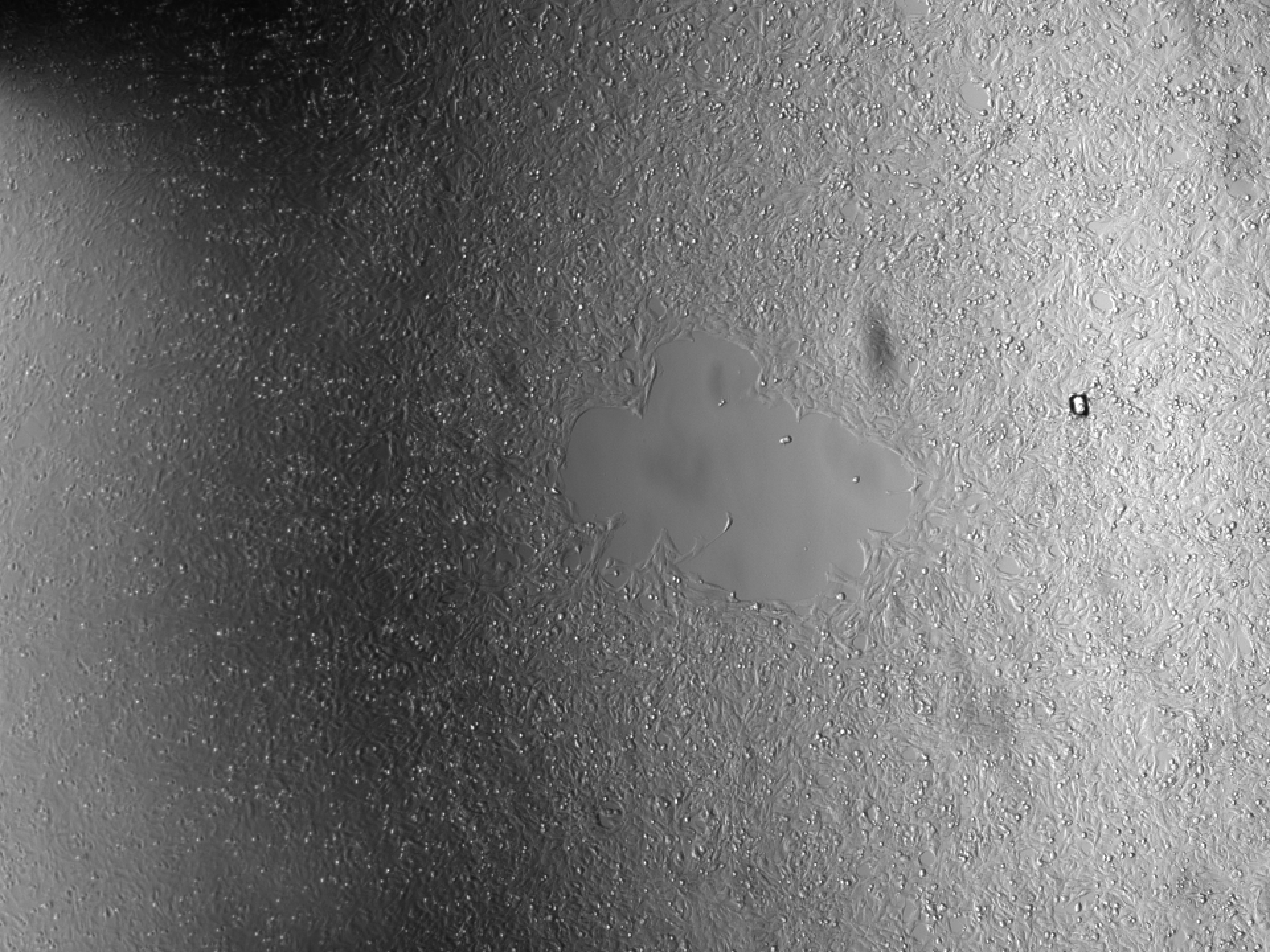

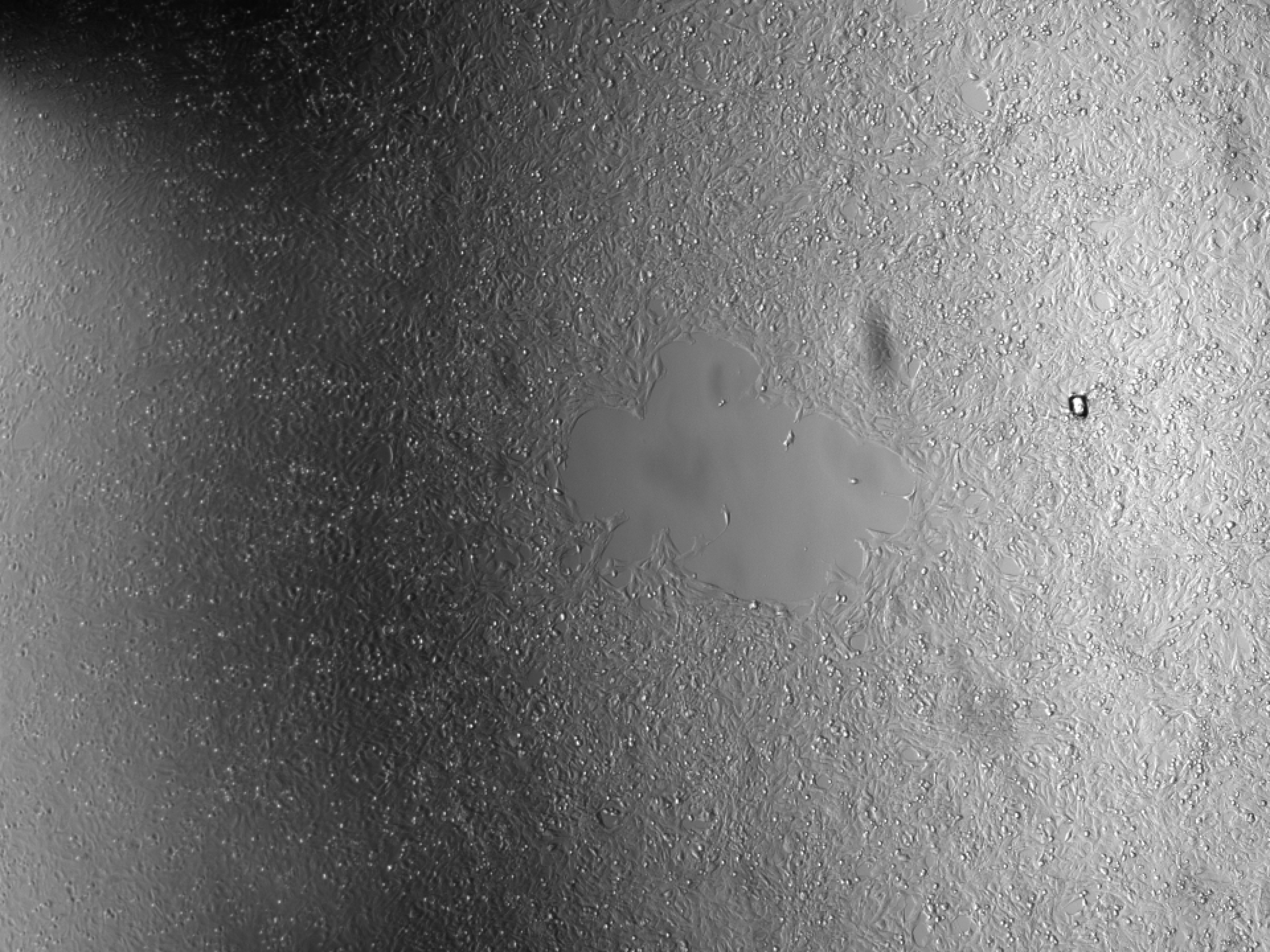

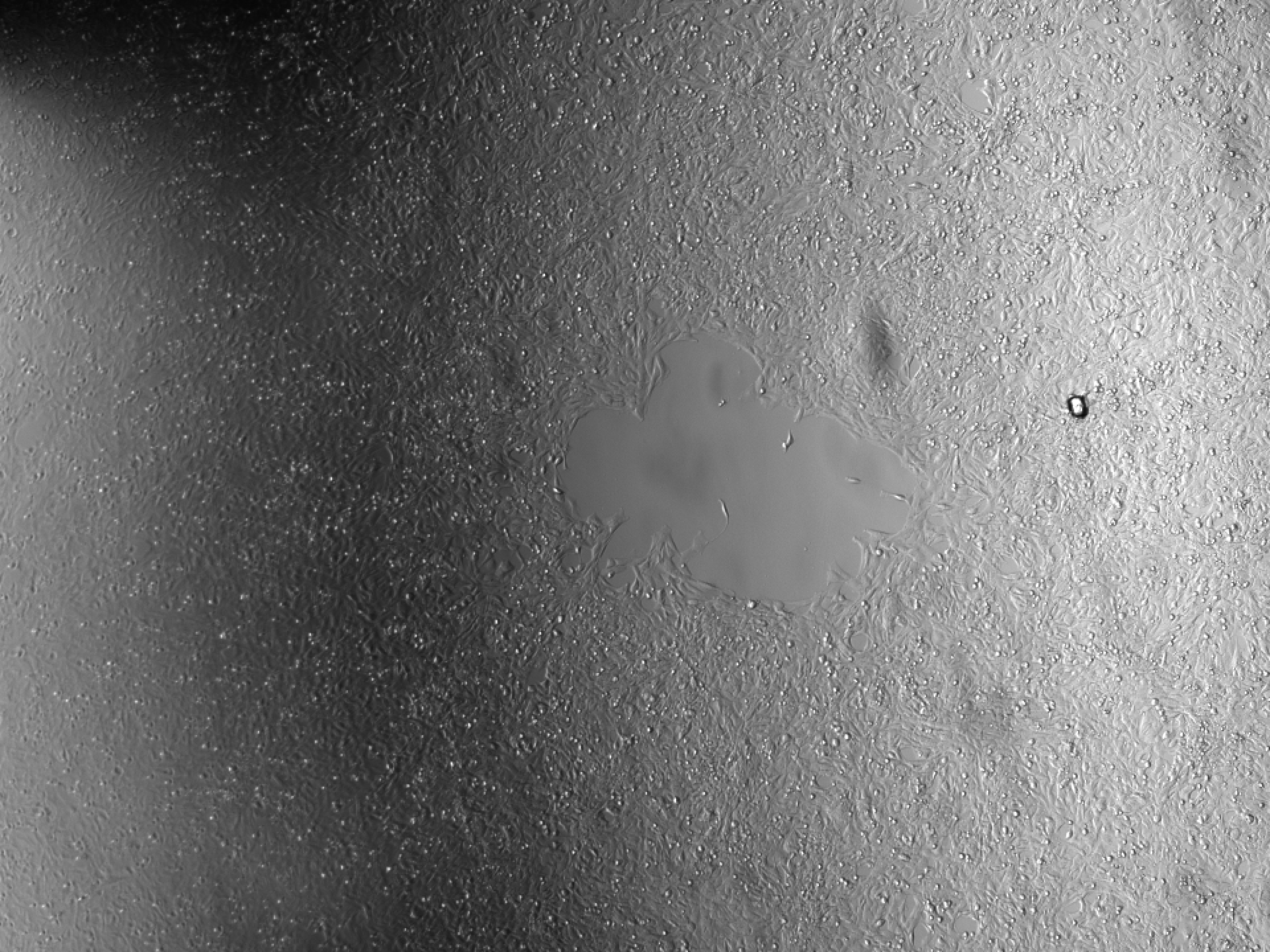

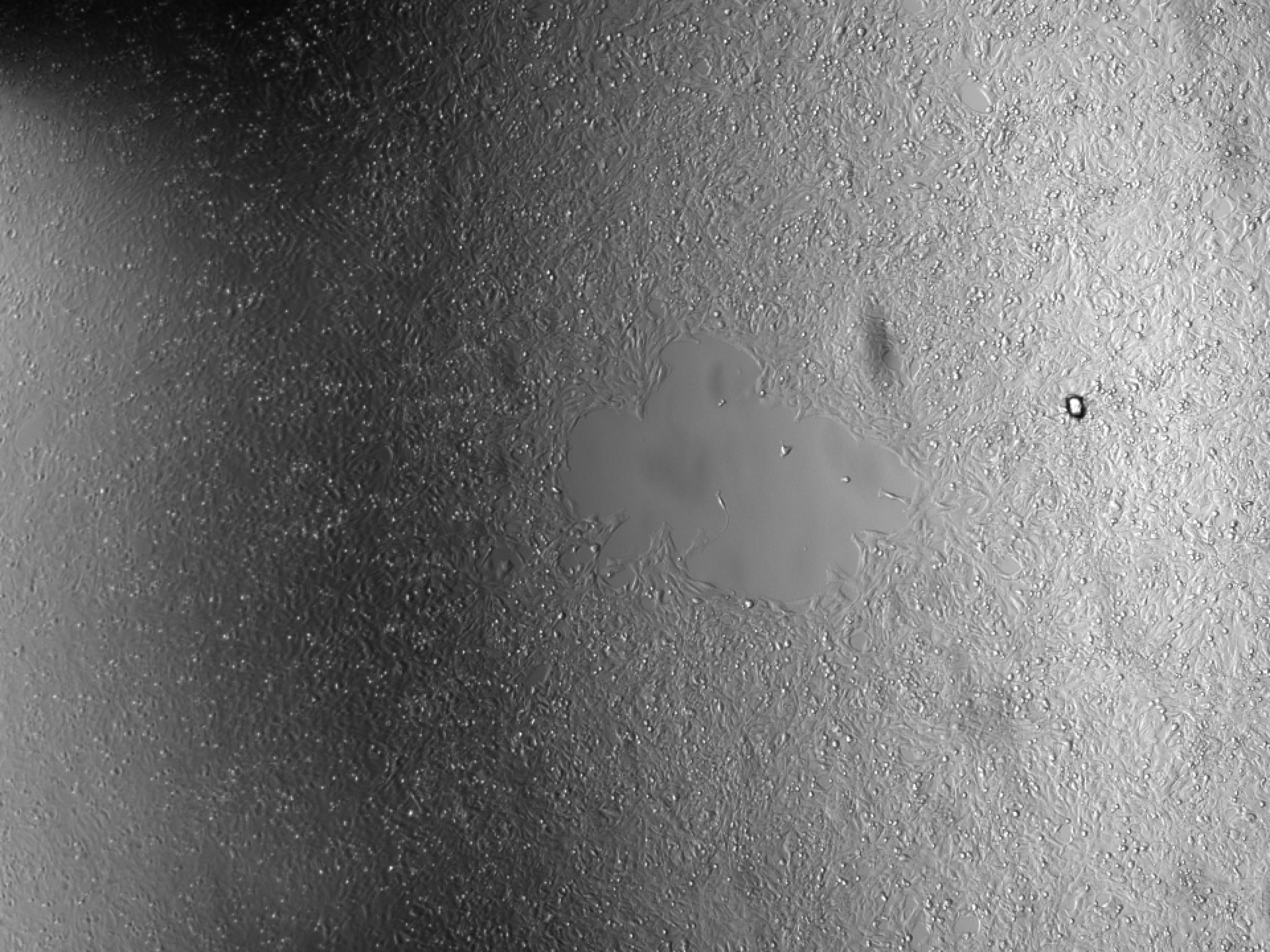

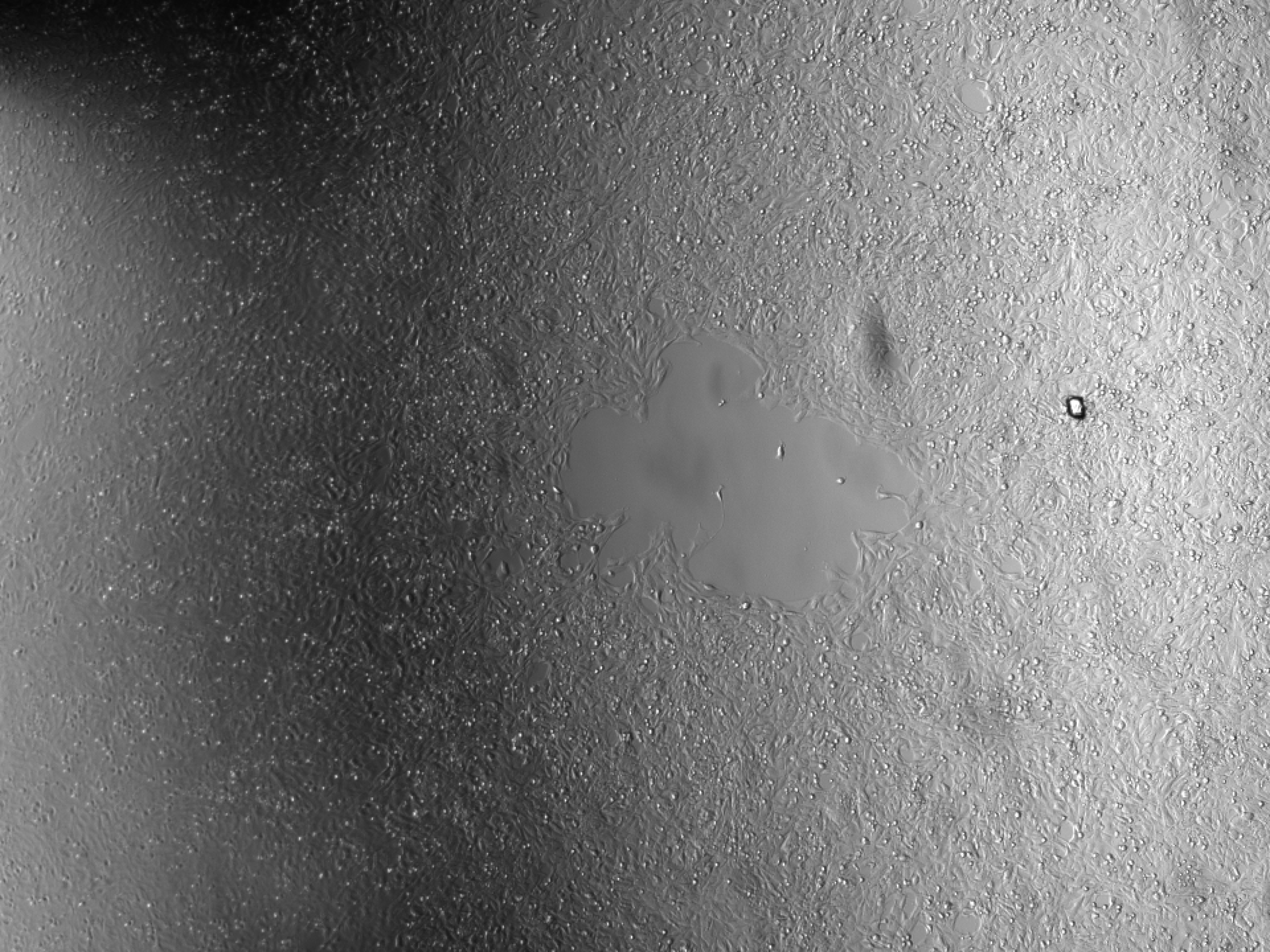

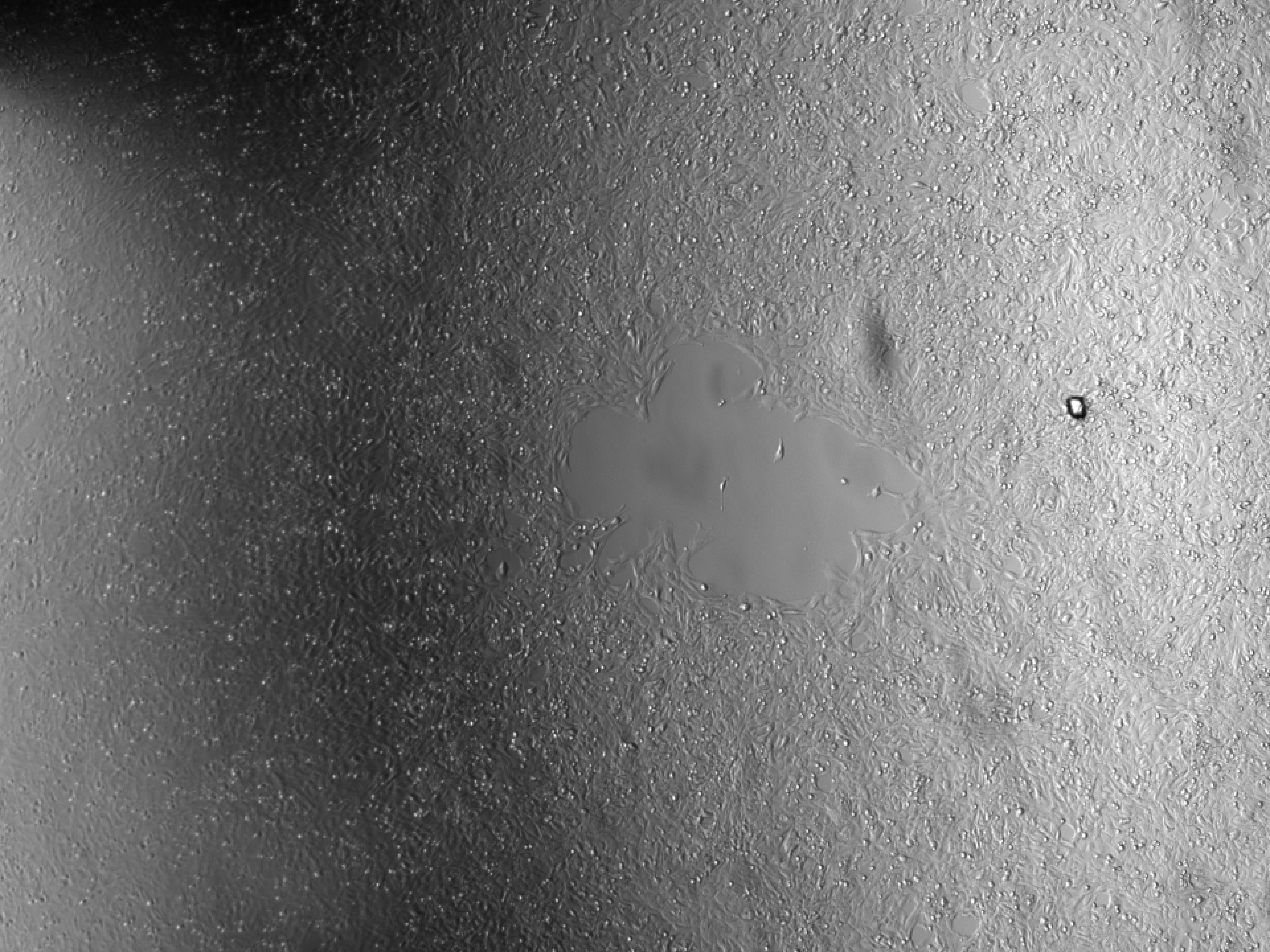

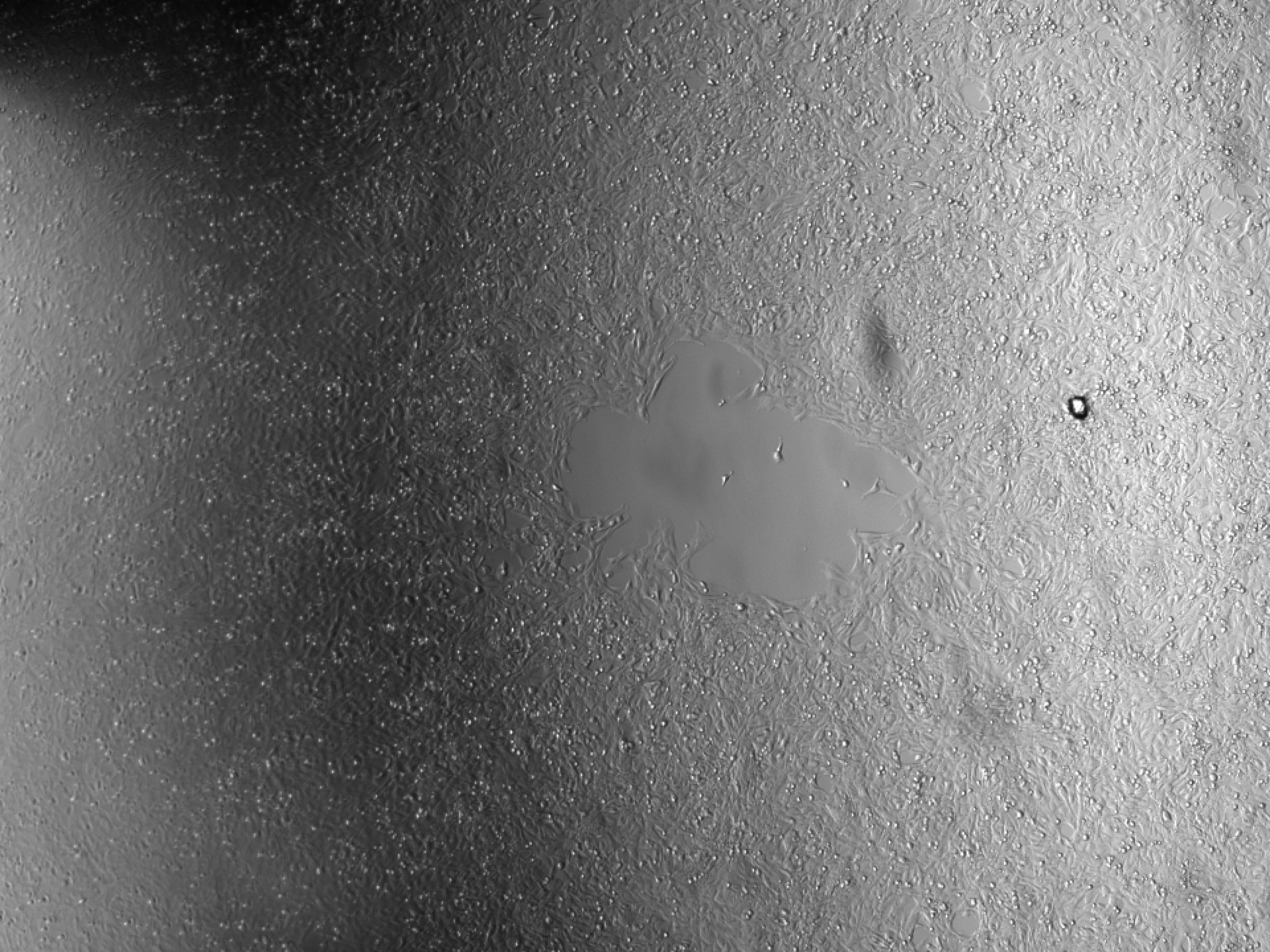

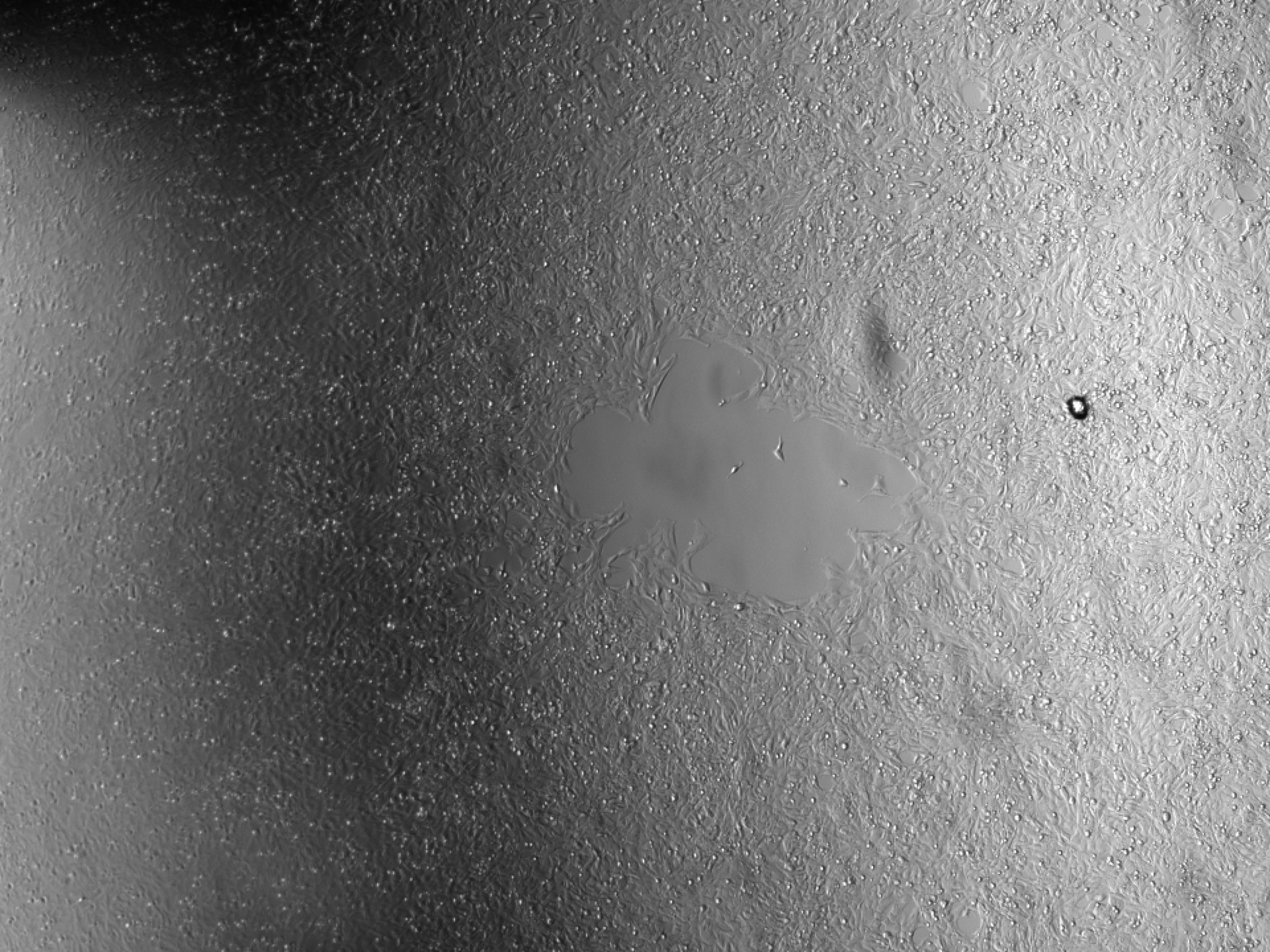

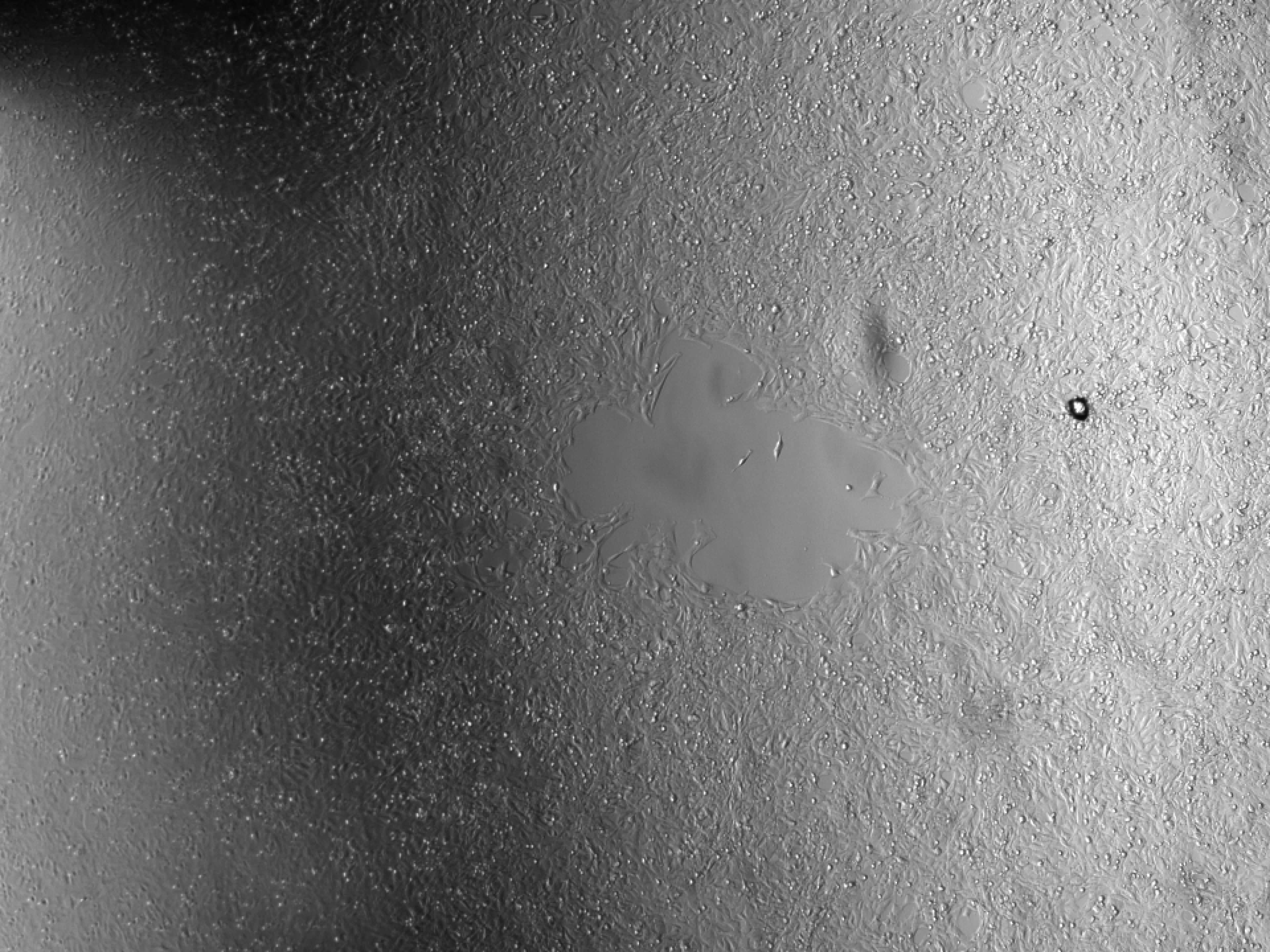

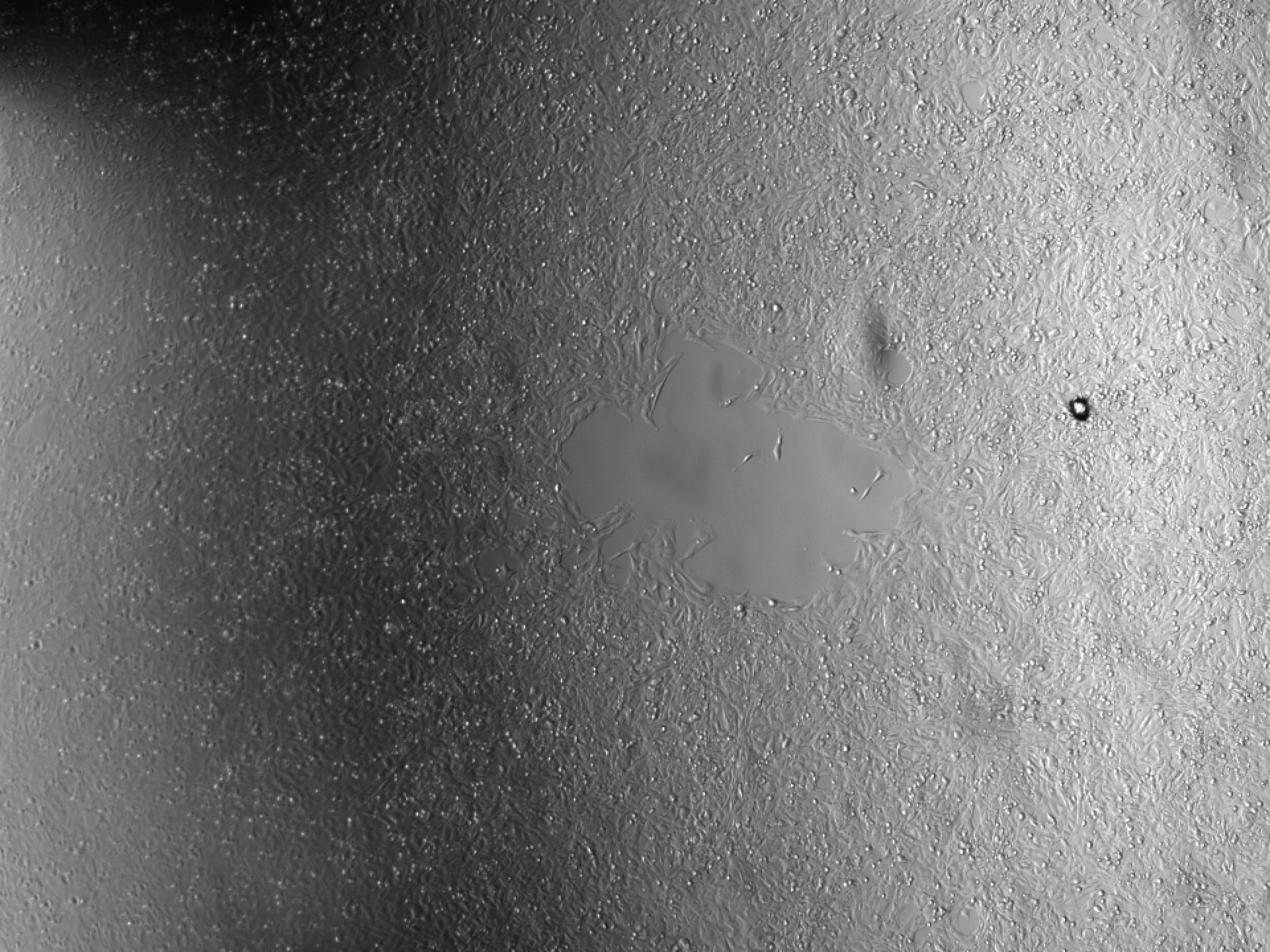

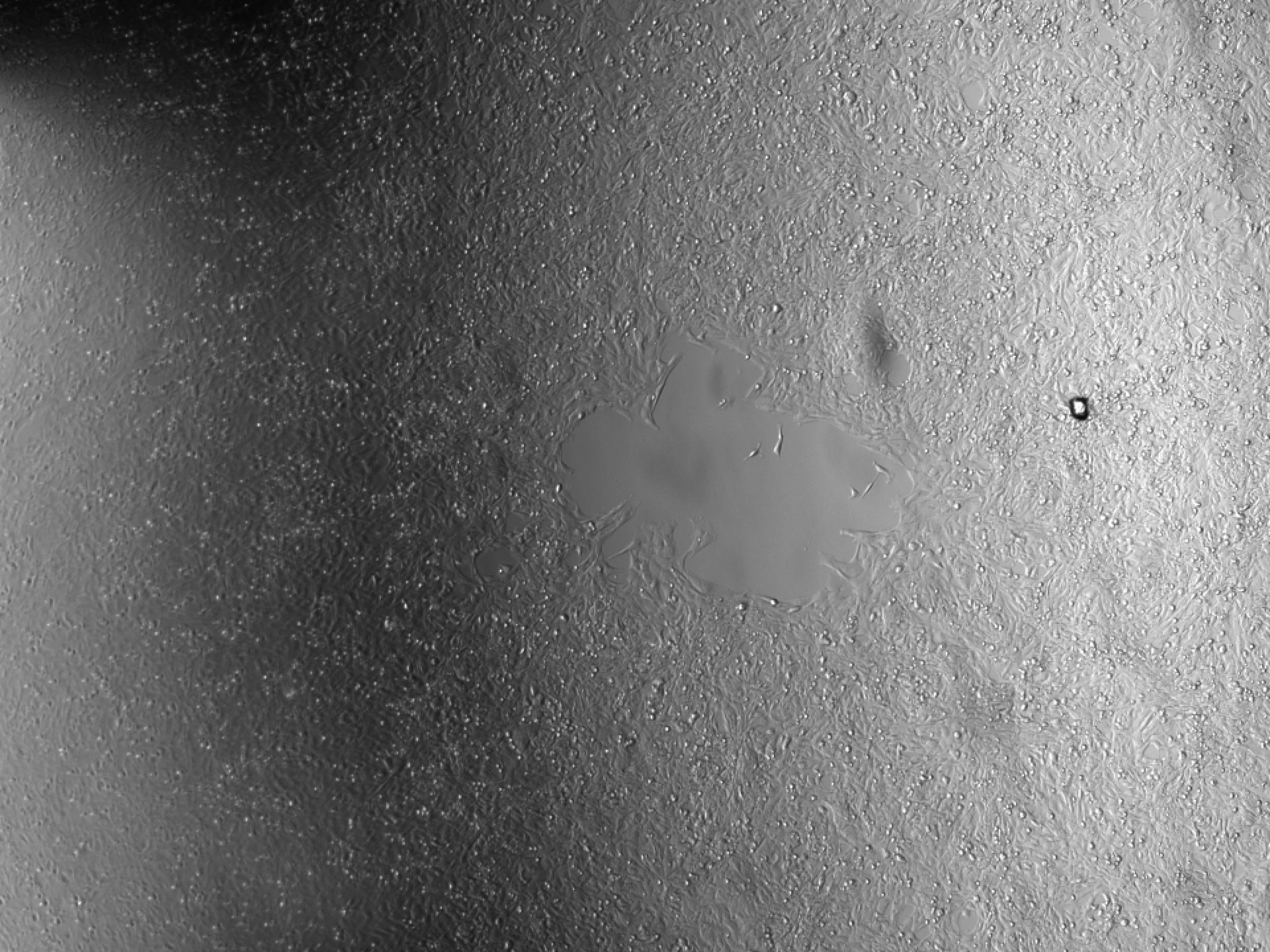

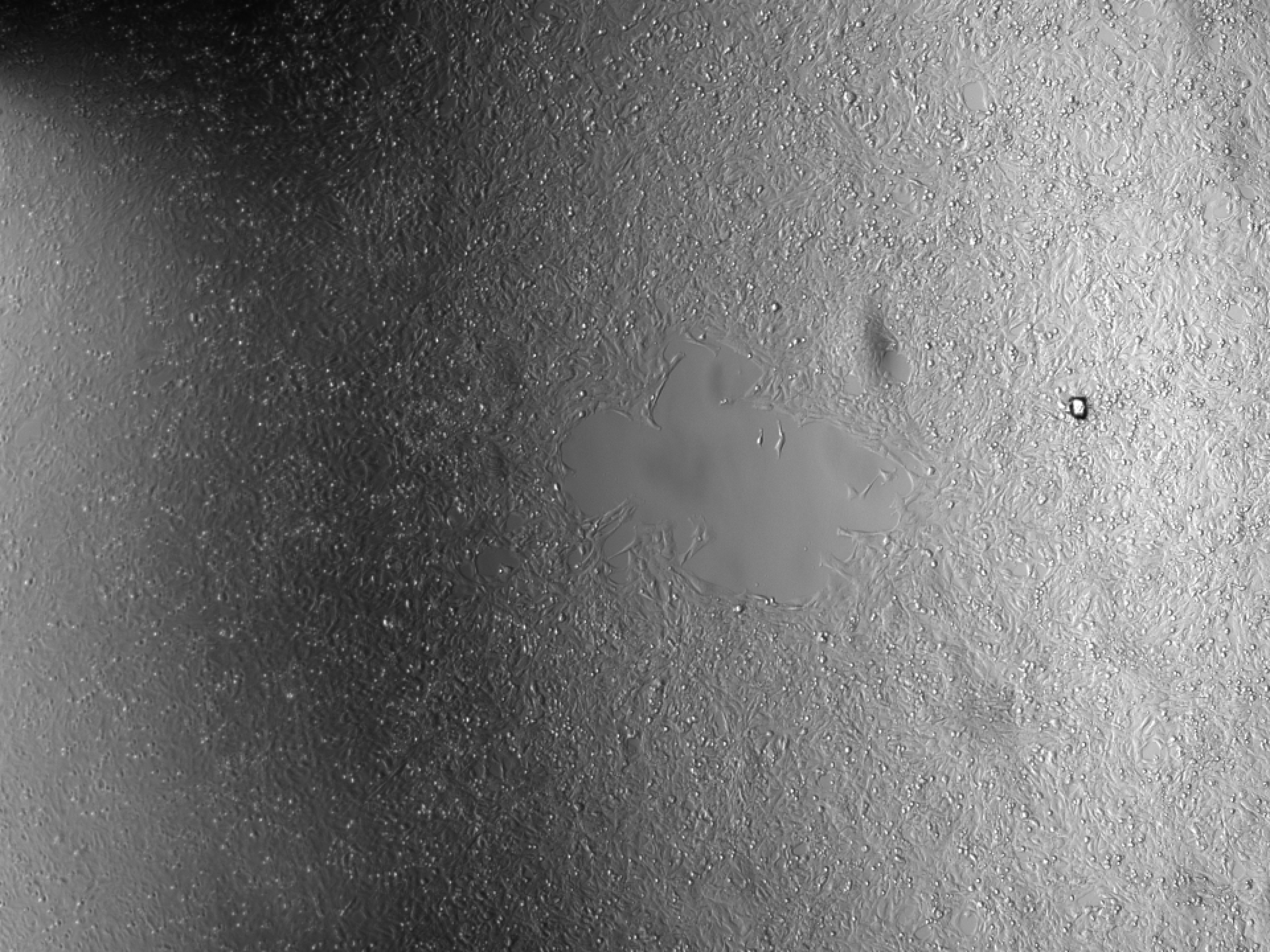

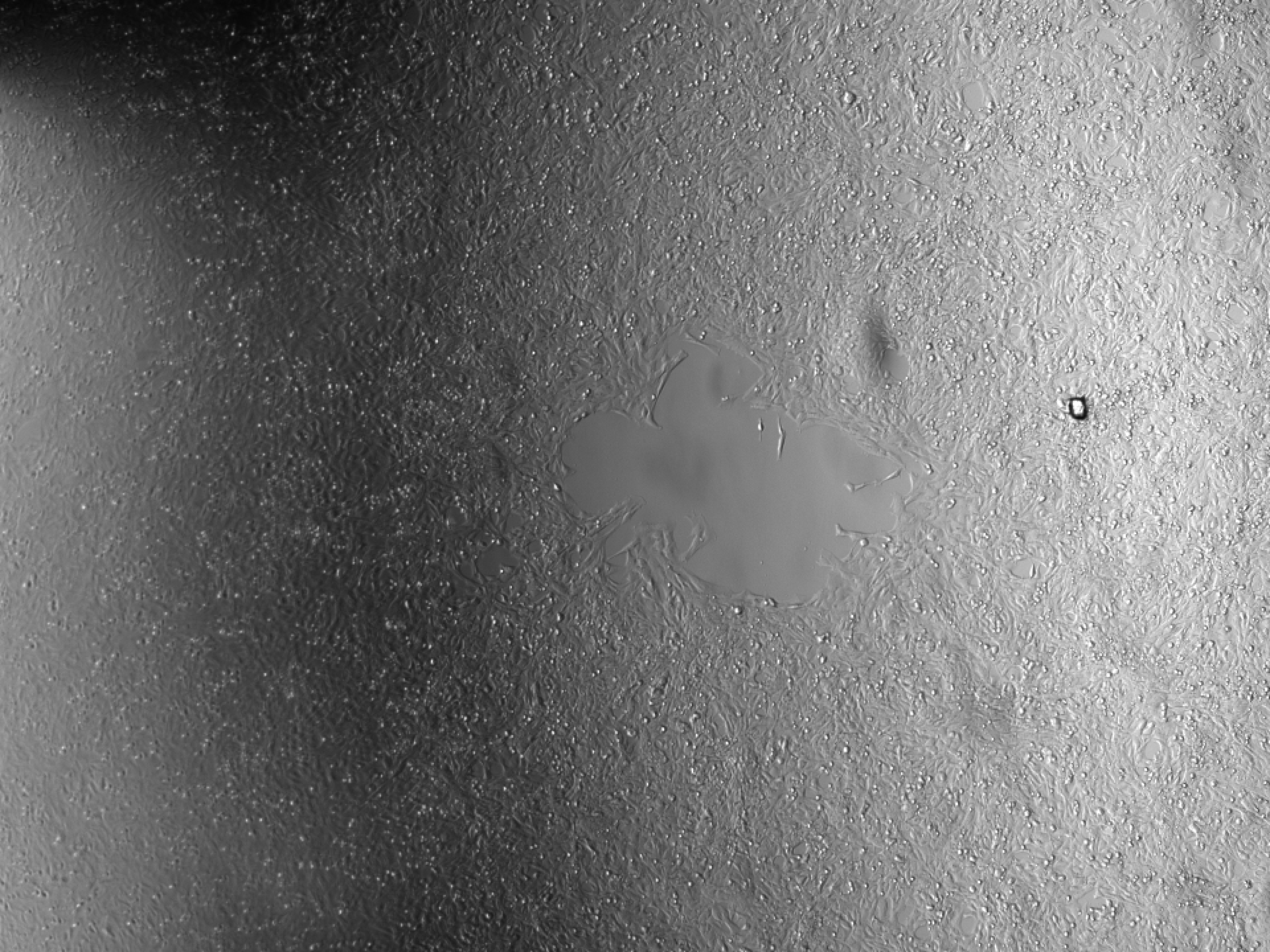

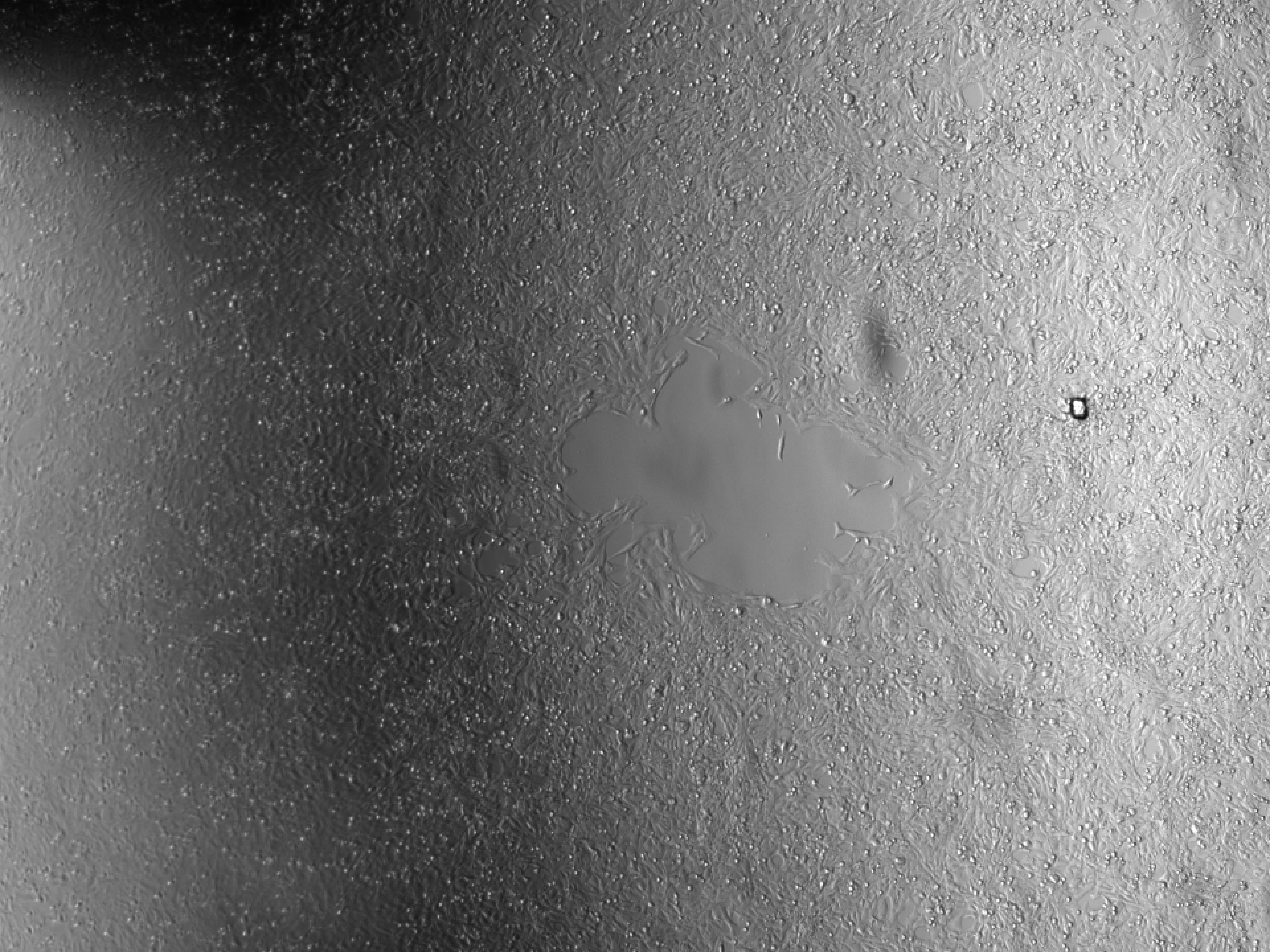

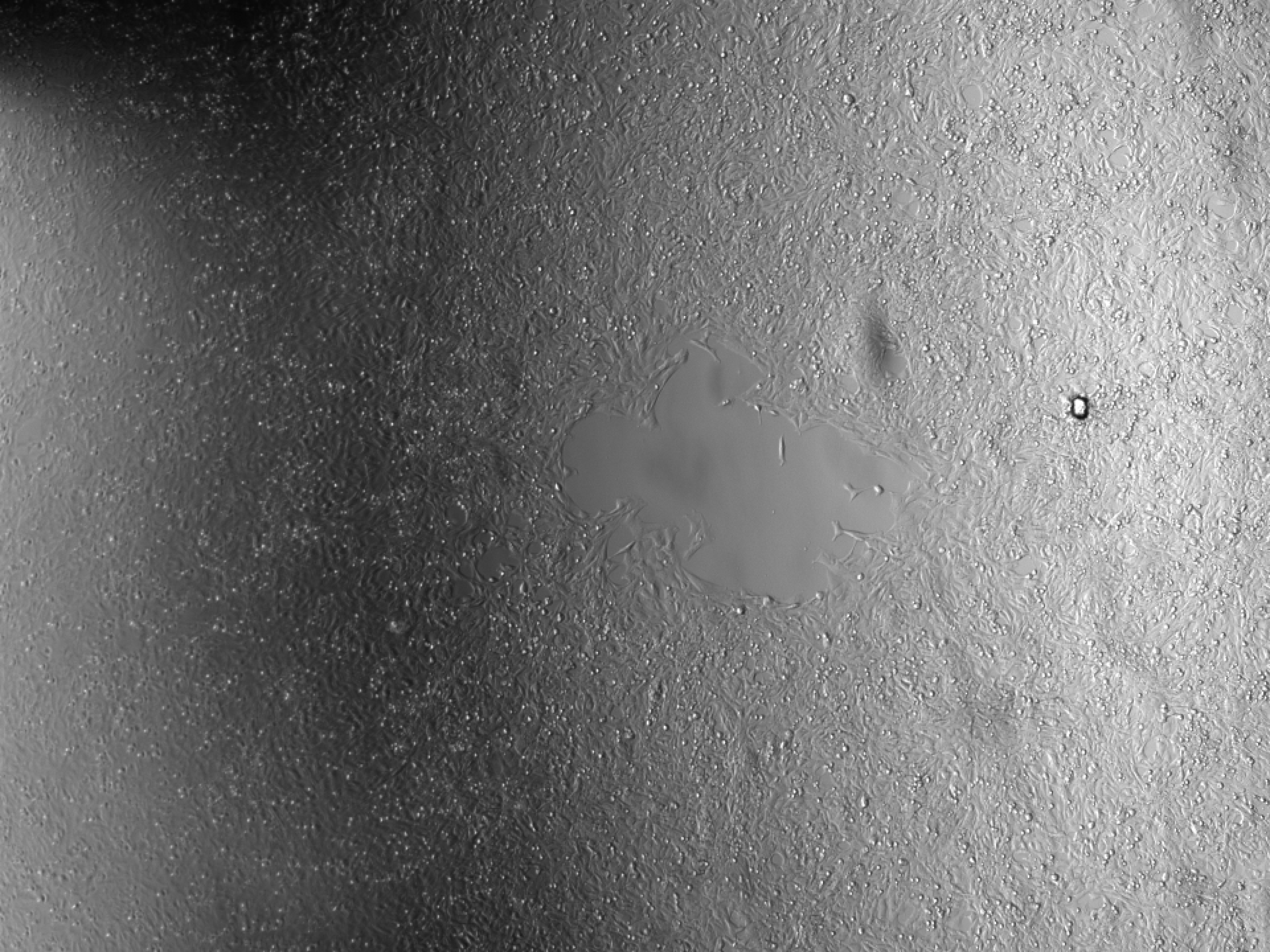

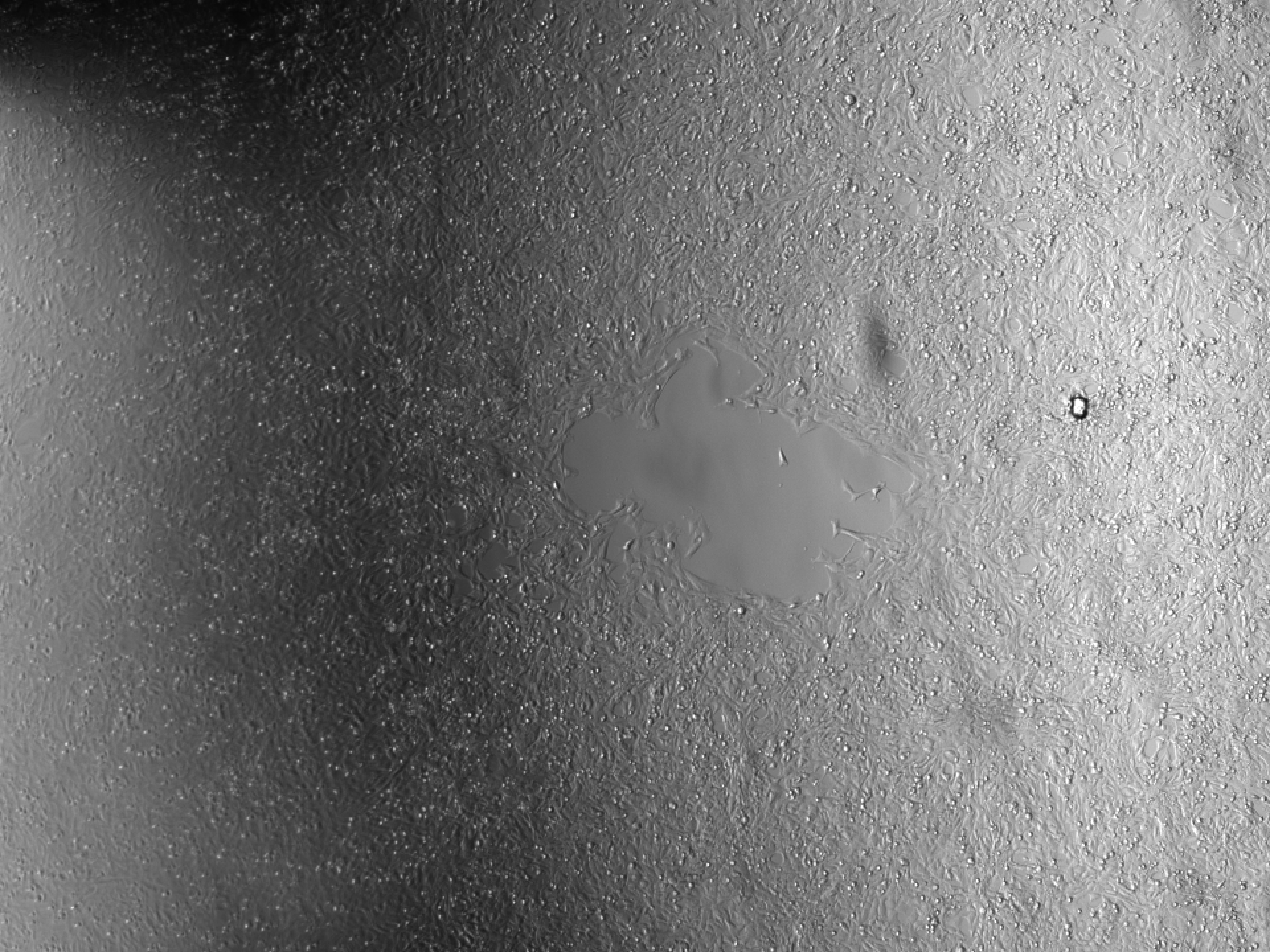

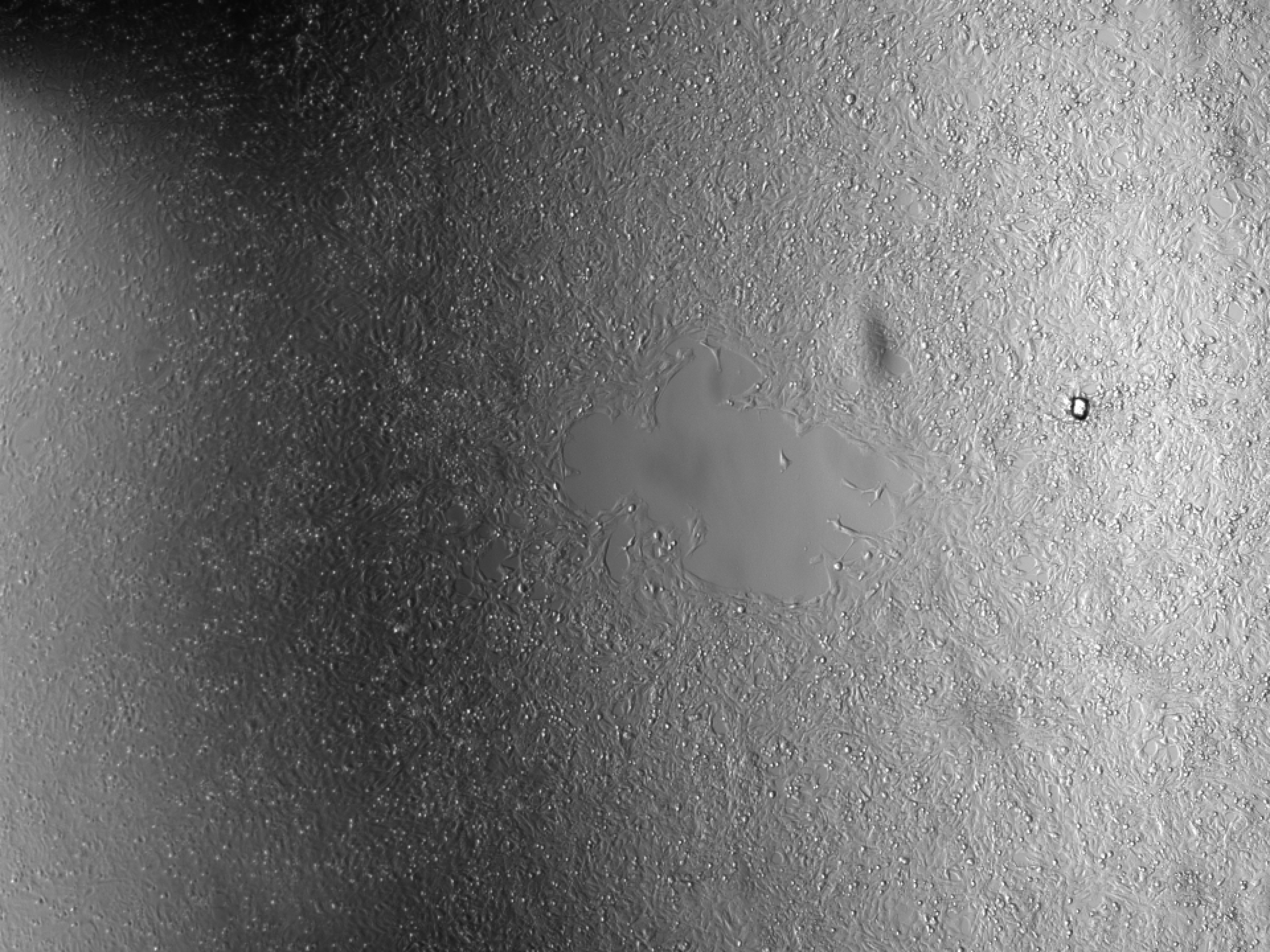

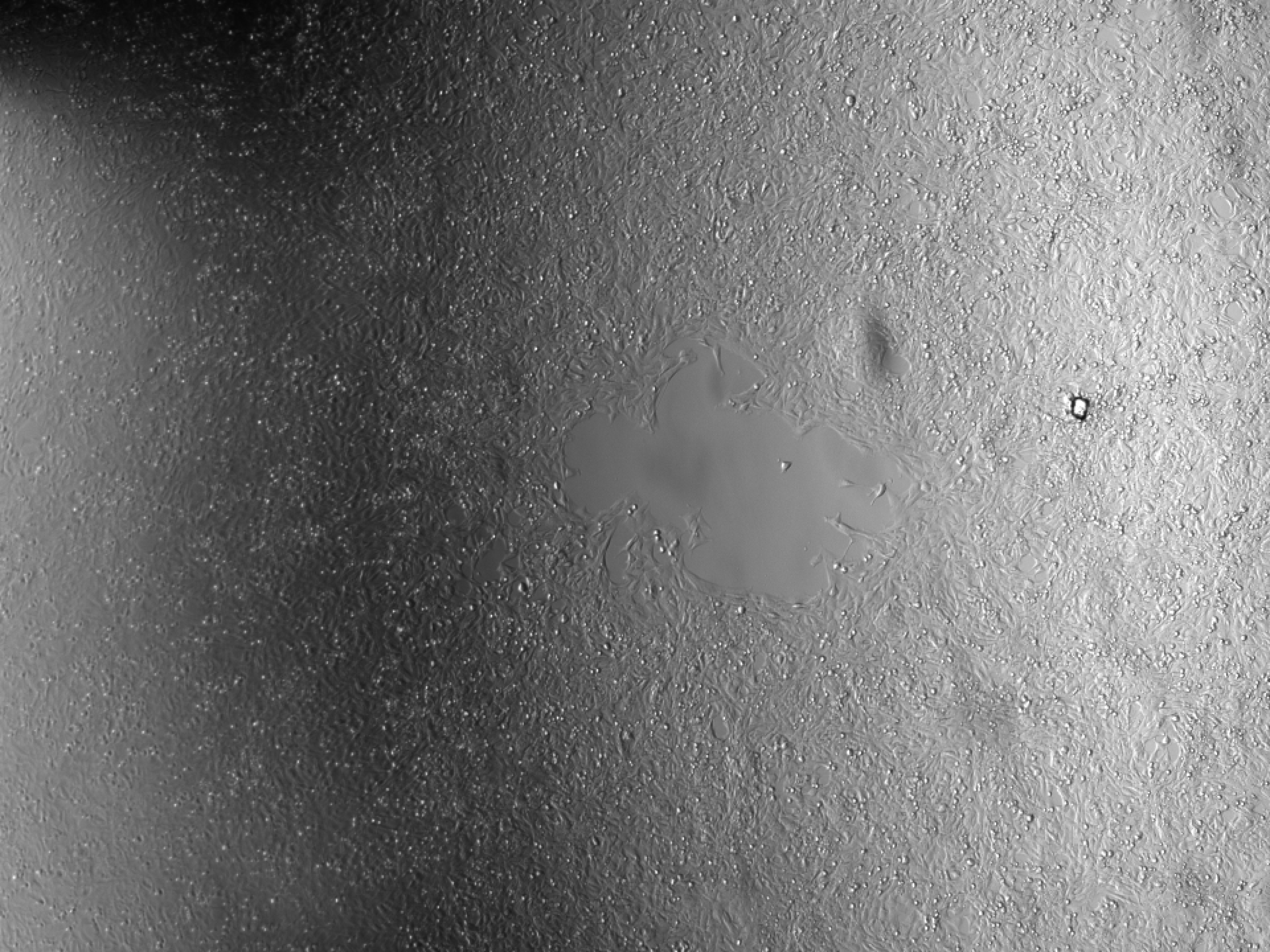

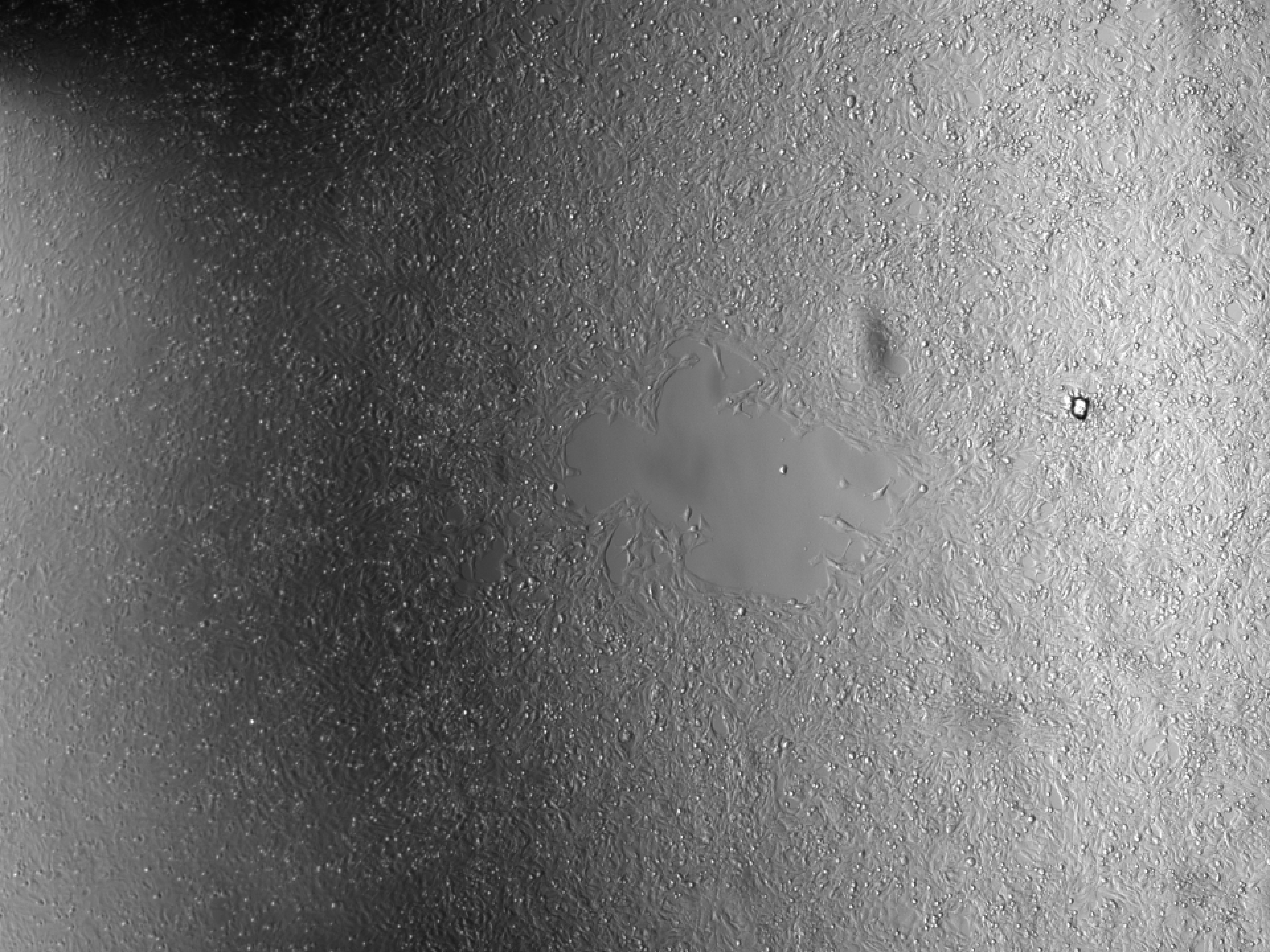

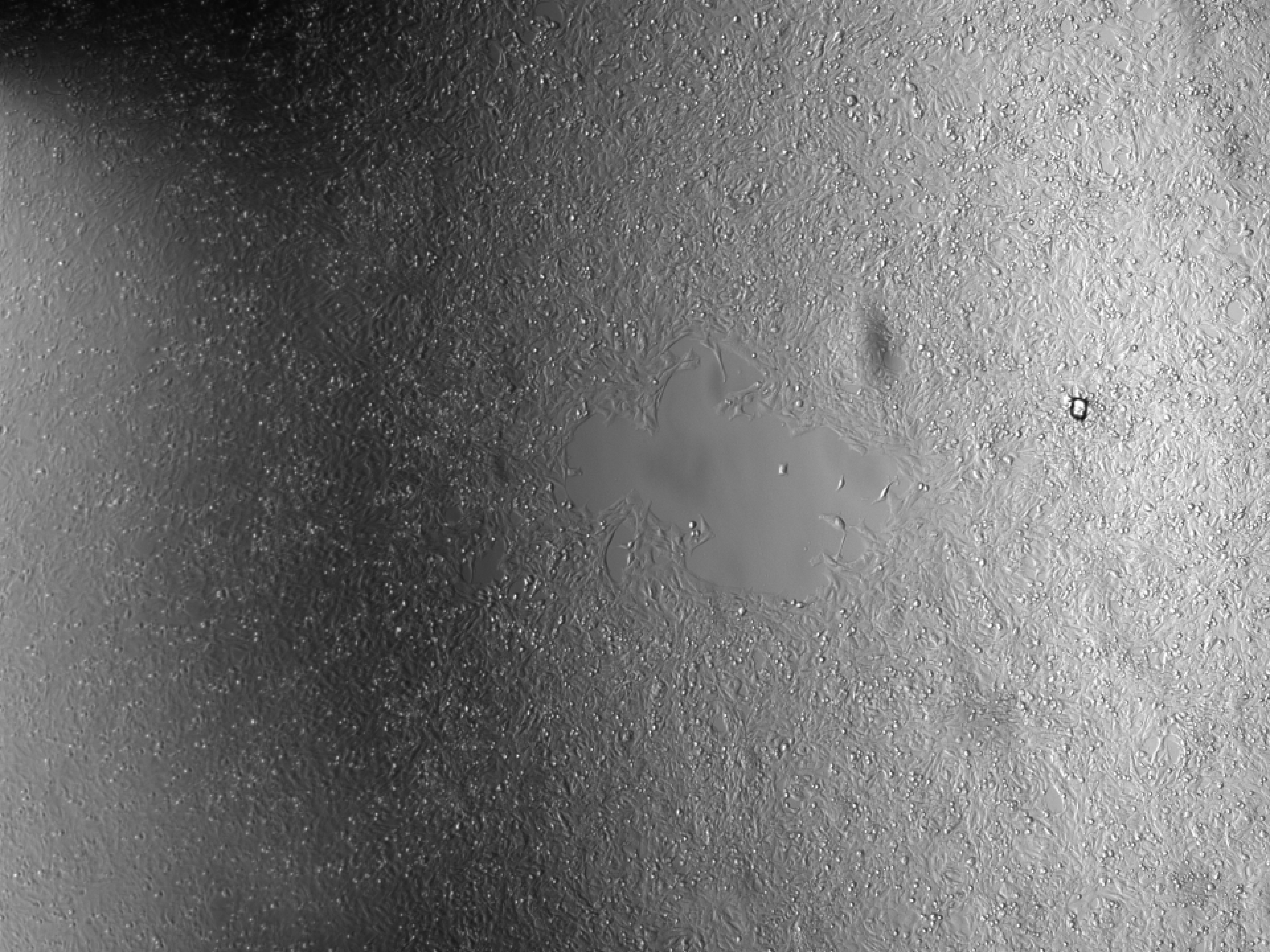

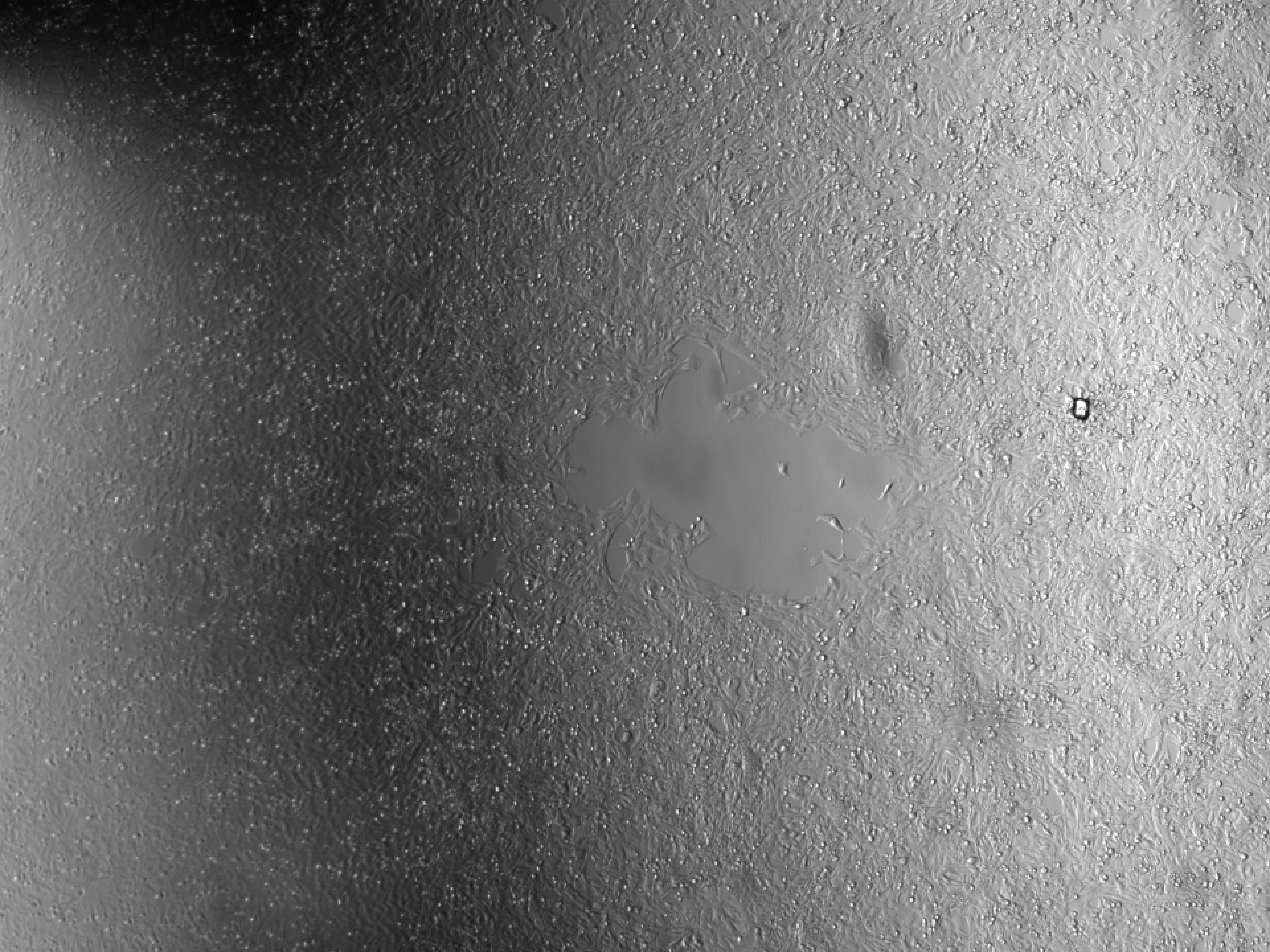

Supplement: Supplementary file 10 — Source Data for Expanded View and Movies [file EMMM-12-e12013-s014.zip › Source Data EV Movies.zip!/Source Data Movie EV2.zip!/Movie_EV2_Patient_BMP_CV2_100_ng_ml.pdf]
